# Safety and immunogenicity of a parenteral trivalent P2-VP8 subunit rotavirus vaccine: a multisite, randomised, double-blind, placebo-controlled trial

**DOI:** 10.1016/S1473-3099(20)30001-3

**Published:** 2020-07

**Authors:** Michelle J Groome, Lee Fairlie, Julie Morrison, Alan Fix, Anthonet Koen, Maysseb Masenya, Lisa Jose, Shabir A Madhi, Nicola Page, Monica McNeal, Len Dally, Iksung Cho, Maureen Power, Jorge Flores, Stanley Cryz

**Affiliations:** a South African Medical Research Council (SAMRC): Respiratory and Meningeal Pathogens Research Unit, Faculty of Health Sciences, University of the Witwatersrand, Johannesburg, South Africa; b Department of Science and Technology/National Research Foundation (DST/NRF): Vaccine Preventable Diseases, Faculty of Health Sciences, University of the Witwatersrand, Johannesburg, South Africa; c Wits Reproductive Health and HIV Institute, School of Clinical Medicine, Faculty of Health Sciences, University of the Witwatersrand, Johannesburg, South Africa; d Family Clinical Research Unit, Department of Paediatrics and Child Health, Stellenbosch University, Stellenbosch, South Africa; e PATH, Washington, DC, USA; f National Institute for Communicable Diseases, National Health Laboratory Service, Johannesburg, South Africa; g Department of Medical Virology, Faculty of Health Sciences, University of Pretoria, Pretoria, South Africa; h Department of Pediatrics, University of Cincinnati College of Medicine, Cincinnati, OH, USA; i Division of Infectious Diseases, Cincinnati Children's Hospital Medical Center, Cincinnati, OH, USA; j The Emmes Corporation, Rockville, MD, USA; k Novavax, Gaithersburg, MD, USA

## Abstract

**Background:**

A monovalent, parenteral, subunit rotavirus vaccine was well tolerated and immunogenic in adults in the USA and in toddlers and infants in South Africa, but elicited poor responses against heterotypic rotavirus strains. We aimed to evaluate safety and immunogenicity of a trivalent vaccine formulation (P2-VP8-P[4],[6],[8]).

**Methods:**

A double-blind, randomised, placebo-controlled, dose-escalation, phase 1/2 study was done at three South African research sites. Healthy adults (aged 18–45 years), toddlers (aged 2–3 years), and infants (aged 6–8 weeks, ≥37 weeks' gestation, and without previous receipt of rotavirus vaccination), all without HIV infection, were eligible for enrolment. In the dose-escalation phase, adults and toddlers were randomly assigned in blocks (block size of five) to receive 30 μg or 90 μg of vaccine, or placebo, and infants were randomly assigned in blocks (block size of four) to receive 15 μg, 30 μg, or 90 μg of vaccine, or placebo. In the expanded phase, infants were randomly assigned in a 1:1:1:1 ratio to receive 15 μg, 30 μg, or 90 μg of vaccine, or placebo, in block sizes of four. Participants, parents of participants, and clinical, data, and laboratory staff were masked to treatment assignment. Adults received an intramuscular injection of vaccine or placebo in the deltoid muscle on the day of randomisation (day 0), day 28, and day 56; toddlers received a single injection of vaccine or placebo in the anterolateral thigh on day 0. Infants in both phases received an injection of vaccine or placebo in the anterolateral thigh on days 0, 28, and 56, at approximately 6, 10, and 14 weeks of age. Primary safety endpoints were local and systemic reactions (grade 2 or worse) within 7 days and adverse events and serious adverse events within 28 days after each injection in all participants who received at least one injection. Primary immunogenicity endpoints were analysed in infants in either phase who received all planned injections, had blood samples analysed at the relevant timepoints, and presented no major protocol violations considered to have an effect on the immunogenicity results of the study, and included serum anti-P2-VP8 IgA, IgG, and neutralising antibody geometric mean titres and responses measured 4 weeks after the final injection in vaccine compared with placebo groups. This trial is registered with ClinicalTrials.gov, NCT02646891.

**Findings:**

Between Feb 15, 2016, and Dec 22, 2017, 30 adults (12 each in the 30 μg and 90 μg groups and six in the placebo group), 30 toddlers (12 each in the 30 μg and 90 μg groups and six in the placebo group), and 557 infants (139 in the 15 μg group, 140 in the 30 μg group, 139 in the 90 μg group, and 139 in the placebo group) were randomly assigned, received at least one dose, and were assessed for safety. There were no significant differences in local or systemic adverse events, or unsolicited adverse events, between vaccine and placebo groups. There were no serious adverse events within 28 days of injection in adults, whereas one serious adverse event occurred in a toddler (febrile convulsion in the 30 μg group) and 23 serious adverse events (four in placebo, ten in 15 μg, four in 30 μg, and five in 90 μg groups) occurred among 20 infants, most commonly respiratory tract infections. One death occurred in an infant within 28 days of injection due to pneumococcal meningitis. In 528 infants (130 in placebo, 132 in 15 μg, 132 in 30 μg, and 134 in 90 μg groups), adjusted anti-P2-VP8 IgG seroresponses (≥4-fold increase from baseline) to P[4], P[6], and P[8] antigens were significantly higher in the 15 μg, 30 μg, and 90 μg groups (99–100%) than in the placebo group (10–29%; p<0·0001). Although significantly higher than in placebo recipients (9–10%), anti-P2-VP8 IgA seroresponses (≥4-fold increase from baseline) to each individual antigen were modest (20–34%) across the 15 μg, 30 μg, and 90 μg groups. Adjusted neutralising antibody seroresponses in infants (≥2·7-fold increase from baseline) to DS-1 (P[4]), 1076 (P[6]), and Wa (P[8]) were higher in vaccine recipients than in placebo recipients: p<0·0001 for all comparisons.

**Interpretation:**

The trivalent P2-VP8 vaccine was well tolerated, with promising anti-P2-VP8 IgG and neutralising antibody responses across the three vaccine P types. Our findings support advancing the vaccine to efficacy testing.

**Funding:**

Bill & Melinda Gates Foundation.

**Research in context**

**Evidence before this study**

Live-attenuated, orally administered rotavirus vaccines have been introduced in more than 100 countries worldwide, leading to substantial reductions in diarrhoeal-related mortality and hospital admissions in young children. However, effectiveness of oral vaccines was shown to be lower in low-income and middle-income countries in Africa and Asia than in high-income countries. Challenges associated with globally available rotavirus vaccines in low-income countries include poor implementation, duration of protection beyond the first year of life, and the roles of maternal antibody, environmental enteric dysfunction, the gut microbiome, and host genetic factors. One strategy to address some of these challenges is the development of new vaccines, and several rotavirus vaccine candidates are in the pipeline, including parenterally administered, non-replicating rotavirus vaccines, which bypass the intestine and can potentially lead to an enhanced efficacy and safety profile. We searched the PubMed database for trials published in English between Jan 1, 2000, and Nov 20, 2019, using the terms “rotavirus”, “vaccine”, and “parenteral”. The most advanced candidate consists of a truncated VP8 subunit protein fused with the P2 epitope from tetanus toxin. A monovalent formulation of this vaccine (P2-VP8-P[8]) was well tolerated and immunogenic in South African infants when administered intramuscularly at 6, 10, and 14 weeks of age. However, although good immune responses were elicited to homologous P[8] strains of rotavirus, immune responses against heterologous P[4] and P[6] strains were poor, suggesting that a multivalent formulation might be required to provide protection against the common circulating rotavirus strains.

**Added value of this study**

We expanded the valency of the P2-VP8-P[8] monovalent vaccine to include P[4] and P[6] antigens, and this trivalent formulation (P2-VP8-P[4],[6],[8]) was evaluated for safety and immunogenicity in South African adults, toddlers, and infants. The vaccine was generally well tolerated in all participants, with neutralising antibody responses to P[4] and P[6] strains and IgG responses to P[4] and P[6] antigens in infants that were similar to responses to the Wa strain and P[8] antigen. In addition, we observed reduced faecal shedding of the Rotarix vaccine strain in the first week after challenge with Rotarix among infants who received the 90 μg dose of the trivalent P2-VP8 vaccine compared with infants who received placebo.

**Implications of all the available evidence**

Vaccinating infants against rotavirus disease using parenterally administered rotavirus vaccines could have several advantages over currently licensed live oral vaccines, such as improved protection against rotavirus disease in countries with high mortality from this disease, improved safety, potential for coformulation with other vaccines, and lower cost. The addition of antigens from P[4] and P[6] strains to the monovalent vaccine achieved high and similar IgG and neutralising antibody responses across all three strain types. Our results support further testing of this trivalent vaccine formulation, with a phase 3 study currently recruiting (NCT04010448).

## Introduction

The introduction of oral rotavirus vaccines into national immunisation programmes has led to substantial reductions in rotavirus and all-cause acute gastroenteritis hospital admissions and mortality among children younger than 5 years.^1^ However, because of reduced vaccine effectiveness and low vaccine coverage in some settings, rotavirus remains the leading cause of diarrhoea morbidity and mortality, accounting for an estimated 128 515 deaths worldwide in 2016.^2^ Live-attenuated oral rotavirus vaccines have shown lower efficacy and effectiveness in low-income and middle-income settings than in high-income settings, with effectiveness of 84–90% in countries with low child mortality compared with 47–57% in countries with high child mortality.^3^, ^4^ This disparity is not unique to oral rotavirus vaccines and has been observed for live oral cholera and polio vaccines.^5^, ^6^ Possible reasons for differences in the effectiveness of oral rotavirus vaccines include higher incidence of rotavirus infection in early life, malnutrition, and anti-rotavirus antibodies in breast milk in low-income settings, as well as host luminal, mucosal, and immune factors.^7^, ^8^, ^9^ Post-licensure studies in some countries showed a low-level risk of intussusception 1–7 days after the first or second dose of rotavirus vaccine administration, although the benefits of vaccination strongly outweigh this risk.^10^, ^11^ Studies from Africa have not shown an association between rotavirus vaccination and intussusception.^12^, ^13^

Rotavirus vaccine candidates using different strains, formulations, and routes of administration are in development.^14^ Parenterally administered, non-replicating rotavirus vaccines bypass the intestine and could lead to enhanced efficacy and safety profiles.^15^, ^16^ The most advanced candidate consists of a truncated VP8 subunit protein, which is derived from the cleavage of the rotavirus outer capsid spike protein VP4 (which defines the rotavirus P type) in the presence of trypsin, fused to the P2 epitope from tetanus toxin. The recombinant fusion protein is then expressed in *Escherichia coli*.^17^, ^18^ A monovalent formulation containing the VP8 subunit from a P[8] rotavirus strain (Wa strain) was well tolerated and immunogenic in adults in the USA, and in toddlers and infants in South Africa.^19^, ^20^ This vaccine elicited robust neutralising antibody responses to homologous P[8] strains but modest responses to heterologous P[4] and P[6] rotavirus strains. Following a challenge with Rotarix (GlaxoSmithKline, Belgium), an oral monovalent rotavirus vaccine, 1 month after the third dose of P2-VP8-P[8] vaccine, significantly fewer vaccinated infants than placebo recipients shed rotavirus, showing a potentially protective immune response mediated at the gut surface.^20^

A trivalent formulation of the vaccine, containing VP8 subunits from P[4], P[6], and P[8] strains, was developed to optimise responses to these three P types, which account for the majority of cases of severe rotavirus disease worldwide.^21^, ^22^ Our primary aim was to assess safety and tolerability of the trivalent vaccine at escalating dose levels in South African adults, toddlers, and infants and to evaluate the immunogenicity of three doses of the vaccine at different dose levels in infants compared with placebo.

## Methods

### Study design

This phase 1/2, double-blind, randomised, placebo-controlled, descending-age, dose-escalation trial consisted of a dose-escalation phase and an expanded phase. In the dose-escalation phase, 30 μg then 90 μg doses of the vaccine were assessed for safety, tolerability, and immunogenicity in adults, followed by assessment of the same doses in toddlers and 15 μg, 30 μg, and 90 μg doses in infants (appendix p 1). The expanded phase recruited additional infants and evaluated all three doses of the vaccine.

In the dose-escalation phase, progression from one dose to the next and from one age group to the next required review by a safety review committee of safety data up to 7 days following the first injection at each dose or in each age group (appendix pp 2–4). The expanded-cohort phase was done after completion of enrolment into the dose-escalation phase and safety assessment of each dose (appendix pp 7, 14).

The dose-escalation phase was done at the Respiratory and Meningeal Pathogens Research Unit (RMPRU; Johannesburg, South Africa), and the expanded-cohort phase was done at the RMPRU, the Wits Reproductive Health and HIV Institute Shandukani Research Centre (Johannesburg, South Africa), and the Family Clinical Research Unit (Cape Town, South Africa).

The protocol (appendix p 20) was approved by the Human Research Ethics Committee of the University of the Witwatersrand (Johannesburg, South Africa), Stellenbosch University Health Research Ethics Committee (Cape Town, South Africa), the Western Institutional Review Board (Puyallup, WA, USA), and the South African Health Products Regulatory Authority (Pretoria, South Africa), and was done under a US Food and Drug Administration investigational new drug application.

### Participants

Eligibility was assessed through medical history, physical examination, and screening laboratory tests. Healthy adults (aged 18–45 years), toddlers (aged 2–3 years), and infants (aged 6–8 weeks, ≥37 weeks' gestation, and without previous receipt of rotavirus vaccination), all without HIV infection, were eligible for enrolment. Exclusion criteria included acute illness, pregnant or breastfeeding women, presence of malnutrition or any systemic disorder that would compromise the participant's health or result in non-conformance to the protocol, congenital disorders, known or suspected impaired immunological function based on medical history and physical examination, immunoglobulin therapy or chronic immunosuppressant medications, a clinically significant screening laboratory value, HIV infection, and concurrent participation in another clinical trial. Full inclusion and exclusion criteria are listed in the appendix, pp 1–2. Investigators used their clinical judgment in considering a participant's overall fitness for inclusion in the trial. All adult participants were literate and provided written informed consent. Children were enrolled if their parents were literate and provided written informed consent. At RMPRU, adults were invited for screening by advertisements in the Soweto community, and parents of toddlers identified from hospital birth registers and infants identified in postnatal wards were invited to bring their children to RMPRU for screening. At the Family Clinical Research Unit, pregnant women were informed of the study at antenatal clinics and followed up at the obstetric unit and labour ward. At Shandukani Research Centre, pregnant women at antenatal clinics and mothers of infants attending the day 3 postnatal visit or 6-week vaccination visit were approached.

### Randomisation and masking

In the dose-escalation phase, adults and toddlers were randomly assigned to receive 30 μg or 90 μg of vaccine, or placebo; infants were randomly assigned to receive 15 μg, 30 μg, or 90 μg of vaccine, or placebo (appendix p 1). Permuted block randomisation was used throughout. Adults and toddlers were randomly assigned to receive the vaccine or placebo in groups of 15, using three blocks of five participants (four vaccine, one placebo) per group. Infants in the dose-escalation phase were randomly assigned in groups of 16: four blocks of four infants (three vaccine, one placebo) per group. In the expanded phase, infants were randomly assigned in a 1:1:1:1 ratio to the 15 μg, 30 μg, or 90 μg dose groups or placebo in block sizes of four. The randomisation sequence was computer-generated and maintained by the Statistical and Data Management Group at The Emmes Corporation (Rockville, MD, USA). A masked study investigator enrolled participants, who were then randomly assigned electronically. An unmasked pharmacist prepared and dispensed the injection, which was masked using an opaque sticker and administered by the masked study investigator. Participants, parents of participants, and clinical, data, and laboratory staff were masked to treatment assignment. Any deviation from the protocol was reported.

### Procedures

In the dose-escalation phase, adults received an intramuscular injection of vaccine or placebo in the deltoid on the day of randomisation (day 0), day 28, and day 56; toddlers received a single injection of vaccine or placebo in the anterolateral thigh on day 0. Infants in both the dose-escalation phase and the expanded phase received an injection of vaccine or placebo in the anterolateral thigh on day 0, day 28, and day 56, which roughly corresponded to ages 6, 10, and 14 weeks. The trivalent P2-VP8 vaccine was manufactured and supplied by the Walter Reed Army Institute of Research Pilot Bioproduction Facility (Silver Spring, MD, USA), as described previously.^19^ Vaccine, formulated as a sterile suspension containing 360 μg of protein—120 μg of each VP8 antigen derived from P[4] (DS-1), P[6] (1076), and P[8] (Wa) rotavirus strains—per mL adsorbed to aluminium hydroxide (Alhydrogel, Brenntag Biosector, Frederikssund, Denmark; 1·125 mg of aluminium per mL), was diluted with aluminium hydroxide diluent (1·125 mg/mL) within 6 h of administration to yield dose concentrations of 15 μg, 30 μg, and 90 μg per 0·5 mL containing 0·56 mg aluminium hydroxide. Sterile saline was used as placebo. In infants, two additional vaccines were given in the opposite thigh to the P2-VP8 vaccine or placebo: Hexaxim (Sanofi Pasteur, France), a diptheria, tetanus, pertussis, poliovirus, hepatitis B virus, and *Haemophilus influenzae* type B vaccine, was given at 6, 10, and 14 weeks of age; and Prevnar 13 (Pfizer, USA), a 13-valent pneumococcal conjugate vaccine, was given at 6 and 14 weeks of age. All infants in both phases received three oral doses of Rotarix, one each at 4, 8, and 12 weeks after the third study injection.

Participants were observed for 30 min after the administration of each injection. Local symptoms (injection site pain or tenderness, redness, swelling, and itching) and systemic symptoms (fever, headache, vomiting, nausea, fatigue, chills, and myalgia in adults, and fever, vomiting, poor appetite, irritability, and decreased activity in toddlers and infants) were recorded daily for 7 days following each injection. Clinic visits were done 7 days after each injection in adults, and 3 and 7 days after each injection in toddlers and infants. Unsolicited adverse events were recorded from randomisation until the final study visit, 6 months after the last injection. Adverse events were graded by investigators from mild (grade 1) to life threatening (grade 4) using a grading scale developed on the basis of the Division of AIDS Table for Grading the Severity of Adult and Pediatric Adverse Events, version 2.0, November 2014, of the US National Institutes of Health, with modifications to reflect local population norms (appendix pp 2–4). Safety data were reviewed by the safety review committee and an independent data safety and monitoring board periodically throughout the study.

Haemoglobin, white blood cell count, platelet count, total bilirubin, creatinine, and alanine transaminase were measured at baseline in all participants in both phases and 7 days after the first injection in the adult, toddler, and infant dose-escalation cohorts. Serum albumin was assessed at baseline only. A serum pregnancy test at screening and a urine pregnancy test before each injection was done for adult female participants. Serum was collected at baseline and 4 weeks after the final injection in all participants, as well as 4 weeks after the first and second injections in adults and 4 weeks after the second injection in infants. Anti-P2-VP8 IgG and IgA against P[4], P[6], and P[8] antigens were quantitated using standard ELISA assay techniques.^19^ Neutralising antibodies to DS-1 (G2P[4]), 1076 (G2P[6]), and Wa (G1P[8]) rotavirus strains were measured as previously described.^23^ Details of the serological testing are provided in appendix p 5. Serological testing was done at Division of Infectious Diseases, Cincinnati Children's Hospital Medical Center, Cincinnati, OH, USA. Stool was collected from infants 5, 7, and 9 days after the first dose of Rotarix in the subset of infants enrolled at the RMPRU and tested for the presence of rotavirus using the commercially available ProsPecT Rotavirus Microplate Assay (Oxoid, Ely, UK), according to the manufacturer's instructions. ELISA-positive specimens were confirmed and genotyped by PCR amplification of the *VP7* and *VP4* genes, as previously described.^20^ Stool testing was done at the National Institute for Communicable Diseases (Johannesburg, South Africa).

### Outcomes

The primary safety endpoints in all three age groups were the number of serious adverse events and adverse events up to 28 days after the last injection and the number of local and systemic reactions (grade 2 or worse) during the 7 days after vaccination in vaccine recipients compared with placebo recipients.

The primary immunogenicity endpoints were the proportion of infants with anti-P2-VP8 IgG and IgA seroresponses (at least a 4-fold increase in antibody titres between baseline and 4 weeks after the third study injection) for each of the three vaccine antigens (P[4], P[6], and P[8]); the proportion of infants with neutralising antibody responses (at least a 2·7-fold increase in antibody titres between baseline and 4 weeks after the third study injection) to each of the three rotavirus strains (DS-1, 1076, and Wa) from which the vaccine antigens were derived; the proportion of infants with neutralising antibody responses to at least two of the three strains from which the vaccine antigens were derived; and the change in geometric mean titres (GMTs) of anti-P2-VP8 IgG, IgA, and neutralising antibodies from baseline to 4 weeks after the third injection in infants. A 4-fold increase in neutralising antibody responses to each of the three rotavirus strains between baseline and 4 weeks after the third study injection was evaluated in infants as an exploratory endpoint.

Secondary safety endpoints were the number of serious adverse events and adverse events up to 6 months after the last vaccination. Secondary immunogenicity endpoints were anti-P2-VP8 IgG, IgA, and neutralising antibody responses and GMTs between baseline and 4 weeks after the second injection in infants or after the final injection in adults and toddlers (appendix p 6). Assessments of immunogenicity in adults at timepoints other than 4 weeks after the final injection were exploratory. Also as an exploratory endpoint, we assessed the proportion of infants enrolled at the RMPRU site who tested rotavirus-positive on an ELISA stool test at any time (5, 7, or 9 days) after administration of the first dose of Rotarix.

### Statistical analysis

For the adult and toddler cohorts, 12 vaccine recipients per dose provided a greater than 90% chance of observing an adverse event that had a frequency of 17·5%, and 24 vaccine recipients for the two doses combined provided a greater than 90% chance of observing an adverse event that had a frequency of 9·2%. In the infant cohorts, the 150 vaccine recipients planned per dose provided a greater than 90% chance of observing an adverse event that had a frequency of 1·6%, and the 450 vaccine recipients planned for the three dose groups combined provided a greater than 90% chance of observing an adverse event that had a frequency of 0·5%. On the basis of the results in South African infants who received monovalent P2-VP8 vaccine or placebo, the strain-specific seroresponse rates were expected to be 80% or more for at least one of the three P2-VP8 vaccine doses and less than 20% for the placebo group.^20^ For the infant cohort, 135 evaluable vaccine recipients per dose (assuming 10% loss of study participants due to drop-out) provided at least 74% power (Fisher's exact test) to detect a difference of 15 percentage points and at least 95% power to detect a difference of 20 percentage points in seroresponse rates between any two dose groups (ie, assuming true rates of 65% *vs* 80% and 60% *vs* 80%), and at least 99% power to detect a difference of 30 percentage points or more between a vaccine group and the combined placebo groups.

Safety was analysed by treatment received, and the safety population included all participants in the dose-escalation and expansion cohorts who were randomly assigned and received at least one dose of vaccine or placebo. Immunogenicity was assessed in the per-protocol population, which included all randomly assigned participants who received all planned injections, had blood samples analysed at the relevant timepoints, and presented no major protocol violations considered to have an effect on the immunogenicity results of the study.

Categorical results (serum IgA, IgG, and neutralising antibody seroresponses) are presented as frequency, proportion (%), and exact two-sided binomial (Clopper-Pearson) 95% CI. Continuous outcomes (serum IgA, IgG, and neutralising antibody responses) are presented as GMT and two-sided 95% CI obtained from the t-distribution on log-transformed titres. To compare safety outcomes between treatment groups, we used Fisher's exact two-tailed test, or χ^2^ test if the expected number of events was sufficient (all expected cell frequencies equal to five or more). The binomial immunogenicity response variables were compared between each dose group and placebo with logistic regression, and p values from prespecified pairwise comparisons are reported (if there was a significant difference overall between groups, then pairwise comparison was done). Continuous immunogenicity variables (GMTs) were modelled by ANOVA.

To adjust for decay in maternal antibody occurring concurrently with IgG and neutralising antibody immune responses to the vaccine in infants, we did an analysis of the adjusted seroresponse rates using the exponential decay function based on the estimated half-life (for each immunogenicity assay separately) of maternal antibodies in infants in the placebo group who had detectable baseline titres that were higher than at the post-injection visit. Adjusted seroresponse was defined as at least a 4-fold increase in titre between baseline and 4 weeks after the third injection (adjusted titre) in infants with an unadjusted post-injection titre greater than the limit of detection. Shedding of Rotarix virus was assessed for each of the three specified post-vaccination days (5, 7, or 9 days) and for any of the three days. Proportions of infants with shedding were compared between the placebo group and each dose group and between the placebo group and all dose groups combined using Fisher's exact test. Data were analysed with SAS software (version 9.3), and statistical significance was defined as a two-tailed p value of less than 0·05. This trial is registered with ClinicalTrials.gov, NCT02646891.

### Role of the funding source

The funder of the study had no role in study design, data collection, data analysis, data interpretation, or writing of the report. The corresponding author had full access to all the data in the study and had final responsibility for the decision to submit for publication.

## Results

**Figure 1:**
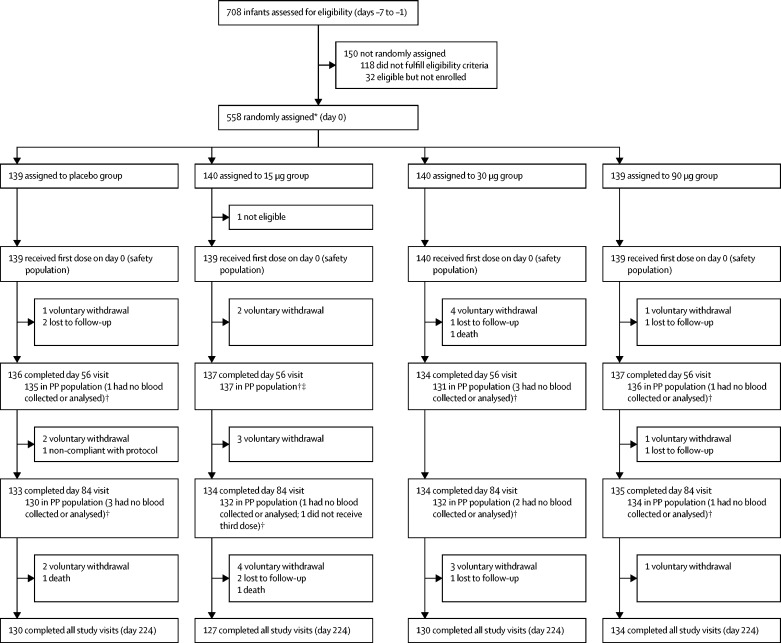
Trial profile for infants in the dose-escalation and expanded cohorts PP=per protocol. *254 at the Respiratory and Meningeal Pathogens Research Unit, 168 at Wits Shandukani Research Centre, and 136 at the Family Clinical Research Unit. †PP population included participants who received two doses and had immunogenicity data at baseline and day 56 (for secondary immunogenicity analyses) or who received all three doses and had immunogenicity data at baseline and day 84 (for the primary immunogenicity analysis). ‡The infant who did not receive the third dose because they were ineligible attended the day 56 follow-up visit, had a blood sample collected, and was included in the day 56 PP population.

Participants were enrolled and followed up from Feb 15, 2016, to Dec 22, 2017. In the dose-escalation phase, 30 adults (12 each in the 30 μg and 90 μg groups and six in the placebo group), 30 toddlers (12 each in the 30 μg and 90 μg groups and six in the placebo group), and 48 infants (12 each in the 15 μg, 30 μg, 90 μg, and placebo groups) were randomly assigned to receive vaccine or placebo (figure 1; appendix pp 15–16). 510 infants (128 each in the 15 μg and 30 μg groups and 127 each in the 90 μg and placebo groups) were randomly assigned to vaccine or placebo in the expanded phase, giving a total of 558 infant participants across both study phases (figure 1). 549 (98%) infants were black, 285 (51%) were boys, and the mean age of infants was 48 days (SD 3·5). Enrolment was stratified by site, but different enrolment rates resulted in some imbalance in the number enrolled at each site (figure 1). Because of the onset of the rotavirus season around July, consistent with the protocol-specified avoidance of that period because of the potential confounding effect of wild-type infection, enrolment was stopped before the specified sample size was reached. However, the number of infants enrolled was sufficient to address study objectives (appendix p 6). Demographic characteristics were similar across treatment groups for adults, toddlers, and infants (table 1; appendix p 8).

**Table 1:** Baseline characteristics for the infant population in both the dose-escalation and expansion phases

		**15 μg (n=140)**	**30 μg (n=140)**	**90 μg (n=139)**	**Placebo (n=139)**
Age (days)		48·7 (3·5)	49·0 (3·7)	48·9 (3·2)	48·8 (3·7)
Sex					
	Male	74 (53%)	64 (46%)	75 (54%)	72 (52%)
	Female	66 (47%)	76 (54%)	64 (46%)	67 (48%)
Race					
	Black	138 (99%)	138 (99%)	136 (98%)	137 (99%)
	Mixed race	2 (1%)	2* (1%)	3 (2%)	2 (1%)
Length (cm)		54·9 (2·5)	55·1 (2·2)	55·3 (2·0)	55·4 (2·4)
Weight (kg)		4·8 (0·7)	4·8 (0·5)	4·9 (0·6)	4·9 (0·6)

Data are n (%) for sex and race and mean (SD) for age, length, and weight.

* Includes one infant with unspecified race.

30 adults and 30 toddlers received at least one dose of vaccine or placebo and were included in the safety assessment. The highest severity of solicited local and systemic reactions and unsolicited adverse events in adults was grade 2 (all occurring in vaccine recipients within 28 days of injection); the majority of events in adults were mild (appendix p 8). The highest severity of solicited local and systemic reactions and unsolicited adverse events up to 28 days post-injection for toddlers was grade 2 (two in the 30 μg group had local reactions, one in the placebo group had a systemic reaction, and one in each of the 30 μg and 90 μg groups had unsolicited adverse events); the majority of events were mild (appendix p 8). No serious adverse events were reported in adults and one unsolicited serious adverse event was reported in a toddler in the 30 μg group within 28 days of injection (febrile convulsion of moderate severity on day 21 post-injection, which resolved without sequelae; appendix p 8).

Among 557 infants who received at least one dose of vaccine or placebo, the highest severity of solicited local reactions was grade 2, which occurred in similar proportions of infants across the four groups (p=0·23; table 2). 44 (32%) infants in the 15 μg group, 30 (21%) in the 30 μg group, 42 (30%) in the 90 μg group, and 30 (22%) in the placebo group had grade 2 systemic reactions or worse (p=0·091; table 2). One infant in the 15 μg group had grade 4 fever (axillary temperature 41·3°C) on the day of the third injection, which resolved rapidly without other symptoms or sequelae. One infant in the 15 μg group and two infants in the 30 μg group had grade 3 fever, two infants in the 15 μg group had grade 3 vomiting, and one infant in the 15 μg group and two infants in the 90 μg group had grade 3 irritability; all were solicited reactions within 7 days of injection. The proportion of infants who had an unsolicited adverse event of grade 2 or worse within 28 days after injection was similar across the groups (p=0·74; table 2). 23 unsolicited serious adverse events (four in the placebo group, ten in the 15 μg group, four in the 30 μg group, and five in the 90 μg group) occurred in 20 (4%) of 557 infants within 28 days after injection. No serious adverse events were assessed as related to the study product. One infant in the 30 μg group was admitted to hospital 20 days after the first injection with pneumococcal meningitis and died 7 days later. Two other infant deaths were reported more than 28 days after the third injection (one in the placebo group died from sudden infant death syndrome and one in the 15 μg group died from bronchiolitis); all deaths were assessed as unrelated to the study vaccine. 43 (31%) infants in the 15 μg group, 28 (20%) in the 30 μg group, 36 (26%) in the 90 μg group, and 27 (19%) in the placebo group had grade 2 unsolicited adverse events or worse (p=0·080; table 2) up to 6 months of age. Details of adverse events are provided in the appendix (pp 9–10).

**Table 2:** Number of infants with solicited and unsolicited adverse events (maximum severity per participant)

	**15 μg**	**30 μg**	**90 μg**	**Placebo**	**p value**
**Grade 1 (mild) solicited local reactions within 7 days after injection**					
After injection 1	66/139 (48%)	84/140 (60%)	76/139 (55%)	67/139 (48%)	..
After injection 2	49/139 (35%)	58/136 (43%)	64/139 (46%)	59/135 (44%)	..
After injection 3	51/135 (38%)	53/133 (40%)	58/137 (42%)	49/133 (37%)	..
Overall	83/139 (60%)	96/140 (69%)	92/139 (66%)	84/139 (60%)	..
Grade 2 or higher solicited local reactions within 7 days after injection*					
After injection 1	10/139 (7%)	3/140 (2%)	9/139 (7%)	6/139 (4%)	0·21
After injection 2	6/139 (4%)	6/136 (4%)	3/139 (2%)	2/135 (2%)	0·38
After injection 3	8/135 (6%)	5/133 (4%)	6/137 (4%)	2/133 (2%)	0·31
Overall	19/139 (14%)	13/140 (9%)	16/139 (12%)	9/139 (7%)	0·23
**Grade 1 (mild) solicited systemic reactions within 7 days after injection**					
After injection 1	77/139 (55%)	77/140 (55%)	64/139 (46%)	75/139 (54%)	..
After injection 2	61/139 (44%)	51/136 (38%)	57/139 (41%)	61/135 (45%)	..
After injection 3	52/135 (39%)	55/133 (41%)	55/137 (40%)	56/133 (42%)	..
Overall	70/139 (50%)	83/140 (59%)	73/139 (53%)	86/139 (62%)	..
**Grade 2 or higher solicited systemic reactions within 7 days after injection**†					
After injection 1	23/139 (17%)	18/140 (13%)	26/139 (19%)	18/139 (13%)	0·45
After injection 2	17/139 (12%)	13/136 (10%)	15/139 (11%)	15/135 (11%)	0·92
After injection 3	17/135 (13%)	11/133 (8%)	15/137 (11%)	11/133 (8%)	0·57
Overall	44/139 (32%)	30/140 (21%)	42/139 (30%)	30/139 (22%)	0·091
**Unsolicited adverse events within 28 days after injection**					
Any	118/139 (85%)	122/140 (87%)	124/139 (89%)	119/139 (86%)	0·72
Grade 2 or higher	24/139 (17%)	18/140 (13%)	20/139 (14%)	19/139 (14%)	0·74
Injection related (any)‡	2/139 (1%)	3/140 (2%)	2/139 (1%)	3/139 (2%)	1·0§
Injection related (grade 2 or higher)‡	0/139 (0%)	1/140 (1%)	1/139 (1%)	1/139 (1%)	1·0§
Serious adverse event	8/139 (6%)	4/140 (3%)	4/139 (3%)	4/139 (3%)	0·83§
**Unsolicited adverse events up to 6 months after the last injection**					
Any	129/139 (93%)	134/140 (96%)	130/139 (94%)	132/139 (95%)	0·72
Grade 2 or higher	43/139 (31%)	28/140 (20%)	36/139 (26%)	27/139 (19%)	0·080
Injection related (any)‡	2/139 (1%)	3/140 (2%)	3/139 (2%)	3/139 (2%)	1·0§
Injection related (grade 2 or higher)‡	0/139 (0%)	1/140 (1%)	1/139 (1%)	1/139 (1%)	1·0§
Serious adverse event	13/139 (9%)	6/140 (4%)	8/139 (6%)	8/139 (6%)	0·35

Data are n/N (%) unless otherwise indicated. p values refer to the difference between all four groups and are from χ^2^ test unless otherwise indicated.

* The highest severity for local reactions was grade 2 (moderate).

† One infant in the 15 μg group had grade 4 fever (axillary temperature 41·3°C) on the day of the third injection, which resolved rapidly without other symptoms or sequelae; one infant in the 15 μg group and two infants in the 30 μg group had grade 3 fever; two infants in the 15 μg group had grade 3 vomiting; one infant in the 15 μg group and two infants in the 90 μg group had grade 3 irritability; all other adverse events were grade 2.

‡ Assessed by the site investigator as having a reasonable possibility that the study injection caused the event; one adverse event in each of the placebo, 30 μg, and 90 μg groups were deemed to be of moderate (grade 2) severity, whereas all others adverse events were mild (grade 1), and no serious adverse event was considered to be related to the study injection.

§ Fisher's exact test.

528 infants (130 in the placebo group, 132 in the 15 μg group, 132 in the 30 μg group, and 134 in the 90 μg group) received three injections of vaccine or placebo, had a serum sample collected at baseline and 4 weeks after the final injection, and were included in the per-protocol primary immunogenicity analysis (figure 1). For each of the three antigens, anti-P2-VP8 IgG GMTs and adjusted seroresponses in infants 4 weeks after the third dose were significantly higher in each dose group than in the placebo group; unadjusted results were similar to the adjusted results (table 3). Anti-P2-VP8 IgG adjusted seroresponses to all three antigens were observed in 131 (99%) infants in the 15 μg group, 130 (99%) in the 30 μg group, 134 (100%) in the 90 μg group, and 11 (9%) in the placebo group (p<0·0001 for all pairwise comparisons between vaccine groups and the placebo group).

**Table 3:** Serum IgA and IgG antibody responses and GMTs pre-vaccination and 4 weeks after the third injection of trivalent P2-VP8 or placebo in infants, according to treatment group

		**15 μg**	**30 μg**	**90 μg**	**Placebo**	p value*
**Anti-P2-VP8 IgG**						
P[4]						
	Number of observations	132	132	134	130	..
	Pre-vaccination GMT	87 (69–109)	62 (48–79)	76 (62–94)	73 (57–94)	..
	Post-vaccination GMT	3888 (3313–4562)	4205 (3692–4789)	5244 (4729–5815)	28 (22–36)	<0·0001
	p value†	<0·0001	<0·0001	<0·0001	..	..
	Unadjusted seroresponse	123 (93%; 87–97)	122 (92%; 87–96)	131 (98%; 94–100)	9 (7%; 3–13)	<0·0001
	Adjusted seroresponse‡	131 (99%; 96–100)	130 (99%; 95–100)	134 (100%; 97–100)	16 (12%; 7–19)	<0·0001
	p value§	<0·0001	<0·0001	<0·0001	..	..
P[6]						
	Number of observations	132	132	134	130	..
	Pre-vaccination GMT	78 (62–97)	63 (49–82)	68 (54–86)	65 (51–84)	..
	Post-vaccination GMT	14 028 (11980–16426)	14 724 (13181–16447)	17 085 (15295–19085)	50 (39–64)	<0·0001
	p value†	<0·0001	<0·0001	<0·0001	..	..
	Unadjusted seroresponse	131 (99%; 96–100)	130 (99%; 95–99)	132 (99%; 95–100)	18 (14%; 8–21)	<0·0001
	Adjusted seroresponse‡	131 (99%; 96–100)	132 (100%; 97–100)	134 (100%; 97–100)	38 (29%; 22–38)	<0·0001
	p value§	<0·0001	<0·0001	<0·0001	..	..
P[8]						
	Number of observations	132	132	134	130	..
	Pre-vaccination GMT	93 (74–117)	74 (59–94)	81 (65–100)	86 (67–110)	..
	Post-vaccination GMT	5365 (4554–6321)	5792 (5086–6595)	7088 (6314–7958)	30 (23–39)	<0·0001
	p value†	<0·0001	<0·0001	<0·0001	..	..
	Unadjusted seroresponse	123 (93%; 87–97)	124 (94%; 88–97)	131 (98%; 94–100)	8 (6%; 3–12)	<0·0001
	Adjusted seroresponse‡	131 (99%; 96–100)	130 (99%; 95–100)	134 (100%; 97–100)	13 (10%; 5–16)	<0·0001
	p value§	<0·0001	<0·0001	<0·0001	..	..
**Anti-P2-VP8 IgA**						
P[4]						
	Number of observations	130	132	134	130	..
	Pre-vaccination GMT	5 (5–6)	5 (5–5)	5 (5–6)	5 (5–5)	..
	Post-vaccination GMT	13 (10–17)	12 (10–15)	10 (9–12)	7 (5–8)	<0·0001
	p value†	<0·0001	<0·0001	0·0024	..	..
	Unadjusted seroresponse¶	35 (27%; 20–35)	40 (30%; 23–39)	32 (24%; 17–32)	13 (10%; 5–16)	0·0002
	p value§	0·0004	<0·0001	0·0024	..	..
P[6]						
	Number of observations	131	131	134	130	..
	Pre-vaccination GMT	5 (5–6)	5 (4–5)	5 (5–6)	5 (4–5)	..
	Post-vaccination GMT	12 (10–15)	13 (10–15)	12 (10–14)	7 (6–8)	<0·0001
	p value†	<0·0001	<0·0001	<0·0001	..	..
	Unadjusted seroresponse¶	37 (28%; 21–37)	44 (34%; 26–42)	34 (25%; 18–34)	13 (10%; 5–16)	<0·0001
	p value§	0·0001	<0·0001	0·0009	..	..
P[8]						
	Number of observations	129	130	131	130	..
	Pre-vaccination GMT	5 (4–5)	5 (4–5)	5 (5–5)	5 (4–5)	..
	Post-vaccination GMT	11 (9–13)	9 (8–11)	8 (7–10)	6 (5–7)	<0·0001
	p value†	<0·0001	0·0004	0·0048	..	..
	Unadjusted seroresponse¶	36 (28%; 20–36)	26 (20%; 14–28)	28 (21%; 15–29)	11 (9%; 4–15)	0·0005
	p value§	<0·0001	0·0070	0·0030	..	..

Data are n, GMT (95% CI), or n (%; 95% CI), unless otherwise indicated. GMT=geometric mean titre.

* Overall p value of the difference between all groups in GMTs and proportions of infants with seroresponse.

† p value indicates pairwise comparison of each vaccine dose group with the placebo group in GMT increase from pre-vaccination to post-vaccination; titres were adjusted for maternal antibodies. Significant pairwise differences were observed between the vaccine groups, as follows: IgG P[4] p=0·040 (30 μg *vs* 15 μg) and p=0·034 (90 μg *vs* 15 μg); IgG P[8] p=0·036 (90 μg *vs* 15 μg); IgA P[8] p=0·017 (90 μg *vs* 15 μg).

‡ IgG titres after injection were adjusted for decay in maternal antibodies (half-life was 36·6 days for [P4], 49·1 days for [P6], and 38·3 days for [P8]). Adjusted seroresponse was defined as ≥4-fold increase in titre between baseline and 4 weeks after the third injection (adjusted titre) in infants with an unadjusted post-injection titre greater than the limit of detection.^15^

§ p value indicates pairwise comparison of each vaccine dose group to placebo in adjusted seroresponse (IgG) or unadjusted seroresponse (IgA); no significant pairwise between-group differences were observed among the active dose groups.

¶ IgA seroresponse was defined as ≥4-fold increase in titre between baseline and 4 weeks after the third injection in infants with a post-injection titre greater than the limit of detection.^9^

For each of the three antigens, anti-P2-VP8 IgA seroresponses after the third dose were significantly higher in each dose group than in the placebo group (table 3). Proportions of infants with anti-P2-VP8 IgA seroresponses to any two antigens were significantly higher in each vaccine dose group than in the placebo group (11 [9%] of 130): 34 (26%) of 130 in the 15 μg group (p=0·0001), 35 (27%) of 132 in the 30 μg group (p<0·0001), and 29 (22%) of 134 in the 90 μg group (p=0·0024). Seroresponses to all three antigens were observed in 19 (15%) of 128 infants in the 15 μg group, 16 (12%) of 129 in the 30 μg group, 16 (12%) of 131 in the 90 μg group, and seven (5%) of 130 in the placebo group; there was no difference between treatment groups (p=0·065). No significant differences between active dose groups were observed for anti-P2-VP8 IgG or IgA seroresponses.

**Figure 2:**
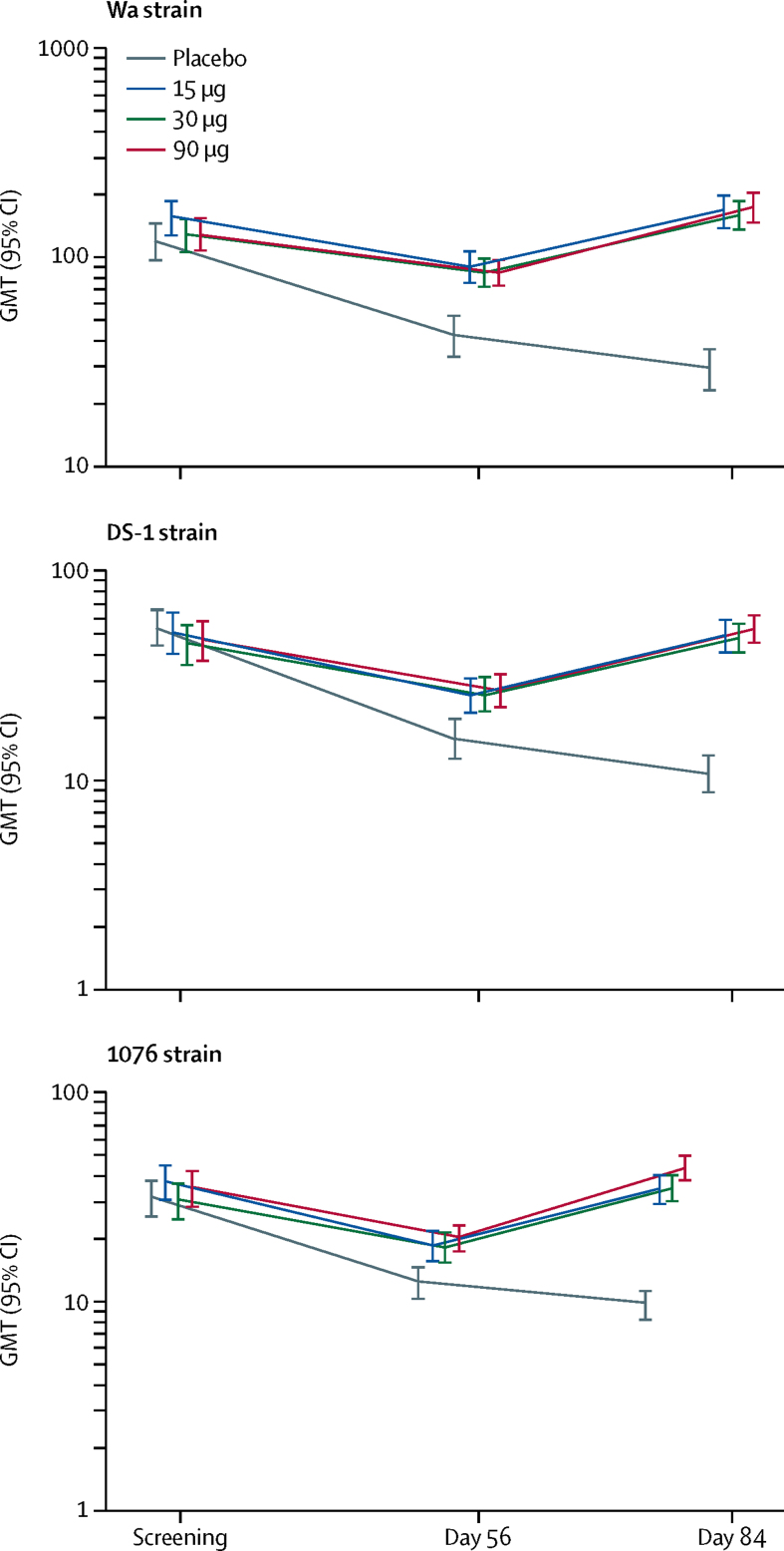
Neutralising antibodies to Wa, DS-1, and 1076 rotavirus strains 4 weeks after the second and third injection of trivalent P2-VP8 or placebo in the per-protocol infant population, according to treatment group GMT and 95% CI unadjusted for decrease in maternal antibodies. GMT=geometric mean titre.

After the third injection, adjusted neutralising antibody seroresponses (2·7-fold increase) to DS-1 (P[4]), 1076 (P[6]), and Wa (P[8]) were significantly higher in each dose group than in the placebo group (p<0·0001 for all comparisons; table 4, figure 2). The adjusted seroresponse rate for strain 1076 in the 90 μg group (81%) was significantly higher than in the 15 μg group (68%, p=0·013). Adjusted seroresponses to any two strains were seen in 101 (77%) of 132 infants in the 15 μg group, 108 (82%) of 132 in the 30 μg group, 114 (85%) of 134 in the 90 μg group, and 15 (12%) of 130 in the placebo group (p<0·0001 for all comparisons). Adjusted seroresponses to all three strains were observed in 65 (50%) of 131 infants in the 15 μg group, 81 (61%) of 132 in the 30 μg group, 83 (62%) of 134 in the 90 μg group, and ten (8%) of 130 in the placebo group (p<0·0001 for all comparisons). Results for adjusted seroresponses based on a 4-fold increase from baseline were consistent with those based on a 2·7-fold increase (appendix p 11).

**Table 4:** Serum neutralising antibody responses (2·7-fold increase) pre-vaccination and 4 weeks after the third injection of trivalent P2-VP8 or placebo in infants, according to treatment group

	**15 μg**	**30 μg**	**90 μg**	**Placebo**	p value*
**Wa strain**					
Number of observations	132	132	134	130	..
Pre-vaccination GMT	160 (136–189)	128 (108–152)	130 (110–154)	118 (98–141)	..
Post-vaccination GMT	166 (141–195)	158 (137–182)	172 (149–199)	29 (24–36)	<0·0001
p value†	<0·0001	<0·0001	<0·0001	..	..
Unadjusted seroresponse	30 (23%; 16–31)	32 (24%; 17–32)	43 (32%; 24–41)	5 (4%; 1–9)	<0·0001
Adjusted‡ seroresponse	96 (73%; 64–80)	107 (81%; 73–87)	105 (78%; 70–85)	26 (20%; 14–28)	<0·0001
p value§	<0·0001	<0·0001	<0·0001	..	..
**DS-1 strain**					
Number of observations	131	132	134	130	..
Pre-vaccination GMT	54 (44–66)	44 (36–54)	46 (38–57)	53 (44–63)	..
Post-vaccination GMT	49 (42–58)	48 (42–55)	53 (46–61)	11 (9–13)	<0·0001
p value†	<0·0001	<0·0001	<0·0001	..	..
Unadjusted seroresponse	26 (20%; 13–28)	29 (22%; 15–30)	33 (25%; 18–33)	4 (3%; 1–8)	<0·0001
Adjusted‡ seroresponse	100 (76%; 68–83)	105 (80%; 72–86)	108 (81%; 73–87)	26 (20%; 14–28)	<0·0001
p-value§	<0·0001	<0·0001	<0·0001	..	..
**1076 strain**					
Number of observations	132	132	134	130	..
Pre-vaccination GMT	39 (32–46)	30 (25–36)	34 (28–42)	31 (25–38)	..
Post-vaccination GMT	34 (29–40)	35 (30–40)	43 (38–49)	10 (8–11)	<0·0001
p value†	<0·0001	<0·0001	<0·0001	..	..
Unadjusted seroresponse	24 (18%; 12–26)	32 (24%; 17–32)	30 (22%; 16–30)	4 (3%; 1–8)	<0·0001
Adjusted‡ seroresponse	90 (68%; 60–76)	103 (78%; 70–85)	109 (81%; 74–88)	16 (12%; 7–19)	<0·0001
p value§	<0·0001	<0·0001	<0·0001	..	..

Data are n, GMT (95% CI), or n (%; 95% CI), unless otherwise indicated. GMT=geometric mean titre.

* Overall p value for the difference between groups in GMTs and proportions of infants with seroresponse.

† p value indicates pairwise comparison of each vaccine dose group with the placebo group in GMT increase from pre-vaccination to post-vaccination; titres were adjusted for maternal antibodies. Significant pairwise comparisons between vaccine groups are as follows: 1076 strain p=0·050 (30 μg *vs* 15 μg) and p=0·011 (90 μg *vs* 15 μg).

‡ Neutralising antibody post-injection titres were adjusted for decay in maternal antibodies (half-life of 35·9 days for Wa, 30·1 days for DS-1, and 34·8 days for 1076). Adjusted seroresponse was defined as ≥2·7-fold increase in titre between baseline and 4 weeks after the third injection (adjusted titre) in infants with an unadjusted post-injection titre greater than the limit of detection.^10^

§ p value indicates pairwise comparison of each vaccine dose group to placebo; no significant pairwise between-group differences were observed among the active dose groups, except for strain 1076 for which a seroresponse of 68% in the 15 μg group was significantly lower than an 81% seroresponse in the 90 μg group (p=0·013).

IgA and adjusted IgG seroresponses in infants 4 weeks after the second dose were significantly higher in each dose group than in the placebo group for all three antigens except for anti-P2-VP8 IgA P[6] responses in the 15 μg group (appendix p 12). Adjusted neutralising antibody seroresponses to all three strains (Wa, DS-1, and 1076) after the second dose were significantly higher in each dose group than in the placebo group (appendix p 12). Seroresponses observed in infants after the second dose were lower than those observed after the third dose.

The proportions of adults with anti-P2-VP8 IgG seroresponses for each antigen after the second and third doses were 0% in the placebo group, 91–100% in the 30 μg group, and 100% in the 90 μg group, with similar responses for the three antigens (appendix pp 13, 17). After the second and third injections, no adult in the placebo group had an anti-P2-VP8 IgA seroresponse compared with 82–100% of adults in the vaccine groups (appendix pp 13, 17). Similarly, no toddler in the placebo group had anti-P2-VP8 IgG or anti-P2-VP8 IgA seroresponses, whereas 58–83% of toddlers in the 30 μg group and 83–92% in the 90 μg group had an anti-P2-VP8 IgG seroresponse and 50–83% in the 30 μg group and 67–92% in the 90 μg group had an anti-P2-VP8 IgA seroresponse (appendix pp 13, 18). Neutralising antibody seroresponses to strain Wa were significantly higher in the 90 μg group (eight [73%] of 11) than in the placebo group (zero [0%] of four) after the third injection in adults, and in the 90 μg group (five [42%] of 12) than in placebo group (zero [0%] of six) after the injection in toddlers (appendix pp 13, 19).

Rotarix shedding data were available for 217 infants: 53 in the placebo group, 52 in the 15 μg group, 56 in the 30 μg group, and 56 in the 90 μg group. Faecal shedding of rotavirus (ELISA-positive) among infants at any timepoint (5 days, 7 days, or 9 days after the first dose of Rotarix) was observed for 26 (49%) infants in the placebo group, 23 (44%) in the 15 μg group, 20 (36%) in the 30 μg group, and 16 (29%) in the 90 μg group. PCR-confirmed Rotarix shedding at any timepoint was observed for 24 (45%) infants in the placebo group, 22 (42%) in the 15 μg group, 19 (34%) in the 30 μg group, and 15 (27%) in the 90 μg group. The reduction in shedding compared with the placebo group was not significant in the 15 μg and 30 μg groups but was significant in the 90 μg group (ELISA-positive: 42%, 95% CI 4–65, p=0·023; PCR-confirmed: 41%, 0·1–65, p=0·035).

## Discussion

The trivalent P2-VP8 rotavirus vaccine was generally well tolerated at all dose levels tested in adults, toddlers, and infants, with most adverse events being mild. Despite a slightly higher number of grade 2 adverse reactions in adult and toddler vaccine recipients than in adult and toddler placebo recipients, the number of events was small. No significant differences were observed in the proportions of infants with grade 2 or worse local reactions, systemic reactions, or unsolicited adverse events up to 28 days post-injection between the vaccine and placebo groups. When local reactogenicity was reported, it was rarely greater than mild in severity and never severe; systemic reactogenicity was also transient and generally mild, with few moderate events and rare severe events. No safety signals were identified, as reported in studies with the precursor monovalent P2-VP8-P[8] vaccine.^19^, ^20^ Immune responses to the trivalent vaccine among infants were promising in terms of both frequency and breadth of antibody seroresponses, showing good responses to P[4], P[6], and P[8] rotavirus strains; by contrast, responses to the previously tested monovalent vaccine were limited mainly to homologous P[8] strains.^20^ Licensed rotavirus vaccines have been shown to be effective against heterologous strains, and oral vaccines appear to confer immunity in a non-strain-specific manner.^24^ The protection potentially afforded by a parenteral subunit vaccine might be due to a different mechanism than the protection afforded by an oral vaccine and, thus, might be more strain specific; this question needs to be addressed with future efficacy studies.

Anti-P2VP8 IgG titres to P[4], P[6], and P[8] were high and similar for all three vaccine antigens. 99–100% of infants across all vaccine groups had a seroresponse 4 weeks after the third vaccine dose. Adjusted neutralising antibody responses to each of the strains (Wa, DS-1, and 1076) were shown after the third injection in 78–81% of infants in the 30 μg and 90 μg dose groups, and were similar across all three strains. The majority of vaccine recipients who were infants showed responses to at least two strains (77–85%), and lower proportions showed responses to all three strains (50–62%). Neutralising antibody responses to DS-1 (P[4]) and 1076 (P[6]) strains and IgG responses to P[4] and P[6] antigens were similar to those for the Wa (P[8]) strain and P[8] antigen and greater than the poor to modest responses to these antigens and strains that were observed with the monovalent P[8] formulation.^20^ The addition of antigens from P[4] and P[6] strains to the monovalent vaccine thus achieved the goal of high and similar IgG and neutralising antibody responses across all three strain types. The genetic diversity of circulating rotavirus strains in Africa is high, and although P[8] strains were the most prevalent strains (41%) during 2007–11 in 16 African countries, P[6] genotypes accounted for 31% of strains and P[4] genotypes accounted for 14% of strains.^21^ Thus, it is important for a rotavirus vaccine to protect against multiple rotavirus P types.

By contrast with the P2-VP8 IgG and neutralising antibody responses, P2-VP8 IgA seroresponses to the three vaccine antigens in infants receiving the vaccine, although significantly higher than responses in the placebo group, ranged from 20% to 34%. P2-VP8 IgA responses to P[8] were lower than those observed for the monovalent vaccine (20–28% *vs* 58–81%).^20^ These results were unexpected given the promising P2-VP8 IgG and neutralising antibody responses, and the reason for these low IgA responses with the trivalent vaccine is not clear. However, it is possible for parenteral vaccines to induce lower titres of specific IgA than IgG and still be associated with protection.^25^ Increased titres of serum neutralising antibody and IgG might result in protection through their transudation or permeation into the intestinal lumen, a possibility suggested by a study in non-human primates, which were protected from rotavirus infection by parenteral treatment with serum with high anti-rotavirus IgG titres.^25^

Antibody seroresponses measured 4 weeks after the second dose in infants receiving the 30 μg and 90 μg doses were all significantly higher than in the placebo group. However, responses were lower than those observed after the third dose, which suggests that three doses of the parenteral vaccine are needed to provide an optimal immune response. Anti-P2-VP8 IgG and IgA seroresponses were not significantly different across the different doses. However, for some of the neutralising antibody responses, the 30 μg and 90 μg doses had significantly higher responses than the 15 μg dose, suggesting that the higher doses might elicit better immune responses.

Shedding of Rotarix was assessed as a measure of the effect of the vaccine on replication of rotavirus in the gut in a subset of infants. There was a significant reduction of shedding of Rotarix in the first week after the first dose of the oral vaccine among infants who received the 90 μg dose compared with infants who received placebo, as previously shown with the monovalent formulation.^20^ This finding suggests that the P2-VP vaccine confers protection at the gut level and that the higher dose confers superior protection over the lower doses against disease. However, the use and interpretation of a Rotarix challenge dose as a potential proxy measure of efficacy still needs investigation.

Immune responses in adults were high, particularly after the second and third doses, and they were reasonably good in toddlers who received only one dose of parenteral vaccine. The toddlers had been primed by receipt of two doses of Rotarix through routine vaccination as infants, and then received a single dose of trivalent P2-VP8 vaccine at around 31 months of age as part of the study. None of the toddlers in the placebo group showed a 4-fold or greater increase in anti-P2-VP8 IgG or IgA after vaccination compared with baseline, whereas IgG seroresponses were observed in 83–92% and IgA responses in 67–92% of toddlers in the 90 μg group. This result is of interest when considering possible combination oral–parenteral rotavirus vaccine schedules: such combinations might increase protection against rotavirus.^15^ A prime–boost infant immunisation strategy could be used, in which infants are primed with an oral live-attenuated vaccine—for example, at 6 and 10 weeks of age—then boosted with a parenteral rotavirus vaccine at 14 weeks of age. A study assessing the ability of inactivated poliovirus vaccine to boost mucosal immunity showed that a dose of this vaccine was more effective than an oral poliovirus vaccine at boosting intestinal immunity in children who had previously been immunised with the oral vaccine.^26^ Our results suggest that a prime–boost vaccination strategy could be an area for future rotavirus vaccine research.

There are limitations to our study. First, no correlate of protection has been established for rotavirus disease.^27^ Serum IgA antibody detected by whole-rotavirus lysate ELISA, which primarily detects anti-VP6 antibodies, was shown to be correlated with efficacy of Rotarix and RotaTeq (Merck & Co, USA).^28^ An analysis of data from the RotaTeq efficacy studies found that serum neutralising antibody titres to the G1 serotype are the best correlate of protection for RotaTeq.^29^ We have yet to establish whether the immune responses measured in our study—serum IgA to the vaccine antigens rather than whole viral lysate—are associated with protection from rotavirus diarrhoea. Second, coadministration of the study vaccine with other vaccines might have reduced our ability to adequately assess systemic reactogenicity of the study vaccine in infants. Finally, although we vaccinated infants outside of the rotavirus season, it is possible that some infants had been exposed to natural rotavirus infection, which might have affected our results. In addition, we included infants who were being breastfed; thus, maternal antibodies present in breastmilk might have affected the shedding results.^30^ However, these factors would probably introduce non-differential bias and drive results towards the null.

Vaccinating infants against rotavirus disease using parenterally administered rotavirus vaccines could have several advantages over the currently licensed live oral vaccines, including improved efficacy in low-income countries, lower production costs, improved safety because the vaccine is unlikely to be associated with increased risk of intussusception, and potential coformulation with other Expanded Program on Immunization vaccines or future combination parenteral vaccines targeting multiple enteric pathogens. The non-replicating, parenterally administered, trivalent P2-VP8 subunit vaccine showed promising safety and immunogenicity, which support advancing the vaccine to efficacy testing: phase 3 studies using the 90 μg dose have started (eg, NCT04010448).

## Data sharing

Individual participant data that underlie the results reported in this Article, after de-identification, will be made available to researchers who provide a methodologically sound proposal, for analyses to achieve aims in the approved proposal, immediately following Article publication. Proposals should be directed to groomem@rmpru.co.za.
