# Supplementary material for: Safety and immunogenicity of a parenteral trivalent P2-VP8 subunit rotavirus vaccine: a multisite, randomised, double-blind, placebo-controlled trial
Source: Lancet Infect Dis. 2020 Jul;20(7):851–63. doi: 10.1016/S1473-3099(20)30001-3 (PMC7322558; doi:10.1016/S1473-3099(20)30001-3)
Supplement: Supplementary appendix [file mmc1.pdf]

THE LANCET
Infectious Diseases

Supplementary appendix

This appendix formed part of the original submission and has been peer reviewed. We post it as supplied by the authors.

Supplement to: Groome MJ, Fairlie L, Morrison J, et al. Safety and immunogenicity of a parenteral trivalent P2-VP8 subunit rotavirus vaccine: a multisite, randomised, double-blind, placebo-controlled trial. *Lancet Infect Dis* 2020; published online April 3. https://doi.org/10.1016/S1473-3099(20)30001-3.

Appendix

Rationale for dose selection

Dose selection for this study was based upon previous findings with the monovalent P2-VP8-P[8] vaccine in South African infants (Groome et al. 2017). Briefly, the three doses evaluated (10µg, 30µg and 60µg) in that study elicited comparable IgG, IgA and neutralizing antibody responses. Of particular note was the finding that the 60µg dose was not superior to the 30µg dose of vaccine, based on antibody responses or the ability to reduce shedding of the Rotarix vaccine strain. Therefore, 30µg per antigen (total of 90µg, combined) was the highest dose selected. In such dose ranging studies, it is highly desirable to show a true dose response which led us to select 5µg per antigen as the lowest dose (infants only). The total dose for the current study of the trivalent vaccine contained 30µg of each of the three antigens for the 90µg dose, 10µg of each of the three antigens for the 30µg dose, and 5µg of each of the three antigens for the 15µg dose.

The reason for the dose-escalation phase was to first demonstrate that the vaccine exhibited acceptable safety and tolerability in modestly-sized cohorts before the vaccine was tested in a larger group of the target population (infants), especially since the P4 and P6 antigens have not been previously evaluated in humans.

Dose-escalation phase: The two highest dose-levels (30µg and 90µg) were first tested in adults and toddlers to assess tolerability of the vaccine before moving into infants. Three doses were tested in the adult groups, consistent with the Phase I study (Fix et al. 2015) where three doses were assessed. A single dose was tested in toddlers, consistent with the single dose tested in the Phase I/II study of the monovalent vaccine (Groome et al. 2017). Toddlers were included in order to demonstrate tolerability and safety before moving down to the target infant population. Data following a single vaccination was considered sufficient to be able to progress to infants, which was also the approach in the prior study with the monovalent vaccine. As the adult and toddler groups were included primarily to assess safety prior to progressing to the target infant population, it was deemed unnecessary to assess the lowest dose to be assessed in infants. Three doses of the 15µg, 30µg and 90µg dose levels were then tested in a small group of infants to establish tolerability of the vaccine. Based on safety data obtained during the dose-escalation phase, three doses of each of the dose levels were then assessed in a larger group of infants: the expanded infant phase of the study. The reason for the extra placebo in the infant groups (n=16) vs the adult and toddler groups (n=15) during the dose escalation phase was to get the right ratio (1:1:1:1) for placebo and each of the three dose-levels, once infants from the dose-escalation cohorts and expanded cohorts were combined.

Toddlers would have received two doses of Rotarix at 6 and 14 weeks of age, as per the South African EPI schedule. Three doses of Rotarix were administered to infants, rather than two doses as per the EPI schedule, to counterbalance the remote theoretical possibility that the P2-VP8 subunit vaccine could reduce response to Rotarix.

Study inclusion and exclusion criteria

<u>Inclusion criteria</u>

Fulfillment of all of the following criteria is required to accept an adult, toddler or infant in the study:

1. Healthy adults, toddlers and infants as established by medical history and clinical examination before entering the study
2. Age:
    a. Adults: ≥18 and ≤45 years old at time of enrollment
    b. Toddlers: ≥2 and <3 years old at the time of enrollment
    c. Infants: ≥6 and <8 weeks at the time of enrollment
3. Participant (adults) or parental (toddlers and infants) ability and willingness to provide written informed consent
4. Intention for the participant (and for toddlers and infants, the parent) to remain in the area with the child during the study period
5. If female and of childbearing potential, be not breastfeeding and not pregnant (based on a negative serum pregnancy test at screening and a negative urine pregnancy test at each study injection visit, prior to injection), planning to avoid pregnancy for at least 4 weeks after the last injection, and willing to use an adequate method of contraception consistently and have repeated pregnancy tests prior to second and third injections.

Exclusion Criteria

Any of the following will exclude an adult, toddler or infant from the study:

1. Presence of fever on the day of enrollment (axillary or oral temperature >37.6°C)
2. Acute disease at the time of enrollment
3. Concurrent participation in another clinical trial throughout the entire timeframe for this study
4. Presence of malnutrition or any systemic disorder (cardiovascular, pulmonary, hepatic, renal, gastrointestinal, hematological, endocrine, immunological, dermatological, neurological, cancer or autoimmune disease) as determined by medical history and/or physical examination that would compromise the participant's health or is likely to result in nonconformance to the protocol
5. For infant cohort, history of premature birth (<37 weeks' gestation)
6. History of congenital abdominal disorders, intussusception, or abdominal surgery
7. Known or suspected impairment of immunological function based on medical history and physical examination
8. For infant cohort only, prior receipt of rotavirus vaccine
9. A known sensitivity or allergy to any components of the study vaccine
10. History of anaphylactic reaction
11. Major congenital or genetic defect
12. Parents of participating toddlers or infants not able, available or willing to accept active weekly follow-up by the study staff
13. Receipt of any immunoglobulin therapy and/or blood products in the last 6 months or planned administration during the study period
14. History of chronic administration (defined as more than 14 days) of immunosuppressant medications, including corticosteroids, in the last 6 months (those on inhaled or topical steroids may be permitted to participate in the study)
15. Any medical condition in the participant (or parents of toddler and infant participants) that, in the judgment of the investigator, would interfere with or serves as a contraindication to protocol adherence or a participant's (or parents') ability to give informed consent
16. Clinically significant screening laboratory value*
17. HIV infection
    a. For adults and toddlers, to be assessed by HIV ELISA
    b. For infants, to be assessed by PCR, if mother is not known to be negative (negative test result between 24 weeks' gestation and screening)

*Grade 1 laboratory abnormalities will not be considered to be exclusionary at screening unless judged to be clinically significant by the PI.

Safety assessments

The Safety Review Committee (SRC) evaluated clinical and laboratory safety data through Day 7 after the first injection for all participants. The SRC, comprised of the site PIs from each study site, the Sponsor medical monitor, the Emmes medical monitor and two independent local medical experts, monitored safety throughout the duration of the study (in addition to the Data Safety Monitoring Board). In addition to routine review of safety information, a central role of the SRC was the review of safety data for the recommendation of whether to progress to the next dose level in each age cohort and to the next age cohort. Cumulative safety data were available continuously for review by SRC members, and safety reports were prepared for each SRC deliberation of dose escalation and progression to subsequent cohorts (adults to toddlers and toddlers to infants).
The SRC members reviewed the safety data throughout the study to determine whether pause criteria had been met.

Study pause rules

Meeting one or more of the following criteria would automatically pause or halt further study injections in the study. These pause rules referred to suspected adverse reactions across all cohorts, as defined below:

- One participant with a vaccine-related serious AE (SAE).
- Two participants with ≥grade 3 (severe) localized inflammatory reaction at the injection site.
- The same objective ≥grade 3 (severe) systemic reactogenicity signs or symptoms (fever, vomiting), within seven days following study injection in three participants in Groups A (adults), B (toddlers) or C (infant dose escalation), and ≥5% of participants in Group D (expanded infant group).
- Two participants with a systemic rash, including, but not limited to, generalized urticaria, generalized petechiae, or erythema multiforme, within seven days following study injection.
- Two participants with the same ≥grade 3 (severe) vaccine-related abnormal clinical laboratory evaluation, within seven days following study injection.

Toxicity Grading Scales

The study clinic assigned toxicity grades to indicate the severity of adverse experiences and toxicities. The toxicity grading criteria graded AEs from mild (grade 1) to life-threatening (grade 4). All AEs leading to death were Grade 5 events. AEs were graded with the worst severity grade during the illness/symptoms. AE severity were graded using the grading scale below, which was developed on the basis of Division of AIDS (DAIDS) Table for Grading the Severity of Adult and Pediatric Adverse Events, version 2.0, November 2014, of the US National Institutes of Health, with modifications to reflect local population norms. For laboratory values not included below, grading was based on the Division of AIDS (DAIDS) Table for Grading the Severity of Adult and Pediatric Adverse Events, version 2.0, November 2014, available at webpage: http://rsc.tech-res.com/safetyandpharmacovigilance/gradingtables.aspx

Systemic Illness	Mild (Grade 1)	Moderate (Grade 2)	Severe (Grade 3)	Potentially Life Threatening (Grade 4)
Illness or clinical AE (as defined according to applicable regulations)	No or minimal interference with usual activities; no medical intervention/ therapy required	Greater than minimal interference with usual activities; no or minimal medical intervention/ therapy required	Marked limitation in ability to perform usual activities; medical intervention/ therapy required	Inability to perform basic functions OR Medical or operative intervention indicated to prevent permanent impairment, persistent disability, or death

Local Reaction to Injectable Product	Mild (Grade 1)	Moderate (Grade 2)	Severe (Grade 3)	Potentially Life Threatening (Grade 4)
Injection site pain (pain without touching) OR Tenderness (pain when area touched)	Pain/tenderness causing no or minimal limitation of use of limb	Pain or tenderness causing greater than minimal limitation of use of limb	Pain/tenderness causing inability to perform usual activities	Pain/tenderness causing inability to perform basic functions OR Hospitalization indicated
Injection site erythema or induration<br><br>Infants and toddlers	≤ 2.5 cm in diameter	> 2.5 cm in diameter with < 50% surface area of the extremity segment involved (thigh)	≥ 50% surface area of the extremity segment involved (thigh) OR Ulceration OR Secondary infection OR Phlebitis OR Sterile abscess OR Drainage	Potentially life-threatening consequences (e.g., abscess, exfoliative dermatitis, necrosis involving dermis or deeper tissue)
Injection site erythema or induration<br><br>Adults	2.5 to < 5 cm in diameter OR 6.25 to < 25 cm^2 surface area AND Symptoms causing no or minimal interference with usual social & functional activities	≥ 5 to < 10 cm in diameter OR ≥ 25 to < 100 cm^2 surface area OR Symptoms causing greater than minimal interference with usual social & functional activities	≥ 10 cm in diameter OR ≥ 100 cm^2 surface area OR Ulceration OR Secondary infection OR Phlebitis OR Sterile abscess OR Drainage OR Symptoms causing inability to perform usual social & functional activities	Potentially life-threatening consequences (e.g., abscess, exfoliative dermatitis, necrosis involving dermis or deeper tissue)
Injection site pruritus	Itching localized to the injection site that is relieved spontaneously or in < 48 hours of treatment	Itching beyond the injection site that is not generalized OR Itching localized to the injection site requiring ≥ 48 hours treatment	Generalized itching causing inability to perform usual social & functional activities	NA

Systemic (General)	Mild (Grade 1)	Moderate (Grade 2)	Severe (Grade 3)	Potentially Life Threatening (Grade 4)
Acute systemic allergic reaction	Localized urticaria (wheals) with no medical intervention indicated	Localized urticaria with medical intervention indicated OR Mild angioedema with no medical intervention indicated	Generalized urticaria OR Angioedema with medical intervention indicated OR Symptomatic mild bronchospasm	Acute anaphylaxis OR Life-threatening bronchospasm OR laryngeal edema
Fever	37.7 – 38.6°C	38.7 – 39.3°C	39.4 – 40.5°C	> 40.5°C
Vomiting	Transient or intermittent vomiting with no or minimal interference with oral intake	Frequent episodes of vomiting with no or mild dehydration	Persistent vomiting resulting in greater than milde dehydration OR Aggressive rehydration indicated (e.g., IV fluids)	Life-threatening consequences (e.g., hypotensive shock)
Irritability	Crying more than usual but easily consoled	Crying more than usual and somewhat difficult to console	Continuous crying that is inconsolable for 4 hours or more	

Systemic (General)	Mild (Grade 1)	Moderate (Grade 2)	Severe (Grade 3)	Potentially Life Threatening (Grade 4)
Decreased activity	Slightly subdued, but responds normally to stimuli	Subdued and does not respond as readily as normal to stimuli	Lethargic	Obtunded
Decreased appetite	Loss of appetite without decreased oral intake	Loss of appetite associated with decreased oral intake without significant weight loss	Loss of appetite associated with significant weight loss	Life-threatening consequences OR Aggressive intervention indicated [e.g., tube feeding or total parenteral nutrition (TPN)]
Nausea	Transient (< 24 hours) or intermittent AND No or minimal interference with oral intake	Persistent nausea resulting in decreased oral intake for 24 to 48 hours	Persistent nausea resulting in minimal oral intake for > 48 hours OR Rehydration indicated (e.g., IV fluids)	Life-threatening consequences (e.g., hypotensive shock)
Myalgia (generalized)	Muscle pain causing no or minimal interference with usual social & functional activities	Muscle pain causing greater than minimal interference with usual social & functional activities	Muscle pain causing inability to perform usual social & functional activities	Disabling muscle pain causing inability to perform basic self-care functions
Headache	Symptoms causing no or minimal interference with usual social & functional activities	Symptoms causing greater than minimal interference with usual social & functional activities	Symptoms causing inability to perform usual social & functional activities	Symptoms causing inability to perform basic self-care functions OR Hospitalization indicated OR Headache with significant impairment of alertness or other neurologic function
Chills	Symptoms causing no or minimal interference with usual social & functional activities	Symptoms causing greater than minimal interference with usual social & functional activities	Symptoms causing inability to perform usual social & functional activities	NA
Fatigue	Symptoms causing no or minimal interference with usual social & functional activities	Symptoms causing greater than minimal interference with usual social & functional activities	Symptoms causing inability to perform usual social & functional activities	Incapacitating symptoms of fatigue or malaise causing inability to perform basic self-care functions

Chemistry	Mild (Grade 1)	Moderate (Grade 2)	Severe (Grade 3)	Potentially Life Threatening (Grade 4)**
Creatinine	1.1-1.3 ULN	>1.3-1.8 ULN	>1.8 to <3.5 ULN	≥3.5 ULN
Albumin – Hypoalbuminemia G/L	NA	20 to <30	<20	--
ALT	1.25 to <2.5 ULN	2.5 to <5.0 ULN	5.0 to <10.0 ULN	≥10 ULN
Bilirubin	1.1 to <1.6 ULN	1.6 to <2.6 ULN	2.6 to <5.0 ULN	≥5.0 ULN
**Hematology**	**Mild (Grade 1)**	**Moderate (Grade 2)**	**Severe (Grade 3)**	**Potentially Life Threatening (Grade 4)**
Hemoglobin - g/dL				
36 to 56 days of age (male and female)	8.5 to 9.6	7.0 to <8.5	6.0 to <7.0	<6.0
57 days of age to < 13 years of age (male and female)	9.5 to 10.4	8.5 to <9.5	6.5 to <8.5	<6.5
≥ 13 years of age (male only)	10.0 to 10.9	9.0 to <10.0	7.0 to <9.0	<7.0
≥ 13 years of age (female only)	9.5 to 10.4	8.5 to <9.5	6.5 to <8.5	<6.5
WBC Decrease - cell/mm^3	2,000 to 2,499	1,500 to 1,999	1,000 to 1,499	< 1,000
Platelets - cell/mm^3	100 to <124 $X10^3$	50 to <100 $X10^3$	25 to <50 $X10^3$	<25 $X10^3$

Supplementary laboratory methods

ELISA assay to determine antibody to P8, P6 and P4 vaccine antigens

Anti-P2-VP8 Immunoglobulin G (IgG) or A (IgA) antibody against each VP8 protein (P8, P6, and P4) was quantitated using an ELISA assay. For each vaccine antigen, ELISA plates were coated with 100 µL of 1.0 µg/mL of P2-VP8 (either P8, P6 or P4) antigen in calcium carbonate buffer (pH 9.6) and incubated overnight at 4° C. The plates were washed with phosphate buffered saline + 0.05% Tween 20 (Fisher Scientific, Pittsburgh, PA) (PBST) and blocked with 100 µl of diluent (PBST with1% non-fat dry milk) for 1 hour on a rotating platform at 37°C. Reference standards, controls and samples were diluted in diluent, plates were washed and 50 µl of each were added to the plates. The plates were incubated at 37°C for 1 hour. After washing, biotinylated goat anti human IgG or IgA (Sigma Aldrich, St. Louis, MO) was added (50 µl/well) and incubated at 37°C for 30 minutes. Plates were washed and then 50 ul per well of peroxidase conjugated avidin:biotin (Vector Laboratories, Inc., Burlingame, CA) diluted in wash buffer was added. The plates were incubated for 30 minutes at 37°C. After a final wash, the substrate O-phenylenediamine (OPD)(Sigma) was added. Development was stopped after 15 minutes with 75 µl per well of 1.0M $H2SO4$. Plates were read at 492nm on a Molecular Devices SpectraMax 190 plate reader. A standard curve was modeled using a four parameter logistic regression function in the SoftMax software for the reader. The concentration of anti- P2-VP8 specific IgG or IgA in a sample was derived by extrapolation from a reference standard curve established for each antigen. The reference standards were assigned a value of 1000, 800 or 500 units per mL IgG and 500, 200 or 500 units per mL for IgA for P8, P6 and P4 antigens, respectively. Each reference standard was diluted so that the starting dilution was 10 units/mL for both IgG and IgA. The lower limit of quantification was determined during qualification of the assay to be 15.15 units per mL for IgG and 8.72 units/mL for IgA.

Serum rotavirus neutralization assay

The viruses used in this study were Wa G1P8, 1076 G4P6 and DS-1 G2P4. Viruses were originally obtained from the National Institute of Health and a stock was grown for use in the assay. The rotavirus serum neutralizing antibody assay was performed by titrating a serum sample and incubating it with a constant amount of a specific strain of rotavirus, allowing neutralization of the virus. The rotavirus/serum mix is then incubated on a rotavirus susceptible cell line, MA104 (monkey kidney) cells, cultured in 96-well plates. Control wells on each 96-well plate received either diluted virus without sera (virus control, VC) or no virus or sera (cell controls, CC). After an overnight incubation allowing for rotavirus growth, the cells were frozen, lysed, and resuspended. The relative amount of unneutralized rotavirus antigen is determined using an antigen capture format ELISA to detect rotavirus. The ELISA plates were coated with rabbit anti-rotavirus IgG antibody. The culture supernatants from the neutralization part of the assay were added and incubated. Following a blocking step with nonfat milk, guinea pig anti-rotavirus antiserum is added to the wells to detect captured rotavirus. Horseradish peroxidase (HRP) conjugated rabbit anti-guinea pig IgG (Jackson ImmunoResearch, West Grove, PA) is added to detect the guinea pig anti-rotavirus antibody. The wells are developed with o-Phenylenediamine (OPD (SigmaAldrich)), a peroxidase sensitive colorimetric substrate. Optical density (OD) of each well is read as absorbance at 492 nm. The amount of rotavirus present in the resuspended lysate from each well is inversely related to the amount of neutralizing antibody present in the serum. Each serum dilution series is modeled using a logistic regression function. For each fitted curve the dilution which corresponds to a 40% response (ED40), compared to the virus controls, is determined and reported as the titer. The ED40 represents the titer of the serum against a given virus, which represents a 60% reduction in amount of virus.[1]

1. Knowlton DR, Spector DM, Ward RL. Development of an improved method for measuring neutralizing antibody to rotavirus. Journal of virological methods 1991; 33(1-2): 127-34

Secondary Immunogenicity Endpoints

Infants:

- Proportion of infants with anti-P2-VP8 IgG & IgA seroresponses by ELISA (four-fold increase in antibody titers between baseline and 4 weeks post-second study injection) in assays using each of the three vaccine antigens (P[4], P[6] and P[8])
- Proportion of infants with neutralizing antibody responses (2.7-fold increase in antibody titers between baseline and 4 weeks post-second study injection) to each of the three rotavirus strains from which the vaccine antigens are derived, as well as heterologous strains
- Anti-P2-VP8 IgG & IgA geometric mean titers (GMT) (baseline and 4 weeks post-second study injection) in ELISA assays using each of the three vaccine antigens (P[4], P[6] and P[8])
- Neutralizing antibody GMT (baseline and 4 weeks post-second study injection) to each of the three rotavirus strains from which the vaccine antigens are derived, as well as heterologous strains
- Proportion of infants with neutralizing antibody responses to at least two of the three strains from which the vaccine antigens are derived (4 weeks post-second study injection)

Adults and toddlers:

- Proportion of adults and toddlers with anti-P2-VP8 IgG & IgA seroresponses by ELISA (four-fold increase in antibody titers between baseline and 4-weeks post-final study injection) in assays using each of the three vaccine antigens (P[4], P[6] and P[8])
- Proportion of adults and toddlers with neutralizing antibody responses (2.7-fold increases in antibody titers between baseline and 4-weeks post-final study injection) to each of the three rotavirus strains from which the vaccine antigens are derived, as well as heterologous strains
- Anti-P2-VP8 IgG & IgA geometric mean titers (GMT) (baseline and 4 weeks post final study injection) in ELISA assays using each of the three vaccine antigens (P[4], P[6] and P[8])
- Neutralizing antibody GMT (baseline and 4 weeks post-final study injection) to each of the three rotavirus strains from which the vaccine antigens are derived, as well as heterologous strains
- Proportion of adults and toddlers with neutralizing antibody responses to at least two of the three strains from which the vaccine antigens are derived

Additional statistical methods

Sample size: The sample size of 150 participants per group gave 90% power to detect an event with a 1.6% rate of occurrence. When enrollment was stopped, we had a minimum of 139 participants per group which gave the same power to detect a 1.7% rate, which was close enough to the original calculation. For immunogenicity, we had calculated that 135 (assuming 10% loss) infants per group would have been enough to test for differences in seroresponse rates. In the end we enrolled 139 infants or more in each group.

Protocol deviations: Major protocol deviations include any deviation to the protocol that may have had an effect on the safety or rights of the participant, or the integrity of the study. For the purpose of immunogenicity assessment in the per-protocol population, a "major" violation was defined as a protocol deviation that was considered to have an impact on the immunogenicity results of the study. All major violations were identified prior to database lock and unblinding.

Selection of 2.7-fold rise for assessment of neutralising antibody responses: The 2.7-fold rise was found to be significant based on the estimated variability of this validated assay assessing neutralizing antibodies to each of the three rotavirus strains from which the vaccine antigens were derived. This was the criteria used in the studies for the oral rotavirus vaccines (Rotateq) and based on the validation of the neutralization assay. The post-hoc analysis was performed using a 4-fold rise as this has used as a response cut-off in similar studies.

Supplementary Table 1: Study schema

Group		TV P2-VP8 Dose	N	-7[a] to -1	0	7	28	56	84	168	224
A Adult	A1	30 µg	12	B(S,I)	X	B(S)	X, B(I)	X, B(I)	B(I)		F
		Placebo	3	B(S,I)	P	B(S)	P, B(I)	P, B(I)	B(I)		F
	A2	90 µg	12	B(S,I)	X	B(S)	X, B(I)	X, B(I)	B(I)		F
		Placebo	3	B(S,I)	P	B(S)	P, B(I)	P, B(I)	B(I)		F
A Total			30								
B Toddler	B1	30 µg	12	B(S,I)	X	B(S)	B(I)			F	
		Placebo	3	B(S,I)	P	B(S)	B(I)			F	
	B2	90 µg	12	B(S,I)	X	B(S)	B(I)			F	
		Placebo	3	B(S,I)	P	B(S)	B(I)			F	
B Total			30								
C Infant	C1	15 µg	12	B(S,I)	X	B(S)	X	X, B(I)	B(I)		F
		Placebo	4	B(S,I)	P	B(S)	P	P, B(I)	B(I)		F
	C2	30 µg	12	B(S,I)	X	B(S)	X	X, B(I)	B(I)		F
		Placebo	4	B(S,I)	P	B(S)	P	P, B(I)	B(I)		F
	C3	90 µg	12	B(S,I)	X	B(S)	X	X, B(I)	B(I)		F
		Placebo	4	B(S,I)	P	B(S)	P	P, B(I)	B(I)		F
C Total			48								
D Infant		15 µg	138	B(S,I)	X		X	X, B(I)	B(I)		F
		30 µg	138	B(S,I)	X		X	X, B(I)	B(I)		F
		90 µg	138	B(S,I)	X		X	X, B(I)	B(I)		F
		Placebo	138	B(S,I)	P		P	P, B(I)	B(I)		F
D Total			552								
All Cohorts Total			660								

X = TV P2-VP8 vaccine
P = placebo
B = blood sample for (S) safety and/or (I) immunogenicity testing
F = final study f/u visit/call
[a]For adult and toddler cohorts, -28 to -1

Supplementary Table 2: Demographic characteristics – adults and toddlers

	30µg	90µg	Placebo
Adults	n=12	n=12	n=6
Age (years)	32·0 (4·8)	26·0 (6·0)	25·3 (2·1)
Sex (male)	6 (50)	3 (25)	3 (50)
Race (black)	12 (100)	12 (100)	6 (100)
Height (cm)	164·4 (10·6)	160·6 (8·3)	167·0 (12·5)
Body mass index	25·7 (5·2)	24·0 (4·5)	22·7 (3·9)
Toddlers	n=12	n=12	n=6
Age (months)	30·9 (1·9)	30·3 (2·0)	31·0 (0·9)
Sex (male)	5 (42)	7 (58)	2 (33)
Race (black)	12 (100)	12 (100)	6 (100)
Height (cm)	86·4 (7·6)	88·5 (4·3)	88·8 (6·8)
Weight (kg)	12·6 (1·5)	13·5 (2·3)	13·8 (2·4)

Data are n (%) for sex and race, and mean (standard deviation) for age, height, body mass index and weight.

Supplementary Table 3: Proportion of participants with solicited and unsolicited adverse events following any injection in adults and a single injection in toddlers, according to treatment group

	30µg	90µg	Placebo
Adults	n=12	n=12	n=6
Solicited reactions (first seven days)			
Local – Grade 1 (mild)	6 (50)	6 (50)	1 (17)
Local – Grade 2 (moderate)	1 (8)	3 (25)	0 (0)
Systemic - Grade 1 (mild)	6 (50)	6 (50)	5 (83)
Systemic - Grade 2 (moderate)	0 (0)	3 (25)	0 (0)
Unsolicited adverse events - first 28 days			
Any	8 (67)	9 (75)	2 (33)
Grade 2 (moderate)	2 (17)	1 (8)	0 (0)
Related, any severity	0 (0)	1 (8) #	0 (0)
Related, Grade 2 (moderate)	0 (0)	0 (0)	0 (0)
Serious adverse event – any severity	0 (0)	0 (0)	0 (0)
Unsolicited adverse events - through 6 months			
Any	8 (67)	11 (92)	2 (33)
Grade 2 (moderate)	2 (17)	1 (8)	0 (0)
Related, any severity	0 (0)	1 (8) #	0 (0)
Related, Grade 2 (moderate)	0 (0)	0 (0)	0 (0)
Serious adverse event – any severity	0 (0)	0 (0)	0 (0)
Toddlers	n=12	n=12	n=6
Solicited reactions (first seven days)			
Local - Grade 1 (mild)	4 (33)	2 (17)	2 (33)
Local - Grade 2 (moderate)	2 (17)	0 (0)	0 (0)
Systemic - Grade 1 (mild)	5 (42)	2 (17)	3 (50)
Systemic - Grade 2 (moderate)	0 (0)	0 (0)	1 (17)
Unsolicited adverse events - first 28 days			
Any	8 (67)	6 (50)	3 (50)
Grade 2 (moderate)	1 (8)	1 (8)	0 (0)
Related, any severity	0 (0)	0 (0)	0 (0)
Related, Grade 2 (moderate)	0 (0)	0 (0)	0 (0)
Serious adverse event – any severity	1 (8)	0 (0)	0 (0)
Unsolicited adverse events - through 6 months			
Any	9 (75)	8 (67)	5 (83)
Grade 2 (moderate)	2 (17)	2 (17)	0 (0)
Related, any severity	0 (0)	0 (0)	0 (0)
Related, Grade 2 (moderate)	0 (0)	0 (0)	0 (0)
Serious adverse event – any severity	1 (8)	1 (8)	0 (0)

Data are n (%). No adults or toddlers experienced greater than moderate local reactions, systemic reactions, or unsolicited adverse events.

#One adult in 90µg group experienced a mild unsolicited AE (diarrhoea) which was assessed by the site investigator as having a reasonable possibility that the study product caused the event.

Supplementary Table 4: Proportion of participants with any unsolicited adverse events occurring within 28 days of vaccination, by MedDRA® system organ class and treatment group

MedDRA® system organ class	Adults			Toddlers			Infants			
	30µg	90µg	Placebo	30µg	90µg	Placebo	15µg	30µg	90µg	Placebo
	N=12	N=12	N=6	N=12	N=12	N=6	N=139	N=140	N=139	N=139
Any event	8 (67)	9 (75)	2 (33)	8 (67)	6 (50)	3 (50)	118 (85)	122 (87)	124 (89)	119 (86)
Blood and lymphatic system disorders	0 (0)	0 (0)	0 (0)	0 (0)	0 (0)	0 (0)	0 (0)	2 (1)	0 (0)	0 (0)
Congenital, familial and genetic disorders	0 (0)	0 (0)	0 (0)	0 (0)	0 (0)	0 (0)	0 (0)	0 (0)	1 (0.7)	0 (0)
Eye disorders	0 (0)	0 (0)	0 (0)	0 (0)	0 (0)	0 (0)	1 (0.7)	1 (0.7)	1 (0.7)	1 (0.7)
Gastrointestinal disorders	1 (8)	1 (8)	1 (17)	2 (17)	0 (0)	1 (17)	19 (14)	18 (13)	21 (15)	26 (19)
General disorders and administration site conditions	1 (8)	2 (17)	0 (0)	0 (0)	1 (8)	0 (0)	3 (2)	5 (4)	2 (1)	4 (3)
Immune system disorders	0 (0)	1 (12)	0 (0)	0 (0)	0 (0)	0 (0)	0 (0)	0 (0)	1 (0.7)	0 (0)
Infections and infestations	5 (42)	6 (50)	1 (17)	7 (58)	5 (42)	2 (33)	96 (69)	96 (69)	100 (72)	97 (70)
Injury, poisoning and procedural complications	0 (0)	0 (0)	0 (0)	0 (0)	0 (0)	0 (0)	0 (0)	2 (1)	1 (0.7)	0 (0)
Investigations	0 (0)	0 (0)	0 (0)	0 (0)	0 (0)	0 (0)	0 (0)	0 (0)	1 (0.7)	1 (0.7)
Metabolism and nutrition disorders	0 (0)	0 (0)	0 (0)	0 (0)	0 (0)	0 (0)	0 (0)	3 (2)	1 (0.7)	2 (1)
Musculoskeletal and connective tissue disorders	1 (8)	2 (17)	0 (0)	0 (0)	0 (0)	0 (0)	0 (0)	0 (0)	1 (0.7)	0 (0)
Neoplasms benign, malignant and unspecified (incl cysts and polyps)	0 (0)	0 (0)	0 (0)	0 (0)	0 (0)	0 (0)	1 (0.7)	0 (0)	0 (0)	1 (0.7)
Nervous system disorders	0 (0)	0 (0)	0 (0)	2 (17)	0 (0)	0 (0)	0 (0)	0 (0)	0 (0)	1 (0.7)
Psychiatric disorders	0 (0)	0 (0)	0 (0)	0 (0)	0 (0)	0 (0)	1 (0.7)	2 (1)	1 (0.7)	4 (3)
Reproductive system and breast disorders	0 (0)	2 (17)	0 (0)	0 (0)	0 (0)	0 (0)	0 (0)	1 (0.7)	2 (1)	0 (0)
Respiratory, thoracic and mediastinal disorders	0 (0)	1 (8)	0 (0)	2 (17)	0 (0)	0 (0)	37 (27)	31 (22)	49 (35)	35 (25)
Skin and subcutaneous tissue disorders	0 (0)	1 (8)	0 (0)	0 (0)	0 (0)	0 (0)	43 (31)	45 (32)	54 (39)	50 (36)

Cells show number and percent of participants with any AE or SAE.

Supplementary Table 5: Listing of serious adverse events during the study period according to treatment group

Treatment group	Adverse event description (MedDRA© preferred term)	Onset of event	Severity	Outcome
Toddlers				
30µg	Febrile convulsion	21 d post injection	Moderate	Resolved
90µg	Febrile convulsion	107 d post injection	Moderate	Resolved
Infants				
15µg (18 SAEs in 13 infants)	Pneumonia	5 d post injection 1	Moderate	Resolved
	Pyelonephritis[1]	10 d post injection 1	Moderate	Resolved
	Bronchiolitis	3 d post injection 2	Moderate	Resolved
	Pneumonia	22 d post injection 1	Moderate	Resolved
	Upper respiratory tract infection	10 d post injection 2	Moderate	Resolved
	Bronchiolitis[1]	20 d post injection 2	Moderate	Resolved
	Lower respiratory tract infection[2]	28 d post injection 2	Moderate	Resolved
	Pneumonia	1 d post injection 3	Moderate	Resolved
	Bronchiolitis[3]	7 d post injection 3	Severe	Resolved
	Bronchiolitis[1]	17 d post injection 3	Moderate	Resolved
	Pneumonia[3]	37 d post injection 3	Severe	Resolved
	Gastroenteritis	48 d post injection 3	Moderate	Resolved
	Bronchiolitis	74 d post injection 3	Death	Death
	Bronchiolitis	76 d post injection 3	Moderate	Resolved
	Bronchiolitis[2]	100 d post injection 3	Moderate	Resolved
	Lower respiratory tract infection[4]	164 d post injection 3	Severe	Resolved
	HIV infection[4]	168 d post injection 3	Severe	Ongoing at final assessment
	Bronchiolitis	175 d post injection 3	Moderate	Resolved
30µg (7 SAEs in 6 infants)	Meningitis pneumococcal	20 d post injection 1	Death	Death
	Gastroenteritis	5 d post injection 2	Moderate	Resolved
	Lower respiratory tract infection	22 d post injection 2	Severe	Resolved
	Abscess soft tissue	24 d post injection 3	Moderate	Resolved
	Lower respiratory tract infection[5]	34 d post injection 3	Severe	Resolved
	Encephalitis[5]	34 d post injection 3	Severe	Resolved
	Bronchiolitis	107 d post injection 3	Severe	Resolved
90µg (11 SAEs in 8 infants)	Urinary tract infection	0 d post injection 2	Severe	Resolved
	Head injury[6]	0 d post injection 3	Moderate	Resolved
	Pneumonia	18 d post injection 3	Moderate	Resolved
	Respiratory syncytial virus bronchiolitis[6]	24 d post injection 3	Severe	Resolved
	Gastroenteritis	29 d post injection 3	Severe	Resolved
	Bronchiolitis	27 d post injection 3	Severe	Resolved
	H1N1 influenza[6]	77 d post injection 3	Severe	Resolved
	Upper respiratory tract infection	78 d post injection 3	Moderate	Resolved
	Gastroenteritis[6]	84 d post injection 3	Moderate	Resolved
	Bronchiolitis	122 d post injection 3	Moderate	Resolved
	Cellulitis	174 d post injection 3	Severe	Resolved
Placebo (9 SAEs in 8 infants)	Urinary tract infection	1 d post injection 1	Moderate	Resolved
	Seizure	1 d post injection 1	Moderate	Resolved
	Bronchiolitis	2 d post injection 3	Moderate	Resolved
	Gastroenteritis	26 d post injection 3	Moderate	Resolved
	Bronchiolitis[7]	51 d post injection 3	Moderate	Resolved
	Diarrhoea[7]	53 d post injection 3	Moderate	Resolved
	Sudden infant death syndrome	60 d post injection 3	Death	Death
	Bronchiolitis	73 d post injection 3	Moderate	Resolved
	Pneumonia	89 d post injection 3	Severe	Resolved

There were no SAEs reported in adults.

Seven infants had >1 SAE during the study period – SAEs from the same participant are identified by a number (from 1-7).

Supplementary Table 6: Serum neutralising antibody responses (4-fold increase) pre-vaccination and four weeks after the third injection of P2-VP8-P[8] or placebo in infants, according to treatment group

	15μg	30μg	90μg	Placebo
**Wa strain**	n=132	n=132	n=134	n=130
Seroresponse –unadjusted n (%; 95% CI)	20 (15; 10–22)	23 (17; 11–25)	28 (21; 14–29)	4 (3; 1–8)
Seroresponse –adjusted* n (%; 95% CI)	73 (55; 46–64)	95 (72; 63–79)	92 (69; 60–76)	17 (13; 8–20)
p-value+	<0·0001	<0·0001	<0·0001	–
**DS-1 strain**	n=131	n=132	n=134	n=130
Seroresponse –unadjusted n (%; 95% CI)	20 (15; 10–23)	20 (15; 10–22)	24 (18; 12–25)	3 (2; 0·5–7)
Seroresponse –adjusted* n (%; 95% CI)	85 (65; 56–73)	96 (73; 64–80)	93 (69; 61–77)	17 (13; 8–20)
p-value+	<0·0001	<0·0001	<0·0001	–
**1076 strain**	n=132	n=132	n=134	n=130
Seroresponse –unadjusted n (%; 95% CI)	11 (8; 4–14)	24 (18; 12–26)	23 (17; 11–25)	4 (3; 1–8)
Seroresponse –adjusted* n (%; 95% CI)	73 (55; 46–64)	86 (65; 56–73)	98 (73; 65–80)	13 (10; 5–16)
p-value+	<0·0001	<0·0001	<0·0001	–

*Neutralising antibody post-injection titres were adjusted for decay in maternal antibodies using the half-life calculated from participants in the placebo group who had detectable baseline titres that were higher than at the post-injection visit, and established for each assay separately. Half-life (days) of 35·9 for Wa, 30·1 for DS-1, and 34·8 for 1076. Adjusted seroresponse was defined as 4-fold increase in titre between baseline and four weeks after the third injection (adjusted titre) in infants with an unadjusted post-injection titre greater than the limit of detection (10).

+ P value indicates pair-wise comparison of each vaccine dose group to placebo. None of the pairwise between-group differences among the active dose groups was statistically significant except for: strain Wa, the seroresponse rate (55%) in the 15μg group was significantly lower than in the 30μg group (72%, p=0.0047) and the 90μg group (69%, p=0.0246); for strain 1076, the seroresponse rate (55%) in the 15μg group was significantly lower than in the 90μg group (73%, p=0.0023).

Supplementary Table 7: Serum IgA, IgG and neutralising antibody responses four weeks after the second injection of trivalent P2-VP8 or placebo in infants, according to treatment group

Seroresponse n/N (%; 95% CI)	15μg	p-value+	30μg	p-value+	90μg	p-value+	Placebo
Anti-P2-VP8 IgG to P[4] - adjusted*	126/137 (92; 86–96)	<0·0001	121/131 (92; 86–96)	<0·0001	131/136 (96; 92–99)	<0·0001	7/135 (5; 2–10)
Anti-P2-VP8 IgG to P[6] - adjusted*	136/137 (99; 96–100)	<0·0001	125/131 (95; 90–98)	<0·0001	133/136 (98; 94–100)	<0·0001	25/135 (19; 12–26)
Anti-P2-VP8 IgG to P[8] - adjusted*	126/137 (92; 86–96)	<0·0001	119/131 (91; 85–95)	<0·0001	131/136 (96; 92–99)	<0·0001	7/134 (5; 2–10)
Anti-P2-VP8 IgA to P[4] – unadjusted#	22/136 (6; 10–23)	0·0012	29/131 (22; 15–30)	<0·0001	20/136 (15; 9–22)	0·0035	6/134 (5; 2–9)
Anti-P2-VP8 IgA to P[6] – unadjusted#	22/137 (16; 10–23)	0·0719	33/131 (25; 18–34)	0·0003	27/136 (20; 14–28)	0·0093	12/135 (9; 5–15)
Anti-P2-VP8 IgA to P[8] – unadjusted#	19/136 (14; 9–21)	0·0056	22/130 (17; 11–25)	0·0007	18/135 (13; 8–20)	0·0088	6/135 (4; 2–9)
Neutralising antibodies to Wa strain – adjusted^	49/137 (36; 28–44)	<0·0001	51/131 (39; 31–48)	<0·0001	49/136 (36; 28–45)	<0·0001	16/135 (12; 7–19)
Neutralising antibodies to DS-1 strain – adjusted^	38/137 (28; 20–36)	0·0058	44/131 (34; 26–42)	0·0002	4136 (35; 27–44)	<0·0001	19/134 (14; 9–21)
Neutralising antibodies to 1076 strain – adjusted^	29/137 (21; 15–29)	0·0078	33/131 (25; 18–34)	0·0007	32/136 (24; 17–32)	0·0018	13/135 (10; 5–16)

*IgG antibody post-injection titres were adjusted for decay in maternal antibodies using the half-life calculated from participants in the placebo group who had detectable baseline titres that were higher than at the post-injection visit, and established for each assay separately. Half-life (days) for IgG: 36·6 for [P4], 49·1 for [P6], and 38·3 for [P8]. Adjusted seroresponse was defined as ≥4-fold increase in titre between baseline and four weeks after the third injection (adjusted titre) in infants with an unadjusted post-injection titre greater than the limit of detection (15).

IgA seroresponse was defined as ≥4-fold increase in titre between baseline and four weeks after the third injection in infants with a post-injection titre greater than the limit of detection (9).

^Neutralising antibody post-injection titres were adjusted for decay in maternal antibodies using the half-life calculated from participants in the placebo group who had detectable baseline titres that were higher than at the post-injection visit, and established for each assay separately. Half-life (days) of 35·9 for Wa, 30·1 for DS-1, and 34·8 for 1076. Adjusted seroresponse was defined as ≥2·7-fold increase in titre between baseline and four weeks after the third injection (adjusted titre) in infants with an unadjusted post-injection titre greater than the limit of detection (10).

+ P value indicates pair-wise comparison of each vaccine dose group to placebo. No significant pairwise between-group differences were observed among the active dose groups, except for 30μg (95%) vs 15μg (99%) for Anti-P2-VP8 IgG to P[6] (p=0·0383).

Supplementary Table 8: Serum IgA, IgG and neutralising antibody responses four weeks after the third injection of trivalent P2-VP8 or placebo in adults, according to treatment group

**Seroresponse n/N (%)**	**30μg**	**p-value+**	**90μg**	**p-value+**	**Placebo**
Anti-P2-VP8 IgG to P[4]	11/11 (100)	0·0015	11/11 (100)	0·0015	0/4 (0)
Anti-P2-VP8 IgG to P[6]	10/11 (91)	0·0004	11/11 (100)	<0·0001	0/4 (0)
Anti-P2-VP8 IgG to P[8]	11/11 (100)	0·0015	11/11 (100)	0·0015	0/4 (0)
Anti-P2-VP8 IgA to P[4]	11/11 (100)	0·0015	11/11 (100)	0·0015	0/4 (0)
Anti-P2-VP8 IgA to P[6]	9/11 (82)	0·0018	9/11 (82)	0·0018	0/4 (0)
Anti-P2-VP8 IgA to P[8]	11/11 (100)	<0·0001	10/11 (91)	0·0004	0/4 (0)
Neutralising antibodies to Wa strain	3/11 (27)	0·1453	8/11 (73)	0·0051	0/4 (0)
Neutralising antibodies to DS-1 strain	5/11 (46)	0·0472	4/11 (35)	0·0845	0/4 (0)
Neutralising antibodies to 1076 strain	2/11 (18)	0·2454	3/11 (27)	0·1453	0/4 (0)

IgA and IgG seroresponse was defined as ≥4-fold increase in titre between baseline and four weeks after the third injection; neutralising antibody seroresponse was defined as ≥2·7-fold increase in titre between baseline and four weeks after the third injection.

+ P-value indicates pair-wise comparison of vaccine dose group to placebo.

No significant differences were observed in Anti-P2-VP8 IgG and IgA seroresponses between the 30μg and 90μg dose groups. The seroresponse for strain Wa after the third injection was significantly higher in the 90μg (73%) than the 30 μg group (27%, p=0.0299).

Supplementary Table 9: Serum IgA, IgG and neutralising antibody responses four weeks after the single injection of trivalent P2-VP8 or placebo in toddlers, according to treatment group

Seroresponse n/N (%)	30μg	p-value+	90μg	p-value+	Placebo
Anti-P2-VP8 IgG to P[4]	7/12 (58)	0·0054	11/12 (92)	<0·0001	0/6 (0)
Anti-P2-VP8 IgG to P[6]	9/12 (75)	0·0007	11/12 (92)	<0·0001	0/6 (0)
Anti-P2-VP8 IgG to P[8]	10/12 (83)	0·0002	10/12 (83)	0·0002	0/6 (0)
Anti-P2-VP8 IgA to P[4]	8/12 (67)	0·0021	11/12 (92)	<0·0001	0/6 (0)
Anti-P2-VP8 IgA to P[6]	6/12 (50)	0·0122	8/12 (67)	0·0021	0/6 (0)
Anti-P2-VP8 IgA to P[8]	10/12 (83)	0·0002	10/12 (83)	0·0002	0/6 (0)
Neutralising antibodies to Wa strain	1/12 (8)	0·3594	5/12 (42)	0·0258	0/6 (0)
Neutralising antibodies to DS-1 strain	1/12 (8)	0·3594	3/12 (25)	0·0988	0/6 (0)
Neutralising antibodies to 1076 strain	0/12 (0)	>0·999	2/12 (17)	0·1866	0/6 (0)

IgA and IgG seroresponse was defined as ≥4-fold increase in titre between baseline and four weeks after the single injection; neutralising antibody seroresponse was defined as ≥2·7-fold increase in titre between baseline and four weeks after the single injection.

+ P-value indicates pair-wise comparison of vaccine dose group to placebo.

No significant differences were observed in Anti-P2-VP8 IgG and IgA or neutralising antibody seroresponses between the 30μg and 90μg dose groups.

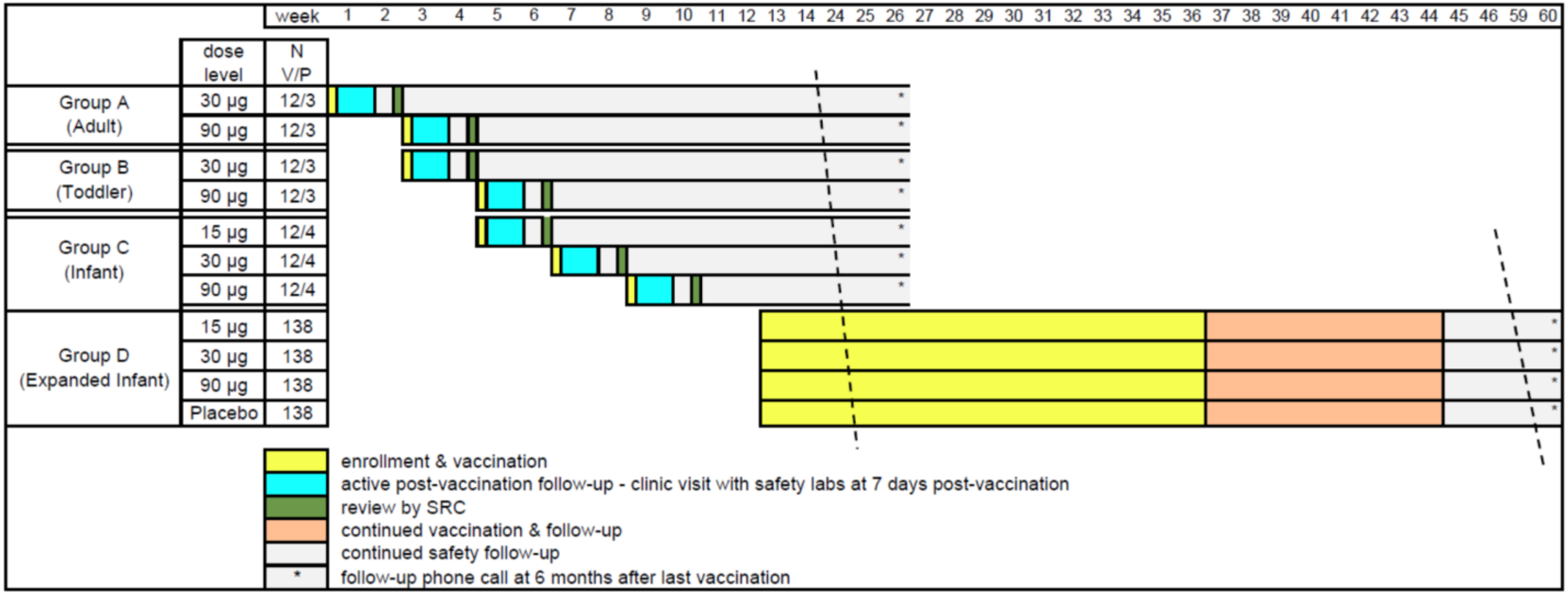

Supplementary Figure 1: Study overview and projected timeline

V=vaccine; P=placebo; Groups A, B and C enrolled at RMPRU site only; Group D enrolled at all three study sites

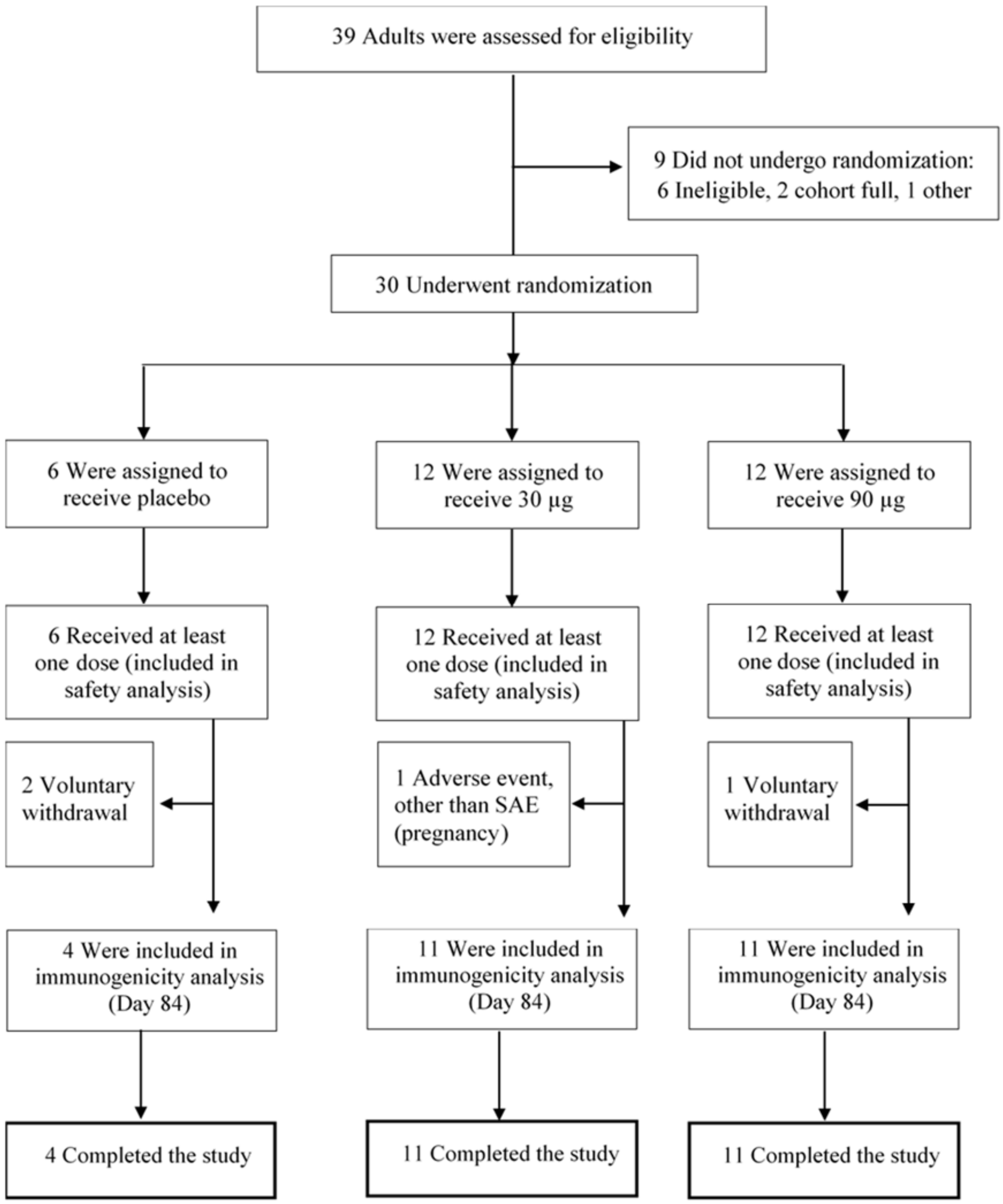

Supplementary Figure 2: Trial profile - adults

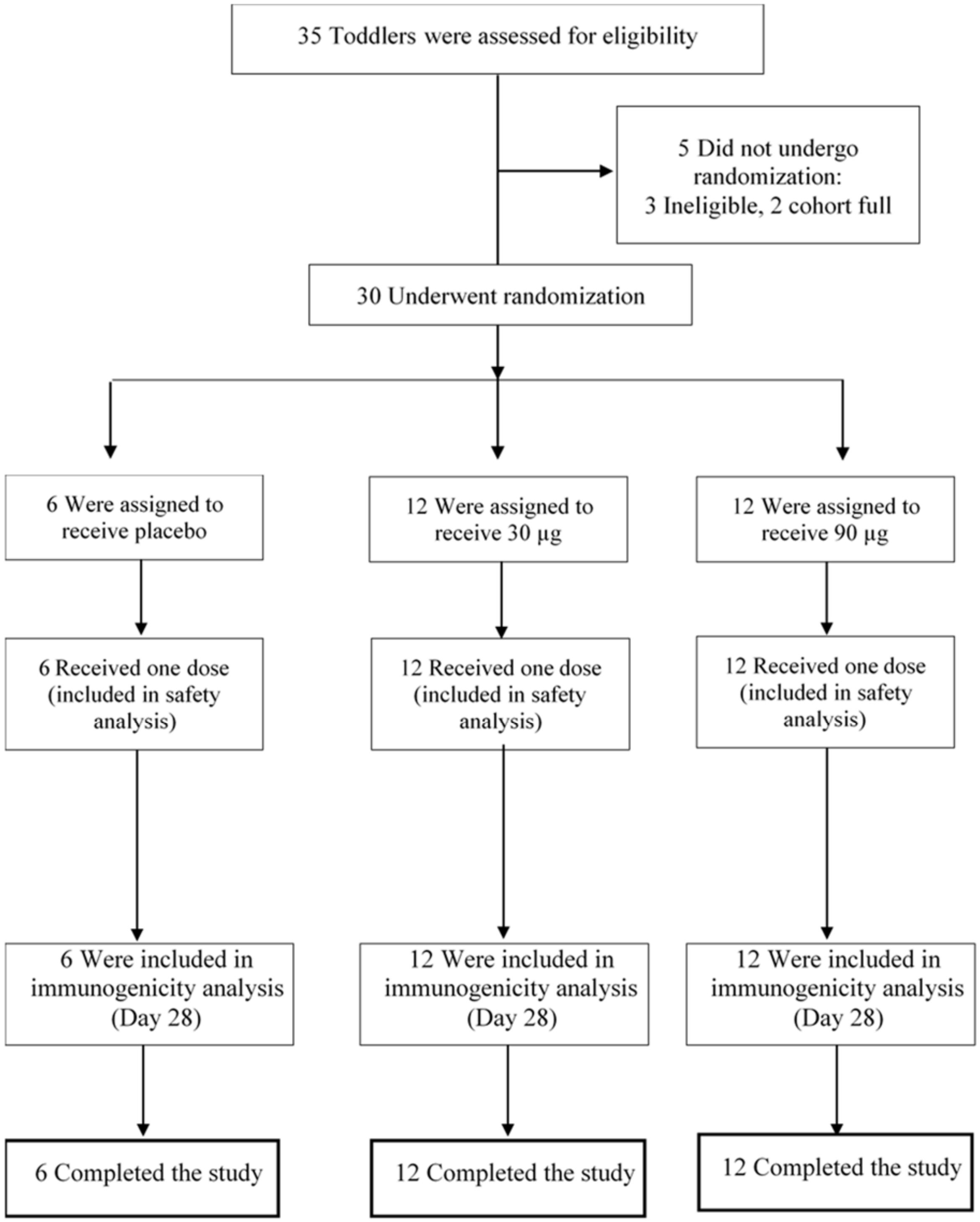

Supplementary Figure 3: Trial profile - toddlers

IgG P[4]

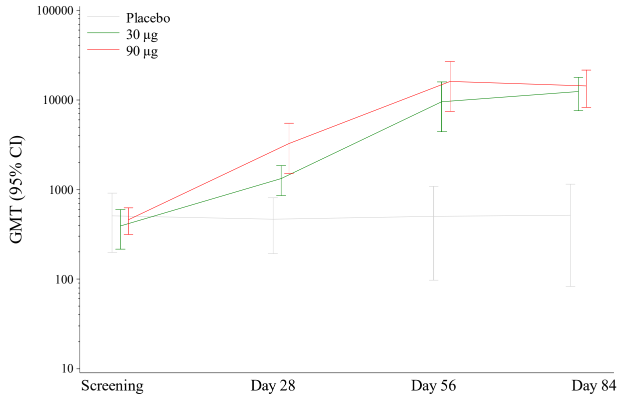

IgA P[4]

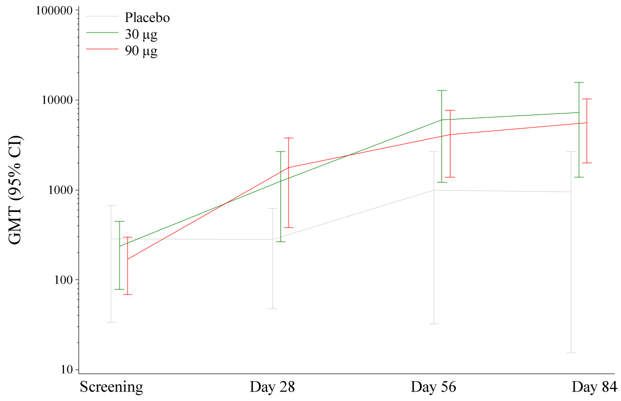

IgG P[6]

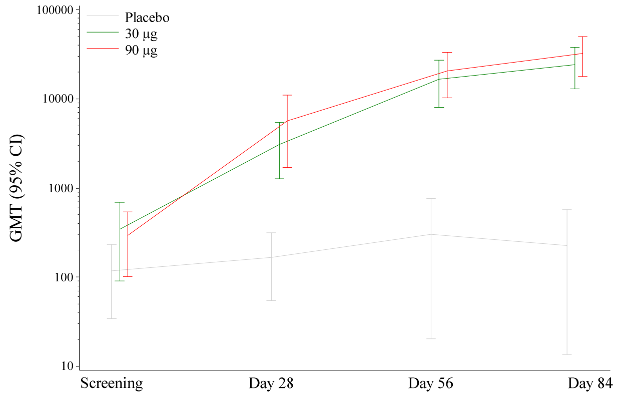

IgA P[6]

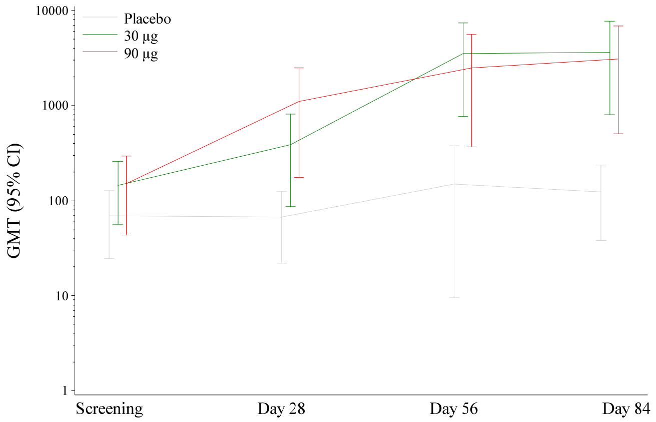

IgG P[8]

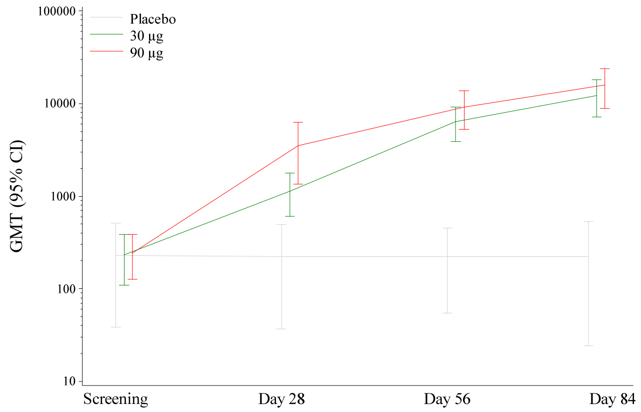

IgA P[8]

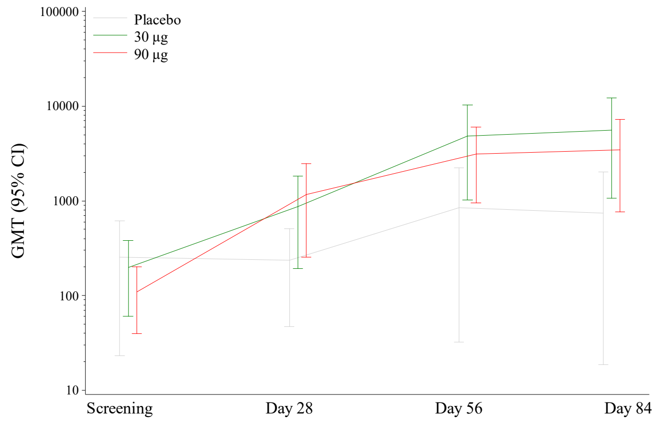

Supplementary Figure 4: Serum IgA and IgG antibody responses four weeks after each injection of trivalent P2-VP8 or placebo injection in adults, according to treatment group

IgG P[4]

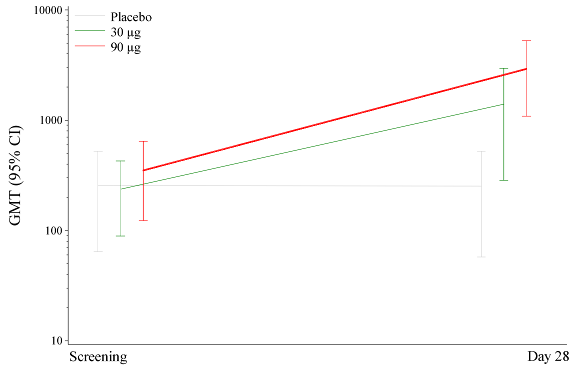

IgA P[4]

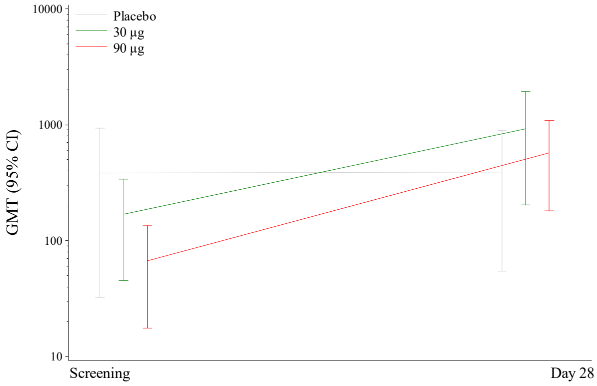

IgG P[6]

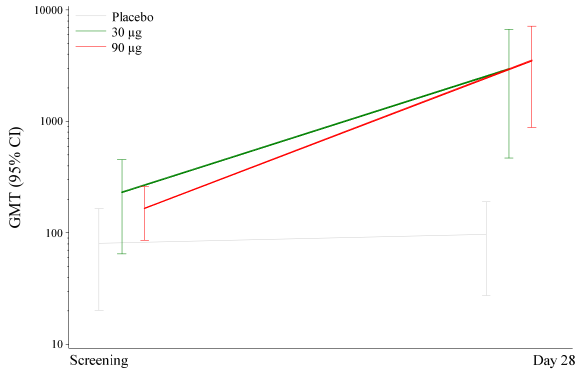

IgA P[6]

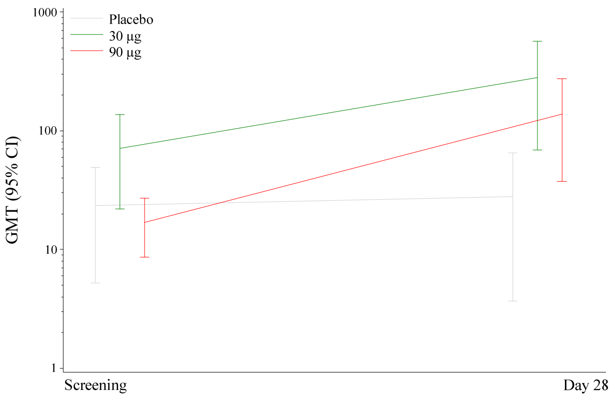

IgG P[8]

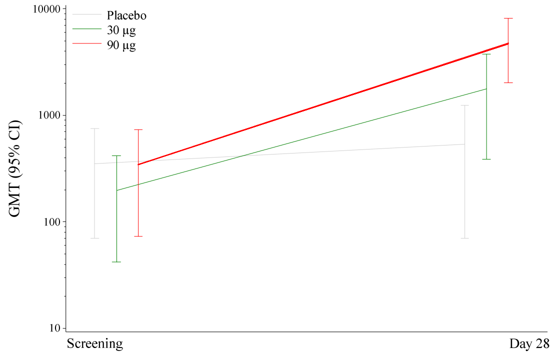

IgA P[8]

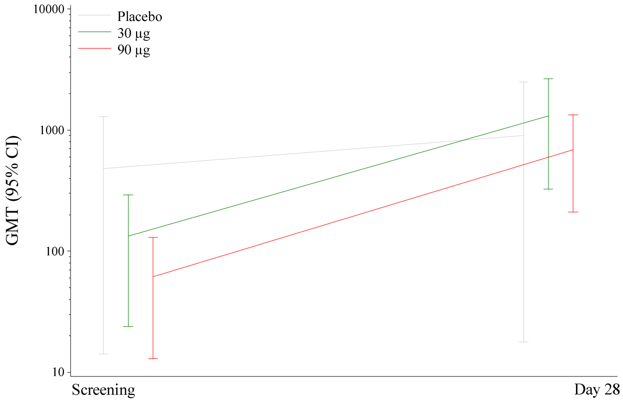

Supplementary Figure 5: Serum IgG and IgA antibody responses four weeks after single injection of trivalent P2-VP8 or placebo injection in toddlers, according to treatment group

Wa strain - adults

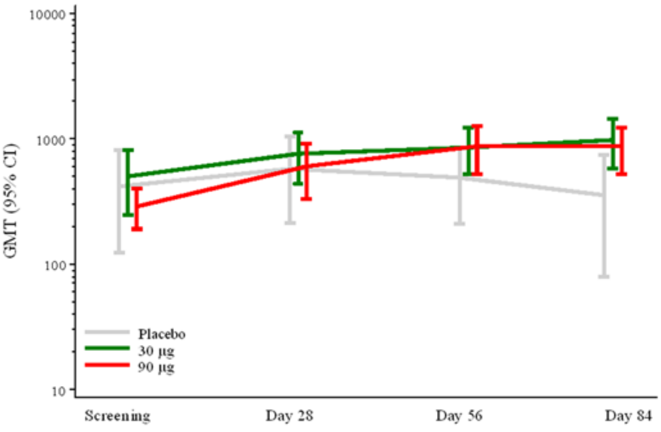

Wa strain - toddlers

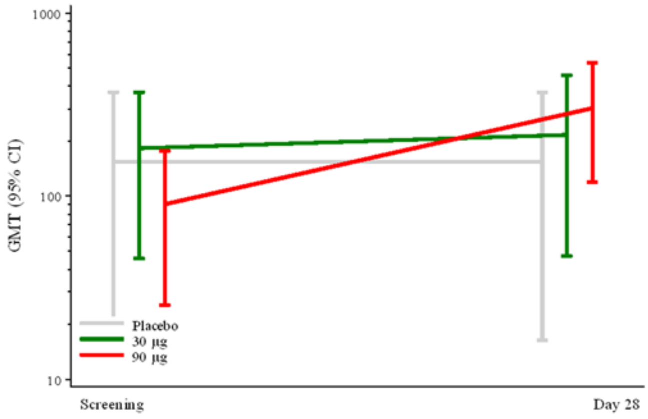

DS-1 strain - adults

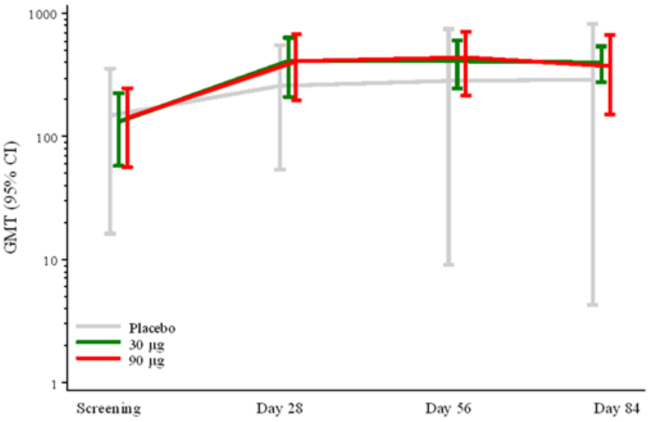

DS-1 strain - toddlers

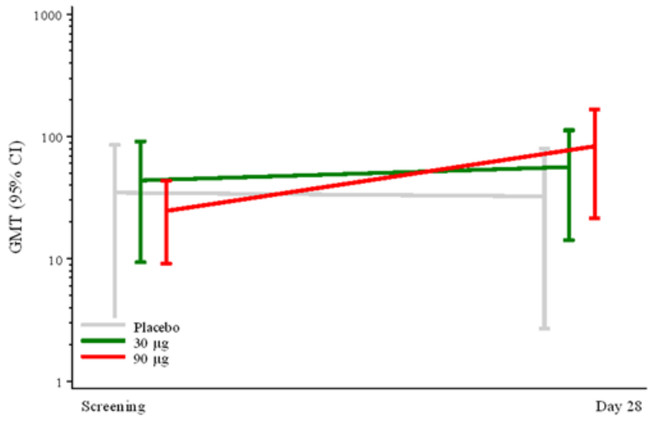

1076 strain - adults

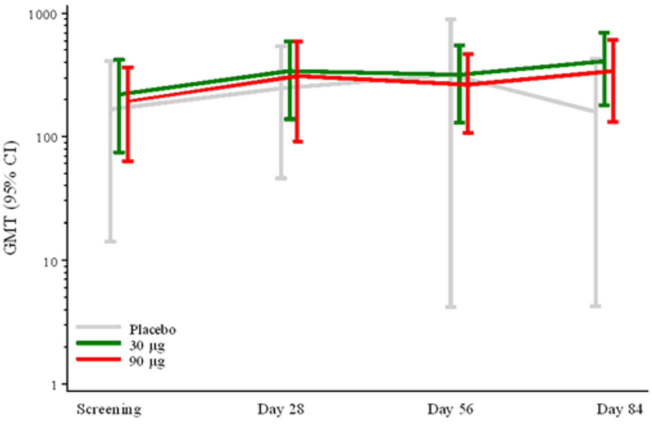

1076 strain - toddlers

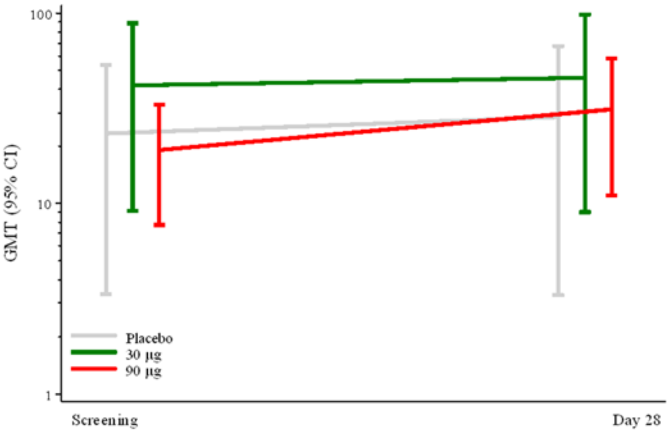

Supplementary Figure 6: Serum neutralising antibody responses four weeks after injection of trivalent P2-VP8 or placebo injection in adults (three injections) and toddlers (single injection), according to treatment group

Protocol

VAC 041

A Phase I/II double-blind, randomized, placebo-controlled, descending-age, dose-escalation study to examine the safety, tolerability and immunogenicity of the trivalent P2-VP8 subunit rotavirus vaccine in healthy South African adults, toddlers and infants

16.1.1 Protocol and Protocol Amendments

VAC 041

A Phase I/II double-blind, randomized, placebo-controlled, descending-age, dose-escalation study to examine the safety, tolerability and immunogenicity of the trivalent P2-VP8 subunit rotavirus vaccine in healthy South African adults, toddlers and infants

CONFIDENTIAL

21 June 2016

Sponsored by:

PATH Vaccine Solutions

National Principal Investigator:

Michelle Groome, MBBCh, DCH, MSc Med

Version 4.0

Confidentiality Statement

This document is confidential and is to be distributed for review only to investigators, potential investigators, consultants, study staff, and applicable Independent Ethics Committees or Institutional Review Boards. The contents of this document shall not be disclosed to others without written authorization from PVS (or others, as applicable), unless it is necessary to obtain informed consent from parents of potential study participants.

TABLE OF CONTENTS

List of ABBREVIATIONS and ACRONYMS viii
PROTOCOL SIGNATURE PAGE 1
PARTICIPATING INSTITUTIONS 2
STUDY SYNOPSIS 4
1. BACKGROUND AND INTRODUCTION 16
1.1. Burden of Disease 16
1.2. Pathogen 16
1.3. Non-replicating Rotavirus Vaccine Approach Using VP8 Subunit 17
1.4. Potential Safety Risks 18
1.5. Summary of Nonclinical Studies with Monovalent (P[8]) P2-VP8 18
1.5.1. Non-GLP Nonclinical Pharmacology/Proof of Concept Studies 19
1.5.1.1. Evaluation of the Antibody Response in Guinea Pigs Administered Monovalent (P[8]) VP8 or P2-VP8 19
1.5.1.2. Evaluation of the Antibody Response in Guinea Pigs Administered Monovalent (P[8]) P2-VP8 with or without Aluminum Phosphate Adjuvant 20
1.5.1.3. Evaluation of the Rotavirus Neutralizing Antibody Response in Guinea Pigs 20
1.5.1.4. Protection from Rotavirus Disease in Neonatal Piglets 21
1.5.2. GLP Immunogenicity and Toxicology Study in NZW Rabbits 23
1.5.2.1. Study Description 23
1.5.2.2. Toxicology Results 24
1.5.2.3. Immunogenicity Results 25
1.6. Summary of Nonclinical Studies with Trivalent (P[4], P[6], P[8]) P2-VP8 Vaccine 27
1.6.1. Evaluation of the Antibody Response in Guinea Pigs Administered Either Trivalent or Monovalent P2-VP8 Vaccine 27
1.6.2. GLP Immunogenicity and Toxicology Study of the Trivalent P2-VP8 Subunit Vaccine in NZW Rabbits 28
1.6.2.1. Study Description 29
1.6.2.2. Toxicology Results 29
1.6.2.3. Immunogenicity Results 30
1.7. Clinical Studies 31
1.7.1. First-in-human testing of monovalent (P[8]) P2-VP8 subunit vaccine in adults (VAC 009) 31
1.7.2. Clinical testing of monovalent (P[8]) P2-VP8 subunit vaccine in toddlers and infants in South Africa (VAC 013) 36
2. HYPOTHESIS, SCIENTIFIC RATIONALE, OBJECTIVES AND STUDY DESIGN 42
2.1. Study Hypothesis 42
2.2. Scientific Rationale 42

2.3. Overall Clinical Development Strategy 43
2.4. Study Design 44
2.4.1. Primary Objectives...44
2.4.2. Secondary Objectives 44
2.4.3. Exploratory Objective..45
2.4.4. Primary Endpoints...45
2.4.5. Secondary Endpoints:..45
2.4.6. Exploratory Endpoint...46
3. STUDY PRODUCT 46
3.1. Product Description..46
3.2. Products and Manufacturers..46
3.3. Presentation and Formulation 47
3.4. Stability and Storage...47
3.5. Preparation and Administration...47
3.5.1. Dose Preparation and Administration 48
3.5.2. Accountability and Disposal 48
4. STUDY POPULATION 49
4.1. Clinical Trial Sites 49
4.2. Study Population...49
4.3. Eligibility 49
4.4. Inclusion Criteria..49
4.5. Exclusion Criteria...50
5. STUDY PROCEDURES...51
5.1. Recruitment 51
5.2. Screening, Randomization, and Masking Procedures (for all age-groups)..........51
5.2.1. Initial and Continuing Informed Consent...51
5.2.2. Screening – Day -7 (infants) or Day -28 (adults and toddlers) to Day -1 52
5.2.3. Randomization 52
5.2.4. Masking procedures...53
5.2.5. Group A (adults)..53
5.2.5.1. Day 0 – First Dose 53
5.2.5.2. Day 7, 35 and 63 ± 1 day (to be defined by 7 days after most recent study injection day) – Clinic visit follow-up...54
5.2.5.3. Day 28 (-2 to +7 days) and Day 56 (-2 to +7 days) days (to be defined by 28 days after the previous study injection) – Study injection days...55
5.2.5.4. Day 84 ± 4 days (to be defined by 28 days post-3rd study injection day) – Clinic follow-up visit and immunogenicity sampling 56
5.2.5.5. Day 224 ± 14 days (to be defined by 168 days post-3rd study injection day) – Final participant contact (telephone or clinic visit) 56
5.2.6. Group B (toddlers) 56
5.2.6.1. Day 0 – Study Injection Day 56

5.2.6.2. Day 3 (to be defined by 3 days after study injection day) – Clinic follow-up visit....57
5.2.6.3. Day 7 ± 1 day (to be defined by 7 days after study injection day) – Clinic follow-up visit....58
5.2.6.4. Day 28 ± 4 days (to be defined by 28 days after study injection day) – Clinic follow-up....58
5.2.6.5. Day 168 ± 14 days (to be defined by 168 days post-injection day) – Final participant contact (telephone or clinic visit)....59
5.2.7. Groups C and D (infants)....59
5.2.7.1. Day 0 – First Dose....59
5.2.7.2. Day 3, 31 and 59 (to be defined by 3 days after most recent study injection day) – Clinic visit follow-up....60
5.2.7.3. Day 7, 35 and 63 ± 1 day (to be defined by 7 days after most recent study injection day) – Clinic visit follow-up....60
5.2.7.4. Day 28 (-2 to +7 days) (to be defined by 28 days after the previous study injection) – Study injection day....61
5.2.7.5. Day 56 (-2 to +7 days) (to be defined by 28 days after the previous study injection) – Study injection day....62
5.2.7.6. Day 84 ± 4 days (to be defined by 28 days post-3rd Study injection day) – Clinic follow-up visit, immunogenicity sampling and first dose of Rotarix....63
5.2.7.7. Day 89 – Clinic visit for stool sampling (RMPRU only)....64
5.2.7.8. Day 91 – Clinic visit for stool sampling (RMPRU only)....64
5.2.7.9. Day 93 – Clinic visit for stool sampling (RMPRU only)....64
5.2.7.10. Day 112 and 140 + 14 days (to be defined by 28 days after the previous Rotarix vaccination) – Clinic visit for second and third dose of Rotarix 64
5.2.7.11. Day 224 ± 14 days (to be defined by 168 days post-3rd injection day) – Final contact (telephone or clinic visit)....64

5.3. Interim Contacts and Unscheduled Visits....65

5.4. Stool Samples from Infants with Diarrhea....65

5.5. Early Termination....65

5.6. Procedure Methods....65

5.6.1. Vital Signs....65
5.6.2. Height/length and Weight....65
5.6.3. Physical Examination....65
5.6.4. Medical History....65
5.6.5. Injection Site Examination....66
5.6.6. Reactogenicity Assessment....66
5.6.7. Clinical Laboratory Testing....66
5.6.8. Withdrawal from Further Study Injection and Early Termination from Study....67
5.6.9. Interim Contacts and Visits....67
5.6.10. Contraception and Pregnancy....67

6. LABORATORY EVALUATIONS....68

6.1. Sample collection, distribution and storage....68

6.2. Safety clinical laboratory assays....68

6.3. Immunological assays 68
6.4. Assay qualification, standardization and validation 69
6.5. Stool testing for rotavirus 69
6.6. Rotarix shedding 69
6.7. Biohazard Containment 70
7. STATISTICAL DESIGN AND ANALYSIS 70
7.1. Overview and General Design 70
7.2. Randomization and blinding procedures 70
7.3. Objectives and Endpoints 71
7.3.1. Primary Objectives: 71
7.3.2. Secondary Objectives 72
7.3.3. Exploratory Objective 73
7.4. Sample Size 73
7.4.1. Primary Safety Endpoints 73
7.4.2. Primary Immunogenicity Endpoints 74
7.5. Analytical Methodology 75
8. SAFETY ASSESSMENT AND REPORTING 77
8.1. Safety Events 77
8.2. Reporting Period 77
8.3. Definitions 78
8.3.1. Adverse Event (AE) or Medical Event 78
8.3.2. Serious Adverse Events (Defined as Serious Adverse Events, Serious Suspected Adverse Reactions or Serious Adverse Reactions) 78
8.3.3. Unexpected Adverse Event 78
8.4. Toxicity Grading 78
8.4.1. Guidelines for Determining Causality of an Adverse Event 79
8.5. Adverse Event Identification, Resolution and Reporting 79
8.5.1. AE Resolution 80
8.5.2. General Recording and Reporting Procedures 80
8.5.3. SAE Recording and Reporting Procedures 80
8.5.4. Site Reporting of Events 81
8.6. Serious Adverse Event Notification 81
8.6.1. Notifications and Review 81
8.6.2. Expedited Reporting 82
8.6.3. Notifying the Protocol Safety Review Committee 83
8.6.4. Notifying the Ethics Committee/Institutional Review Board 83
9. SAFETY MONITORING 83
9.1. Safety Review Committee 83
9.2. Data and Safety Monitoring Board (DSMB) 83

9.3. Withdrawal for Safety....84

9.4. Study Pause....84

9.4.1. Study Pause Rules....84

9.4.2. Study Pause Procedure....85

9.5. Dose Escalation....85

10. DATA MANAGEMENT....86

10.1. Case Report Form Development and Completion....86

10.2. Details of Data Management....86

10.2.1. Data Validation....86

10.2.2. Source Data Verification....86

10.2.3. Definition of Source Data....87

10.2.4. Definition of Source Document....87

10.3. Database Locking Procedures....87

10.4. Record Archival....87

10.5. Screen Failures....87

10.6. Protocol Deviations....87

11. STUDY MONITORING....88

11.1. Independent Auditing....88

11.2. Regulatory Agency Auditing....89

12. OBLIGATIONS AND ROLES OF THE SPONSOR, SITE PIS AND STUDY PERSONNEL....89

13. ETHICAL CONSIDERATIONS AND INFORMED CONSENT....90

13.1. Informed Consent Process....90

13.2. Risk/Benefit....90

13.3. Protocol Review Process....90

13.4. Participant Confidentiality....91

13.5. Reimbursement....91

13.6. Storage of Specimens....91

14. APPENDICES....91

14.1. APPENDIX I: Schedule of Events....92

14.1.1. Schedule of Events for Group A (Adults)....92

14.1.2. Schedule of Events for Group B (Toddlers)....93

14.1.3. Schedule of Events for Groups C & D (Infants)....94

14.2. APPENDIX II: Toxicity Grading Scales....95

14.3. APPENDIX III: Memory Aid....98

14.4. APPENDIX IV: Literature Cited....99

LIST OF TABLES AND FIGURES

Figure 1: Study overview and timeline ... 15
Figure 2: Continental Variation in the Distribution of Human Group A Rotavirus P Types Ascertained by Analysis of Strains Collected between 1982-2003 ... 17
Table 1: Preclinical Studies Supporting P2-VP8 Subunit Vaccine Development ... 19
Table 2: Geometric Mean Neutralizing Titers to Human Rotavirus Strains: Before and After Immunization ... 21
Table 3: Seroconversion to Rotavirus Strains Following Immunization ... 21
Figure 3: Rotavirus Geometric Mean Neutralizing Antibody Titers Following Vaccination and Challenge ... 22
Figure 4: Days to Onset of Diarrhea and Viral Shedding by Treatment ... 23
Table 4: Geometric Mean Titers by Treatment Following Four Doses of Monovalent (P[8]) P2-VP8 Subunit Vaccine with or without Aluminum Hydroxide [$Al(OH)_3$] ... 26
Figure 5: Geometric Mean Titers by Interval and Treatment ... 26
Table 5: Preclinical Studies Supporting Trivalent Rotavirus P2-VP8 Subunit Vaccine Development ... 27
Table 6: Serum IgG Binding Antibody Responses in Guinea Pigs following vaccination with either Monovalent or Trivalent P-VP8 Vaccines. ... 28
Table 7. Neutralizing Antibody titers to Strains of Rotavirus Expressing Various P Types ... 28
Table 8. Serum IgG Binding Antibody Responses in NZW Rabbits Following Vaccination with 180 µg Trivalent P2-VP8 Vaccine (Study Day 45) ... 30
Table 9: Maximum Injection Site Reactogenicity per Participant ... 31
Table 10: Maximum Systemic Reactogenicity per Participant ... 32
Table 11: Maximum Adverse Event Severity per Participant ... 32
Figure 6: Serum Anti-P2-VP8 IgG Titers by ELISA ... 33
Table 12: Proportion of Participants with ≥4-fold Increase in Anti-P2-VP8 IgG Titers by ELISA33
Figure 7: Serum Anti-P2-VP8 IgA Titers by ELISA ... 34
Table 13: Proportion of Participants with ≥4-fold Increase in Anti-P2-VP8 IgA Titers by ELISA34
Figure 8: Serum Neutralizing Antibody Titers against Wa Strain ... 35
Table 14: Proportion of Participants with ≥4-fold Increase in Neutralizing Antibody Titers against Wa Strain ... 35
Table 15: Proportion of Participants with ≥4-fold Increase in Neutralizing Antibody Titers at Day 84 against Other Strains ... 35
Table 16: Participants with Detectable Antibody in Lymphocyte Supernatant (ALS) to P2-VP8 Antigen 7 Days after the Third Study Injection ... 36
Table 17: Maximum Injection Site Reactogenicity per Participant in VAC 013 ... 37
Table 18: Maximum Systemic Reactogenicity per Participant in VAC 013 ... 39
Table 19: Maximum Adverse Event Severity per Participant in VAC 013 ... 41

Table 20: Anti-P2-VP8 IgG Responses in Infants 41
Table 21: Anti-P2-VP8 IgA Responses in Infants 41
Table 22: Neutralizing Antibody Responses to Wa Strain in Infants 42
Table 23: Rotarix Shedding in Infants 42
Table 24: Dosing Schedule 48
Table 25: Probabilities of observing AEs 74
Table 26: Power calculations for Comparing GMT in Two Groups of Infants (Cohorts C and D combined) 75
Figure 9: Reporting Decisions for Adverse Events 81

VAC 041

LIST OF ABBREVIATIONS AND ACRONYMS

ABBREVIATION/ ACRONYM	DEFINITION
Ab	Antibody
AE	Adverse Event
$Al(OH)_3$	Aluminum Hydroxide Adjuvant
CCHMC	Cincinnati Children’s Hospital Medical Center
CDC	(U.S.) Centers for Disease Control
CFR	Code of Federal Regulations
cm	Centimeter
CRF/eCRF	Case Report Form/electronic Case Report Form
CSR	Clinical Study Report
CRO	Contract Research Organization
dL	Deciliter
DSMB	Data Safety Monitoring Board
EC	Ethics Committee
ECG	Electrocardiogram
EDC	Electronic Data Capture
ELISA	Enzyme-linked Immunosorbent Assay
EPI	Expanded Program on Immunization
FAM-CRU	Family Clinical Research Unit, Tygerberg Hospital
FDA	(U.S.) Food and Drug Administration
g	Gram
GCP	Good Clinical Practice
GCLP	Good Clinical Laboratory Practice
GLP	Good Laboratory Practice
GMT	Geometric Mean Titer
IB	Investigators’ Brochure
ICH	International Conference on Harmonisation
ICF	Informed Consent Form
IND	Investigational New Drug
kg	Kilogram
L	Liter
MCC	Medicines Control Council, South Africa
MedDRA	Medical Dictionary for Regulatory Activities
MedDRA PT	Medical Dictionary for Regulatory Activities Preferred Term
MedDRA SOC	Medical Dictionary for Regulatory Activities System Organ Class
mEq	Milliequivalent
mg	Milligram
mL	Milliliter
µg	Microgram

ABBREVIATION/ ACRONYM	DEFINITION
NIAID	(U.S.) National Institute for Allergy and Infectious Diseases
NICD	National Institute for Communicable Diseases
NIH	(U.S.) National Institutes of Health
NS	Normal Saline
OHRP	(U.S.) Office of Human Research Protections
PBMCs	Peripheral Blood Mononuclear Cells
PE	Physical Examination
PI	Principal Investigator
PVS	PATH Vaccine Solutions
SAE	Serious Adverse Event
SDMC	Statistical Data Management Center
SDV	Source Data Verification
SRC	Safety Review Committee
SOP	Standard Operating Procedure(s)
TV	Trivalent
ULN	Upper Limit of Normal
VS	Vital Signs
WRAIR	Walter Reed Army Institute of Research
WBC	White Blood Cell
WHO	World Health Organization
WIRB	Western Institutional Review Board

VAC 041

PROTOCOL SIGNATURE PAGE

PATH Vaccine Solutions, the study Sponsor, will keep a list of Investigator(s), who must provide a *Curriculum Vita* (CV) and a copy of their medical licenses to the Sponsor or Sponsor representatives. The Sponsor will keep a list and qualification records of all relevant Sponsor study personnel.

The Emmes Corporation will provide medical monitoring, data management, statistical analysis, quality assurance and safety and pharmacovigilance services. Site monitoring will be performed through SCT Consulting, a contract research organization (CRO) experienced in clinical site monitoring.

**Site Contact**	**Phone Number**	**Email Address**
Respiratory and Meningococcal Pathogens Research Unit (RMPRU) PI		
Michelle Groome, MBBCh, DCH, MSc Med	+27 011 983 4283	groomem@rmpru.co.za
Shandukani Research Centre PI		
Lee Fairlie, MD	+27 11 358 5317	lfairlie@wrhi.ac.za
Family Clinical Research Unit (FAM-CRU)		
Julie Morrison, MBCHB, MRCPCH, DCH, FCPaed	+27 21 938-4153	morrison@sun.ac.za

SIGNATURE OF SITE PI:

“I have read the foregoing protocol and agree to conduct the study as outlined herein.”

-- -----------------------
Principal Investigator DD/MM/YY

VAC 041
PARTICIPATING INSTITUTIONS

**Sponsor**	PATH Vaccine Solutions<br>2201 Westlake Avenue<br>Suite 200<br>Seattle, WA 98121<br>USA
**Clinical Trial Sites**	Family Clinical Research Unit (FAM-CRU)<br>Site 8950<br>Faculty of Medicine Health Sciences,<br>Stellenbosch University<br>Ward J8, Tygerberg Academic Hospital,<br>Francie Van Zijl Drive, Parow Valley, 7505<br>South Africa<br>Telephone: +27 21 938 4302<br><br>Respiratory and Meningeal Pathogens Research Unit (RMPRU)<br>West wing, Nurses Residence<br>Chris Hani Baragwanath Academic Hospital<br>Soweto<br>South Africa<br><br>Shandukani Research Centre<br>Hillbrow Health Precinct<br>22 Esselen Street<br>Hillbrow<br>Johannesburg, 2001<br>South Africa<br>Telephone: 011 358 5919
**Research Laboratories**	National Institute for Communicable Diseases<br>Centre for Enteric Diseases<br>1 Modderfontein Rd.<br>Sandringham<br>Johannesburg, South Africa<br><br>Cincinnati Children's Hospital Medical Center<br>Laboratory for Specialized Clinical Studies<br>Division of Infectious Diseases<br>333 Burnet Avenue ML6014<br>Cincinnati, OH 45229-3039<br>USA
**Site Monitoring**	SCT Consulting<br>15 Bryanston Homestead Ben Road<br>Bryanston, 2021, South Africa<br>Mailing address: Postnet Suite 376, P/Bag X51,<br>Bryanston, Gauteng 2021, South Africa<br>Telephone: +27 82 654 8395

**Medical Monitoring**	The Emmes Corporation<br>401 N. Washington St., Suite 700<br>Rockville, MD 20850<br>USA
**Data Center and Statistical Support**	The Emmes Corporation<br>401 N. Washington St., Suite 700<br>Rockville, MD 20850<br>USA
**Vaccine Developer**	PATH Vaccine Solutions<br>2201 Westlake Avenue<br>Suite 200<br>Seattle, WA 98121<br>USA
**Vaccine Manufacturer**	Walter Reed Army Institute of Research (WRAIR)<br>Pilot BioProduction Facility<br>501 Robert Grant Avenue<br>Silver Spring, MD 20910

STUDY SYNOPSIS

**TITLE**	A Phase I/II double-blind, randomized, placebo-controlled, descending-age, dose-escalation study to examine the safety, tolerability and immunogenicity of the trivalent P2-VP8 subunit rotavirus vaccine in healthy South African adults, toddlers and infants
**STUDY NUMBER**	VAC 041
**PROJECT PHASE**	Phase 1/2
**SPONSOR**	PATH Vaccine Solutions (PVS)<br>2201 Westlake Avenue, Suite 200<br>Seattle, WA 98121
**MANUFACTURER**	Walter Reed Army Institute of Research (WRAIR)<br>Pilot BioProduction Facility<br>501 Robert Grant Avenue<br>Silver Spring, MD 20910
**NATIONAL PRINCIPAL INVESTIGATOR**	Michelle Groome, MBBCh, DCH, MSc Med<br>Respiratory and Meningeal Pathogens Research Unit (RMPRU)<br>West wing, Nurses Residence<br>Chris Hani Baragwanath Hospital<br>Soweto<br>South Africa
**SITE PRINCIPAL INVESTIGATORS**	Julie Morrison, MBCHB, MRCPCH, DCH, FCPaed<br>Family Clinical Research Unit (FAM-CRU)<br>Site 8950<br>Faculty of Medicine Health Sciences, Stellenbosch University<br>Ward J8, Tygerberg Academic Hospital,<br>Francie Van Zijl Drive, Parow Valley, 7505<br>South Africa<br>Telephone: +27 21 938 4153<br><br>Lee Fairlie, MD<br>Shandukani Research Centre<br>Hillbrow Health Precinct<br>22 Esselen Street<br>Hillbrow<br>Johannesburg, 2001<br>South Africa<br>Tel: 011 358 5919<br><br>Michelle Groome, MBBCh, DCH, MSc Med<br>Respiratory and Meningeal Pathogens Research Unit (RMPRU)<br>West wing, Nurses Residence<br>Chris Hani Baragwanath Hospital<br>Soweto<br>South Africa
**CRO MEDICAL MONITOR**	Robert Lindblad, M.D.<br>The Emmes Corporation<br>401 N. Washington St., Suite 700<br>Rockville, MD 20850

**PATH MEDICAL OFFICER**	Alan Fix, MD, MS<br>455 Massachusetts Avenue, Suite 1000<br>Washington, DC 20001
**PATH CLINICAL OPERATIONS SPECIALIST**	Maureen Power, RN, MPH<br>455 Massachusetts Avenue, Suite 1000<br>Washington, DC 20001
**PATH CONTACT REGARDING INVESTIGATIONAL PRODUCT**	Stanley Cryz, MD, PhD<br>455 Massachusetts Avenue, Suite 1000<br>Washington, DC 20001
**CONTRACT RESEARCH ORGANIZATIONS**	**Site Monitoring:**<br>SCT Consulting<br>15 Bryanston Homestead Ben Road<br>Bryanston, 2021, South Africa<br>Mailing address: Postnet Suite 376, P/Bag X51, Bryanston, Gauteng 2021, South Africa<br>Phone: 27 82 654 8395<br><br>**Safety Monitoring & Data Management:**<br>The Emmes Corporation<br>401 N. Washington St., Suite 700<br>Rockville, MD 20850
**LABORATORIES**	Research Laboratories:<br>• Nicola Page, PhD<br>Head: Virology Division<br>National Institute for Communicable Disease<br>1 Modderfontein Rd.<br>Sandringham<br>Johannesburg, South Africa<br><br>• Monica McNeal, MS<br>Cincinnati Children's Hospital Medical Center<br>Laboratory for Specialized Clinical Studies<br>Division of Infectious Diseases<br>3333 Burnet Avenue ML6014<br>Cincinnati, OH 45229-3039<br><br>Clinical Laboratory:<br>• Clinical Laboratory Services,<br>Spencer Lister Building, NHLS Central Complex<br>Cnr Hospital and De Korte Street<br>Braamfontein, Johannesburg<br>South Africa<br><br>• Bio Analytical Research Corporation (BARC)<br>Waverley Business Park, Unit 7<br>Bldg 23B, Wycroft Road<br>Mowbray, 7700<br>South Africa

**INVESTIGATIONAL PHARMACIES**	Respiratory and Meningeal Pathogens Research Unit (RMPRU)<br>West wing, Nurses Residence<br>Chris Hani Baragwanath Hospital<br>Soweto, South Africa<br><br>Family Clinical Research Unit (FAM-CRU)<br>Tygerberg Hospital<br>Francie Van Zijl Drive<br>Parow Valley 7505<br>Tygerberg, South Africa<br><br>Shandukani Research Pharmacy<br>2nd Floor Shandukani Building<br>Hillbrow Health Precinct<br>C/O Esselen and Klein Street<br>Hillbrow<br>Gauteng, South Africa
**STUDY HYPOTHESES - PRIMARY**	**Safety**<br>The trivalent P2-VP8 subunit rotavirus vaccine is safe and well-tolerated in healthy South African adults, toddlers and infants.<br><br>**Immunogenicity**<br>The trivalent P2-VP8 subunit rotavirus vaccine is immunogenic in healthy South African infants and will induce neutralizing immune responses to at least 2 of the 3 strains from which vaccine antigens are derived in 60% or more of participants in at least one of the study groups.
**STUDY OBJECTIVES**	**Primary Objectives:**<br><br>**Safety**<br>• To evaluate the safety and tolerability of the trivalent P2-VP8 subunit rotavirus vaccine at escalating dose levels in healthy South African adults, toddlers and infants<br>**Immunogenicity**<br>• To evaluate the immunogenicity of three doses of the trivalent P2-VP8 subunit rotavirus vaccine at different dose levels in healthy South African infants<br><br>**Secondary Objectives:**<br><br>**Safety**<br>• To evaluate the longer term safety (through 6 months after the last vaccination) of the trivalent P2-VP8 subunit rotavirus vaccine at escalating dose levels in healthy South African adults, toddlers and infants

**STUDY OBJECTIVES (cont.)**	**Immunogenicity**<br>• To evaluate the immunogenicity of two doses of the trivalent P2-VP8 subunit rotavirus vaccine at different dose levels in healthy South African infants<br>• To evaluate the immunogenicity of the trivalent P2-VP8 subunit rotavirus vaccine at different dose levels in healthy South African adults and toddlers<br><br>**Exploratory Objective:**<br><br>**Efficacy**<br>• To evaluate the impact of the trivalent P2-VP8 subunit rotavirus vaccination on shedding of Rotarix subsequently administered in healthy South African infants as a test of concept
**STUDY ENDPOINTS**	**Primary Endpoints:**<br>**Safety (all 3 age-groups)**<br>• Number of SAEs through 28 days after the last study injection<br>• Number of AEs through 28 days after the last study injection<br>• Number of vaccine-induced local and systemic reactions<br><br>**Immunogenicity (infants):**<br>• Proportion of infants with anti-P2-VP8 IgG & IgA seroresponses by ELISA (four-fold increase in antibody titers between baseline and 4-weeks post-third study injection) in assays using each of the three vaccine antigens (P[4], P[6] and P[8])<br>• Proportion of infants with neutralizing antibody responses (2.7-fold increase in antibody titers between baseline and 4-weeks post-third study injection) to each of the three rotavirus strains from which the vaccine antigens are derived, as well as heterologous strains<br>• Anti-P2-VP8 IgG & IgA geometric mean titers (GMT) (baseline and 4 weeks third study injection) in ELISA assays using each of the three vaccine antigens (P[4], P[6] and P[8])<br>• Neutralizing antibody GMT (baseline and 4 weeks post-third study injection) to each of the three rotavirus strains from which the vaccine antigens are derived, as well as heterologous strains<br>• Proportion of infants with neutralizing antibody responses to at least two of the three strains from which the vaccine antigens are derived

**STUDY ENDPOINTS (cont.)**	**Secondary Endpoints:**<br><br>**Safety (all 3 age-groups)**<br>• Number of SAEs at any time during the study<br>• Number of AEs at any time during the study<br><br>**Immunogenicity (infants):**<br>• Proportion of infants with anti-P2-VP8 IgG & IgA seroresponses by ELISA (four-fold increase in antibody titers between baseline and 4 weeks post-second study injection) in assays using each of the three vaccine antigens (P[4], P[6] and P[8])<br>• Proportion of infants with neutralizing antibody responses (2.7-fold increase in antibody titers between baseline and 4 weeks post-second study injection) to each of the three rotavirus strains from which the vaccine antigens are derived, as well as heterologous strains<br>• Anti-P2-VP8 IgG & IgA geometric mean titers (GMT) (baseline and 4 weeks post-second study injection) in ELISA assays using each of the three vaccine antigens (P[4], P[6] and P[8])<br>• Neutralizing antibody GMT (baseline and 4 weeks post-second study injection) to each of the three rotavirus strains from which the vaccine antigens are derived, as well as heterologous strains<br>• Proportion of infants with neutralizing antibody responses to at least two of the three strains from which the vaccine antigens are derived (4 weeks post-second study injection)<br><br>**Immunogenicity (adults and toddlers)**<br>• Proportion of adults and toddlers with anti-P2-VP8 IgG & IgA seroresponses by ELISA (four-fold increase in antibody titers between baseline and 4-weeks post-final study injection) in assays using each of the three vaccine antigens (P[4], P[6] and P[8])<br>• Proportion of adults and toddlers with neutralizing antibody responses (2.7-fold increases in antibody titers between baseline and 4-weeks post-final study injection) to each of the three rotavirus strains from which the vaccine antigens are derived, as well as heterologous strains<br>• Anti-P2-VP8 IgG & IgA geometric mean titers (GMT) (baseline and 4 weeks post final study injection) in ELISA assays using each of the three vaccine antigens (P[4], P[6] and P[8])<br>• Neutralizing antibody GMT (baseline and 4 weeks post-final study injection) to each of the three rotavirus strains from which the vaccine antigens are derived, as well as heterologous strains<br>• Proportion of adults and toddlers with neutralizing antibody responses to at least two of the three strains from which the vaccine antigens are derived<br><br>**Exploratory Endpoint**<br>• Proportion of participants shedding Rotarix after administration of Rotarix "challenge"

**STUDY RATIONALE**	Live oral rotavirus vaccines have not performed optimally in developing-country populations, similar to the discrepancy in performance observed between developed versus developing countries for other oral, live attenuated enteric vaccines such as those targeted against cholera and poliomyelitis. Several direct and indirect observations support the possibility that parenterally-administered non-replicating rotavirus vaccines may be successful, including observations derived from natural rotavirus infection studies in infants, rotavirus challenge studies in adult volunteers, human rotavirus vaccine trials, and animal rotavirus vaccine studies. The P2-VP8 vaccine contains a truncated rotavirus VP8 subunit fused to the tetanus toxin P2 CD4 epitope. It is parenterally administered, which may overcome limitations of oral vaccines in developing countries. A monovalent formulation containing the VP8 subunit from a P[8] rotavirus strain has been demonstrated to be well-tolerated and immunogenic in US adults (VAC 009) and is currently being tested in toddlers and infants in South Africa (VAC 013), where it has been generally well-tolerated. In the study in adults, the monovalent vaccine elicited robust neutralizing antibody responses to several homologous P[8] strains of rotavirus, but modest response to P[4] strains and meager responses to P[6] strains. Thus, the trivalent formulation to be tested in this study, containing VP8 subunits from P[4], P[6] and P[8] strains was developed, with the goal to optimize responses to those strains that account for the vast majority of the burden of rotavirus disease globally. Preclinical studies of the trivalent vaccine indicate improved responses to P[4] and P[6] strains. Preliminary analysis of serologic responses and impact of the monovalent P2-VP8 vaccine on shedding of Rotarix, as a proxy model for efficacy, in infants in VAC 013 has provided promising results. Those results have also provided the basis for the selection of the dose levels of the trivalent vaccine to be tested in this protocol.
**INVESTIGATIONAL PRODUCTS**	**Vaccine:** The TV P2-VP8 subunit rotavirus vaccine produced in *E. coli* is adsorbed onto aluminum hydroxide (0.5625 mg/dose). Three dose levels of vaccine will be tested: 15 µg, 30 µg and 90 µg (consisting of equal amounts of VP8 antigen derived from a P[4], a P[6] and a P[8] strain of rotavirus), all to be administered in 0.5 mL volumes intramuscularly. The three antigens are derived from DS1, 1076 and Wa strains, respectively.<br><br>**Placebo:** Normal Saline (NS)
**STUDY POPULATION**	• 30 healthy South African adults (18-45 years old, inclusive, at time of first vaccination)<br>• 30 healthy South African toddlers (≥2 & < 3 years old at time of first vaccination)<br>• 600 healthy South African infants (≥6 & <8 weeks old at the time of first vaccination)
**STUDY DURATION**	Adult and infant participants will be followed for approximately 8 months after randomization (6 months following last [3rd] injection). Toddler participants will be followed for approximately 6 months after randomization (6 months following the single injection).

**STUDY DESIGN**	The trial will be a double-blind, randomized, placebo-controlled, descending-age, dose-escalation study in which two dose-levels of vaccine will be tested in South African adults and toddlers, and three dose-levels will be tested in infants, with progression from one dose-level to the next and one age-group to the next based on assessment of safety and tolerability.<br><br>The adult cohorts (Cohort A1 and A2) will consist of 15 participants (12 vaccine recipients and 3 placebo recipients) per dose level, who will receive three intramuscular injections four weeks apart. The two dose levels of vaccine to be tested will be 30 µg (consisting of 10 µg each of the P[4], P[6] and P[8] P2-VP8 subunit proteins) and 90 µg (consisting of 30 µg each of the P[4], P[6] and P[8] P2-VP8 subunit proteins). Progression from low- to high-dose in adults, and to low-dose in toddlers, will be based on assessment of safety and tolerability during the week after the first injection in the adult low-dose cohort. The adult cohorts will receive a series of 3 study injections to allow optimal comparison with the results of the VAC 009 study, in which US adults received a series of 3 study injections of the monovalent vaccine (containing P[8] only, up to 60 µg).<br><br>Similarly, the toddler cohorts (Cohorts B1 and B2) will consist of 15 participants (12 vaccine recipients and 3 placebo recipients) per dose level. Participants will receive a single intramuscular injection. If found to be safe in the adult cohorts, the two dose levels of vaccine to be tested will be 30 µg (consisting of 10 µg each of the P[4], P[6] and P[8] P2-VP8 subunit proteins) and 90 µg (consisting of 30 µg each of the P[4], P[6] and P[8] P2-VP8 subunit proteins). Initiation of the toddler low-dose cohort will be based on assessment of safety and tolerability of that dose during the week after the first injection in the adult low-dose cohort. Progression from low- to high-dose in the toddlers, and to low-dose in infants, will be based on assessment of safety and tolerability during the week after the first injection in the toddler low-dose cohort. Further, progression to the high-dose in toddlers will also be based on assessment of safety and tolerability during the week after first injection in the adult high-dose cohort. The toddler cohorts will receive only one study injection, as was the case for toddlers in VAC 013, as it is the safety data following this single dose that will be assessed to determine whether to proceed to the infant cohorts.<br><br>Initial assessment in infants will consist of 16 participants (12 vaccine recipients and 4 placebo recipients) per dose level (Cohorts C1, C2 and C3). Participants will receive three intramuscular injections four weeks apart. If found to be safe in the toddler cohorts, the three dose levels of vaccine to be tested will be 15 µg (consisting of 5 µg each of the P[4], P[6] and P[8] P2-VP8 subunit proteins), 30 µg (consisting of 10 µg each of the P[4], P[6] and P[8] P2-VP8 subunit proteins) and 90 µg (consisting of 30 µg each of the P[4], P[6] and P[8] P2-VP8 subunit proteins). Initiation of the infant low-dose (15 µg) cohort will be based on assessment of safety and tolerability during

	the week after first injection in the toddler low-dose (30 µg) cohort. Progression from the 15 µg to the 30 µg dose cohort in the infants will be based on assessment of safety and tolerability during the week after the first injection both in the infant 15 µg -dose cohort and the toddler 30 µg dose cohort. Progression from the 30 µg to the 90 µg dose cohort in the infants will be based on assessment of safety and tolerability during the week after the first injection in the infant 30 µg dose cohort.<br><br>Should all three doses be tolerated in the initial assessment in infants, testing in infants will be expanded (Group D) to obtain both (a) greater safety experience and (b) more robust immunogenicity data for comparison of the three doses to support planning of subsequent, advanced phase testing. Group D will consist of 4 groups of 138 participants each: 15 µg, 30 µg and 90 µg of vaccine and placebo.<br><br>All infants will receive 3 doses of Rotarix, at monthly intervals, starting a month after the final study injection (on study Day 84, at the time the post-vaccination blood sample is obtained), and fecal shedding of Rotarix will be assessed during the week after the first dose on a subset of infants.

STUDY SCHEMA (see Appendix I for full schedule of events)

Group		TV P2-VP8 Dose	N	-7[a] to -1	0	7	28	56	84	168	224
A Adult	A1	30 µg	12	B(S,I)	X	B(S)	X, B(I)	X, B(I)	B(I)		F
		Placebo	3	B(S,I)	P	B(S)	P, B(I)	P, B(I)	B(I)		F
	A2	90 µg	12	B(S,I)	X	B(S)	X, B(I)	X, B(I)	B(I)		F
		Placebo	3	B(S,I)	P	B(S)	P, B(I)	P, B(I)	B(I)		F
**A Total**			**30**								
B Toddler	B1	30 µg	12	B(S,I)	X	B(S)	B(I)			F	
		Placebo	3	B(S,I)	P	B(S)	B(I)			F	
	B2	90 µg	12	B(S,I)	X	B(S)	B(I)			F	
		Placebo	3	B(S,I)	P	B(S)	B(I)			F	
**B Total**			**30**								
C Infant	C1	15 µg	12	B(S,I)	X	B(S)	X	X, B(I)	B(I)		F
		Placebo	4	B(S,I)	P	B(S)	P	P, B(I)	B(I)		F
	C2	30 µg	12	B(S,I)	X	B(S)	X	X, B(I)	B(I)		F
		Placebo	4	B(S,I)	P	B(S)	P	P, B(I)	B(I)		F
	C3	90 µg	12	B(S,I)	X	B(S)	X	X, B(I)	B(I)		F
		Placebo	4	B(S,I)	P	B(S)	P	P, B(I)	B(I)		F
**C Total**			**48**								
D Infant		15 µg	138	B(S,I)	X		X	X, B(I)	B(I)		F
		30 µg	138	B(S,I)	X		X	X, B(I)	B(I)		F
		90 µg	138	B(S,I)	X		X	X, B(I)	B(I)		F
		Placebo	138	B(S,I)	P		P	P, B(I)	B(I)		F
**D Total**			**552**								
**All Cohorts Total**			**660**								

X = TV P2-VP8 vaccine

P = placebo

B = blood sample for (S) safety and/or (I) immunogenicity testing

F = final study f/u visit/call

[a]For adult and toddler cohorts, -28 to -1

For each age group, progression from one dose level to the next will require review of clinical and safety lab data through 7 days after the first dose at the lower dose level. Safety laboratory blood specimens will be drawn at baseline for all participants (screening) and 7 days after first study injection in Groups A, B and C.

Blood samples for immunogenicity analysis will be obtained at baseline for all participants, Days 28, 56 and 84 for Group A (adults), Day 28 for Group B (toddlers), and Days 56 and 84 for Groups C and D (infants).

For each dose level, progression from adults in Group A to toddlers in Group B and then to infants in Group C will require review of clinical and safety laboratory data through 7 days after study injection in the older group. (see Figure 1)

These determinations will be made by the Safety Review Committee (SRC).

**STUDY PROCEDURES & VISIT SCHEDULE**	Participants at each dose level in Group A will receive three intramuscular injections in the deltoid of P2-VP8+ $Al(OH)_3$ or placebo at Days 0, 28 and 56.<br>Participants at each dose level in Group B will receive a single intramuscular injections in the anterolateral thigh of P2-VP8+ $Al(OH)_3$ or Placebo at Day 0.<br>Participants at each dose level in Groups C and D will receive three intramuscular injections in the anterolateral thigh of P2-VP8+ $Al(OH)_3$ or placebo at Days 0, 28 and 56. EPI vaccines will be co-administered with the three study vaccines in all infants (at 6, 10 and 14 weeks of age), with the exception of rotavirus. All infants will receive 3 doses of Rotarix, at monthly intervals, starting at approximately 28 days after the final study injection (on study Day 84, at the time the post-vaccination blood sample is obtained).<br>Safety will be assessed according to the following schedule:<br>1. Blood draw obtained for eligibility screening and as baseline measure for all participants<br>2. Blood draw for safety laboratory assessment 7 days after the first study injection in Groups A, B and C<br>3. Clinic visit for reactogenicity and safety assessment (including monitoring safety laboratory tests) 7 days following each injection for all participants<br>4. Final clinic visit for safety assessment 6 months following last injection for all cohorts<br>In addition to the blood draws above, adults in Group A will have blood drawn for assessment of immune response assessment at baseline and one month following each vaccination (Days 28, 56 and 84), toddlers in Group B will have blood drawn for immune response assessment at baseline and one month following vaccination (Day 28), and infants in Groups C and D will have blood drawn for assessment of immune response at baseline and at one month after the second and third study injection (Days 56 and 84). When possible, stool samples will be obtained from infants experiencing diarrhea to test for rotavirus.<br>Infants will receive Rotarix vaccination 28, 56 and 84 days following completion of the 3 study injections (Study Days 84, 112 and 140). Stool samples will be obtained to assess Rotarix shedding 5, 7, and 9 days following the first dose of Rotarix (Study Days 89, 91, and 93) on a subset of infants.
**SAFETY MONITORING**	For progression from lower to higher dose-level and to successively lower age-groups, a Safety Review Committee (SRC) will evaluate clinical and laboratory safety data through Day 7 after the first injection for all participants before recommending whether to proceed.<br>Throughout the study, the site PIs will provide continuous monitoring for safety, and the SRC will review safety data regularly. A DSMB will oversee the study, with scheduled periodic meetings, and, as needed, ad hoc meetings.

**IMMUNOLOGY ASSESSMENTS**	• Anti-P2-VP8 IgG & IgA by ELISA (assessed separately with antigens from P[4], [6] and [8] strains)<br>• Homologous and heterologous rotavirus neutralization using strains expressing the P[4], P[6] and P[8] serotypes<br>• Anti-rotavirus IgA (to whole viral lysate) by ELISA
**STATISTICAL CONSIDERATIONS**	It is assumed that almost all participants enrolled will provide data for safety analysis and at least 90% of enrolled participants will be evaluable for immunogenicity assessments.<br><br><u>Safety</u><br><br>For Cohorts A and B, 12 vaccine recipients per dose group and 24 vaccine recipients for the two dose groups combined will provide a greater than 90% chance of observing an AE that has a 17.5% and 9.2% rate of occurrence, respectively. The two infant cohorts combined (C and D), with 150 vaccine recipients per dose group and 450 vaccine recipients for the three dose groups combined, will provide a greater than 90% chance of observing an AE that has a 1.6% and 0.5% rate of occurrence, respectively.<br><br>Conversely, if no SAEs are observed in 24 and 450 vaccine recipients, the study will be able to rule out SAEs occurring at a rate of approximately 11.7% and 0.7%, respectively based on the upper bounds of the one-sided 95% confidence interval.<br><br><u>Immunogenicity (Infant Cohorts)</u><br><br>Based on the results in South African infants who received monovalent P2-VP8 vaccine or placebo (VAC-013), the strain specific seroresponse rates are expected to be ≥80% for at least one of the three P2-VP8 vaccine doses and <20% for the placebo group. For the two infant cohorts combined with 135 evaluable vaccine recipients per dose level (assuming 10% loss), this study is designed to provide at least 74% and 95% power (Fisher's exact test) to detect 15 and 20 percentage points difference (e.g. 65% vs. 80% and 60% vs. 80%), respectively, in seroresponse rates between any two P2-VP8 dose groups. For comparisons between a P2-VP8 dose group and the combined placebo groups, this study is designed to provide ≥99% power to detect ≥30 percentage point difference (e.g. 50% vs. 20%).<br><br>For the sample size calculations of the Geometric Mean Titer (GMT) endpoint based on the immunogenicity results in VAC 013, the LOG10 standard deviations were estimated to be < 0.6 (range: 0.24 – 0.56) for the P2-VP8 doses and the placebo group. Using a conservative LOG10 standard deviation of 0.6, this study with 135 evaluable infants per group (assuming 10% loss) is designed to provide at least 98% power to detect as low as a 2.0-fold difference between any two P2-VP8 dose groups or between any P2-VP8 vaccine dose (15 µg, 30 µg or 90 µg dose) and the placebo group.

Figure 1: Study overview and timeline

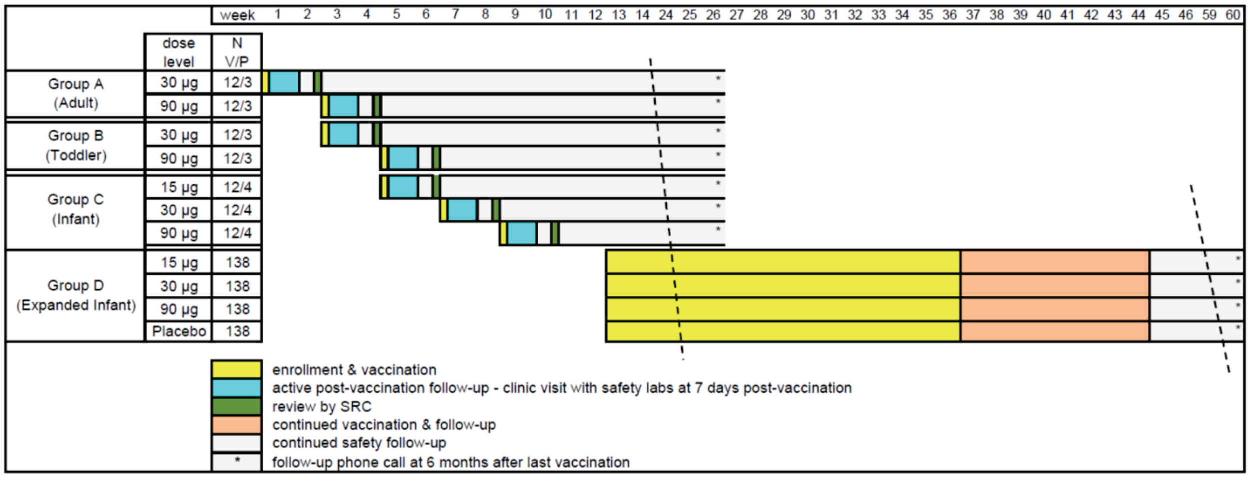

Note: Time line does not take into account delay of enrollment of infant groups until after peak of rotavirus season.

1. BACKGROUND AND INTRODUCTION

1.1. Burden of Disease

Diarrhea is the second-leading cause of death worldwide among children under the age of five, killing an estimated 1.3 million annually and hospitalizing millions more, mostly in developing countries [1]. The most common cause of severe infantile diarrhea is rotavirus; almost all children are infected with rotavirus before their third birthday regardless of geographic location or economic status. Global surveillance indicates that rotavirus is associated with up to 40 percent of severe, dehydrating diarrheal episodes in young children, resulting in hospitalization and more than 450,000 deaths each year. More than 85 percent of these deaths occur in the world's poorest countries [1]. Children in low-income countries are at greater risk of severe rotavirus disease because they are more likely to be infected earlier in life, access to urgent medical care can be limited or unavailable, and there is a greater prevalence of malnutrition and other co-morbidities. In addition to causing loss of life, rotavirus places a burden on health-care systems and families. Vaccination offers the best hope for protecting all infants, regardless of economic status, against rotavirus disease [2].

1.2. Pathogen

Rotavirus infection is primarily acquired through the fecal oral route. Universal infection in both developing and developed countries indicate that improved sanitation alone will not control rotavirus infection [3]. Treatment is symptomatic, with an emphasis on maintaining hydration either by oral administration of fluids or, in severe disease, via the intravenous route, with a favorable prognosis [4]. Unfortunately, provision of such rudimentary supportive care in many impoverished areas of the world is limited, contributing to the high mortality rate observed.

The vast majority of human disease (~90%) is caused by group A rotavirus. Two surface proteins, VP4 and VP7, are able to induce neutralizing antibody during infection, and both are very divergent across different rotavirus strains: the VP7 glycoprotein represents the G serotype, while the VP4 protein represents the P serotype. The seroepidemiology of rotavirus strains causing human disease has been extensively studied [5]. The findings have potentially important implications for vaccine development. While geographical differences have been noted in the absolute frequency of the various P serotypes, the P[4] and P[8] serotypes predominate (Figure 2, below), accounting for between 90-99% of strains isolated from Asia, North America, Europe, South America and Australia/Oceania. However, in Africa they represent approximately 70% of clinical isolates with P[6] accounting for nearly all of the remaining strains. There is no clear evidence that protection against rotavirus infection is exclusively homotypic, but if that were the case, a bivalent vaccine that contains P[4] and P[8] would provide 90% coverage in all areas except Africa. For Africa, a P[6] component would be required for a similar rate of coverage. However, more recent data indicate the prevalence of P[6] currently may be about half of previous estimates [6, 7]. In contrast, the diversity among G serotypes is greater, with 4-5 serotypes representing ~90% of clinical isolates worldwide.

Like the influenza hemagglutinin, the rotavirus VP4 must be cleaved by proteases before the virus can be activated [8]. Such cleavage results in the generation of two proteins, VP5 and VP8, which remain attached to the virion. Most of the antigenic epitopes in VP4 that induce neutralizing activity have been mapped to VP8, including those responsible for the serotype differences among different strains.

Figure 2: Continental Variation in the Distribution of Human Group A Rotavirus P Types Ascertained by Analysis of Strains Collected between 1982-2003
[Santos 2005, Rev Med Virol (5)]

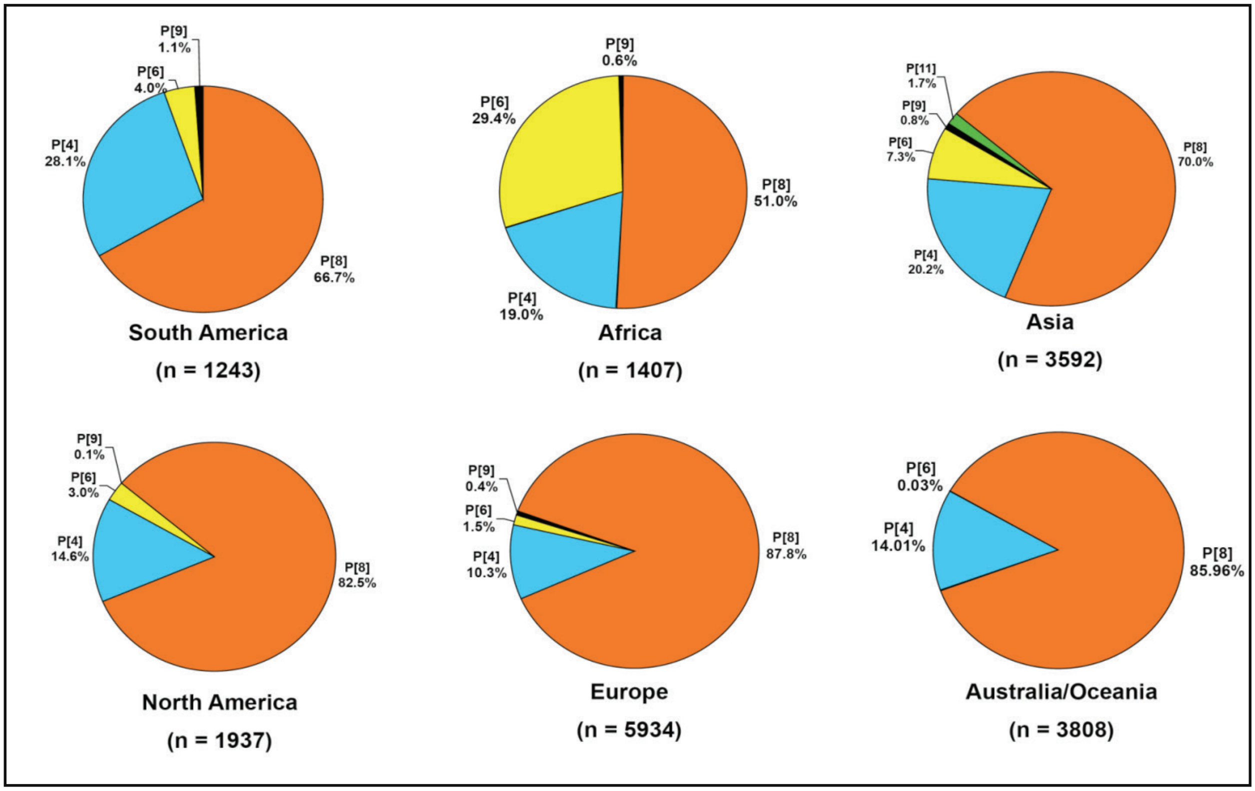

1.3. Non-replicating Rotavirus Vaccine Approach Using VP8 Subunit

The VP8 subunit of VP4 protein has been expressed in various systems and demonstrated to induce rotavirus-specific neutralizing antibodies and/or protection in various animal models [9-18]. Monoclonal antibodies to VP8 administered orally are able to confer passive protection in mice [19] and natural rotavirus infection, as well as vaccination with live vaccines, induces protective anti-VP8 neutralizing antibodies [16]. Mapping of neutralizing monoclonal antibodies against VP8 reveals that they recognize linear epitopes in the region between amino acids 64 and 223 of VP8 [13, 20]. All epitopes that induce neutralizing antibody responses to VP8 serotypes P[4], P[6] and P[8] have been mapped to that region [14, 21]), which was selected for inclusion in the proposed P2-VP8 subunit rotavirus vaccine. Recombinant VP8 protein vaccine candidates have been produced in several systems, including *E. coli* [13, 18, 22, 23]), lactobacillus [15], yeast [24] and plants [25]. A norovirus particle recombinant fusion protein utilizing a truncated VP8 sequence has also been developed and shown to confer protection against rotavirus in a murine challenge model [18]. VP8 produced in *E. coli* induces high-titered homotypic neutralizing antibodies in mice, cattle, rabbits, chickens and guinea pigs [12, 13, 23]), as well heterotypic neutralizing antibodies [23]. Passively transmitted antibodies derived from VP8 immunization protected offspring of vaccinated dams [12, 25, 26]. The magnitude and breadth of the heterotypic neutralizing antibodies induced has been variable and strain dependent. To improve the vaccine potential of the recombinant VP8 subunit protein, we synthesized a fusion protein comprised of the P2 universal $CD4^+$ T cell epitope (aa 830-844 of tetanus toxin) and a truncated VP8 moiety, which contains all known neutralizing epitopes. The P2 epitope has been employed in the development of various viral and bacterial experimental vaccines including malaria, *H. influenza* type b and others [27, 28].

The P2-VP8 vaccine to be tested in this study was developed by Dr. Taka Hoshino at NIAID [29]. The monovalent (P[8]) vaccine previously tested consists of bacteria-expressed VP8 subunit from the Wa strain of human rotaviruses (G1[P8]). A DNA segment encoding the sequence of P2 epitope from tetanus toxin was fused to the VP8 sequence, resulting in this chimeric protein vaccine (P2-VP8). Dr. Hoshino's laboratory has demonstrated in preclinical testing that the immunogenicity of these VP8 proteins could be significantly enhanced when fused with the P2 epitope of tetanus toxin, which exerts a strong T cell helper function. Further, immunization of neonatal piglets with a P2-VP8-P[8] chimeric protein conferred significant protection against experimental rotavirus gastroenteritis [30]. Based on results of the initial first-in-human testing of the monovalent (P[8]) vaccine (see section 1.7), a trivalent vaccine that includes antigens from P[4], P[6] and P[8] strains (DS1, 1076 and Wa, respectively) has been developed to broaden responses for these 3 P-types, which together are responsible for the vast majority of global disease burden. The trivalent vaccine will be assessed in this study.

1.4. Potential Safety Risks

Based on pre-clinical studies, the most commonly expected adverse events are reactions at the injection site, including redness, swelling and pain; these findings are consistent with reactions seen with licensed vaccines. In addition, as with any vaccine, allergic reactions, including the extremely rare occurrence of life-threatening allergic reactions, cannot be excluded.

The vaccine is to be adsorbed on aluminum hydroxide [$Al(OH)_3$]. There is a wealth of experience demonstrating the safety of aluminum salts as an adjuvant, with potential local reactions of erythema, subcutaneous nodules and contact hypersensitivity [31].

Safety data from first-in-human phase 1 testing of the monovalent (P[8]) P2-VP8 vaccine in adults, toddlers and infants have been reassuring and consistent with anticipated predominantly mild local reactions. (see Section 1.7 for details).

1.5. Summary of Nonclinical Studies with Monovalent (P[8]) P2-VP8

The safety and immunogenicity of the P2-VP8 subunit vaccine was evaluated in three animal models (guinea pigs, Gnotobiotic piglets, and New Zealand White rabbits). An overview of these preclinical studies supporting the development of the P2-VP8 subunit vaccine is provided below in Table 1.

Table 1: Preclinical Studies Supporting P2-VP8 Subunit Vaccine Development

Study Title	Study Site	Study Type & Compliance	Animal Model	Adjuvant	Location of Summary
Evaluation of the Antibody Response in Guinea Pigs Administered VP8 or P2-VP8	National Institutes of Health, Bethesda, MD	Discovery Non-GLP	Guinea Pig	None	Section 1.5.1.1
Evaluation of the Antibody Response in Guinea Pigs Administered P2-VP8 with or without Aluminum Phosphate Adjuvant	National Institutes of Health, Bethesda, MD	Discovery Non-GLP	Guinea Pig	Aluminum Phosphate	Section 1.5.1.2
Evaluation of the Rotavirus Neutralizing Antibody Response in Guinea Pigs	Covance, Denver, PA	Immunogenicity Non-GLP	Guinea Pig	Aluminum Phosphate	Section 1.5.1.3
Protection from Rotavirus Infection in Neonatal Piglets	Virginia Tech, Blacksburg, VA	Immunogenicity Challenge<br>Non-GLP	Gnotobiotic Piglets	Aluminum Phosphate	Section 1.5.1.4
Repeat Dose Intramuscular Safety and Immunogenicity Study of P2-VP8 subunit vaccine (with and without Aluminum Hydroxide Adjuvant) in Rabbits	AVANZA Laboratories, Gaithersburg, MD	Immunogenicity Toxicology<br>GLP	New Zealand White Rabbits	Aluminum Hydroxide	Section 1.5.2

1.5.1. Non-GLP Nonclinical Pharmacology/Proof of Concept Studies

1.5.1.1. Evaluation of the Antibody Response in Guinea Pigs Administered Monovalent (P[8]) VP8 or P2-VP8

To improve the vaccine potential of the recombinant VP8 subunit protein, we synthesized a fusion protein comprised of the P2 universal CD4+ T-cell epitope (aa 830-844 of tetanus toxin) and a truncated VP8 moiety which contains all known VP8 neutralizing epitopes. Increased immunogenicity was assessed in guinea pigs inoculated with either VP8 alone or fused to the P2 epitope [Dr. T. Hoshino, National Institute of Allergy and Infectious Diseases (NIAID), National Institutes of Health (NIH), personal communication]. The sera were tested in a 60% plaque neutralization assay against the homologous strain, which showed that inclusion of a P2 CD4+ T-cell epitope sequence with VP8 enhances the immunogenicity of VP8 by engendering a more rapid rise in neutralizing antibody titers.

1.5.1.2. Evaluation of the Antibody Response in Guinea Pigs Administered Monovalent (P[8]) P2-VP8 with or without Aluminum Phosphate Adjuvant

Adsorption of monovalent (P[8]) P2-VP8 subunit onto aluminum phosphate increases overall titer and promotes an earlier neutralizing antibody titer compared to monovalent (P[8]) P2-VP8 subunit vaccine alone in guinea pigs. High neutralizing antibodies titers were observed for up to eight months in animals immunized with 10 or 20 µg monovalent (P[8]) P2-VP8 subunit vaccine adsorbed to aluminum phosphate.

Aluminum hydroxide [$Al(OH)_3$] was selected as the adjuvant for further preclinical and clinical development based on its success in other clinical trials conducted by PVS with other antigens, citations in literature that indicate aluminum hydroxide [$Al(OH)_3$] may have greater adjuvant activity than aluminum phosphate (27), and availability of clinical-grade material. Similarly, as noted in Section 1.5.2.3, rabbits immunized with aluminum hydroxide [$Al(OH)_3$] adjuvanted vaccine in the GLP toxicology study displayed higher anti-P2-VP8 IgG binding antibody levels than those immunized with non-adjuvanted vaccine.

1.5.1.3. Evaluation of the Rotavirus Neutralizing Antibody Response in Guinea Pigs

A non-GLP study was conducted in Hartley Guinea Pigs (Elm Hill) to evaluate the magnitude and breadth of the immune response following vaccination with research grade monovalent (P[8]) P2-VP8 subunit vaccine, adsorbed to aluminum adjuvant, at Covance (Denver, PA).

Five guinea pigs were immunized with 25 µg monovalent (P[8]) P2-VP8 subunit vaccine adsorbed to aluminum phosphate adjuvant by intramuscular (IM) injection, the planned route of human exposure, on Days 0, 30, and 60. Three doses were used to mimic the EPI schedule. Serum was collected for immunological analyses at baseline (Day -7), on Days 0, 30, and 60 (prior to dosing) and at termination on Day 74. Animals were observed daily for viability and general health.

With the exception of a single animal that was found with a broken leg that was subsequently euthanized, no clinical signs were noted throughout the study.

Rotavirus neutralizing antibodies, which can be directed against either the G (VP7) or P (VP4) antigen expressed by rotavirus, were quantified using a neutralization assay run in a blinded manner by an independent testing laboratory (M. McNeal, Cincinnati Children's Hospital Medical Center, Cincinnati, OH). To analyze the breadth of the immune response, sera were run against six human strains of rotavirus expressing various combinations of G (1/2/3/4 and 9) and P (4/6/8) genotypes, which have been found to predominate among human field isolates. The results are shown below in Table 2 and Table 3. Immunization with the monovalent (P[8]) P2-VP8 subunit vaccine elicited a vigorous antibody response to the Wa, P, and WI61 strains, all of which displayed the homologous P[8] genotype (Table 2). However, the post-immunization titers to the VA70 strain, which also displays the P[8] genotype were substantially lower. Responses were meager to the ST3 strain, which has a P[6] genotype; there were intermediate responses to the DS1 strain, which has a P[4] genotype. Overall, immunization resulted in all animals mounting a 4-fold or greater rise in titer over baseline values, regardless of the P genotype (Table 3).

Table 2: Geometric Mean Neutralizing Titers to Human Rotavirus Strains: Before and After Immunization

WA		P		WI61		ST3		VA70		DS-1	
(G1, P[8])		(G3, P[8])		(G9, P[8])		(G4, P[6])		(G4, P[8])		(G2, P[4])	
Pre	Post	Pre	Post	Pre	Post	Pre	Post	Pre	Post	Pre	Post
5	4,147	5	1,459	5	1,986	5	50	5	233	32	732

Table 3: Seroconversion to Rotavirus Strains Following Immunization *

WA	P	WI61	ST3	VA70	DS-1
(G1, P[8])	(G3, P[8])	(G9, P[8])	(G4, P[6])	(G4, P[8])	(G2, P[4])
100	100	100	100	100	100

**Values represent the percentage of animals that showed a ≥4-fold rise in titer after immunization.*

These data demonstrate that monovalent (P[8]) P2-VP8 subunit vaccine elicits a good heterotypic neutralizing antibody response to strains expressing the P4 but not the P6 genotype. Furthermore, the magnitude of the immune response could vary significantly for strains expressing the homologous P[8] types, as evidenced by a substantially muted response to strain VA70.

1.5.1.4. Protection from Rotavirus Disease in Neonatal Piglets

The protective capacity of the P2-VP8 vaccine against a live oral rotavirus challenge was evaluated in a gnotobiotic piglet model. This model is widely viewed as the most rigorous and relevant animal model for evaluation of rotavirus vaccine efficacy. Three newborn gnotobiotic piglets were immunized via the intramuscular route with three doses of research-grade monovalent (P[8]) P2-VP8 subunit vaccine (100 µg per dose) adjuvanted with 600 µg aluminum phosphate, the first given at birth and the subsequent doses at 10-day intervals. An additional nine piglets served as unvaccinated controls. Seven days after the last vaccination, the animals were challenged with the Wa strain of rotavirus (homologous P[8] serotype) at a dose of 50 $TCID_{50}$ which consistently induces diarrhea in this model (28).

Titers of P[8] VP4-specific antibodies increased following each vaccination with monovalent (P[8]) P2-VP8 subunit vaccine plus aluminum phosphate, with titers first detectable at approximately two weeks following the first vaccination. Titers further increased at 10 days post-challenge, indicating a boosting effect from natural exposure to a homotypic challenge.

Titers of rotavirus-neutralizing antibodies increased significantly by Post-immunization Day (PID) 27, one week following the final vaccination. Titers increased substantially within one week of homotypic challenge in monovalent (P[8]) P2-VP8 subunit vaccine treated animals compared to concurrent control animals (Figure 3).

Figure 3: Rotavirus Geometric Mean Neutralizing Antibody Titers Following Vaccination and Challenge

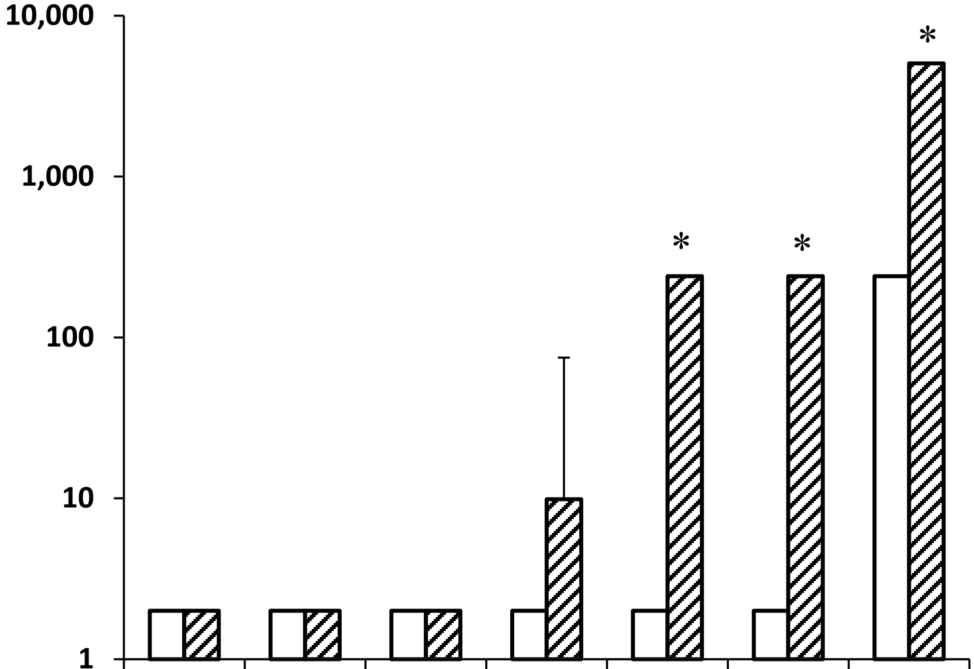

	PID 0	PID 6	PID 13	PID 20	PID 27	PCD 2	PCD 7
□ Control	2	2	2	2	2	2	240
▨ P2-VP8*	2	2	2	9.9	240	240	5054

$^*p < 0.001$

Shedding of the Wa rotavirus was measured after challenge in control unimmunized gnotobiotic piglets and piglets receiving three inoculations of monovalent (P[8]) P2-VP8 subunit vaccine. Vaccination with monovalent (P[8]) P2-VP8 subunit vaccine induced a drop of more than 10-fold in the titer of virus shed in the stools; however, there was no significant difference between the P2-VP8 vaccine and control pig groups.

Monovalent (P[8]) P2-VP8 subunit vaccine significantly reduced post-challenge diarrhea, as estimated by the use of a cumulative, 4-point scoring system (0 points = normal stool consistency [no diarrhea]; 1 = pasty [mild diarrhea]; 2 = semi liquid [moderate diarrhea]; and 3 = liquid [severe diarrhea]). Days to virus shedding and diarrhea onset in addition to the duration of virus shedding and diarrhea in control and immunized piglets is shown in Figure 4. The delayed onset and shortening of diarrhea was statistically significant in vaccinated animals compared to concurrent controls. Immunization did not significantly ($p > 0.05$) alter the onset of virus shedding nor the mean number of days virus was shed post challenge.

Figure 4: Days to Onset of Diarrhea and Viral Shedding by Treatment

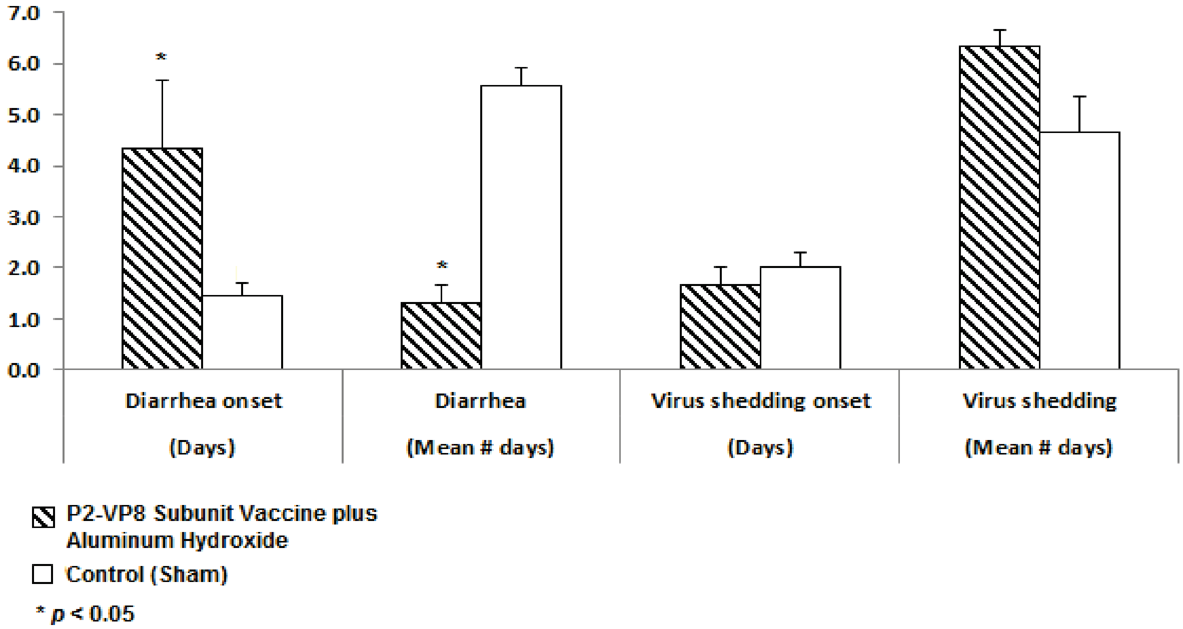

Immunization with the monovalent (P[8]) P2-VP8 subunit vaccine conferred significant protection against rotavirus diarrhea, as gauged by both time of onset and severity after an oral challenge. While the monovalent (P[8]) P2-VP8 subunit vaccine did not completely prevent disease, it was superior to a heat-inactivated rotavirus vaccine (IRV) previously tested in the same model (28). This model has also been used to test live oral rotavirus vaccines. These vaccines, though protective, induce diarrhea when administered to gnotobiotic piglets. Numerous field trials evaluating live oral rotavirus vaccines have consistently demonstrated that they do not prevent infection or substantially reduce mild illness; rather, their protective efficacy is primarily manifested against severe cases of diarrhea. In the piglet study, the monovalent (P[8]) P2-VP8 subunit vaccine significantly delayed the onset of diarrhea, significantly reduced the duration of diarrhea, and significantly reduced the cumulative diarrhea score. These effects may potentially lead to protection against severe diarrhea in infants, the goal of rotavirus immunization.

1.5.2. GLP Immunogenicity and Toxicology Study in NZW Rabbits

Monovalent ([P8]) P2-VP8 subunit vaccine plus aluminum hydroxide [$Al(OH)_3$] was well-tolerated and immunogenic at 30 or 60 µg. No clinical toxicity was observed during the in-life phase. Changes in clinical pathology were transient, expected with immune stimulation associated with vaccination, and non-adverse. Microscopic changes observed one day following the final administration of monovalent (P[8]) P2-VP8 subunit vaccine were limited to the injection site and were resolved or resolving following a four-week recovery period.

1.5.2.1. Study Description

Saline control or monovalent (P[8]) P2-VP8 subunit vaccine (30 or 60 µg, with or without 0.56 mg aluminum hydroxide [$Al(OH)_3$]) was administered in a 0.5 mL volume intramuscularly at 14 day intervals on Study Days 1, 15, 29, and 43, and a unique site (left and right quadriceps and dorsal lumbar muscles) was used for each injection.

Animals were observed for dose-site reactogenicity, clinical observations (including ophthalmology), body weight changes, food consumption, body temperatures, and clinical pathology (hematology, coagulation parameters, and clinical chemistry). Animals were sacrificed two days or 6 weeks following the final dose of vaccine. A full set of tissues were examined microscopically at two days (Study Day 45) following the final dose of vaccine. Injection sites and underlying muscle tissue were examined six weeks following the final dose of vaccine.

1.5.2.2. Toxicology Results

Vaccination with monovalent (P[8]) P2-VP8 subunit vaccine with or without aluminum hydroxide [$Al(OH)_3$] adjuvant was well-tolerated in NZW rabbits at doses of 30 and 60 µg.

There were no treatment-related, clinically significant observations on study, and no changes or pain assessed by observing reaction to palpation were observed at any dose sites. Following immunization, particularly subsequent to the first dose on Study Day 1, slight decreases in body weights were observed, but animals generally gained weight on a week to week basis. There were no treatment-related significant differences in food consumption, post-dose body temperatures, hematology, ophthalmology, or organ weights.

Slightly, but statistically significant, lower creatinine levels were observed on Study Day 3 in females treated with 60 µg monovalent (P[8]) P2-VP8 subunit vaccine and aluminum hydroxide [$Al(OH)_3$] adjuvant and on Study Day 8 in females treated with 30 µg monovalent (P[8]) P2-VP8 subunit vaccine and aluminum hydroxide [$Al(OH)_3$] or 60 µg monovalent (P[8]) P2-VP8 subunit vaccine with or without aluminum hydroxide [$Al(OH)_3$]; however, these changes were not considered toxicologically significant. At Study Day 3 and 8 slight, but statistically significant, increases in fibrinogen were observed in animals treated with 60 µg monovalent (P[8]) P2-VP8 subunit vaccine with or without aluminum hydroxide [$Al(OH)_3$] compared to concurrent controls, which is consistent with inflammatory processes associated with vaccination. At Day 45, all treated animals had significantly higher levels of fibrinogen in an apparent dose-related manner, with those animals treated with aluminum hydroxide [$Al(OH)_3$] having slightly higher fibrinogen levels at each dose than those animals treated with monovalent (P[8]) P2-VP8 subunit vaccine alone.

Males and females treated with 60 µg monovalent (P[8]) P2-VP8 subunit vaccine with or without aluminum hydroxide [$Al(OH)_3$] had statistically significantly higher prothrombin time (PT) at Day 3. For groups immunized with vaccine alone, significantly higher PT was observed on Day 8, Day 45 (females only), and Day 85 (females only) compared to concurrent controls. No mean or individual animal values exceeded the upper limit of the historical range for New Zealand White rabbits at AVANZA Laboratories and animals assigned to the vaccinated groups, particularly Groups 4 & 5, had substantially higher baseline values for PT than control animals, which contributed to the post-vaccination differences from control observed.

When PT values were compared to the baseline values for each test group, only females treated with 60 µg P2-VP8 alone had significant increases over baseline on Day 8 ($p = 0.0003$). Slight elevations in PT over baseline were observed in animals treated with 60 µg P2-VP8 with or without aluminum hydroxide [$Al(OH)_3$] on Day 3, although they were not statistically significant. Given the modest increases in PT values seen and the temporal nature of any increases over baseline, these changes were considered to be non-adverse and clinically irrelevant.

With the exception of a single female in Group 4 (60 µg monovalent (P[8]) P2-VP8 subunit vaccine) with subcutaneous red discoloration at the Day 43 injection site on Day 45, there were no gross observations at either necropsy.

Test article-related microscopic findings were limited to the injection sites, and were considered non-adverse based on minimal to mild severity and apparent reversibility (28). Test article effects associated with monovalent (P[8]) P2-VP8 subunit vaccine plus aluminum hydroxide [$Al(OH)_3$] were minimal to mild accumulations of macrophages with abundant slightly basophilic, finely granular cytoplasm (termed “foamy macrophages”) in the subcutaneous tissue and/or skeletal muscle interstitium. These macrophages were observed at each injection site (sites 1-4) in one to several animals and are likely related to co-administration of the vaccine with aluminum hydroxide [$Al(OH)_3$].

Minimal to mild inflammation consisting of a mixture of heterophils and mononuclear inflammatory cells occurred in the dermis or subcutis of animals in all groups, including controls, suggesting these findings are related to mechanical injury from the injection procedure. A few animals in each vaccine-treated group had a similar finding in the underlying skeletal muscle.

The incidence of inflammation was low in all groups at injection sites 1 and 2, indicating a recovery of acute changes related to vaccination. At injection sites 3 and 4, the incidence of inflammation was slightly higher for groups receiving monovalent (P[8]) P2-VP8 subunit vaccine with or without aluminum hydroxide [$Al(OH)_3$]. At injection site 4, the incidence of inflammation was increased in a dose-related manner, with the highest incidence found in Groups 4 and 5, which received 60 µg monovalent (P[8]) P2-VP8 subunit vaccine with or without aluminum hydroxide [$Al(OH)_3$]. All other findings had similar incidence in control and treated groups, and/or were common spontaneous findings in laboratory rabbits of this age.

Six weeks after the final dose (Day 85), test article-related residual foamy macrophages were present in the dermis and subcutaneous tissue at injection site 3 of one male treated with 60 µg monovalent (P[8]) P2-VP8 subunit vaccine plus aluminum hydroxide [$Al(OH)_3$]. Minimal mixed cell inflammation and minimal to mild hemorrhage also persisted in one or more animals at each injection site. The incidence of these findings was lower than observed at the terminal sacrifice, indicating resolution was in progress. A delayed-onset change was minimal mineralization in the subcutaneous tissue at injection site 3 in one male vaccinated with 60 µg monovalent (P[8]) P2-VP8 subunit vaccine plus aluminum hydroxide [$Al(OH)_3$]. This finding was minimal in extent and considered non-adverse.

1.5.2.3. Immunogenicity Results

The immunogenicity of the monovalent (P[8]) P2-VP8 subunit rotavirus vaccine was demonstrated in NZW rabbits in the GLP toxicology study. As described above, animals were administered four doses of 30 µg or 60 µg monovalent (P[8]) P2-VP8 subunit vaccine alone or adsorbed to aluminum hydroxide [$Al(OH)_3$] adjuvant on Study Days 1, 15, 29, and 43. Serum samples for immunological analyses were collected at baseline and two days or six weeks following the final vaccination on Study Days 45 and 85, respectively.

Immunization with 30 or 60 µg of monovalent (P[8]) P2-VP8 subunit vaccine formulations induced a 4-fold or greater rise in serum IgG anti-P2-VP8 antibody levels in all treated animals on Study Day 45 or Study Day 85. No control animals immunized with saline manifested a

similar rise in antibody at any time point, with all samples tested being at the lower limit of quantitation. The addition of aluminum hydroxide [$Al(OH)_3$] as an adjuvant potentiated the immune response, as determined by comparing GMTs in 3 of 4 analyses performed (Table 4).

Table 4: Geometric Mean Titers by Treatment Following Four Doses of Monovalent (P[8]) P2-VP8 Subunit Vaccine with or without Aluminum Hydroxide [$Al(OH)_3$]

Group	Treatment	Geometric Mean Titer (units/mL)	
		Day 45	Day 85
1	Vehicle Control (0.9% USP Saline)	<20.41 ± 0	<20.41 ± 0
2	30 µg P2-VP8 subunit vaccine	19,042 ± 13,567	11,404 ± 2,787
3	30 µg P2-VP8 subunit vaccine + 0.56 mg aluminum hydroxide adjuvant	22,342 ± 9,694	21,953 ± 6,381
4	60 µg P2-VP8 subunit vaccine	13,096 ± 26,601	6,223 ± 11,697
5	60 µg P2-VP8 subunit vaccine + 0.56 mg aluminum hydroxide adjuvant	25,881 ± 18,718	20,215 ± 11,807

As shown in Figure 5, the presence of aluminum hydroxide [$Al(OH)_3$] did not result in a statistically significant rise in GMT on Study Day 45 (two days after the fourth dose of vaccine was given) when the 30 µg dose was compared ($p = 0.05$), but did so for the 60 µg dose ($p = 0.041$). At Study Day 85, the addition of the aluminum hydroxide [$Al(OH)_3$] adjuvant resulted in significantly higher GMT both for the 30 µg treated group ($p = 0.001$) and 60 µg treated group ($p = 0.024$).

Figure 5: Geometric Mean Titers by Interval and Treatment

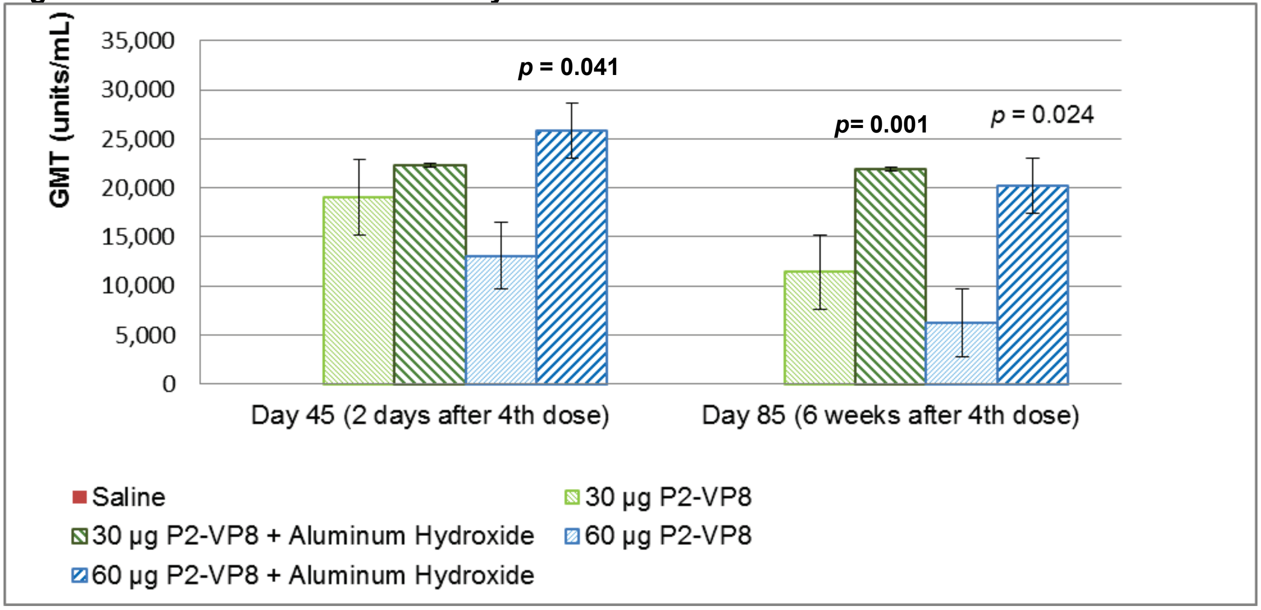

Rotavirus neutralizing antibody titers were measured against the Wa strain at baseline and on Study Day 85, six weeks following the final vaccination. On Study Day 85, neutralizing antibody titers were detectable in all animals vaccinated with 60 µg monovalent (P[8]) P2-VP8 subunit vaccine with or without aluminum hydroxide [$Al(OH)_3$]. Animals receiving aluminum hydroxide [$Al(OH)_3$] adjuvant had higher neutralizing antibody titers than those not receiving adjuvanted vaccine.

1.6. Summary of Nonclinical Studies with Trivalent (P[4], P[6], P[8]) P2-VP8 Vaccine

The safety and immunogenicity of the trivalent rotavirus P2-VP8 subunit vaccine was evaluated in guinea pigs and rabbits. An overview of these preclinical studies supporting the development of this vaccine is provided below in Table 5. Information about these studies is presented in the subsequent subsections.

Table 5: Preclinical Studies Supporting Trivalent Rotavirus P2-VP8 Subunit Vaccine Development

Study Title	Study Site	Study Type & Compliance	Animal Model	Adjuvant	Location of Summary
Evaluation of the Antibody Response in Guinea Pigs Administered trivalent or monovalent vaccines	Spring Valley Laboratories, Woodbine, MD	Immunogenicity Non-GLP	Guinea Pig	Aluminum hydroxide	Section 1.6.1
Repeat Dose Intramuscular Safety and Immunogenicity Study of P2-VP8 Subunit (with and without Aluminum Hydroxide Adjuvant) in Rabbits	MPI Research, Mattawan, MI	Immunogenicity Toxicology GLP	New Zealand White Rabbits	Aluminum Hydroxide	Section 1.6.2

1.6.1. Evaluation of the Antibody Response in Guinea Pigs Administered Either Trivalent or Monovalent P2-VP8 Vaccine

Trivalent vaccine was produced by combining the three individual monovalent P2-VP8 vaccines, adsorbed onto aluminum hydroxide, and used to immunize guinea pigs. Animals received three doses of vaccine at bi-weekly intervals with serum samples taken at baseline and two weeks after the third dose for determination of IgG binding and rotavirus neutralizing antibodies. Immunization with the individual P2-VP8 proteins evoked not only a robust binding antibody response to the homologous antigen but also to the other two vaccine antigens (Table 6). This is not surprising given the level of homology (~65-85%) at the DNA level. In all cases, the GMT after immunization with the trivalent vaccine was comparable to or slightly higher than that seen with the individual components, indicating no blunting of the immune response due to antigen load. However, the overall response engendered by the monovalent P2-VP8-P[6] vaccine was lowest both to the homologous P[6] and heterologous P[4] and P[8]antigens. A similar trend was noted for the neutralizing antibody response (Table 7) where post-immunization titers were lowest to the P[6] vaccine component. While the GMTs for the P[8] and P[6] strains were

comparable in groups of animals immunized with either the trivalent or monovalent vaccines, that for P[4] strain was higher in animals immunized with the monovalent vaccine (338 versus 131, respectively). This could be partially attributed to the fact that one animal in the monovalent group mounted a very high response (1280) given the relatively small number of animals per group (N=6).

Table 6: Serum IgG Binding Antibody Responses in Guinea Pigs following vaccination with either Monovalent or Trivalent P-VP8 Vaccines.

Vaccine Group	Post-immunization Geometric Mean Serum IgG Titer (Range) to Vaccine Antigens[a]		
	P2-VP8 [P4]	P2-VP8 [P6]	P2-VP8 [P8]
P2-VP8 [P8]	50745 (7190-156777)	13747 (4154-33805)	81534 (7847-287569)
P2-VP8 [P6]	31575 (2588-81094)	71363 (10510-175924)	12467 (865-32614)
P2-VP8 [P4]	198808 (53675-326972)	22494 (13429-44099)	70988 (28188-112081)
Trivalent P2-VP8 [P8] + [P6] + [P4]	202245 (152332-281906)	72945 (43079-113257)	131298 (91187-162513)

a: Animals (groups of 6) were immunized on days 1, 14 and 28 with 30 µg of each antigen. Pre-immunization titers were negative at the lowest dilution tested with a titer <78.

Table 7. Neutralizing Antibody titers to Strains of Rotavirus Expressing Various P Types

Vaccine Group	Geometric Mean Neutralizing Antibody Titers (Range)					
	Wa (G1P8)		DS1 (G2P4)		1076 (G2P6)	
	Pre	*Day 43*	*Pre*	*Day 43*	*Pre*	*Day 43*
P2-VP8 [P8]	5 (5-5)	453 (26-1352)	6 (5-18)	41 (15-125)	5 (5-5)	6 (5-5)
P2-VP8 [P4]	5 (5-5)	53 (27-132)	10 (5-37)	338 (133-1280)	5 (5-5)	9 (5-30)
P2-VP8 [P6]	5 (5-5)	7 (5-18)	8 (5-22)	12 (5-35)	5 (5-5)	40 (5-198)
Trivalent P2-VP8 [P8] + [P6] + [P4]	5 (5-5)	380 (48-1156)	18 (5-78)	131 (104-165)	5 (5-5)	37 (5-118)

Pre-immunization titers were <10 for all samples and assigned a titer of 5.

1.6.2. GLP Immunogenicity and Toxicology Study of the Trivalent P2-VP8 Subunit Vaccine in NZW Rabbits

Trivalent P2-VP8 subunit vaccine plus aluminum hydroxide [$Al(OH)_3$] was well-tolerated and immunogenic at 180 µg. No clinical toxicity was observed during the in-life phase. Changes in clinical pathology were transient, expected with immune stimulation associated with vaccination, and non-adverse. Microscopic changes observed two days following the final administration of trivalent P2-VP8 subunit vaccine were limited to the injection site and were resolved or resolving following a four-week recovery period.

1.6.2.1. Study Description

Saline control or trivalent P2-VP8 subunit vaccine (180 µg, comprising of 60 µg of the P[8], P[6], and P[4] serotype subunits) with 0.56 mg aluminum hydroxide [$Al(OH)_3$]) was administered in a 0.5 mL volume intramuscularly at 14 day intervals on Study Days 1, 15, 29, and 43, and a unique site (the cranial and caudal aspects of the left and right erector spinae muscle) was used for each injection.

Animals were observed for dose-site reactogenicity, clinical observations (including ophthalmology), body weight changes, food consumption, body temperatures, and clinical pathology (hematology, coagulation parameters, and clinical chemistry). Animals were sacrificed two days or 4 weeks following the final dose of vaccine. A full set of tissues were examined microscopically at two days (Study Day 45) following the final dose of vaccine. Injection sites and underlying muscle tissue were examined four weeks following the final dose of vaccine.

1.6.2.2. Toxicology Results

Vaccination with trivalent P2-VP8 subunit vaccine with aluminum hydroxide [$Al(OH)_3$] adjuvant was well-tolerated in NZW rabbits at a dose of 180 µg.

There were no test-article related clinical observations, including ophthalmology, noted on the study. One animal assigned to the vaccine group was noted to have a thin body condition and in appetence, primarily in the recovery phase of the study. Because this finding was consistent with lower food consumption prior to and throughout the study and the animal was able to maintain or gain body weight during the study, this finding was not considered to be related to treatment. Dermal site scoring was performed at each dose site for three days following each vaccination. No positive dermal scores were noted for any animals. There were no test article-related effects on body temperatures. There were slight increases in body temperatures at 6 and 24 hours postdose relative to predose values for each collection interval; however, these were noted for both control and treated groups. There were no adverse effects on body weights throughout the study, and animals gained weight. Lower mean food consumption was noted in the vaccine groups; however, these reduced values were due to a few treated males with consistently low food consumption values Due to the low magnitude and lack of correlating clinical observations or decreases group mean body weights, these decreases in food consumption were not considered adverse.

There were no test article-related effects among hematology or clinical chemistry parameters at any interval. All mean and individual values were considered within expected ranges for biological and/or procedure-related variation despite occasional mean values that reached statistical significance. Two days following the final vaccination (Day 45), in animals of both sexes receiving the test article, there were minimal decreases in APTT (up to -11%), relative to pretest means, minimal increases in fibrinogen (up to +20%) at termination in treated animals of both sexes, and mild increases in CRP (up to 9.1x), relative to pretest means. All of these findings were considered test article-related but not adverse; they had generally resolved by the recovery interval. Furthermore, increases of CRP, while higher than baseline, were still within normal historical range at MPI Research. These findings were typical of a mild immune/inflammatory response expected with vaccine administration. All other mean and individual values were considered within expected ranges for biological variation.

There were no consistent test article-related macroscopic observations or differences in organ weights. All macroscopic findings and organ weight variations were considered to be incidental to test article administration or typical for those seen in rabbits of this sex, strain, and age. Test article-related microscopic findings were seen only at the injection sites for all groups. The

injection site findings were not considered to be adverse, given the low severity, lack of systemic findings, and lack of associated clinical findings.

Test article-related microscopic findings at the terminal necropsy consisted of increased incidence and/or severity of a constellation of findings including granulomatous, acute, and chronic inflammation of the muscle; histiocytic infiltration and mixed cell infiltration/inflammation of the subcutaneous tissues; hemorrhage of the muscle and subcutaneous tissues; and myofiber degeneration/necrosis. Following a 4-week recovery, there was a strong trend towards recovery in all groups, with generally reduced incidence and severity of most findings compared to terminal groups.

1.6.2.3. Immunogenicity Results

The immunogenicity of the trivalent P2-VP8 subunit rotavirus vaccine was demonstrated in NZW rabbits in the GLP toxicology study. As described above, animals were administered four doses of 180 µg (60 µg [P8] P2-VP8 + 60 µg [P6] P2-VP8 + 60 µg [P4] P2-VP8) subunit vaccine adsorbed to aluminum hydroxide [$Al(OH)_3$] adjuvant on Study Days 1, 15, 29, and 43. Serum samples for immunological analyses were collected at baseline and two days following the final vaccination on Study Day 45.

At baseline, some animals in the control and vaccine groups had low, but detectable, titers to one or more of the P2-VP8 constructs. This may be the result of subclinical rotavirus infections in these animals or due to non-specific binding in the assay. The titers are several-fold lower than those observed in the treated group post-vaccination.

All animals vaccinated with trivalent P2-VP8 had substantial titers to all three serotypes on Day 45, as shown in the table, below. The response to the P4 and P8 vaccine components were comparable and roughly 2-fold greater than that attained for the P6 component, similar to what was observed in guinea pigs.

While appreciable titers were observed on Day 45 in some control animals, titers in vaccinated animals were significantly higher ($p < 0.0000002$).

Table 8. Serum IgG Binding Antibody Responses in NZW Rabbits Following Vaccination with 180 µg Trivalent P2-VP8 Vaccine (Study Day 45)

Vaccine Group	Post-immunization Geometric Mean Serum IgG Titer (Range) to Vaccine Antigens*		
	P2-VP8 [P4]	P2-VP8 [P6]	P2-VP8 [P8]
1: Control (Saline)	4.3 (<LLQ – 955)	5.9 (<LLQ – 958)	3.1 (<LLQ – 301)
2: Trivalent P2-VP8 [P8] + [P6] + [P4]	96639 (13585 - 283551)	55561 (7471 - 148253)	102963 (17786 - 252081)

LLQ < 78 units/mL

**Note: geometric mean was calculated for controls by setting all values <LLQ to a value of "1"*

1.7. Clinical Studies

1.7.1. First-in-human testing of monovalent (P[8]) P2-VP8 subunit vaccine in adults (VAC 009)

The first clinical testing of the monovalent (P[8]) P2-VP8 subunit rotavirus vaccine was performed in 18-45 year old adults in North America. The trial was a double-blinded, randomized, placebo-controlled dose-escalation study, in which study volunteers were randomized into three dose level cohorts of 16 individuals (12 vaccine recipients and 4 placebo recipients). Participants in each cohort received three intramuscular injections four weeks apart of one of three dose levels of vaccine (10 µg, 30 µg and 60 µg) or placebo (saline).

Overall, the vaccine was well-tolerated at all three dose levels, was associated with only mild transient local reactogenicity, and no safety concerns were identified. Thirty-four of the 36 volunteers allocated to receive vaccine received all 3 injections (11/12 in the 10 µg cohort, 11/12 in the 30 µg cohort and 12/12 in the 60 µg cohort (10/12 placebo recipients received all 3 vaccines). The vaccine recipient in the 10 µg cohort who did not receive all three vaccines was discontinued due to an unrelated acute illness (pneumonia), and the vaccine recipient in the 30 µg cohort who did not receive all three vaccines was discontinued due to reasons unrelated to the study.

Only one volunteer experienced an objective local reaction greater than mild, and that volunteer received placebo (Table 9). One vaccine recipient each in the 10 and 60 µg cohorts complained of greater than mild pain/tenderness (and none in the 30 µg cohort).

Table 9: Maximum Injection Site Reactogenicity per Participant

Severity	Pain N (%)	Tenderness N (%)	Itching N (%)	Redness N (%)	Swelling N (%)
**Placebo**					
None	10 (83.3)	8 (66.7)	9 (75.0)	11 (91.7)	11 (91.7)
Mild	1 (8.3)	3 (25.0)	3 (25.0)	1 (8.3)	0
Moderate	1 (8.3)	1 (8.3)	0	0	1 (8.3)
**10 µg**					
None	6 (50.0)	5 (41.7)	11 (91.7)	11 (91.7)	10 (83.3)
Mild	5 (41.7)	6 (50.0)	1 (8.3)	1 (8.3)	2 (16.7)
Moderate	1 (8.3)	1 (8.3)	0	0	0
**30 µg**					
None	10 (83.3)	7 (58.3)	11 (91.7)	11 (91.7)	10 (83.3)
Mild	2 (16.7)	5 (41.7)	1 (8.3)	1 (8.3)	2 (16.7)
Moderate	0	0	0	0	0
**60 µg**					
None	8 (66.7)		11 (91.7)	9 (75.0)	10 (83.3)
Mild	3 (25.0)	8 (66.7)	1 (8.3)	3 (25.0)	2 (16.7)
Moderate	1 (8.3)	0	0	0	0

Similarly, systemic reactions were relatively uncommon and restricted to mild (Table 10) No volunteers experienced severe reactions, and moderate reactions were limited to fatigue in one volunteer in each of the groups receiving placebo, 10 µg or 60 µg. None of the volunteers experienced fever.

Table 10: Maximum Systemic Reactogenicity per Participant

Severity	Fever N (%)	Nausea N (%)	Vomiting N (%)	Arthralgia N (%)	Chills N (%)	Fatigue N (%)	Headache N (%)	Myalgia N (%)
Placebo								
None	12 (100)	10 (83.3)	12 (100)	10 (83.3)	11 (91.7)	10 (83.3)	10 (83.3)	11 (91.7)
Mild	0	2 (16.7)	0	2 (16.7)	1 (8.3)	1 (8.3)	2 (16.7)	1 (8.3)
Moderate	0	0	0	0	0	1 (8.3)	0	0
10 µg								
None	12 (100)	11 (91.7)	11 (91.7)	11 (91.7)	11 (91.7)	9 (75.0)	10 (83.3)	10 (83.3)
Mild	0	1 (8.3)	1 (8.3)	1 (8.3)	1 (8.3)	2 (16.7)	2 (16.7)	2 (16.7)
Moderate	0	0	0	0	0	1 (8.3)	0	0
30 µg								
None	12 (100)	12 (100)	12 (100)	11 (91.7)	11 (91.7)	11 (91.7)	10 (83.3)	11 (91.7)
Mild	0	0	0	1 (8.3)	1 (8.3)	1 (8.3)	2 (16.7)	1 (8.3)
Moderate	0	0	0	0	0	0	0	0
60 µg								
None	12 (100)	10 (83.3)	12 (100)	9 (75.0)	11 (91.7)	10 (83.3)	9 (75.0)	9 (75.0)
Mild	0	2 (16.7)	0	3 (25.0)	1 (8.3)	1 (8.3)	3 (25.0)	3 (25.0)
Moderate	0	0	0	0	0	1 (8.3)	0	0

No volunteers experienced a vaccine-related serious adverse event; one participant experienced an SAE 123 days after the last vaccination, a hospitalization for severe pneumonia assessed as unrelated to the vaccine. That participant had previously experienced a severe, non-serious AE that was assessed as unrelated to receipt of vaccine (back pain secondary to trauma). The AE data are summarized in Table 11. There were no discernible trends in AEs or safety monitoring laboratory values, which include basic clinical chemistry and hematology.

Table 11: Maximum Adverse Event Severity per Participant

AE Severity	Placebo	10 µg	30 µg	60 µg
Mild	5 (41.7%)	6 (50.0%)	2 (16.7%)	6 (50.0%)
Moderate	1 (8.3%)	3 (25.0%)	3 (25.0%)	0
Severe	0	0	0	1 (8.3%)
Any	6 (50.0%)	9 (75.0%)	5 (41.7%)	7 (58.3%)

Sera were assessed for IgG and IgA responses to the P2-VP8 antigen by ELISA (Figure 6/Table 12 and Figure 7/Table 13, respectively) and for neutralizing antibodies to the homologous rotavirus Wa (Figure 8/Table 14) at baseline and one month after each of the three study injections. Sera were also assessed for neutralizing antibodies responses at Day 84 to heterologous rotavirus strains (Table 15) and for rotavirus-specific antibody in lymphocyte supernatant (ALS) to P2-VP8 antigen 7 days after the third study injection (Table 16).

As noted in Figures 6 and 7, almost all vaccine recipients demonstrated greater than 4-fold rise in IgG and IgA response to P2-VP8 antigen by ELISA after three vaccinations: only one vaccine recipient did not demonstrate an IgG response (in the 30 µg group) and all vaccine recipients demonstrated IgA responses.

Figure 6: Serum Anti-P2-VP8 IgG Titers by ELISA

Vertical lines represent geometric mean titer and corresponding 95% confidence limits

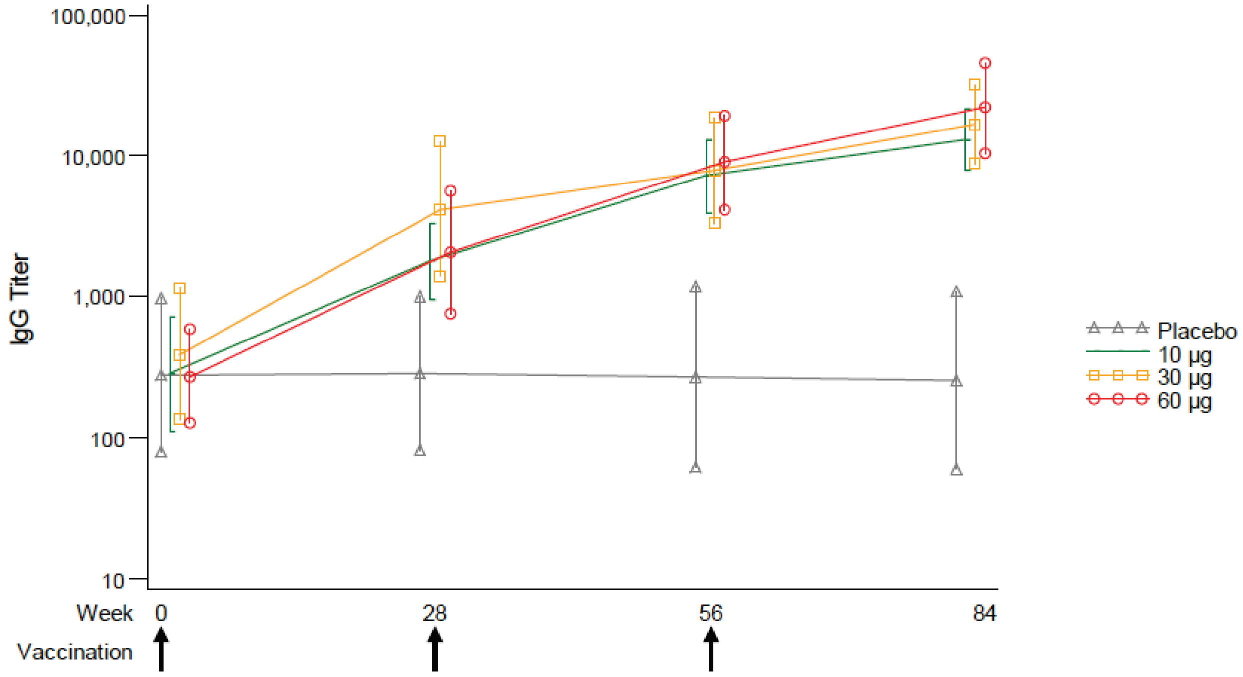

Table 12: Proportion of Participants with ≥4-fold Increase in Anti-P2-VP8 IgG Titers by ELISA

Treatment Arm	Day 28	Day 56	Day 84
Placebo	0.0%	0.0%	0.0%
10 µg	58.3%	90.9%	100%
30 µg	66.7%	83.3%	91.7%
60 µg	58.3%	91.7%	100%

Figure 7: Serum Anti-P2-VP8 IgA Titers by ELISA

Vertical lines represent geometric mean titer and corresponding 95% confidence limits

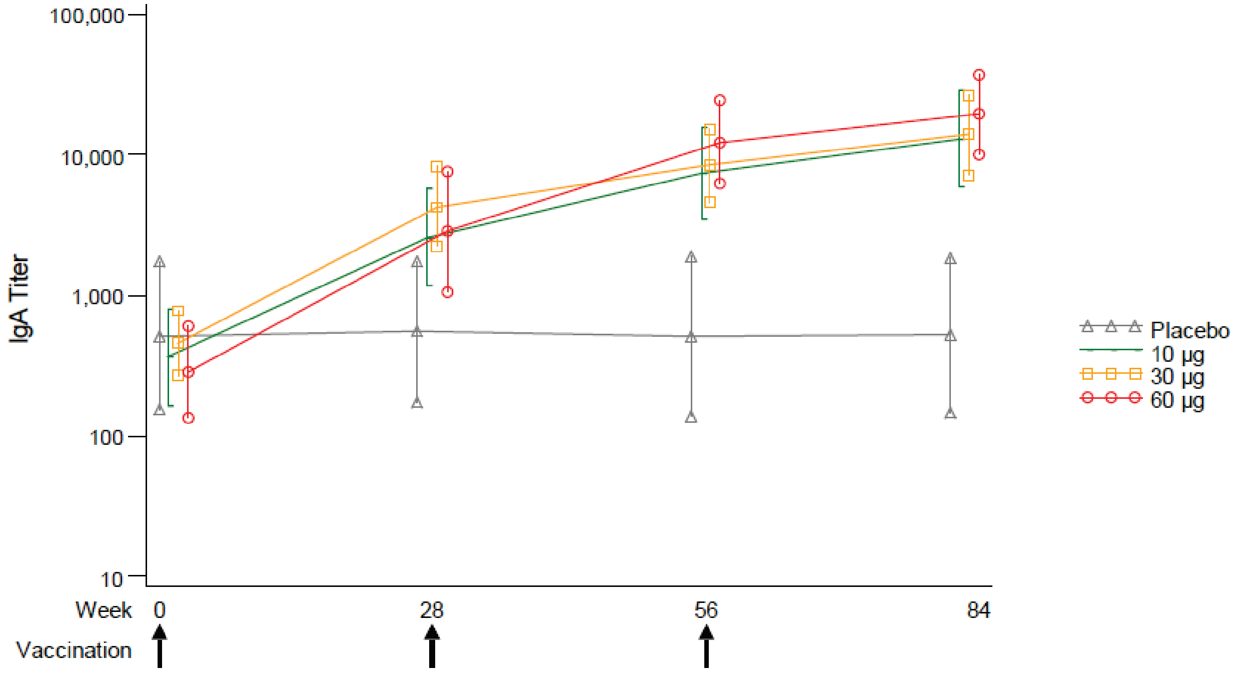

Table 13: Proportion of Participants with ≥4-fold Increase in Anti-P2-VP8 IgA Titers by ELISA

Treatment Arm	Day 28	Day 56	Day 84
Placebo	0.0%	0.0%	0.0%
10 μg	66.7%	90.9%	100%
30 μg	75.0%	91.7%	100%
60 μg	66.7%	91.7%	100%

Neutralizing antibody responses were also encouraging (Figure 8/Table 14), with clear increases in geometric mean titers (GMTs) for all three dose levels at one month post-third study injection (Day 84 in Figure 8) compared to pre-vaccination levels. Of particular interest, of the 12 vaccine recipients (at any dose level) with baseline titers <100, 10 (83%) demonstrated a >4-fold increase in neutralizing antibody; 5 of these 12 were in the 60 μg group, and all demonstrated a >4-fold increase.

Neutralizing antibody responses to heterologous rotavirus strains were most robust to P[8] strains, moderate to the P[4] strain and fairly limited to the P[6] strain (Table 11).

Figure 8: Serum Neutralizing Antibody Titers against Wa Strain

Vertical lines represent geometric mean titer and corresponding 95% confidence limits

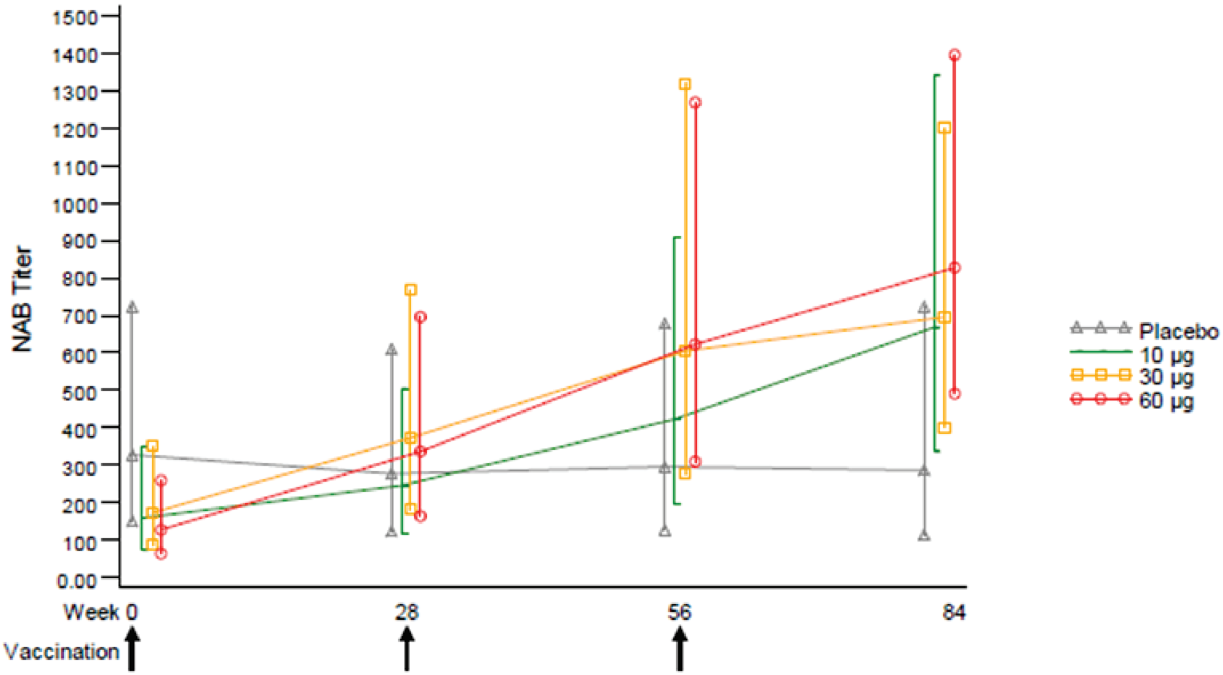

Table 14: Proportion of Participants with ≥4-fold Increase in Neutralizing Antibody Titers against Wa Strain

Treatment Arm	Day 28	Day 56	Day 84
Placebo	0.0%	0.0%	0.0%
10 µg	0.0%	36.4%	66.7%
30 µg	16.7%	41.7%	41.7%
60 µg	33.3%	50.0%	58.3%

Table 15: Proportion of Participants with ≥4-fold Increase in Neutralizing Antibody Titers at Day 84 against Other Strains

Strain	Treatment Arm	Percent response (95% CI)
89-12 (G1P[8])	10 µg	83 (52, 98)
	30 µg	67 (35, 90)
	60 µg	83 (52, 98)
P (G3P[8])	10 µg	58 (28, 85)
	30 µg	67 (35, 90)
	60 µg	83 (52, 98)
DS1 (G2P[4])	10 µg	0 (0, 26)
	30 µg	50 (21, 79)
	60 µg	58 (28, 85)
ST3 (G4P[6])	10 µg	0 (0, 26)
	30 µg	8 (0, 38)
	60 µg	17 (2, 48)

None of the participants had detectable ALS responses at baseline. As seen in Table 16, there was a steady rise in proportion of participants with detectable IgA responses 7 days after the first dose of vaccine from lowest to highest dose level. However the sample sizes are too small for meaningful analysis of statistical significance.

Table 16: Participants with Detectable Antibody in Lymphocyte Supernatant (ALS) to P2-VP8 Antigen 7 Days after the Third Study Injection

Treatment Arm	IgA	IgG
Placebo	0	0
10 µg	4 (33.3%)	3 (25.0%)
30 µg	5 (41.7%)	3 (25.0%)
60 µg	8 (67.7%)	4 (33.3%)

1.7.2. Clinical testing of monovalent (P[8]) P2-VP8 subunit vaccine in toddlers and infants in South Africa (VAC 013)

Clinical testing of the monovalent (P[8]) P2-VP8 subunit rotavirus vaccine was initiated in South African toddlers and infants in 2014, and that trial is now fully enrolled. Preliminary primary safety and immunogenicity results are available and presented below. The trial is a double-blind, randomized, placebo-controlled dose-escalation study. Dose escalation, from 10 to 30 to 60 µg, was first performed in small groups of toddlers (12 vaccine and 2 placebo recipients per dose level), before initiation of similar dose escalation in 6-8 week old infants (12 vaccine and 4 placebo recipients per dose level). The two highest tolerated doses, 30 and 60 µg, were selected for expanded testing in infants, with simultaneous randomization to each of the two doses or placebo (38 infants in each of the three arms). EPI vaccines, excluding oral rotavirus vaccine, were co-administered with the three study vaccines in infants (at 6, 10 and 14 weeks of age).

The vaccine has been generally well-tolerated at all three dose levels. The data presented in Tables 17 and 18, for local and systemic reactogenicity are complete, and the data presented for adverse events in Table 19 represent the available data through 28 days after the last vaccination of all participants. The data are presented by age-group and dose level. In both toddlers and infants, when local reactogenicity was reported, it was transient, rarely greater than mild, and never severe (Table 17). When present, systemic reactogenicity was also transient and generally mild, without discernable dose effect (Table 18). Late in the expanded infant cohort experience, two participants were reported to have severe irritability following one of their three study injections (co-administered with EPI vaccinations), one of whom received placebo. These events occurred after the second dose in one participant and third in the other, and were brief and transient (≤24 hours at the severe intensity level in both cases, completely resolved in one day in one participant and within 3 days in the other).

In the dose-escalation phase of the testing in the infant cohorts, the study injections were paused temporarily due to findings of severe neutropenia on post-vaccination laboratory monitoring in three participants (two infants and one toddler). The Safety Review Committee (SRC, composed of the principal investigator, Emmes medical monitor, PATH medical officer and two, independent local experts) interpretation of the review of the baseline screening data (including data for those who were screened-out), unblinded aggregate data for neutrophil counts at the scheduled 7 day post-vaccination time point, the changes relative to baseline, and the unblinded allocation of the three participants with severe neutropenia was that the data did not indicate a relationship to receipt of the study vaccine, and study injections were resumed.

Table 17: Maximum Injection Site Reactogenicity per Participant in VAC 013

Severity	Pain/ Tenderness N (%)	Erythema/ Redness N (%)	Induration/ Swelling N (%)	Itching N (%)
**Cohort A, 10µg**				
None	11 (91.7)	12 (100.0)	12 (100.0)	11 (91.7)
Mild	1 (8.3)	0	0	0
Moderate	0	0	0	1 (8.3)
Severe	0	0	0	0
**Cohort A, 30µg**				
None	8 (66.7)	11 (91.7)	11 (91.7)	9 (75.0)
Mild	4 (33.3)	0	1 (8.3)	3 (25.0)
Moderate	0	1 (8.3)	0	0
Severe	0	0	0	0
**Cohort A, 60µg**				
None	10 (83.3)	11 (91.7)	12 (100.0)	12 (100.0)
Mild	2 (16.7)	1 (8.3)	0	0
Moderate	0	0	0	0
Severe	0	0	0	0
**Cohort A, Placebo**				
None	5 (83.3)	6 (100.0)	6 (100.0)	6 (100.0)
Mild	1 (16.7)	0	0	0
Moderate	0	0	0	0
Severe	0	0	0	0
**Cohort B1, 10µg**				
None	7 (58.3)	8 (66.7)	8 (66.7)	10 (83.3)
Mild	5 (41.7)	4 (33.3)	4 (33.3)	2 (16.7)
Moderate	0	0	0	0
Severe	0	0	0	0
**Cohort B1, 30µg**				
None	5 (41.7)	9 (75.0)	8 (66.7)	12 (100.0)
Mild	6 (50.0)	3 (25.0)	4 (33.3)	0
Moderate	1 (8.3)	0	0	0
Severe	0	0	0	0

Table 17: Maximum Injection Site Reactogenicity per Participant in VAC 013 (cont'd)

Severity	Pain/ Tenderness N (%)	Erythema/ Redness N (%)	Induration/ Swelling N (%)	Itching N (%)
**Cohort B1, 60µg**				
None	2 (16.7)	8 (66.7)	9 (75.0)	9 (75.0)
Mild	8 (66.7)	4 (33.3)	3 (25.0)	3 (25.0)
Moderate	2 (16.7)	0	0	0
Severe	0	0	0	0
Pot. LT	0	0	0	0
**Cohort B1, Placebo**				
None	4 (33.3)	8 (66.7)	9 (75.0)	6 (50.0)
Mild	8 (66.7)	4 (33.3)	3 (25.0)	6 (50.0)
Moderate	0	0	0	0
Severe	0	0	0	0
**Cohort B2, 30µg**				
None	17 (44.7)	25 (65.8)	23 (60.5)	29 (76.3)
Mild	19 (50.0)	13 (34.2)	15 (39.5)	9 (23.7)
Moderate	2 (5.3)	0	0	0
Severe	0	0	0	0
**Cohort B2, 60µg**				
None	18 (47.4)	27 (71.1)	29 (76.3)	32 (84.2)
Mild	20 (52.6)	10 (26.3)	9 (23.7)	6 (15.8)
Moderate	0	1 (2.6)	0	0
Severe	0	0	0	0
**Cohort B2, Placebo**				
None	16 (42.1)	22 (57.9)	27 (71.1)	29 (76.3)
Mild	18 (47.4)	15 (39.5)	10 (26.3)	9 (23.7)
Moderate	4 (10.5)	1 (2.6)	1 (2.6)	0
Severe	0	0	0	0

Table 18: Maximum Systemic Reactogenicity per Participant in VAC 013

Severity	Vomiting N (%)	Decreased Appetite N (%)	Irritability N (%)	Decreased Activity N (%)	Acute Systemic Allergic Reaction N (%)	Non-Axillary Temperature N (%)
**Cohort A, 10µg**						
None	12 (100.0)	9 (75.0)	11 (91.7)	11 (91.7)	12 (100.0)	12 (100.0)
Mild	0	2 (16.7)	1 (8.3)	1 (8.3)	0	0
Moderate	0	1 (8.3)	0	0	0	0
Severe	0	0	0	0	0	0
**Cohort A, 30µg**						
None	10 (83.3)	7 (58.3)	12 (100.0)	11 (91.7)	12 (100.0)	11 (91.7)
Mild	2 (16.7)	5 (41.7)	0	1 (8.3)	0	1 (8.3)
Moderate	0	0	0	0	0	0
Severe	0	0	0	0	0	0
**Cohort A, 60µg**						
None	11 (91.7)	8 (66.7)	11 (91.7)	10 (83.3)	12 (100.0)	10 (83.3)
Mild	1 (8.3)	4 (33.3)	1 (8.3)	2 (16.7)	0	1 (8.3)
Moderate	0	0	0	0	0	1 (8.3)
Severe	0	0	0	0	0	0
**Cohort A, Placebo**						
None	6 (100.0)	5 (83.3)	6 (100.0)	6 (100.0)	6 (100.0)	5 (83.3)
Mild	0	1 (16.7)[1]	0	0	0	0
Moderate	0	0	0	0	0	1 (16.7)
Severe	0	0	0	0	0	0
**Cohort B1, 10µg**						
None	9 (75.0)	8 (66.7)	7 (58.3)	9 (75.0)	12 (100.0)	6 (50.0)
Mild	3 (25.0)	4 (33.3)	5 (41.7)	3 (25.0)	0	6 (50.0)
Moderate	0	0	0	0	0	0
Severe	0	0	0	0	0	0
**Cohort B1, 30µg**						
None	10 (83.3)	7 (58.3)	4 (33.3)	6 (50.0)	12 (100.0)	8 (66.7)
Mild	2 (16.7)	4 (33.3)	7 (58.3)	5 (41.7)	0	4 (33.3)
Moderate	0	1 (8.3)	1 (8.3)	1 (8.3)	0	0
Severe	0	0	0	0	0	0
**Cohort B1, 60µg**						
None	6 (50.0)	7 (58.3)	5 (41.7)	8 (66.7)	12 (100.0)	8 (66.7)
Mild	6 (50.0)	5 (41.7)	6 (50.0)	4 (33.3)	0	3 (25.0)
Moderate	0	0	1 (8.3)	0	0	0
Severe	0	0	0	0	0	0
Pot. LT	0	0	0	0	0	1 (8.3)

Table 18: Maximum Systemic Reactogenicity per Participant in VAC 013 (cont'd)

Severity	Vomiting N (%)	Decreased Appetite N (%)	Irritability N (%)	Decreased Activity N (%)	Acute Systemic Allergic Reaction N (%)	Non-Axillary Temperature N (%)
**Cohort B1, Placebo**						
None	5 (41.7)	5 (41.7)	3 (25.0)	7 (58.3)	12 (100.0)	6 (50.0)
Mild	6 (50.0)	6 (50.0)	8 (66.7)	3 (25.0)	0	6 (50.0)
Moderate	1 (8.3)	1 (8.3)	1 (8.3)	2 (16.7)	0	0
Severe	0	0	0	0	0	0
**Cohort B2, 30µg**						
None	29 (76.3)	23 (60.5)	18 (47.4)	21 (55.3)	38 (100.0)	21 (55.3)
Mild	8 (21.1)	13 (34.2)	13 (34.2)	14 (36.8)	0	16 (42.1)
Moderate	1 (2.6)	2 (5.3)	6 (15.8)	3 (7.9)	0	1 (2.6)
Severe	0	0	1 (2.6)	0	0	0
**Cohort B2, 60µg**						
None	24 (63.2)	26 (68.4)	18 (47.4)	27 (71.1)	38 (100.0)	22 (57.9)
Mild	13 (34.2)	11 (28.9)	19 (50.0)	10 (26.3)	0	15 (39.5)
Moderate	1 (2.6)	1 (2.6)	1 (2.6)	1 (2.6)	0	1 (2.6)
Severe	0	0	0	0	0	0
**Cohort B2, Placebo**						
None	23 (60.5)	20 (52.6)	15 (39.5)	23 (60.5)	38 (100.0)	23 (60.5)
Mild	15 (39.5)	13 (34.2)	19 (50.0)	9 (23.7)	0	13 (34.2)
Moderate	0	5 (13.2)	3 (7.9)	6 (15.8)	0	2 (5.3)
Severe	0	0	1 (2.6)	0	0	0

Through 11 September 2015, 19 SAEs have been reported in 15 participants, none of which are assessed as related to study product. The study was paused late in the study for the SRC to review the death of a participant which occurred the day following the third vaccination. The participant had tolerated the first two vaccinations well, but had been hospitalized two weeks before the third vaccination with a severe respiratory infection. The infant had been put down to rest while the mother took a bath, and when the mother returned the infant had died. Laboratory results of nasopharyngeal secretions during the hospitalization only became available after the infant's death, with PCR revealing respiratory syncytial virus B, cytomegalovirus and *Pneumocystis jiroveci* (in substantial quantity). A minimally invasive autopsy did not reveal any major organ pathology, however the lung tissue was inadequate for investigation. The SRC assessment after review of the available data was that there was not a reasonable possibility that the study product caused the event.

As stated above, the aggregate unblinded primary safety data will be submitted for review to the MCC, FDA, ECs/IRBs, SRC and DSMB prior to study initiation.

Table 19: Maximum Adverse Event Severity per Participant in VAC 013

	Cohort A				Cohort B1				Cohort B2		
AE Severity	10 µg N (%)	30 µg N (%)	60 µg N (%)	Placebo N (%)	10 µg N (%)	30 µg N (%)	60 µg N (%)	Placebo N (%)	30 µg N (%)	60 µg N (%)	Placebo N (%)
Mild	7 (58.3)	6 (50.0)	10 (83.3)	2 (33.3)	11 (91.7)	10 (83.3)	11 (91.7)	10 (83.3)	32 (84.2)	33 (86.8)	33 (86.8)
Moderate	1 (8.3)	1 (8.3)	0	1 (16.7)	1 (8.3)	0	1 (8.3)	1 (8.3)	4 (10.5)	2 (5.3)	2 (5.3)
Severe	0	0	0	0	0	1 (8.3)	0	1 (8.3)	2 (5.3)	1 (2.6)	0
Any	8 (66.7)	7 (58.3)	10 (83.3)	3 (50.0)	12 (100.0)	11 (91.7)	12 (100.0)	12 (100.0)	38 (100.0)	36 (94.7)	35 (92.1)

Excludes 19 SAEs in 15 subjects. One from cohort B1 and 18 from cohort B2

Tables 20-22 present the primary serology results in the infant cohorts. Whether IgG responses were adjusted for maternal antibody or not (Table 20), there were close to universal responses, with substantial increases in titer as demonstrated by mean-fold increase in GMT. In infants receiving the 30 µg dose, the GMT rose from a baseline value of 107 to a post-vaccination value of 9,583. Although not as dramatic as the IgG responses, there were good IgA responses to P2-VP8, with the seroresponse rate over 80% in infants receiving 30 µg of vaccine. The 60 µg dose did not appear to provide any better response than the 30 µg dose, although the study was not powered for that comparison.

Table 20: Anti-P2-VP8 IgG Responses in Infants

		Unadjusted		Adjusted*	
Study arm	N	Mean fold-increase in GMT	4-fold increase n (%)	Mean fold-increase in GMT	4-fold increase n (%)
Placebo	45	0.3	1 (2.2%)	1.4	9 (20.0%)
10µg	12	131	12 (100%)	556	12 (100%)
30µg	47	89	46 (97.9%)	369	46 (97.9%)
60µg	47	58	46 (97.9%)	239	47 (100.0%)

*Adjusted for maternal antibody

Table 21: Anti-P2-VP8 IgA Responses in Infants

Study arm	N	Mean fold-increase	4-fold increase n (%)
Placebo	45	1.9	9 (20.0%)
10µg	12	7.1	7 (58.3%)
30µg	47	10.0	38 (80.9%)
60µg	47	6.7	32 (68.1%)

Although unadjusted neutralizing antibody responses to the Wa strain from which the vaccine is derived were modest, when adjusted for maternal antibody a high proportion of infants demonstrated a seroresponse. This was the case both when the protocol stipulated 4-fold increase was used to define seroresponse and when the laboratory's more recent validated cut-off of 2.7-fold was used. Again, although not powered to compare responses to the two dose level, the 60 µg dose did not appear to provide any better response than the 30 µg dose.

Table 22: Neutralizing Antibody Responses to Wa Strain in Infants

		Unadjusted			Adjusted		
**Study arm**	**N**	**Mean fold-increase**	**4-fold increase n (%)**	**2.7-fold increase n (%)**	**Mean fold-increase**	**4-fold increase n (%)**	**2.7-fold increase n (%)**
Placebo	45	0.2	0 (0%)	1 (2.2%)	0.9	4 (8.9%)	5 (11.1%)
10µg	12	4.2	7 (58.3%)	7 (58.3%)	24.9	11 (91.7%)	12 (100%)
30µg	47	2.5	17 (36.2%)	21 (44.7%)	14.1	38 (80.9%)	42 (89.4%)
60µg	47	1.6	7 (14.9%)	10 (21.3%)	9.1	37 (78.7%)	42 (89.4%)

*Adjusted for maternal antibody

As a test of concept, shedding of vaccine virus was assessed 5, 7 and 9 days following administration of the first dose of Rotarix after completion of the study vaccine series (Table 23). In this proxy model of efficacy, there were substantial reductions in shedding (at any time) in infants who had received vaccine compared to infants who had received placebo. The reduction was most pronounced for infants receiving 30 µg, with a 65% reduction in shedding (p-value = 0.009). In contrast, the reduction in shedding in infants receiving 60 µg of vaccine was 49%.

Table 23: Rotarix Shedding in Infants

Study arm	N	n (%)
Placebo	44	17 (*38.6%*)
30µg	45	6 (*13.3%*)
60µg	46	9 (*19.6%*)
30µg + 60µg	91	15 (*16.5%*)

In summary, the P2-VP8 monovalent vaccine was generally well-tolerated in healthy South African infants, and there was no evidence that the higher vaccine dose (60 µg) provided any benefit in serologic responses or impact on shedding of Rotarix compared to the lower dose (30 µg).

2. HYPOTHESIS, SCIENTIFIC RATIONALE, OBJECTIVES AND STUDY DESIGN

2.1. Study Hypothesis

Primary Hypotheses:

Safety

The trivalent P2-VP8 subunit rotavirus vaccine is safe and well-tolerated in healthy South African adults, toddlers and infants.

Immunogenicity

The trivalent P2-VP8 subunit rotavirus vaccine is immunogenic in healthy South African infants and will induce neutralizing immune responses to at least 2 of the 3 strains from which vaccine antigens are derived in 60% or more of participants in at least one of the study groups.

2.2. Scientific Rationale

Live oral rotavirus vaccines have not performed optimally in developing-country populations. The discrepancy in performance of these vaccines in developed versus developing countries is not a unique feature of rotavirus vaccines; it has also been observed with other oral, live attenuated enteric vaccines, such as those targeted against cholera and poliomyelitis, for which efficacy is diminished in resource poor countries. Strategies targeting the development of inactivated rotavirus vaccines have recently been proposed [3], which are supported by the successful use of parenteral vaccines to control disease caused by several orally transmitted pathogens including

poliovirus, *Vibrio cholera* and *Salmonella typhi*. Most approaches for developing inactivated rotavirus vaccines have focused on eliciting rotavirus neutralizing antibodies directed at either the VP4 or VP7 rotavirus neutralizing antigens. Several direct and indirect observations support the possibility that parenterally-administered, non-replicating rotavirus vaccines will likely be successful, including observations derived from natural rotavirus infection studies in infants, rotavirus challenge studies in adult volunteers, human rotavirus vaccine trials, and animal rotavirus vaccine studies. The induction of non-neutralizing antibody responses may result in mucosal protection, either through the induction of local IgA responses or, more likely, through "leaking" into the gut of IgG antibodies generated by adjuvanted parenteral injection of non-replicating vaccines. This possibility is supported by the finding that high titers of circulating anti-rotavirus IgG are transmitted trans-placentally in humans. Maternally derived IgG antibody appears to modulate vaccine takes and be protective against early rotavirus disease, and passively administered antibody may prevent or diminish disease [32, 33].

Clinical testing of the monovalent (P[8]) P2-VP8 subunit vaccine has demonstrated the vaccine to be well-tolerated, without safety signals. In adults, neutralizing antibody results against P[8] strains were promising, but less so against P[4] strains and disappointing against P[6] strains. Therefore, a trivalent vaccine, comprising antigens from all 3 strains has been developed to optimize responses against all three strains, which together are responsible for the vast majority of global disease burden. Preclinical testing indicates improved responses to P[4] and P[6] with the trivalent vaccine.

The dose levels to be assessed in this protocol are based on results in infants in VAC 013 (see section 1.7.2), in which the 60 µg dose did not seem to provide any better responses in terms of immune response or impact on Rotarix shedding, compared to the 30 µg dose. The highest dose to be assessed in this protocol is an aggregated dose of 90 µg, composed of 30 µg of each of the three antigens.

Further, as in VAC 013, the impact of the trivalent vaccine on the replication of Rotarix, which will be administered to the infants after completion of the 3-dose regimen of P2-VP8, will be assessed. Impact on shedding of Rotarix will be interpreted as a surrogate of protection. This assessment will provide an indication of whether the addition of two more antigens alters the impact observed in VAC 013 with the monovalent vaccine.

2.3. Overall Clinical Development Strategy

The ultimate goal of the P2-VP8 subunit rotavirus vaccine clinical development plan is to demonstrate the safety and efficacy of this vaccine approach in a series of studies to gain sufficient information to achieve both local registration of the vaccine in the country(ies) where the pivotal trials will be conducted and prequalification by WHO to support product acquisition by GAVI, UNICEF or others for distribution, in general, to low income countries. Briefly, the overall clinical development plan consists of: 1) the first in human phase 1 safety trial of the monovalent (P[8]) P2-VP8 subunit vaccine performed in the US in 2013; 2) the phase 1-2 descending age safety and immunogenicity trial of the monovalent (P[8]) P2-VP8 subunit vaccine in toddlers and infants currently approaching completion in South Africa; 3) the proposed phase 1-2 descending age safety and immunogenicity trial of the trivalent (P[4], P[6] and P[8]) P2-VP8 subunit vaccine in adults, toddlers and infants in South Africa; and 4) phase 3 efficacy trials to evaluate efficacy of the trivalent P2-VP8 subunit vaccine against severe rotavirus diarrhea. Additional clinical trial assessments could include: clinical assessment of lot-to-lot consistency (potentially could be combined with the efficacy trials); assessment of interference of the vaccine with the immune responses to routine EPI vaccinations; and assessment of vaccine safety and immunogenicity in other populations.

2.4. Study Design

The trial is a double-blind, randomized, placebo-controlled dose-escalation study in which two dose-levels (30 µg and 90 µg) of vaccine will be tested in adults and toddlers and then three dose-levels will be assessed in infants (15 µg, 30 µg and 90 µg). In Group A, cohorts of 15 adults (12 vaccine and 3 placebo recipients) per dose level will receive three study injections at 4 week intervals (consistent with the regimen in the adult study of the monovalent vaccine), advancing from the first to the second dose-level after assessment of safety data through the first week after the first injection (A1). Based on the safety data in the adult group, Group B cohorts of 15 toddlers (12 vaccine recipients and 3 placebo recipients) per dose level will receive a single intramuscular injection (consistent with the regimen administered to toddlers in the current study of the monovalent vaccine in toddlers), advancing from the first to the second dose-level after assessment of safety data through the first week after the injection in the lower dose toddler group (B1) and all available data for the adult group. Based on the safety data in the toddler cohorts, Group C cohorts of 16 infants (12 vaccine recipients and 4 placebo recipients) per dose level will receive three study injections at 4 week intervals (concomitant with EPI vaccinations, with the exception of rotavirus vaccination), starting at ≥6 and <8 weeks of age. Advancing from the first to the second dose-level in Group C will occur after assessment of safety data through the first week after the first injection (C1), as well as all available data for toddlers and adults. Advancing from the second to the third dose-level (90 µg) in Group C will occur after assessment of safety data through the first week after the first injection of the second dose-level (30 µg). If all three dose levels are tolerated in the infant dose-escalation phase (Part C), they will be assessed in an expanded infant cohort (Group D) of 552 infants (138 at each dose level and 138 placebo recipients). All infants will receive Rotarix at 4, 8 and 12 weeks after the third study injection (study Days 84, 112 and 140), and fecal shedding of Rotarix will be assessed during the week after the first dose in a subset of infants. Three doses of Rotarix are to be administered rather than two to counterbalance the remote theoretical possibility that the P2-VP8 subunit vaccine could reduce response to Rotarix.

2.4.1. Primary Objectives

Safety

- To evaluate the safety and tolerability of the trivalent P2-VP8 subunit rotavirus vaccine at escalating dose levels in healthy South African adults, toddlers and infants

Immunogenicity

- To evaluate the immunogenicity of three doses of the trivalent P2-VP8 subunit rotavirus vaccine at different dose levels in healthy South African infants

2.4.2. Secondary Objectives

Safety

- To evaluate the longer term safety (through 6 months after the last vaccination) of the trivalent P2-VP8 subunit rotavirus vaccine at escalating dose levels in healthy South African adults, toddlers and infants

Immunogenicity

- To evaluate the immunogenicity of two doses of the trivalent P2-VP8 subunit rotavirus vaccine at different dose levels in healthy South African infants
- To evaluate the immunogenicity of the trivalent P2-VP8 subunit rotavirus vaccine at different dose levels in healthy South African adults and toddlers

2.4.3. Exploratory Objective

Efficacy

- To evaluate the impact of the trivalent P2-VP8 subunit rotavirus vaccination on shedding of Rotarix subsequently administered in healthy South African infants as a test of concept

2.4.4. Primary Endpoints

Safety

- Number of SAEs through 28 days after the last study injection
- Number of AEs through 28 days after the last study injection
- Number of vaccine-induced local and systemic reactions

Immunogenicity (infants):

- Proportion of infants with anti-P2-VP8 IgG & IgA seroresponses by ELISA (four-fold increase in antibody titers between baseline and 4-weeks post-third study injection) in assays using each of the three vaccine antigens (P[4], P[6] and P[8])
- Proportion of infants with neutralizing antibody responses (2.7-fold increases in antibody titers between baseline and 4-weeks post-third study injection) to each of the three rotavirus strains from which the vaccine antigens are derived, as well as heterologous strains
- Anti-P2-VP8 IgG & IgA geometric mean titers (GMT) (baseline and 4 weeks post third study injection) in ELISA assays using each of the three vaccine antigens (P[4], P[6] and P[8])
- Neutralizing antibody GMT (baseline and 4 weeks post-third study injection) to each of the three rotavirus strains from which the vaccine antigens are derived, as well as heterologous strains
- Proportion of infants with neutralizing antibody responses to at least two of the three strains from which the vaccine antigens are derived (4-weeks post-third study injection)

2.4.5. Secondary Endpoints:

Safety (all 3 age-groups)

- Number of SAEs at any time during the study
- Number of AEs at any time during the study

Immunogenicity (infants)

- Proportion of infants with anti-P2-VP8 IgG & IgA seroresponses by ELISA (four-fold increase in antibody titers between baseline and 4-weeks post-second study injection) in assays using each of the three vaccine antigens (P[4], P[6] and P[8])
- Proportion of infants with neutralizing antibody responses (2.7-fold increases in antibody titers between baseline and 4-weeks post-second study injection) to each of the three rotavirus strains from which the vaccine antigens are derived, as well as heterologous strains
- Anti-P2-VP8 IgG & IgA geometric mean titers (GMT) (baseline and 4 weeks post-second study injection) in ELISA assays using each of the three vaccine antigens (P[4], P[6] and P[8])
- Neutralizing antibody GMT (baseline and 4 weeks post-second study injection) to each of the three rotavirus strains from which the vaccine antigens are derived, as well as heterologous strains

Immunogenicity (adults and toddlers)

- Proportion of adults and toddlers with anti-P2-VP8 IgG & IgA seroresponses by ELISA (four-fold increase in antibody titers between baseline and 4-weeks post-final study injection) in assays using each of the three vaccine antigens (P[4], P[6] and P[8])
- Proportion of adults and toddlers with neutralizing antibody responses (2.7-fold increases in antibody titers between baseline and 4-weeks post-final study injection) to each of the three rotavirus strains from which the vaccine antigens are derived, as well as heterologous strains
- Anti-P2-VP8 IgG & IgA geometric mean titers (GMT) (baseline and 4 weeks post final study injection) in ELISA assays using each of the three vaccine antigens (P[4], P[6] and P[8])
- Neutralizing antibody GMT (baseline and 4 weeks post-final study injection) to each of the three rotavirus strains from which the vaccine antigens are derived, as well as heterologous strains
- Proportion of adults and toddlers with neutralizing antibody responses to at least two of the three strains from which the vaccine antigens are derived

2.4.6. Exploratory Endpoint

- Proportion of participants shedding Rotarix after administration of Rotarix “challenge”

3. STUDY PRODUCT

3.1. Product Description

The P2-VP8 subunit rotavirus vaccine is made by inserting a codon optimized synthetic gene encoding the VP8 region of rotavirus VP4 fused to the P2 T-cell epitope coding sequence of tetanus toxin into the Pj411 proprietary cloning vector developed by DNA 2.0, Menlo Park, CA. The vector carries a kanamycin resistance gene as a selection marker. The vector was transfected into the BL21 strain of E. coli. The fusion protein is purified from Isopropyl β-D-1-thiogalactopyranoside (IPTG)-induced and physically lysed cultures using standard column chromatographic techniques employing Q-Sepharose and Butyl 650 as resins in addition to ultrafiltration and diafiltration. The purified protein is co-formulated with aluminum hydroxide [$Al(OH)_3$] (see Section 3.3).

3.2. Products and Manufacturers

Trivalent P2-VP8 subunit rotavirus vaccine is manufactured and supplied by the Walter Reed Army Institute of Research (WRAIR) Pilot Bioproduction Facility (BPF).

Sterile aluminum hydroxide adjuvant Lot Nr 1862 (2 mg of aluminum per mL) was prepared, labeled and sampled for QC release at WRAIR and will be used as a diluent to prepare the 60 µg vaccine dose. The vials were stored at 2-8°C at WRAIR.

Sodium Chloride 0.9%, USP for Injection will be used to dilute the active P2-VP8 vaccine to final dosing concentration and will be used for the placebo for the study. The research pharmacy at each site will obtain enough supply of the same lot of Sodium Chloride 0.9% for Injection, USP from Pharma Logistical Solutions (Pty) Ltd to complete the study.

3.3. Presentation and Formulation

The trivalent P2-VP8 vaccine is formulated as a sterile suspension containing a total of 360 µg of protein (120 µg of each P type) per mL adsorbed to aluminum hydroxide (1.125 mg of aluminum per mL in a phosphate buffer, pH 7). The vaccine is contained within a 2 mL borosilicate glass vial sealed with a butyl rubber stopper and a crimped metal collar. The vaccine is stored at 2-8°C. To produce the 15, 30 and 90 µg doses (containing 5, 10 and 30 µg of each P2-VP8 vaccine component, respectively), the vaccine is diluted with the aluminum hydroxide adjuvant diluent within 6 hours of administration (aluminum hydroxide diluted to a concentration of 1.125 mg/mL before being used to dilute the vaccine).

The doses of the trivalent P2-VP8 subunit vaccine will be prepared in the pharmacy. Blinded study syringes will be prepared for the study coordinator for administration to the volunteers.

3.4. Stability and Storage

The trivalent P2-VP8 subunit vaccine is stored at 2-8°C until used. A pilot lot of vaccine has been shown to be stable when stored for 6 months at 2-8°C (study ongoing). The P2-VP8 trivalent vaccine to be used in this study (Lot Nr 1917) is being monitored for stability at 2-8°C per ICH guidelines. The vaccine has been shown to be stable for 3 months (latest time point available). Stability will be evaluated for a minimum of 24 months. The aluminum hydroxide adjuvant (Lot Nr 1862) to be used as a diluent to prepare the 60 µg dose of vaccine has been shown to be stable for 6 months when stored at 2-8°C. Stability studies are ongoing and will be conducted for a minimum of 24 months. Sodium chloride 0.9% for Injection, USP, will be used to prepare the aluminum hydroxide diluent. These 10 mL vials will be stored at room temperature.

3.5. Preparation and Administration

In Groups A and B, at each dose level 12 participants will be randomized to receive vaccine and three (3) to receive placebo; in Group C, at each dose level 12 participants will be randomized to receive vaccine and four (4) to receive placebo. In Group D, the allocation for the four arms (three dose levels and placebo) will be 1:1:1:1. Vaccine is administered as a 0.5 mL intramuscular injection: in Groups A, C and D, study injections will be administered on Day 0, Day 28 and Day 56; and in Group B, the single injection will be administered on Day 0. Study injections will be administered with 25 gauge, 25 mm needles into muscle at 90°.

Table 24 illustrates the dosing schedule.

Table 24: Dosing Schedule

Group	P2-VP8 dose	N	Study Day		
			0	28	56
**A Adults**	30 µg	12	X	X	X
	Placebo	3	X	X	X
	90 µg	12	X	X	X
	Placebo	3	X	X	X
**B Toddlers**	30 µg	12	X		
	Placebo	3	X		
	90 µg	12	X		
	Placebo	3	X		
**C Infants**	15 µg	12	X	X	X
	Placebo	4	X	X	X
	30 µg	12	X	X	X
	Placebo	4	X	X	X
	90 µg	12	X	X	X
	Placebo	4	X	X	X
**D Infants**	15 µg*	138	X	X	X
	30 µg*	138	X	X	X
	90 µg*	138	X	X	X
	Placebo	138	X	X	X

X = Injection day
*If dose tolerated in Group C

3.5.1. Dose Preparation and Administration

Dose preparation, as described in detail in the Pharmacy Preparation Manual, will be carried out by a qualified unblinded research pharmacist and witnessed by another un-blinded study staff member. Investigational product will be dispensed in a masked syringe with administration needle, labeled with participant number, and will be administered by blinded clinic staff.

Investigational product is prepared at the research pharmacy at each of the trial sites. The prepared vaccine will be stored in the research pharmacy refrigerator (2°C – 8°C) for up to 6 hours after preparation prior to administration.

The study product will be administered intramuscularly: to the deltoid in adults and to the anterolateral thigh muscle in toddlers and infants.

3.5.2. Accountability and Disposal

The site pharmacist is required to maintain complete records of all study products received from the Sponsor and will be responsible for maintaining an accurate record of the randomization codes, inventory, and an accountability record of vaccine and placebo supplies for this study. The site pharmacist will also be responsible for ensuring the security of these documents. The site will receive instruction from the IND Sponsor regarding the final disposition of any remaining study products. Partially used vials will not be used for human administration or for *in vitro* experimental studies. All unused study products must be disposed of according to the Sponsor's instructions.

4. STUDY POPULATION

4.1. Clinical Trial Sites

This study is a multisite clinical trial, to be performed in South Africa at the Family Clinical Research Unit (FAM-CRU) at Tygerberg Children’s Hospital, the Respiratory and Meningeal Pathogens Research Unit (RMPRU) in Soweto, and the Shandukani Research Centre in Hillbrow, Johannesburg. The study population will be recruited, screened and qualified by site staff under the direction of the PIs at each site.

4.2. Study Population

The study population will consist of healthy adults, toddlers and infants from the communities in the vicinity of the study clinics. Adult participants and parents of toddler and infant participants will be fully informed about the study, after which they will provide consent for participation.

4.3. Eligibility

Participants will be in general good health: in the adult group, participants will be ≥18 and ≤45 years of age; in the toddler cohort, participants will be ≥2 and <3 years of age; and in the infant cohorts will be ≥6 and <8 weeks old, at the time of enrollment. Potential volunteer families of infants may be contacted from before the babies are born through the noted targeted age. Final eligibility determination will depend on the results of the medical history, clinical examination, screening laboratory tests and fulfillment of all the inclusion and absence of any of the exclusion criteria, and appropriate understanding of the study and completion of the consent process by adult participants and parents of toddler and infant participants.

Investigators should always use good clinical judgment in considering a participant’s overall fitness for inclusion in the trial. Some participants may not be appropriate for the study, even if they meet all inclusion/exclusion criteria. For instance, medical, occupational or other conditions present in the parents may make safety evaluations difficult or make toddlers and infants poor candidates for retention. All toddlers and infants targeted for enrollment will need to have parents that can comprehend the purpose of the study and provide written informed consent. In addition, the adult participants and families of toddler and infant participants should be resident in the area without plans to leave the study site during the course of the study.

A sufficient number of healthy adults, toddlers and infants will be screened, with consent, to enroll 30 adults, 30 toddlers and 600 infants in the study.

4.4. Inclusion Criteria

Fulfillment of all of the following criteria is required to accept an adult, toddler or infant in the study:

1. Healthy adults, toddlers and infants as established by medical history and clinical examination before entering the study
2. Age:
    a. Adults: ≥18 and ≤45 years old at time of enrollment
    b. Toddlers: ≥2 and <3 years old at the time of enrollment
    c. Infants: ≥6 and <8 weeks at the time of enrollment
3. Participant (adults) or parental (toddlers and infants) ability and willingness to provide written informed consent
4. Intention for the participant (and for toddlers and infants, the parent) to remain in the area with the child during the study period

5. If female and of childbearing potential, be not breastfeeding and not pregnant (based on a negative serum pregnancy test at screening and a negative urine pregnancy test at each study injection visit, prior to injection), planning to avoid pregnancy for at least 4 weeks after the last injection, and willing to use an adequate method of contraception consistently and have repeated pregnancy tests prior to second and third injections.

4.5. Exclusion Criteria

Any of the following will exclude an adult, toddler or infant from the study:

1. Presence of fever on the day of enrollment (axillary or oral temperature >37.6°C)
2. Acute disease at the time of enrollment
3. Concurrent participation in another clinical trial throughout the entire timeframe for this study
4. Presence of malnutrition or any systemic disorder (cardiovascular, pulmonary, hepatic, renal, gastrointestinal, hematological, endocrine, immunological, dermatological, neurological, cancer or autoimmune disease) as determined by medical history and/or physical examination that would compromise the participant's health or is likely to result in nonconformance to the protocol
5. For infant cohort, history of premature birth (<37 weeks gestation)
6. History of congenital abdominal disorders, intussusception, or abdominal surgery
7. Known or suspected impairment of immunological function based on medical history and physical examination
8. For infant cohort only, prior receipt of rotavirus vaccine
9. A known sensitivity or allergy to any components of the study vaccine
10. History of anaphylactic reaction
11. Major congenital or genetic defect
12. Parents of participating toddlers or infants not able, available or willing to accept active weekly follow-up by the study staff
13. Receipt of any immunoglobulin therapy and/or blood products in the last 6 months or planned administration during the study period
14. History of chronic administration (defined as more than 14 days) of immunosuppressant medications, including corticosteroids, in the last 6 months (those on inhaled or topical steroids may be permitted to participate in the study)
15. Any medical condition in the participant (or parents of toddler and infant participants) that, in the judgment of the investigator, would interfere with or serves as a contraindication to protocol adherence or a participant's (or parents') ability to give informed consent
16. Clinically significant screening laboratory value*
17. HIV infection
    a. For adults and toddlers, to be assessed by HIV ELISA
    b. For infants, to be assessed by PCR, if mother is not known to be negative (negative test result between 24 weeks gestation and screening)

*Grade 1 laboratory abnormalities (see toxicity table in Appendix I) will not be considered to be exclusionary at screening unless judged to be clinically significant by the PI. Potential participants with laboratory values of grade 2 or higher are not to be randomized without concurrence of the Emmes medical monitor and PATH medical officer.

After informed consent has been obtained and the potential participant is identified as meeting inclusion and exclusion criteria for enrollment, the (s)he will be enrolled in the study and assigned a randomization number.

5. STUDY PROCEDURES

5.1. Recruitment

Adults will be invited for screening by means of advertisements in the local community. All advertisements will be approved the Ethics Committee before implementation.

Toddlers will be identified from hospital birth registers. The parent will be contacted and invited to bring the child to RMPRU for screening.

Infants will be identified in the postnatal wards at Chris Hani Baragwanath Hospital after delivery, and the mother will be invited to bring her infant to the RMPRU prior to the first clinic visit for screening and potential enrolment.

At FAMCRU, in Tygerberg, pregnant women will be informed of the study at the Kraaifontein Midwife obstetric unit's antenatal clinic. Those interested will then be followed up for delivery at the midwife obstetric unit. Potential participants will also be identified at the Tygerberg hospital antenatal clinic and followed up at the labour ward of Tygerberg hospital with delivery of the infant.

At the Shandukani Research Centre, pregnant women at antenatal clinics and mothers of infants attending the Day 3 visit will be approached. Recruitment activity will also occur at surrounding City of Johannesburg Region F clinics. The study will be discussed with the parents, and, if they agree, permission will be obtained to collect their names and contact details. Closer to the potential enrollment date, parents will be contacted and invited to visit the Shandukani Research Centre. At that time interested parents will be also asked not to give their infants the 6 week Rotarix vaccine. Recruitment activity will also occur at the 6 week EPI vaccination visit. The site will develop a study-specific pamphlet for parents considering participation in the study.

5.2. Screening, Randomization, and Masking Procedures (for all age-groups)

5.2.1. Initial and Continuing Informed Consent

Informed consent is the process of ensuring that study participants, or, in the case of toddlers and infants, their parents, fully understand the purpose of the study and what will and may happen to them/their children while participating in a research study. Initial written informed consent is required before performance of any study-related procedures. The informed consent process continues throughout the study. Key study concepts are reviewed with the study participants/participants' parents at designated times and as needed, and the review is documented. Additionally, if any new information becomes available that, in the judgment of PVS and/or PIs, may affect the participants'/parents' decision to continue in the trial, such information will be shared with the participants/parents, and they may be asked to sign a new consent form.

5.2.2. Screening – Day -7 (infants) or Day -28 (adults and toddlers) to Day -1

After the site PI or designee has obtained informed consent from participants/parents, the following procedures will be completed during screening to determine study eligibility and may occur over multiple screening visits. Additional screening visits may be scheduled for any follow up as needed, but are not required. At the screening visit(s), the site PI or designee will provide prospective participants (adults) and parents of prospective participants (toddlers and infants) a detailed description of the study objectives and study participation requirements, as well as potential health risks and benefits associated with study participation. Baseline data are obtained during screening, which may occur over the course of several contacts/visits, between 7 days prior to and on Day 0, the day of first injection for infants and from 28 days prior to Day 0 for toddlers and adults. All inclusion/exclusion criteria must be assessed from data obtained within that period, unless otherwise specified in the eligibility criteria. After study information has been provided and the appropriate informed consent has been obtained, the following procedures are performed before enrollment:

a) Confirm written informed consent has been obtained and solicit/discuss any remaining questions the participant/parent may have;
b) Assign participant ID once study specific consent form has been signed;
c) Obtain demographic and contact (e.g., address, telephone, email) information;
d) Obtain medical history;
e) Obtain vaccination history for toddlers and infants (for adults, vaccination history for past 30 days);
f) Obtain history of medication use in the past 30 days;
g) Measure height/length & weight;
h) Perform full physical exam (including vital signs); and
i) Collect blood samples for screening laboratory testing: complete blood count (CBC) (including WBC, hemoglobin, platelet count), alanine transaminase (ALT), total bilirubin, albumin (obtained only at screening) and creatinine (Appendix I).* Serum pregnancy test will be performed on women of childbearing potential.
    a. For adults toddlers, HIV ELISA
    b. For infants, HIV PCR (unless mother has negative test result between 24 weeks gestation and screening)
j) Collect blood samples for immunological testing.

* Grade 1 laboratory abnormalities will not be considered to be exclusionary at screening, unless judged to be clinically significant by the site PI.

5.2.3. Randomization

Enrollment will be performed online using the enrollment module of AdvantageEDC^{SM}, the Emmes Corporation's electronic data capture system. Randomization will occur on the day participants are to receive their first study injection, after confirmation of eligibility and immediately prior to injection.

Participants will be assigned to a coded treatment assignment after demographic and eligibility data have been entered into the system. The unblinded research pharmacist will be provided with the treatment assignment codes for preparation of the vaccine or placebo to be given to each participant. The unblinded research pharmacist will maintain the treatment code list in a secure place.

5.2.4. Masking procedures

The research pharmacist will not reveal the randomization code to any other study staff member, participant or parent. Investigational study product will be prepared by a qualified unblinded research pharmacist and witnessed by another unblinded study staff member. Investigational product will be dispensed in a masked syringe (opaque sheath over the syringe barrel) with administration needle.

5.2.5. Group A (adults)

5.2.5.1. Day 0 – First Dose

Prior to Study Injection

a) Provisionally eligible participants are scheduled to arrive at the study clinic on the morning of Day 0;

b) Interval medical history is obtained, including medications;

c) Vital signs are measured prior to dosing;

d) A full physical exam is performed;

e) New health information is reviewed to determine qualification for enrollment;

f) Inclusion/Exclusion criteria are reviewed to assess continued eligibility;

g) Urine pregnancy test is performed for women of childbearing potential.

h) The prospective injection site (deltoid) is examined visually and by palpation, and the axillary lymph nodes are examined. When possible, the first and third injections are to be administered to the deltoid of the non-dominant arm and the second injection to the dominant arm; and

i) Participants are randomized.

Study Injection

a) If a qualified participant selected for enrollment is disqualified prior to randomization and dose administration (e.g., participant withdraws consent or the site PI reconsiders and disqualifies for a documented reason), another qualified participant will be selected for randomization in place of the disqualified participant.

b) The injection site will be observed prior to dose administration (as above).

c) The prospective injection site will be cleaned with an alcohol swab and allowed to dry completely. The study injection will be administered into the deltoid by inserting the needle (25 gauge, 25 mm) into muscle at 90°, holding the muscle mass stable with the non-injecting hand, drawing back to ensure that the needle is not in a blood vessel, and slowly depressing the plunger. The needle is removed and the injection site gently rubbed with cotton wool.

Post Dose – Observation Period

a) Participants will remain at the site for at least 30 minutes post-injection.

b) After at least 30 minutes: Vital signs and the injection site will be assessed, redness and/or induration will be measured and recorded. Any complaints or reactogenicity signs or symptoms will be assessed and documented.

c) The site PI (or designee) may determine that a participant requires further on-site observation; additional site or clinical assessments may be completed as needed.

d) When all study related procedures are complete and the site PI (or designee) determines that a participant's condition is acceptable, the participant will be discharged from the study clinic.

e) Before discharge, the participant will be provided with supplies (including a thermometer, a transparent plastic tool to assess size of injection site redness and swelling, and a Memory Aid); instructed in their use; and provided with written materials, follow-up visit information and contact telephone numbers for study staff. Participants are to be instructed to contact study staff for any reaction greater than mild.

5.2.5.2. Day 7, 35 and 63 ± 1 day (to be defined by 7 days after most recent study injection day) – Clinic visit follow-up

On the 7th day after each injection (±1 day), the participant will return to the study clinic for the following procedures:

a) Study staff will review and record the participant's interval health history, medication use and the participant's assessment of the post-injection experience through personal interview, assisted by the Memory Aid (See Appendix III).

b) Vital sign measurement, injection site and draining lymph node assessment and, if indicated, a symptom-directed physical examination will be performed.

c) Local and systemic reaction and AE review will be performed by the site PI (or designee). The highest oral temperature written in the Memory Aid each day will be recorded in source document as peak temperature.

d) Findings of site PI or study staff that suggest inaccuracy of reported self-assessments will be clearly documented.

e) Any topics or new information considered by site PI to be important to continued informed consent will be shared with the participant.

f) Blood samples will be obtained for safety monitoring laboratory tests on Day 7 (Appendix I).

g) Study staff will instruct the participant regarding continued health assessment (without a Memory Aid) and need to contact study staff (a) in follow-up of specific AEs, (b) if injection site changes worsen or do not resolve, (c) if systemic symptoms worsen or do not resolve, and (d) if other AEs occur.

h) The next visit is scheduled.

5.2.5.3. Day 28 (-2 to +7 days) and Day 56 (-2 to +7 days) days (to be defined by 28 days after the previous study injection) – Study injection days

Every effort should be made to adhere to the protocol injection schedule windows. However, injections may be given outside the specified window on a case-by-case basis, if approved by the Sponsor, provided that the injection will not have any adverse effect on the participant and that it is in the best interest of the scientific soundness of the research plan. The appropriate follow-up and subsequent injection schedule for each participant with delayed injections will be determined by the Sponsor.

Prior to Dose

The participant returns to the study clinic and the following procedures will occur.

a) Review of interval medical history, including AEs and medication use
b) Vital signs measurement prior to dosing
c) Review of inclusion/exclusion criteria to assure continued eligibility
d) Urine pregnancy test for women of childbearing potential
e) A targeted physical exam, if indicated
f) Examination of the prior injection site and corresponding draining lymph nodes
g) Review of safety laboratory test results from Day 7 at the Day 28 visit before vaccination (and discussed with participants assuming there has not been occasion to do so earlier).
h) Blood samples will be obtained for immunological assays (see Appendix I).
i) Examination (visually and by palpation) of the prospective injection site (deltoid), and axillary lymph nodes

Study Injection

a) The injection site is observed before dose administration.
b) The prospective injection site will be cleaned with an alcohol swab and allowed to dry completely. The study injection will be administered into the deltoid by inserting the needle (25 gauge, 25 mm) into muscle at 90°, holding the muscle mass stable with the non-injecting hand, drawing back to ensure that the needle is not in a blood vessel, and slowly depressing plunger. The needle is removed and the injection site gently rubbed with cotton wool.

Post Dose – Observation Period

a) Participants will remain at the site for at least 30 minutes post-injection.
b) After at least 30 minutes: Vital signs and the injection site will be assessed, redness and/or indurations will be measured and recorded. Any complaints or reactogenicity signs or symptoms will be assessed and documented.
c) The site PI (or designee) may determine that a participant requires further on-site observation; additional site or clinical assessments may be completed as needed.
d) When all study related procedures are complete and the site PI (or designee) determines that a participant's condition is acceptable, the participant will be discharged from the study clinic.

e) Before discharge, the participant will be provided with supplies including a thermometer, ruler for measuring injection site redness and swelling, and a Memory Aid; instructed in their use; and provided with written materials, follow-up visit information and contact telephone numbers for study staff. Participants are to be instructed to contact study staff for any reaction greater than mild.

5.2.5.4. Day 84 ± 4 days (to be defined by 28 days post-3rd study injection day) – Clinic follow-up visit and immunogenicity sampling

On Day 84 (plus or minus 4 calendar days if necessary for participant compliance), the participant will return to the study clinic and the following procedures will occur:

a) Interval medical history will be obtained, including medication use.

b) Vital sign measurement, injection site and draining lymph node assessment and, if indicated, a symptom-directed physical examination will be performed.

c) AEs will be evaluated.

d) Blood samples will be obtained for immunological assays (see Appendix I).

e) The participant will be reminded to contact the study clinic with any new information about chronic illnesses, serious health events, and/or hospitalizations, and the Day 224 follow-up will be scheduled.

5.2.5.5. Day 224 ± 14 days (to be defined by 168 days post-3rd study injection day) – Final participant contact (telephone or clinic visit)

On Day 224 (plus or minus 14 calendar days if necessary for participant compliance), the participant will be contacted by telephone or return to the study clinic for a final follow-up visit and the following procedures occur:

a) Interval medical history will be obtained for SAEs and other major or new chronic medical conditions.

b) A targeted physical exam will be performed, if indicated (request to come to clinic for those being contacted by telephone).

5.2.6. Group B (toddlers)

5.2.6.1. Day 0 – Study Injection Day

Prior to Study Injection

a) Provisionally eligible participants are scheduled to arrive at the study clinic on the morning of Day 0;

b) Interval medical history is obtained and inclusion/exclusion criteria reviewed to determine continuous qualification for enrollment, including medications;

c) Vital signs are measured prior to dosing;

d) A full physical exam is performed;

e) The prospective injection site (anterolateral thigh) is examined visually and by palpation, and the axillary lymph nodes are examined; and

f) Participants are randomized.

Study Injection

a) If a qualified participant selected for enrollment is disqualified prior to randomization and dose administration (e.g., parent withdraws consent or the site PI reconsiders and disqualifies for a documented reason), another qualified participant will be selected for randomization in place of the disqualified participant.

b) The injection site will be observed prior to dose administration (as above).

c) The prospective injection site will be cleaned with an alcohol swab and allowed to dry completely. The study injection will be administered into the anterolateral thigh by inserting the needle (25 gauge, 25 mm) into muscle at 90°, holding the muscle mass stable with the non-injecting hand, drawing back to ensure that the needle is not in a blood vessel, and slowly depressing plunger. The needle is removed and the injection site gently rubbed with cotton wool.

Post Dose – Observation Period

a) Participants will remain at the site for at least 30 minutes post-injection.

b) After at least 30 minutes: Vital signs and the injection site will be assessed, redness and/or induration will be measured and recorded. Any complaints or reactogenicity signs or symptoms will be assessed and documented.

c) The site PI (or designee) may determine that a participant requires further on-site observation; additional site or clinical assessments may be completed as needed.

d) When all study related procedures are complete and the site PI (or designee) determines that a participant's condition is acceptable, the participant will be discharged from the study clinic.

e) Before discharge, the participant's parents will be provided with supplies (including a thermometer, a transparent plastic tool to assess size of injection site redness and swelling, and a Memory Aid); instructed in their use; and provided with written materials, follow-up visit information and contact telephone numbers for study staff. Participants' parents are to be instructed to contact study staff for any reaction greater than mild. Participants are to be assessed in clinic for any local site reaction greater than mild.

5.2.6.2. Day 3 (to be defined by 3 days after study injection day) – Clinic follow-up visit

On the third day after the study injection, the participants will return to the study clinic for the following procedures:

a) Study staff will review and record the participant's interval health history, medication use and the parent's assessment of the post-injection experience through personal interview, assisted by the Memory Aid (See Appendix III).

b) Vital sign measurement, injection site and draining lymph node assessment and, if indicated, a symptom-directed physical examination will be performed.

c) Local and systemic reaction and AE review will be performed by the site PI (or designee). The highest axillary temperature written in the Memory Aid each day will be recorded in source document as peak temperature.

d) Findings of site PI or study staff that suggest inaccuracy of reported parental assessments will be clearly documented.

e) Any topics or new information considered by the site PI to be important to continued informed consent will be shared with the parent.

f) Study staff will instruct the parent regarding continued assessment of the child’s health (and use of Memory Aid) and need to contact study staff (a) in follow-up of specific AEs, (b) if any injection site changes worsen or do not resolve, (c) if systemic symptoms worsen or do not resolve, and (d) if other AEs occur.

g) The next visit for Day 7 is scheduled.

5.2.6.3. Day 7 ± 1 day (to be defined by 7 days after study injection day) – Clinic follow-up visit

On the 7th day after the study injection (±1 day), the participant will return to the study clinic for the following procedures:

a) Study staff will review and record the participant’s interval health history, medication use and the parent’s assessment of the post-injection experience through personal interview, assisted by the Memory Aid (See Appendix III).

b) Vital sign measurement, injection site and draining lymph node assessment and, if indicated, a symptom-directed physical examination will be performed.

c) Local and systemic reaction and AE review will be performed by the site PI (or designee). The highest axillary temperature written in the Memory Aid each day will be recorded in source document as peak temperature.

d) Findings of site PI or study staff that suggest inaccuracy of reported self-assessments will be clearly documented.

e) Any topics or new information considered by site PI to be important to continued informed consent will be shared with the parent.

f) Blood samples will be obtained for safety monitoring laboratory tests on Day 7 (Appendix I).

g) Study staff will instruct the parent regarding continued assessment of the child’s health (without a Memory Aid) and need to contact study staff (a) in follow-up of specific AEs, (b) if injection site changes worsen or do not resolve, (c) if systemic symptoms worsen or do not resolve, and (d) if other AEs occur.

h) The next visit is scheduled.

5.2.6.4. Day 28 ± 4 days (to be defined by 28 days after study injection day) – Clinic follow-up

On Day 28 (plus or minus 4 calendar days if necessary for participant compliance), the participant will return to the study clinic and the following procedures occur:

a) Interval medical history will be obtained, including medication use.

b) Vital sign measurement, injection site and draining lymph node assessment and, if indicated, a symptom-directed physical examination will be performed.

c) AEs will be evaluated and recorded.

d) Blood samples will be obtained for immunological assays (see Appendix I).

e) The participant’s parent will be reminded to contact the study clinic with any new information about chronic illnesses, serious health events, and/or hospitalizations, and the Day 168 follow-up will be scheduled.

5.2.6.5. Day 168 ± 14 days (to be defined by 168 days post-injection day) – Final participant contact (telephone or clinic visit)

On Day 168 (plus or minus 14 calendar days if necessary for participant compliance), the participant's parents will be contacted by telephone or return to the study clinic with the participant for a final follow-up visit and the following procedures will occur:

a) Interval medical history will be obtained for SAEs and other major or new chronic medical conditions.
b) A targeted physical exam will be performed, if indicated (request to come to clinic for those being contacted by telephone).

5.2.7. Groups C and D (infants)

5.2.7.1. Day 0 – First Dose

Prior to Study Injection

a) Provisionally eligible participants are scheduled to arrive at the study clinic on the morning of Day 0;
b) Interval medical history is obtained, including medications;
c) Vital signs are measured prior to dosing;
d) A full physical exam is performed;
e) New health information is reviewed to determine qualification for enrollment;
f) Inclusion/Exclusion criteria are reviewed to assess continued eligibility;
g) The prospective injection site (anterolateral thigh) is examined visually and by palpation, and the inguinal lymph nodes are examined. When possible, the first and third injections are to be administered to the left anterolateral thigh and the second injection to the right anterolateral thigh; and
h) Participants are randomized.

Study Injection

a) If a qualified participant selected for enrollment is disqualified prior to randomization and dose administration (e.g., parent withdraws consent or the site PI reconsiders and disqualifies for a documented reason), another qualified participant will be selected for randomization in place of the disqualified participant.
b) The injection site will be observed prior to dose administration (as above).
c) The prospective injection site will be cleaned with an alcohol swab and allowed to dry completely. The study injection will be administered into the outer aspect of the anterolateral thigh by inserting needle (25 gauge, 25 mm) into muscle at 90°, holding the muscle mass stable with the non-injecting hand, drawing back to ensure that the needle is not in a blood vessel, and slowly depressing plunger. The needle is removed and the injection site gently rubbed with cotton wool.

Post Dose – Observation Period

a) Participants will remain at the site for at least 30 minutes post-injection.
b) After at least 30 minutes: Vital signs and the injection site will be assessed, redness and/or induration will be measured and recorded. Any complaints or reactogenicity signs or symptoms will be assessed and documented.

c) The site PI (or designee) may determine that a participant requires further on-site observation; additional site or clinical assessments may be completed as needed.

d) When all study related procedures are complete and the site PI (or designee) determines that a participant's condition is acceptable, the participant will be discharged from the study clinic.

e) Before discharge, the participant's parents will be provided with supplies (including a thermometer, a transparent plastic tool to assess size of injection site redness and swelling, and a Memory Aid); instructed in their use; and provided with written materials, follow-up visit information and contact telephone numbers for study staff. Participants' parents are to be instructed to contact study staff for any reaction greater than mild. Participants are to be assessed in clinic for any local site reaction greater than mild.

5.2.7.2. Day 3, 31 and 59 (to be defined by 3 days after most recent study injection day) – Clinic visit follow-up

On the third day after each study injection, the participants will return to the study clinic for the following procedures:

a) Study staff will review and record the participant's interval health history, medication use and the parent's assessment of the post-injection experience through personal interview, assisted by the Memory Aid (See Appendix III).

b) Vital sign measurement, injection site and draining lymph node assessment and, if indicated, a symptom-directed physical examination will be performed.

c) Local and systemic reaction and AE review will be performed by the site PI (or designee). The highest axillary temperature written in the Memory Aid each day will be recorded in source document as peak temperature.

d) Findings of site PI or study staff that suggest inaccuracy of reported parental assessments will be clearly documented.

e) Any topics or new information considered by the site PI to be important to continued informed consent will be shared with the parent.

f) Study staff will instruct the parent regarding continued assessment of the child's health (and use of Memory Aid) and need to contact study staff (a) in follow-up of specific AEs, (b) if any injection site changes worsen or do not resolve, (c) if systemic symptoms worsen or do not resolve, and (d) if other AEs occur.

g) The next visit is scheduled.

5.2.7.3. Day 7, 35 and 63 ± 1 day (to be defined by 7 days after most recent study injection day) – Clinic visit follow-up

On the 7th day after each study injection (±1 day), the participant will return to the study clinic for the following procedures:

a) Study staff will review and record the participant's interval health history, medication use and the parent's assessment of the post-injection experience through personal interview, assisted by the Memory Aid (See Appendix III).

b) Vital sign measurement, injection site and draining lymph node assessment and, if indicated, a symptom-directed physical examination will be performed.

c) Local and systemic reaction and AE review will be performed by the site PI (or designee). The highest axillary temperature written in the Memory Aid each day will be recorded in source document as peak temperature.

d) Findings of site PI or study staff that suggest inaccuracy of reported self-assessments will be clearly documented.

e) Any topics or new information considered by site PI to be important to continued informed consent will be shared with the parent.

f) Blood samples will be obtained for safety monitoring laboratory tests on Day 7 for participants in Group C only (Appendix I).

g) Study staff will instruct the parent regarding continued assessment of the child's health (without a Memory Aid) and need to contact study staff (a) in follow-up of specific AEs, (b) if injection site changes worsen or do not resolve, (c) if systemic symptoms worsen or do not resolve, and (d) if other AEs occur.

h) The next visit is scheduled.

5.2.7.4. Day 28 (-2 to +7 days) (to be defined by 28 days after the previous study injection) – Study injection day

Every effort should be made to adhere to the protocol injection schedule windows. However, study injections may be given outside the specified window on a case-by-case basis, if approved by the Sponsor, provided that the injection will not have any adverse effect on the participant and that it is in the best interest of the scientific soundness of the research plan. The appropriate follow-up and subsequent injection schedule for each participant with delayed injections will be determined by the Sponsor.

Prior to Dose

The participant returns to the study clinic and the following procedures will occur.

a) Review of interval medical history, including AEs and medication use.

b) Vital signs measurement prior to dosing.

c) Review of inclusion/exclusion criteria to assure continued eligibility.

d) A full physical exam

e) Examination of the prior injection site and corresponding draining lymph nodes

f) Review of safety laboratory test results from Day 7 at the Day 28 visit before vaccination (and discussed with parents assuming there has not been occasion to do so earlier).

g) Examination (visually and by palpation) of the prospective injection site (anterolateral thigh), and inguinal lymph nodes

Study Injection

a) The injection site is observed before dose administration.

b) The prospective injection site will be cleaned with an alcohol swab and allowed to dry completely. The study injection will be administered into the outer aspect of the anterolateral thigh by inserting the needle (25 gauge, 25 mm) into muscle at 90°, holding the muscle mass stable with the non-injecting hand, drawing back to ensure that the needle is not in a blood vessel, and slowly depressing plunger. The needle is removed and the injection site gently rubbed with cotton wool.

Post Dose – Observation Period

a) Participants will remain at the site for at least 30 minutes post-injection.

b) After at least 30 minutes: Vital signs and the injection site will be assessed, redness and/or indurations will be measured and recorded. Any complaints or reactogenicity signs or symptoms will be assessed and documented.

c) The site PI (or designee) may determine that a participant requires further on-site observation; additional site or clinical assessments may be completed as needed.

d) When all study related procedures are complete and the site PI (or designee) determines that a participant's condition is acceptable, the participant will be discharged from the study clinic.

e) Before discharge, the participant's parents will be provided with supplies including a thermometer, ruler for measuring injection site redness and swelling, and a Memory Aid; instructed in their use; and provided with written materials, follow-up visit information and contact telephone numbers for study staff. Participants' parents are to be instructed to contact study staff for any reaction greater than mild. Participants are to be assessed in clinic for any local site reaction greater than mild.

5.2.7.5. Day 56 (-2 to +7 days) (to be defined by 28 days after the previous study injection) – Study injection day

Every effort should be made to adhere to the protocol injection schedule windows. However, study injections may be given outside the specified window on a case-by-case basis, if approved by the Sponsor, provided that the injection will not have any adverse effect on the participant and that it is in the best interest of the scientific soundness of the research plan. The appropriate follow-up and subsequent injection schedule for each participant with delayed injections will be determined by the Sponsor.

Prior to Dose

The participant returns to the study clinic and the following procedures will occur.

a) Review of interval medical history, including AEs and medication use.

b) Vital signs measurement prior to dosing.

c) Review of inclusion/exclusion criteria to assure continued eligibility.

d) A full physical exam

e) Blood samples will be obtained for immunological assays (see Appendix I).

f) Examination of the prior injection site and corresponding draining lymph nodes

g) Review of safety laboratory test results from Day 7 at the Day 28 visit before vaccination (and discussed with parents assuming there has not been occasion to do so earlier).

h) Examination (visually and by palpation) of the prospective injection site (anterolateral thigh), and inguinal lymph nodes

Study Injection

a) The injection site is observed before dose administration.

b) The prospective injection site will be cleaned with an alcohol swab and allowed to dry completely. The study injection will be administered into the outer aspect of the anterolateral thigh by inserting the needle (25 gauge, 25 mm) into muscle at 90°, holding the muscle mass stable with the non-injecting hand, drawing back to ensure that the needle is not in a blood vessel, and slowly depressing plunger. The needle is removed and the injection site gently rubbed with cotton wool.

Post Dose – Observation Period

a) Participants will remain at the site for at least 30 minutes post-injection.

b) After at least 30 minutes: Vital signs and the injection site will be assessed, redness and/or indurations will be measured and recorded. Any complaints or reactogenicity signs or symptoms will be assessed and documented.

c) The site PI (or designee) may determine that a participant requires further on-site observation; additional site or clinical assessments may be completed as needed.

d) When all study related procedures are complete and the site PI (or designee) determines that a participant's condition is acceptable, the participant will be discharged from the study clinic.

e) Before discharge, the participant's parents will be provided with supplies including a thermometer, ruler for measuring injection site redness and swelling, and a Memory Aid; instructed in their use; and provided with written materials, follow-up visit information and contact telephone numbers for study staff. Participants' parents are to be instructed to contact study staff for any reaction greater than mild. Participants are to be assessed in clinic for any local site reaction greater than mild.

5.2.7.6. Day 84 ± 4 days (to be defined by 28 days post-3rd Study injection day) – Clinic follow-up visit, immunogenicity sampling and first dose of Rotarix

On Day 84 (plus or minus 4 calendar days if necessary for participant compliance), the participant will return to study clinic and the following procedures occur:

a) Interval medical history will be obtained, including medication use.

b) Vital sign measurement, injection site and draining lymph node assessment and, if indicated, a symptom-directed physical examination will be performed.

c) AEs will be evaluated.

d) Blood samples will be obtained for immunological assays (see Appendix I).

e) The first dose of Rotarix will be administered.

f) Parents will be provided an empty container to obtain stool sample on the morning of Day 89 (RMPRU only).

g) The participant's parent will be reminded to contact the study clinic with any new information about chronic illnesses, serious health events, and/or hospitalizations.

h) The next clinic visit is scheduled.

5.2.7.7. Day 89 – Clinic visit for stool sampling (RMPRU only)

The parent will return to the clinic with a stool sample obtained that morning. If the parent is unable to bring the specimen to the clinic, a clinic staff member will visit the home to collect the sample. If the infant does not pass stool during the morning, a specimen will be obtained by rectal swab. Parents will be provided an empty container to obtain stool sample on the morning of Day 91.

5.2.7.8. Day 91 – Clinic visit for stool sampling (RMPRU only)

The parent will return to the clinic with a stool sample obtained that morning. If the parent is unable to bring the specimen to the clinic, a clinic staff member will visit the home to collect the sample. If the infant does not pass stool during the morning, a specimen will be obtained by rectal swab. Parents will be provided an empty container to obtain stool sample on the morning of Day 93.

5.2.7.9. Day 93 – Clinic visit for stool sampling (RMPRU only)

The parent will return to the clinic with a stool sample obtained that morning. If the parent is unable to bring the specimen to the clinic, a clinic staff member will visit the home to collect the sample. If the infant does not pass stool during the morning, a specimen will be obtained by rectal swab. The next clinic visit is scheduled.

5.2.7.10. Day 112 and 140 + 14 days (to be defined by 28 days after the previous Rotarix vaccination) – Clinic visit for second and third dose of Rotarix

On Day 112 and Day 140 (up to plus 14 calendar days), the participant will return for the second and third doses of Rotarix, and the following procedures will occur:

a) Interval medical history will be obtained, including medication use.
b) Vital signs will be measured.
c) AEs will be evaluated and recorded.
d) A targeted physical exam will be performed, if indicated.

The participant's parent will be reminded to contact the study clinic with any new information about chronic illnesses, serious health events, and/or hospitalizations, and the Day 224 follow-up will be scheduled.

5.2.7.11. Day 224 ± 14 days (to be defined by 168 days post-3rd injection day) – Final contact (telephone or clinic visit)

On Day 224 (plus or minus 14 calendar days if necessary for participant compliance), the participant's parents will be contacted by telephone or return to study clinic with the participant for a final follow-up visit and the following procedures will occur:

a) Interval medical history will be obtained for SAEs and other major or new chronic medical conditions.
b) A targeted physical exam will be performed, if indicated (request to come to clinic for those being contacted by telephone).

5.3. Interim Contacts and Unscheduled Visits

Interim contacts and visits (those between regularly scheduled follow up visits) may be performed at participants'/parents' request, or as deemed necessary by the site PI or designee for diagnosis and/or management of a finding or AE, at any time during the study. All interim contacts and visits will be documented in participants' study records and on applicable case report forms.

5.4. Stool Samples from Infants with Diarrhea

Parents of infants are to be encouraged to contact study staff if infants develop diarrhea. Should diarrhea be reported (defined as ≥3 looser than normal stools in a 24 hour period), a single stool sample is to be obtained to test for rotavirus, as early as possible after onset, but no later than 5 days after onset.

5.5. Early Termination

If a participant is withdrawn from the study for any reason prior to the planned final visit, every attempt is made to perform the following.

a) Review AEs by site PI (or designee).
b) Review of Memory Aid information to assess reactogenicity, if in use since the last visit.
c) Physical examination, including vital signs and examination of the injection site(s).
d) Safety laboratory testing if withdrawal occurs prior to scheduled safety laboratory testing.

5.6. Procedure Methods

Study procedures are performed and recorded in source documents as outlined in the Schedule of Events (Appendix I) and according to the following subsections.

5.6.1. Vital Signs

- Temperature in degrees Celsius (recorded to the nearest 0.1 degree) will be measured by oral thermometer for adults and axillary thermometer for toddlers and infants.
- Respiratory rate in breaths per minute.
- Heart rate in beats per minute will be measured by automated device or manually.

5.6.2. Height/length and Weight

- Height/length is measured in cm and recorded to the nearest 0.1 cm.
- Weight is measured in kg and recorded to the nearest 0.1 kg.

5.6.3. Physical Examination

Full physical examination will include assessment of vital signs, head, eyes, ears, nose, oropharynx, neck, chest (auscultation), lymph nodes (neck, supraclavicular, axillary, inguinal), abdomen (auscultation and palpation), genitourinary (for toddlers and infants), musculoskeletal, skin (especially injection sites), and neurological.

5.6.4. Medical History

A comprehensive medical history will be collected including details of any previous vaccinations (toddlers and infants – for adults, in the past 30 days) and reaction to vaccinations, birth (toddlers and infants), participation in clinical trials, surgery, previous hospitalization, allergy to food/drugs, current medication and history of any chronic or recurrent medical conditions.

An interval medical history will consist of inquiring regarding changes since the last medical history discussion (healthcare events, signs, symptoms and changes in use of prescription or nonprescription drugs or herbal preparations).

5.6.5. Injection Site Examination

Injection site assessment will be done by trained study personnel. Local reactions will be graded according the toxicity grading scale in Appendix II.

- Erythema/redness will be examined under standardized lighting conditions and measured. Severity of the reaction will be determined by staff on the basis of the severity grading table (Appendix II).
- Swelling/induration will be examined by palpation and visual inspection under standardized lighting conditions; the examiner may temporarily mark skin at margins of visible swelling/induration, then measure maximum diameter (as per Appendix I and as above).
- Pain/tenderness will be assessed by (a) observing signs of discomfort by gentle palpation of the injection site and (b) inquiring whether the parents have noted discomfort.
- Draining lymph nodes will be palpated for the presence of lymphadenopathy.

5.6.6. Reactogenicity Assessment

The following parameters for reactogenicity will be assessed during the 7 days after each study injection.

- Local reactogenicity (adults, toddlers and infants)
    - Pain
    - Tenderness
    - Erythema
    - Induration
    - Pruritus
- Systemic reactogenicity
    - Adults
        - Fever
        - Headache
        - Vomiting
        - Nausea
        - Fatigue
        - Chills
        - Myalgia
    - Toddlers and infants
        - Fever
        - Vomiting
        - Irritability
        - Decreased activity
        - Decreased appetite

5.6.7. Clinical Laboratory Testing

- Laboratory evaluations for screening (hematology, chemistry, testing for HIV) and safety monitoring (hematology and chemistry) are outlined in Appendix I, Schedule of Events.

5.6.8. Withdrawal from Further Study Injection and Early Termination from Study

An enrolled/vaccinated participant may be terminated from the study for any of these reasons.

a) Participant or parent withdraws consent for any reason.

b) Site PI, Emmes medical monitor, Safety Review Committee (SRC), Data Safety Monitoring Board (DSMB) or PATH medical officer decides that termination is in the best interest of the participant.

c) Site PI, Emmes medical monitor, SRC, DSMB or PATH medical officer decides that termination is necessary to protect the integrity of the study or achieve the objectives of the study.

d) Interruption of study schedule makes the participant's data unusable according to protocol requirements.

A participant may be withdrawn from receiving further study injections, but participants/parents will be encouraged to complete the safety-related follow-up. If the participant/parent agrees, other study procedures (e.g., blood sampling for measuring levels of antibodies) may be continued. Withdrawal from further study injection may occur based on the local investigator decision at the time of the event or based on the SRC review of these events on a cumulative basis if:

(a) Participant experiences objective local reactogenicity (erythema/induration) of Grade 2 or higher.

(b) Participant experiences an event that contributes to study stopping rules (see Section 9.4).

The final decision on withdrawing a participant from further study injections due to a and b will be made once the SRC reviews all the available data.

(c) For any other safety-related reasons at the discretion of the site PI, Emmes medical monitor, SRC, PATH medical officer, or the participant/parent.

5.6.9. Interim Contacts and Visits

Interim contacts and visits (those between regularly scheduled follow up visits) may be performed at participants'/parents' request, or as deemed necessary by the site PI or designee, at any time during the study. All interim contacts and visits will be documented in participants' study records and on applicable case report forms.

5.6.10. Contraception and Pregnancy

Contraception status is assessed and documented prior to enrollment and each study injection for female participants who are of childbearing potential. Prior to enrollment and at each injection visit, staff will ask volunteers to verbally confirm their use of adequate contraception methods if they are able to become pregnant. Adequate methods of contraception include barrier contraception, hormonal birth control, IUD, or surgical sterility; abstinence is an acceptable form of birth control. If a female participant becomes pregnant following randomization, study injections will be discontinued and she will be encouraged to complete remaining visits and study procedures unless medically contraindicated. Any participant who becomes pregnant during the period between first injection and visit Day 84 will continue to be followed for pregnancy outcome.

6. LABORATORY EVALUATIONS

Blood samples will be obtained to examine vaccine safety and immunogenicity.

6.1. Sample collection, distribution and storage

Samples to evaluate vaccine safety will be obtained and processed at the clinical trial site and transported to each site's designated laboratory for clinical testing. Research specimens collected for the immunogenicity time points will be separated into aliquots by per study specific process and stored at -20° C before being shipped to the Laboratory for Specialized Clinical Studies in the Division of Infectious Diseases at Cincinnati Children's Hospital Medical Center. Research specimens may be retained for elective analysis at the discretion of PVS. Samples will be stored properly in controlled-temperature refrigerators/freezers. Backup generators are available for proper sample storage.

Stool samples obtained from infants will be processed at the clinical trial site and transported to the laboratory at the National Institute for Communicable Disease, Sandringham, Johannesburg. Samples will be stored at the clinical trial sites and transported to the National Institute for Communicable Diseases (NICD) at 2-8° C. Following testing, residual specimens will be stored at NICD at -20° C.

6.2. Safety clinical laboratory assays

Protocol mandated screening and clinical safety laboratory tests will be conducted in real time. Refer to the Schedule of Events (Appendix I). The clinical laboratory for each site maintains proper accreditation and subscribes to a proficiency testing program.

Laboratory results will be reviewed promptly by the site PI or designee. Participants/parents will be notified of any clinically significant abnormalities. If clinically significant abnormalities are identified during screening, participants will be referred to their primary health provider or appropriate medical center. If identified during the study, participants may be asked to return to the study clinic for further evaluation, including clinical evaluation and repeat or additional laboratory testing, as warranted.

At the discretion of PVS, GCLP audits may be conducted to ascertain adequate processing of immunogenicity specimens.

6.3. Immunological assays

The immunological assays to be performed include:

- Antibody (IgG & IgA by ELISA) to P2-VP8 analysis on sera from the screening visit and Days 56, 84 and 140 in the infant groups, the screening visit and Day 28 in the toddler group, and the screening visit and 28 days after each study injection in the adult group. Magnitude (GMTs) of the ELISA IgG and IgA titers will be determined in serum samples for each of these time points; frequency of seroresponse rate will be determined for each post-study injection time point. Magnitude (GMTs) of the ELISA IgG and IgA will be determined 28 days after the second dose of Rotarix in the infant cohort (Day 140).
- Rotavirus neutralization analysis on sera from the screening visit and Days 56, 84 and 140 in the infant groups, the screening visit and Day 28 in the toddler group, and the screening visit and 28 days after each study injection in the adult group. Neutralization assays against the strains from which the vaccine was based will be conducted. Neutralization against several divergent rotavirus strains will be assessed if homologous neutralization is detected.

- Anti-rotavirus IgA (whole viral lysate) by ELISA will be determined in serum samples collected at baseline and one month after the final study injection.
- Additional immunogenicity assays may be performed by research laboratories to further evaluate post-study injection immune responses and to explore whether correlates of protection may be identified.

6.4. Assay qualification, standardization and validation

Serum anti-rotavirus IgA by ELISA (to whole viral lysate) and neutralizing antibody responses will be assessed in validated assays. Anti-P2-VP8 IgA and IgG by ELISA will be assessed in qualified assays.

6.5. Stool testing for rotavirus

Diagnosis of rotavirus infection in infants experiencing diarrhea during the study will be determined by stool detection of rotavirus using a validated antigen detection assay by ELISA at NICD. ELISA positive specimens will be confirmed and genotyped by PCR amplification of the VP7 and VP4 genes with a panel of G- and P-specific primers, respectively (WHO Manual of rotavirus detection and characterization methods, Methods 14 and 15 with minor modifications). The primer sets include: G-specific primer set (Beg9, End9, aAT8v, aBT1, aCT2, aDT4, mG3, mG9, mG10, mG12b and EndA) for the detection of G1, G2, G3, G4, G8, G9, G10 and G12 rotavirus strains and P-specific primer set (con2, con3, 1T-1v, 2T-1, 3T-1, 4T-1, 5T-1, mP11, p4943 and VP4F) for the detection of P[4], P[6], P[8], P[9], P[10], P[11] and P[14] rotavirus strains. Where the genotype cannot be obtained using the G or P-specific primers, sequencing of the VP7 or VP4 genes will be performed.

6.6. Rotarix shedding

At one study site, RMPRU, stool samples will be obtained 5, 7 and 9 days following the first dose of Rotarix for qualitative assessment of vaccine virus shedding at NICD.

All stool samples will be tested for the presence of rotavirus using the commercially available ProsPecT™ Rotavirus Microplate Assay (Oxoid Ltd, Ely, United Kingdom), according to the manufacturer's instructions.

ELISA positive specimens may be confirmed and genotyped by PCR amplification of the VP7 and VP4 genes with a panel of G- and P-specific primers, respectively (WHO Manual of rotavirus detection and characterization methods, Methods 14 and 15 with minor modifications). The primer sets include: G-specific primer set (Beg9, End9, aAT8v, aBT1, aCT2, aDT4, mG3, mG9, mG10, mG12b and EndA) for the detection of G1, G2, G3, G4, G8, G9, G10 and G12 rotavirus strains and P-specific primer set (con2, con3, 1T-1v, 2T-1, 3T-1, 4T-1, 5T-1, mP11, p4943 and VP4F) for the detection of P[4], P[6], P[8], P[9], P[10], P[11] and P[14] rotavirus strains. Genotype G1 and/or P[8] rotavirus strains will be sequenced to determine if the strain is vaccine derived or a wild-type case. The VP7 (1062bp) and VP4 (876 bp) genes amplified by PCR will be purified and sequenced using the BigDye® Terminator v 3.1 cycle sequencing kits (Applied Biosystems). Reactions will be run on a 3500 Genetic Analyzer (Applied Biosystems) and data analyzed with manufacturer supplied software. Sequences will be directly compared to the VP7 and VP4 genes of the Rotarix strains (sequences available on Genbank; JN849114.1 VP7 and JN849113.1 VP4) and submitted to GenBank for BLASTn analyses to establish whether the VP7 and VP4 genes are from the vaccine or from wild-type G1P[8] strains.

6.7. Biohazard Containment

As transmission of blood-borne pathogens can occur through contact with contaminated needles, blood, and blood products, appropriate blood and secretion precautions will be employed by all personnel in the drawing of blood and processing of blood, processing of stool, and shipping and handling of all specimens for this study. All biological specimens will be transported using packaging mandated by 42 CFR Part 72. All dangerous goods materials, including diagnostic specimens and infectious substances, must be transported according to instructions detailed in the International Air Transport Association (IATA) Dangerous Goods Regulations.

Biohazardous waste will be contained according to institutional, transportation/carrier, and all other applicable regulations.

7. STATISTICAL DESIGN AND ANALYSIS

7.1. Overview and General Design

This study is a Phase 1/2 randomized, double-blind, dose-escalation, descending age clinical trial to assess the safety and immunogenicity of the trivalent rotavirus P2-VP8 subunit rotavirus vaccine (adsorbed onto aluminum hydroxide [$Al(OH)_3$]) as compared to a placebo (NS). The primary objectives are to evaluate the safety, tolerability and immunogenicity of the P2-VP8 subunit rotavirus vaccine with $Al(OH)_3$ in infants. Immunogenicity is to be assessed through detection of vaccine-specific anti-P2-VP8 IgG and IgA antibodies by ELISA (to the P[4], P[6] and P[8] antigens in the vaccine) and neutralization of both homologous (the three strains from which the vaccine antigens are derived) and heterologous rotavirus strains.

The primary hypothesis of safety will be fulfilled if no participants develop a vaccine related SAE and the vaccine is well-tolerated.

The second hypothesis of immunogenicity will be fulfilled if at least 60% of the infants in at least one dose level demonstrate neutralizing responses to at least two of three strains from which the vaccine antigens are derived.

A detailed statistical analysis plan for preparation of the final study report will be created and made final prior to database lock and unblinding for each age group. All statistical analyses will be performed using SAS® software Version 9.2 or later.

Medical history and AEs will be coded using MedDRA dictionary Version 16.1 or later. The frequency count and percentage of participants will be summarized according to the coded terms of system organ class and preferred term. Participant-wise data listings will be provided.

7.2. Randomization and blinding procedures

The randomization scheme will be generated and maintained by the Statistical and Data Management Group (SDMG) at The Emmes Corporation, Rockville, MD. Participants will be enrolled into the study cohorts online and randomized using the enrollment module of The Emmes Corporation's AdvantageEDC℠ electronic data capture system. Each participant enrolled into the trial will be assigned a treatment code based on the cohort he/she is enrolled into after demographic and eligibility data have been entered into the system.

The site pharmacists with primary responsibility for dispensing study products are charged with maintaining security of the treatment assignments.

7.3. Objectives and Endpoints

7.3.1. Primary Objectives:

Safety

To evaluate the safety and tolerability of the trivalent P2-VP8 subunit rotavirus vaccine at escalating dose levels in healthy South African adults, toddlers and infants

Endpoints:

- Number of SAEs through 28 days of last study injection
- Number of AEs through 28 days of last study injection
  - AEs within four weeks after each injection. AEs will be categorized by MedDRA SOC and MedDRA PT and analyzed by study cohort, severity, duration and relationship to vaccine.
    - Maximum severity per participant
    - Proportion of AEs related to vaccine
- Number of Grade 2 or greater local reactogenicity events (injection site pain/ tenderness, redness, swelling, itching, local lymphadenopathy) within 1 week (Day 0 to Day 7), after each injection and for the three combined injections.
- Number of Grade 2 or greater systemic reactogenicity (fever, vomiting, nausea, fatigue, chills and myalgia for adults; fever, vomiting, irritability, decreased activity, and decreased appetite for toddlers and infants) per cohort and product received, within 1 week (Day 0 to Day 7) after each injection and for the three combined injections and maximum severity per participant.

Immunogenicity

To evaluate the immunogenicity of three doses of the trivalent P2-VP8 subunit rotavirus vaccine at different dose levels in healthy South African infants

Endpoints:

- Proportion of infants with anti-P2-VP8 IgG & IgA seroresponses by ELISA (four-fold increase in antibody titers between baseline and 4-weeks post-third study injection) in assays using each of the three vaccine antigens (P[4], P[6] and P[8])
- Proportion of infants with neutralizing antibody responses (2.7-fold increases in antibody titers between baseline and 4-weeks post-third study injection) to each of the three rotavirus strains from which the vaccine antigens are derived, as well as heterologous strains
- Anti-P2-VP8 IgG & IgA geometric mean titers (GMT) (baseline and 4 weeks post third study injection) in ELISA assays using each of the three vaccine antigens (P[4], P[6] and P[8])
- Neutralizing antibody GMT (baseline and 4 weeks post-third study injection) to each of the three rotavirus strains from which the vaccine antigens are derived, as well as heterologous strains
- Proportion of infants with neutralizing antibody responses to at least two of the three strains from which the vaccine antigens are derived

7.3.2. Secondary Objectives

Safety

To evaluate the longer term safety (through 6 months after the last vaccination) of the trivalent P2-VP8 subunit rotavirus vaccine at escalating dose levels in healthy South African adults, toddlers and infants

Endpoints:

- Number of SAEs at any time during the study
- Number of AEs at any time during the study

Immunogenicity

To evaluate the immunogenicity of two doses of the trivalent P2-VP8 subunit rotavirus vaccine at different dose levels in healthy South African infants

Endpoints:

- Proportion of infants with anti-P2-VP8 IgG & IgA seroresponses by ELISA (four-fold increase in antibody titers between baseline and 4 weeks post-second study injection) in assays using each of the three vaccine antigens (P[4], P[6] and P[8])
- Proportion of infants with neutralizing antibody responses (2.7-fold increases in antibody titers between baseline and 4 weeks post-second study injection) to each of the three rotavirus strains from which the vaccine antigens are derived, as well as heterologous strains
- Anti-P2-VP8 IgG & IgA geometric mean titers (GMT) (baseline and 4 weeks post-second study injection) in ELISA assays using each of the three vaccine antigens (P[4], P[6] and P[8])
- Neutralizing antibody GMT (baseline and 4 weeks post-second study injection) to each of the three rotavirus strains from which the vaccine antigens are derived, as well as heterologous strains
- Proportion of infants with neutralizing antibody responses to at least two of the three strains from which the vaccine antigens are derived (4 weeks post-second study injection)

To evaluate the immunogenicity of the trivalent P2-VP8 subunit rotavirus vaccine at different dose levels in healthy South African adults and toddlers

Endpoints:

- Proportion of adults and toddlers with anti-P2-VP8 IgG & IgA seroresponses by ELISA (four-fold increase in antibody titers between baseline and 4-weeks post-final study injection) in assays using each of the three vaccine antigens (P[4], P[6] and P[8])
- Proportion of adults and toddlers with neutralizing antibody responses (2.7-fold increases in antibody titers between baseline and 4-weeks post-final study injection) to each of the three rotavirus strains from which the vaccine antigens are derived, as well as heterologous strains
- Anti-P2-VP8 IgG & IgA geometric mean titers (GMT) (baseline and 4 weeks post final study injection) in ELISA assays using each of the three vaccine antigens (P[4], P[6] and P[8])

- Neutralizing antibody GMT (baseline and 4 weeks post-final study injection) to each of the three rotavirus strains from which the vaccine antigens are derived, as well as heterologous strains
- Proportion of adults and toddlers with neutralizing antibody responses to at least two of the three strains from which the vaccine antigens are derived

7.3.3. Exploratory Objective

Efficacy

To evaluate the impact of the trivalent P2-VP8 subunit rotavirus vaccine on shedding of Rotarix administered following P2-VP8 subunit rotavirus vaccination of healthy South African infants as a test of concept

Endpoint:

- Proportion of participants with Rotarix vaccine virus detected in stools on Days 5-9 following the first dose of Rotarix, which is to be administered 28 days after the third study injection

7.4. Sample Size

7.4.1. Primary Safety Endpoints

Safety will be assessed by analyses of the following primary endpoints (events), where the unit of analysis in each case will be the proportion of participants with at least one event:

- Moderate or greater solicited local reactions
- Moderate or greater solicited systemic reactions
- Moderate or greater adverse events
- Moderate or greater adverse events where there is a reasonable possibility that the study product caused the event, i.e., are suspected adverse reactions
- Serious adverse events

It is assumed that almost all participants enrolled will provide data for safety analysis and at least 90% of enrolled participants will be evaluable for immunogenicity assessments.

Safety

For Cohorts A and B, 12 vaccine recipients per dose group and 24 vaccine recipients for the two dose groups combined will provide a greater than 90% chance of observing an AE that has a 17.5% and 9.2% rate of occurrence, respectively. For the two infant cohorts combined (C and D), with 150 vaccine recipients per dose group and 450 vaccine recipients for the three dose groups combined will provide a greater than 90% chance of observing an AE that has a 1.6% and 0.5% rate of occurrence, respectively.

Conversely, if no SAEs are observed in 24 and 450 vaccine recipients, the study will be able to rule out SAEs occurring at a rate of approximately 11.7% and 0.7%, respectively based on the upper bounds of the one-sided 95% confidence.

Table 25: Probabilities of observing AEs

Cohort		Sample Size	Underlying Event Rate	Probability to observe an AE
A (Adults) OR B (toddlers)	Each Dose Group	12	1.0%	11%
			2.0%	22%
			5.0%	46%
			17.5%	90%
	Both Doses Combined	24	1.0%	21%
			2.0%	38%
			5.0%	71%
			6.2%	90%
C+D (Infants)	Each Dose Group	150	1.0%	78%
			1.6%	91%
			2.0%	95%
			5.0%	>99%
	All 3 Doses Combined	450	0.2%	59%
			0.3%	74%
			0.4%	84%
			2.0%	90%

7.4.2. Primary Immunogenicity Endpoints

Based on the results in South African infants who received monovalent P2-VP8 vaccine or placebo (VAC-013), the strain specific seroresponse rates for both P2-VP8 vaccine doses are expected to be ≥80% for at least one of the three P2-VP8 vaccine doses and <20% for the placebo group. For the two infant cohorts combined with 135 evaluable vaccine recipients per dose level (assuming 10% loss), this study is designed to provide at least 74% and 95% power (Fisher's exact test) to detect 15 and 20 percentage points difference (e.g. 65% vs. 80 and 60% vs. 80%%), respectively, in seroresponse rates between any two P2-VP8 dose groups. For comparisons between a P2-VP8 dose group and the combined placebo groups, this study is designed to provide ≥99% power to detect ≥30 percentage point difference (e.g. 50% vs. 20%).

For the sample calculations of the Geometric Mean Titer (GMT) endpoint based on the (VAC 013 study, the LOG10 standard deviations were estimated to be <0.6 (range: 0.24 – 0.56) for the P2-VP8 doses and the placebo group. Using a conservative LOG10 standard deviation of 0.6, this study with 135 evaluable infants per group (assuming 10% loss) is designed to provide at least 98% power to detect as low as a 2.0-fold difference between any two P2-VP8 dose groups or between any P2-VP8 vaccine dose (15 µg, 30 µg or 90 µg dose) and the placebo group.

Table 26: Power calculations for Comparing GMT in Two Groups of Infants (Cohorts C and D combined)

LOG10 Standard Deviation	Difference to detect	Power**
0.3	1.5-fold	>99%
	2-fold	>99%
	4-fold	>99%
0.4	1.5-fold	95%
	2-fold	>99%
	4-fold	>99%
0.5	1.5-fold	82%
	2-fold	>99%
	4-fold	>99%
0.6	1.5-fold	67%
	2-fold	98%
	4-fold	100%

* Between two P2-VP8 dose groups or between a P2-VP8 vaccine dose and the placebo group

** Two-Sample T-Test Assuming Equal Variance using PASS 13.

7.5. Analytical Methodology

The different populations to be used in data analysis are the following:

- The per-protocol population (PP), which is defined as all randomized participants who adhere to the protocol, complete all their scheduled visits, and present no major protocol violations, as defined prior to database lock. A “major” violation is defined as a protocol deviation that is considered to have an impact on the immunogenicity results of the study. Volunteers are analyzed according to randomized treatment arm.
- The safety population, which is defined as all participants who are randomize and receive at least one injection of the investigational product. Volunteers are analyzed according to treatment received rather than randomized treatment arm.

The primary objectives of the study are to assess the safety, tolerability and immunogenicity of the study vaccine. All safety data (solicited local and systemic reactions and unsolicited AEs and SAEs) collected after participants are exposed to the study product will be included in the primary analysis of safety.

To assess safety, the number and percentage of participants experiencing at least one AE, and the number and percentage of participants experiencing each specific AE, categorized by body system and preferred term, will be tabulated by study cohort and product received along with their corresponding 95% confidence intervals. Overall summaries by cohort and by product received include the number and percentage of participants experiencing: (1) any SAE; (2) any adverse experience; (3) any Grade 2 or greater AE; (4) any AE judged related to study product; (5) any Grade 2 or greater AE judged related to study product. In addition, AEs will be summarized by grade.

To assess tolerability, the number and percentage of participants with Grade 2 or higher local or systemic reactions will be tabulated by study cohort and by product received along with their corresponding 95% confidence intervals. Local and systemic reactions will be tabulated separately. Overall summaries by cohort and by product received will include the number and percentage of participants experiencing: (1) each specific reaction at Grade 2 or greater after each injection; (2) each specific reaction at Grade 2 or greater after any injection; (3) any reaction at Grade 2 or greater after each injection; and (4) any reaction at Grade 2 or greater after any injection. Descriptive statistics will be used to summarize the duration of local and systemic reactions. In addition, local and systemic reactions will be summarized by grade.

Changes in clinical laboratory values between baseline and one week after vaccination will be analyzed descriptively.

Immunogenicity analysis will compare the rate of responses and geometric mean titers (GMTs) between the various treatment groups based on injections received per protocol. To adjust for the decay in maternal antibody occurring concurrently with the IgG and neutralizing antibody immune responses to the vaccine, an analysis of the adjusted seroresponse rates utilizing the exponential decay function based on the estimated half-life of the maternal antibody will be performed in addition to the analysis based on the unadjusted raw values. For each assay, an estimated maternal antibody half-life will be derived from linear regression of LOG transformed titers in all infant placebo recipients, combined. Multiple components within each assay will be pooled (e.g. categorical class variable in the regression analysis) for the regression analysis to obtain a single half-life estimate per assay. Any placebo recipient with suspected community acquired rotavirus infection as identified by having a higher post vaccination titer higher than that at baseline, as well as those with values below the detectable limit of the assay at baseline will be excluded from the regression analysis.

The following formula will be used to calculate the adjusted fold-rise and titer:

frma = 2^[(LOG2(titerx / titer0) + LOG2(2x/MH))

titerma = 2^[LOG2(titerx) + LOG2(2x/MH)]

where frma = maternal antibody adjusted fold-rise

titerma = maternal antibody adjusted post-vaccination titer

titerx = titer at Day x post Dose One

titer0 = titer at pre-Dose One

x = number of days post Dose One

MH = maternal half-life in LOG2 scale

For each immune assay, the proportion of participants with a positive response at a specific time point or with any positive response at any time point will be evaluated using the 2-sided exact binomial (Clopper-Pearson) 95% CI for each treatment group and compared between 2 treatment groups (i.e., between vaccine dose groups or between vaccine and placebo groups) using Fisher's exact test. For continuous variables (i.e., titer/level of response), 2-sided 95% CI for the GMT for each treatment group using a t-distribution on log transformed titers/levels and 2-sample t-test on log transformed titers/levels will be used to compare between 2 treatment groups (i.e., between vaccine dose groups or between vaccine and placebo groups). A non-parametric method (e.g., bootstrap or Wilcoxon rank sum test) will be used for continuous variables if the data are not consistent with the normal distribution.

Assessment of shedding of Rotarix virus will be performed for each of the three specified post-vaccination days and for shedding on any of the three days. Rates of shedding between the placebo and (1) each dose group and (2) the combined three dose groups will be compared using the Fisher's exact test.

When the use of descriptive statistics to assess group characteristics or differences is required, the following methods may be used: for categorical variables, the number and percent of participants in each category; for continuous variables, the mean, median, standard deviation, quartiles and range (minimum, maximum). Within-group assessment of the change from the baseline measurement to a follow-up measurement will be analyzed using McNemar's test (for categorical response variables) or the paired t-test or Wilcoxon signed-rank test (for continuous variables). The following methods will be used for formal testing of differences between the study vaccine and placebo: for binomial response variables, Fisher's exact test and/or logistic regression; for continuous variables, analysis of variance and/or Student's t-test. The specific summary statistics and methods to be used for each endpoint will be described in the Statistical Analysis Plan.

All testing and inference will be performed with 2-sided p-value of 0.05. For each immunogenicity endpoint, a multiplicity adjustment for 4 treatment groups will be applied using a sequential testing approach by performing an overall comparison of all treatment groups prior to pairwise comparisons. No multiplicity adjustment for multiple immunogenicity endpoints is planned, since the dose selection for the Phase 3 will be based on comprehensive statistical and clinical evaluation of all immunogenicity endpoints including safety results.

8. SAFETY ASSESSMENT AND REPORTING

This section defines the types of safety events that should be reported and outlines the procedures for appropriately collecting, grading, recording and reporting them.

8.1. Safety Events

All safety events observed under this protocol will be reported through the AdvantageEDC data system throughout the study. Information about vaccine reactogenicity (during the 7 days following vaccination) are collected on study-specific forms and are not captured on an Adverse Event form. Reactogenicity data (solicited signs or symptoms) will be collected through visit Day 7 post-study injection; if a solicited sign or symptom has started during the 7 days post study injection and continues beyond Day 7 it will continue to be reported as a reactogenicity symptom. Any symptom starting after 7 days post any study injection will be recorded as an AE. Only when a solicited sign or symptom is considered an SAE, as defined below, will it be reported on an AE/SAE form set, in addition to the reactogenicity form. All other safety events that meet the definition of an AE or SAE that occur throughout the study are reported on the AE/SAE form set. AE's will be collected through study visit Day 84 in Groups A, C and D, and through study Day 28 in Group B. SAEs and new major medical diagnoses will be assessed through Day 224 in Groups A, C and D, and through Day 168 in Group B, and will be reported on the AE/SAE form set.

8.2. Reporting Period

Safety events are reported from the time of the first study injection through completion of the study at 6 months after the final injection (as above). For participants who withdraw or are withdrawn from the study, an early termination visit should occur 5-7 days after the last injection or study visit to elicit occurrence of AEs (serious and non-serious).

8.3. Definitions

8.3.1. Adverse Event (AE) or Medical Event

An adverse event is any untoward medical occurrence in humans, whether or not considered related to study product, that occurs during the conduct of a clinical trial. Any change from baseline assessment of clinical status, ECGs, routine laboratory tests, x-rays, physical examinations, etc., that is considered clinically significant by the site PI is considered an AE.

Suspected adverse drug reaction is any AE for which there is a reasonable possibility that the study product caused the AE. A reasonable possibility implies that there is evidence that the study product caused the event.

Adverse reaction is any AE caused by the vaccine.

8.3.2. Serious Adverse Events (Defined as Serious Adverse Events, Serious Suspected Adverse Reactions or Serious Adverse Reactions)

An SAE, including a serious suspected adverse reaction or serious adverse reaction as determined by the site PI or the Sponsor, is any event that results in any of the following outcomes:

1. Death
2. Life-threatening AE (Life-threatening means that the study participant was, in the opinion of the site PI or Sponsor, at immediate risk of death from the event as it occurred.)
3. Inpatient hospitalization or prolongation of existing hospitalization
4. Persistent or significant incapacity or substantial disruption of the ability to conduct normal life functions
5. Congenital abnormality or birth defect
6. Important medical event that may not result in one of the above outcomes but may jeopardize the health of the study participant or require medical or surgical intervention to prevent one of the outcomes listed in the above definition of serious event

8.3.3. Unexpected Adverse Event

An AE is “unexpected” when its nature (specificity) or severity is not consistent with applicable product information, such as safety information provided in the Investigators’ Brochure (IB), the investigational plan or the protocol.

8.4. Toxicity Grading

The study clinic assigns toxicity grades to indicate the severity of adverse experiences and toxicities. The toxicity grading criteria provided in Appendix II grade AEs from Mild (grade 1) to Life Threatening (grade 4). All AEs leading to death are Grade 5 events. AEs are graded with the worst severity grade during the illness/symptoms. AE severity will be graded using the attached grading scale (Appendix II), which has been developed on the basis of *Division of AIDS (DAIDS) Table for Grading the Severity of Adult and Pediatric Adverse Events*, version 2.0, November 2014, of the US National Institutes of Health, with modifications to reflect local population norms. For laboratory values not included in Appendix II, grading will be based on the *Division of AIDS (DAIDS) Table for Grading the Severity of Adult and Pediatric Adverse Events*, version 2.0, November 2014, available at webpage:

http://rsc.tech-res.com/safetyandpharmacovigilance/gradingtables.aspx

8.4.1. Guidelines for Determining Causality of an Adverse Event

All AEs, and SAEs, will be assessed for a causal relationship with the study product by the site PI. Appropriate medical judgment should be used to determine the relationship, considering all relevant factors, including pattern of reaction, temporal sequence and relationship, biological plausibility, confounding factors such as concomitant medication, concomitant diseases and relevant history. Assessment of causal relationship should be recorded in the AE and in SAE forms.

The site PI should consider the following question when assessing causality of an AE to study product:

Is there a reasonable possibility that the product caused the event?

Reasonable possibility implies there is evidence that the study product caused the reported event. An affirmative answer designates the event as a suspected adverse reaction, and the AE is therefore considered “related.” If the answer is no, then the AE is considered “unrelated.”

8.5. Adverse Event Identification, Resolution and Reporting

To improve the quality and precision of acquired AE data, the site PI should observe the following guidelines:

- Whenever possible, use recognized medical terms when recording AEs on the AE CRF. Do not use colloquialisms and/or abbreviations.
- If known, record the diagnosis (i.e., disease or syndrome) rather than component signs, symptoms and laboratory values on the AE CRF (e.g., record congestive heart failure rather than dyspnea, rales, and cyanosis). However, signs and symptoms that are considered unrelated to an encountered syndrome or disease should be recorded as individual AEs on the CRF (e.g., if congestive heart failure and severe headache are observed at the same time, each event should be recorded as an individual AE).
- AEs occurring secondary to other events (e.g., sequelae) should be identified by the primary cause. A “primary” AE, if clearly identifiable, generally represents the most accurate clinical term to record on the AE CRF. If a primary serious AE (SAE) is recorded on an SAE CRF, events occurring secondary to the primary event should be described in the narrative description of the case.
- Grade 2 or higher abnormal laboratory test results will be entered as AEs with the toxicity grade associated with the abnormal laboratory test and attribution of the abnormality relative to the study product. Grade 1 abnormal laboratory test results will be entered as AEs if the site PI determines them to be clinically significant.
- Death is an outcome of an event. The event that resulted in the death should be recorded and reported on the SAE CRF.
- For hospitalizations for surgical or diagnostic procedures, the illness leading to the surgical or diagnostic procedure should be recorded as the SAE, not the procedure itself. The procedure should be captured in the case narrative as part of the action taken in response to the illness.

AEs may be discovered through any of these methods.

- Observing the participant.
- Questioning the participant’s parent, which should be done in an objective manner.
- Receiving an unsolicited complaint from the participant’s parent.
- Review of medical records/source documents.

Each participant will have a scheduled observation following each study injection, including a symptom-directed physical examination, if indicated. Participants will be monitored in the clinic for at least 30 minutes after each study injection. On Days 0 to 7 after each study injection, participants and parents will be instructed to assess and record daily in a participant memory aid any signs and symptoms at the study injection site (injection site pain/tenderness, redness, swelling, itching), as well as systemic signs and symptoms (fever, headache, vomiting, nausea, fatigue, chills and myalgia for adults; and fever, vomiting, decreased appetite, irritability, and decreased activity for toddlers and infants). Follow-up visits will be conducted on post-study injection Days 3 and 7 in toddlers and infants and Day 7 in adults, including verification of memory aid notes and directed assessment. On Day 7 in Groups A, B and C, blood will be drawn for monitoring hematology and chemistry tests. Participants/parents will be instructed to call the study team if they observe significant injection site or systemic signs or symptoms, and then to continue monitoring and reporting their children's condition. All participants will be followed for safety for six months after the last injection, with follow-up by telephone or clinic visit.

8.5.1. AE Resolution

All reported AEs should be followed until resolution or stabilization, or until completion of the study. Participants who have an ongoing study product-related SAE at study completion or at discontinuation from the study will be followed by the site PI or designee until the event is resolved or determined to be irreversible, chronic, or stable by the site PI.

8.5.2. General Recording and Reporting Procedures

A multi-page AE/SAE form set will be used, allowing all AEs to be submitted through a single reporting mechanism. SAEs will require additional information reported on additional pages within the AdvantageEDC data system. As appropriate or per request of the Emmes medical monitor or PATH medical officer, source documents (e.g., hospitalization discharge summaries) may be uploaded to the AE/SAE form set, as well (with care taken to remove personal identifiers). The site PI will treat or refer, as appropriate, participants experiencing AEs and observe them at suitable intervals until their symptoms resolve or their status stabilizes.

8.5.3. SAE Recording and Reporting Procedures

SAEs will be recorded on the AE case report form (CRF). The site is obligated to report SAEs to the Emmes Coordinating Center within 24 hours of the site's knowledge of the event. The following attributes will be assigned by the site PI or assignment by designee will undergo documented review by the site PI:

- Description
- Date of onset and resolution (if known when reported)
- Severity
- Assessment of relatedness to test article
- Action taken

The site PI will apply clinical judgment to determine whether an AE is of sufficient severity to require discontinuation of administration of study injection for that participant. If necessary, the site PI will suspend any trial procedures and institute the necessary medical therapy to protect a participant from any immediate danger.

8.5.4. Site Reporting of Events

- Notify the site PI.
- Complete and transmit an AE Form through the AdvantageEDC data system. Information regarding a SAE report must be recorded in the participant's medical chart and entered in the AdvantageEDC data system.
- SAE reports should include hospital admittance notes, hospital discharge summary, clinical notes, resolution date, treatment, and any other pertinent information regarding the event. If not immediately available, reporting should not be delayed to provide these documents.
- In the event of a death, the SAE Form must be completed and transmitted along with other supporting data (e.g., death certificate, medical notes).

Figure 9: Reporting Decisions for Adverse Events

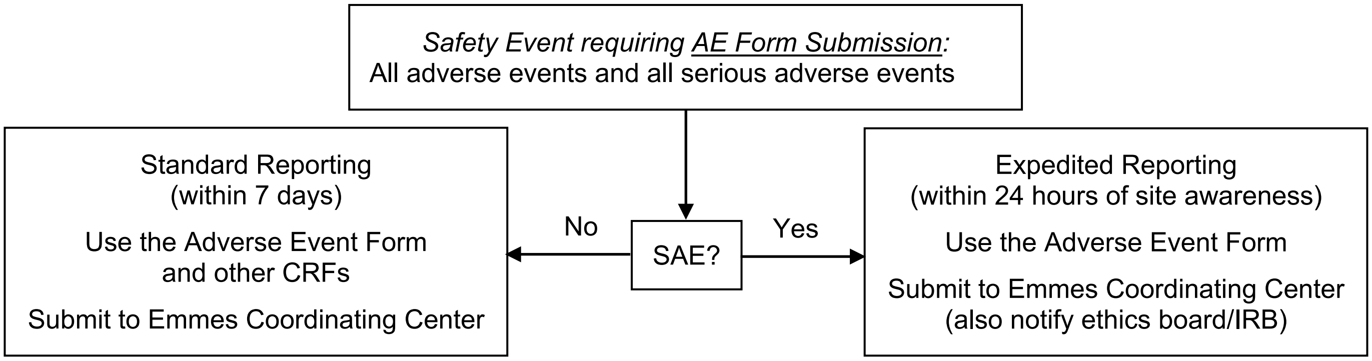

8.6. Serious Adverse Event Notification

8.6.1. Notifications and Review

The site PIs will provide the Emmes Coordinating Center with data for all SAEs, as defined per the protocol, on an ongoing basis by entering the information in AdvantageEDC. The Emmes Coordinating Center is responsible for notifying the Sponsor and the CRO performing site monitoring and will do so simultaneously with the reporting to the clinical database. Notification of reported events will be generated at the time of entry into the data system, and all SAEs will be reviewed promptly by the Emmes medical monitor within the data system. As noted above, this notification should be within 24 hours of site awareness of the event. Site personnel will be trained in reporting AEs and SAEs.

The Emmes medical monitor will review all unanticipated events involving risk to participants or others, SAEs and all participant deaths associated with the protocol and will provide a written report. At a minimum, the Emmes medical monitor must comment on the outcomes of the event or problem and, in case of an SAE or death, comment on the relationship to participation in the study. The Emmes medical monitor must also indicate whether he/she concurs with the details of the report provided by the site PI.

Summary review of all reported AEs and SAEs will be compiled on a weekly basis to identify any safety trends. Review of reactogenicity and safety monitoring laboratory tests will also be conducted on a weekly basis during the periods such monitoring is being performed.

8.6.2. Expedited Reporting

The Emmes Coordinating Center will be responsible for expedited Safety Reports and IND Annual reports to the US Food and Drug Administration (FDA). SCT Consulting will be responsible for reporting to the South African Medicines Control Council (MCC).

FDA Reporting

When expedited reports as defined below are required, the cover memorandum, MedWatch Form FDA 3500A, and any pertinent attachments will be processed by the Emmes medical monitor and a copy of the completed report will be submitted by fax or courier delivery before the regulatory reporting deadline, to the following persons:

- FDA medical officer as appropriate (submitted as an amendment to the applicable IND)
- Site PI (who is responsible for forwarding the report to the local EC/IRB)
- SRC independent medical monitors
- PATH medical officer

If relevant follow-up information becomes available, the Emmes medical monitor will be responsible for obtaining and reviewing details from the site. A follow-up MedWatch form will be completed and forwarded to all parties that received the earlier SAE report. A copy of the safety sections for annual FDA reports will be forwarded to PVS.

Suspected adverse reactions that are serious and unexpected will be reported to the FDA within 15 days, or for deaths and life threatening events that are both suspected adverse reactions and unexpected, within 7 days (per 21 CFR 312.32).

MCC Reporting

SCT Consulting will be responsible for reporting to the MCC.

All fatal and life-threatening, unexpected adverse drug reactions should be reported within 7 calendar days after first knowledge by the applicant. The initial notification must be followed by as complete a report as possible, within an additional 8 calendar days.

Serious, unexpected adverse drug reactions that are not fatal or life-threatening must be reported as soon as possible, and not later than 15 calendar days after first knowledge by the applicant.

The MCC must be notified, within 15 calendar days after first knowledge by the applicant, when there is a suggestion of a change in the nature, severity or frequency of expected adverse drug reactions or when new risk factors are identified. The basis on which these assessments are made should be included.

Any information, that may in any way influence the benefit-risk assessment of a medicine or that would be sufficient to consider changes in the administration of the medicine or in the overall conduct of a clinical trial, must be reported to the MCC. The applicant must submit this information to the MCC within three calendar days of first knowledge by the applicant.

Subsequent review by the MCC, FDA, the SRC, EC/IRB, or the Sponsor may suspend further study product administration or procedures. The study Sponsor, MCC, FDA and SRC retain the authority to suspend additional enrollment and treatments for the entire study as applicable.

8.6.3. Notifying the Protocol Safety Review Committee

The Emmes Coordinating Center will provide the SRC with listings of all SAEs on an ongoing basis. Furthermore, the SRC will be informed of expedited reports of SAEs.

8.6.4. Notifying the Ethics Committee/Institutional Review Board

The site PIs will ensure the timely dissemination of required SAE information, including expedited reports, to their respective local ethics committees in compliance with applicable local regulations and guidelines. The site PIs are responsible for submitting the safety report (initial and follow up SAE reports) or other safety information (e.g., revised IB) to their respective ethics committees and for retaining a copy in the site's study file.

WIRB Guidelines

All SAEs will be reported to WIRB by PVS according to WIRB guidelines and using the WIRB Ten Day Adverse Event Form.

WIRB Phone: 800-562-4789, Fax: 360-252-2498.

9. SAFETY MONITORING

Extensive safety monitoring will be provided for this protocol. The site PIs and/or designated site staff will be responsible for continuous close safety monitoring of all study participants and for alerting the Sponsor if unexpected concerns arise or stopping criteria are met.

9.1. Safety Review Committee

A SRC, comprised of the site PIs from each study site, the Sponsor medical monitor, the Emmes medical monitor and two independent local medical experts, will monitor safety throughout the duration of the study (in addition to the Data Safety Monitoring Board described in section 8.2). The responsibilities and procedures of the SRC will be defined in the SRC Charter. The study statistician with assistance of the data management staff will prepare safety reports as needed for SRC discussions. In addition to routine review of safety information, a central role of the SRC is the review of safety data for the recommendation of whether to progress to the next dose level in each age cohort and to the next age cohort. Cumulative safety data will be available continuously for review by SRC members, and safety reports will be prepared for each SRC deliberation of dose escalation (see Section 9.4, Dose Escalation) and progression to subsequent cohorts (adults to toddlers and toddlers to infants).

The SRC members will review the safety data throughout the study to determine whether pause criteria have been met. The SRC will be notified by the Emmes Coordinating Center and convene by conference call if pause criteria may have been met. The SRC may request review of unblinded safety data by the independent SRC members in deliberating whether to refer concerns and events to the DSMB. If pause criteria are met, the DSMB is to be notified and convened, as described in section 9.4.

The SRC may make recommendations regarding permanent discontinuation of administration of study product in individual participants based on careful review of all relevant data.

SRC reviews will be summarized with recommendations to the study Sponsor.

9.2. Data and Safety Monitoring Board (DSMB)

A DSMB formed by independent vaccine, pediatric and infectious disease experts, as well as a biostatistician, will be established to periodically review cumulative data, as needed. The responsibilities and procedures of the DSMB will be defined in the DSMB Charter. The DSMB will be responsible for safeguarding the interests of trial participants, assessing safety during the trial, and for monitoring the overall conduct of the clinical trial. The DSMB will provide

recommendations about continuing, modifying or stopping the trial. Items reviewed by the DSMB will include: study participant accrual and demographic information; interim/cumulative safety data; discontinuations of study injections; factors that might affect the study outcome or compromise the confidentiality of the trial data (such as treatment and endpoint unbinding); data quality, completeness, and timeliness; and factors external to the study, such as scientific or therapeutic developments that may impact participant safety or the ethics of the study.

The DSMB will convene prior to study initiation and then at least every six months. In addition to routinely scheduled calls, if the protocol team or SRC has serious safety concerns or study pause criteria are met, the DSMB will convene by teleconference to jointly review the data. The DSMB reviews will be summarized with recommendations to the study Sponsor as to whether there are safety concerns and whether the study should continue without change, be modified, or be terminated.

If at any time, a decision is made to permanently discontinue administration of study product in all participants, expeditious notification will be provided by PVS to the U.S. FDA (via Emmes Coordinating Center) and WIRB, to the MCC (via SCT Consulting) and by the site PIs to their respective local ECs.

9.3. Withdrawal for Safety

A participant may be withdrawn from further study injections for safety-related reasons, at the discretion of the site PIs, Emmes medical monitor, PATH medical officer, SRC independent members, or the participant/participant's parent.

Participants/parents may withdraw from study product administration for any other reason. If withdrawal is requested, the participants/parents should be asked to continue (at least limited) scheduled evaluations, complete a study termination form, and be given appropriate care under medical supervision until symptoms of any AE resolve or any condition becomes stable.

9.4. Study Pause

Study pause is defined as a decision to cease, temporarily or permanently, enrollment and all study injections. Study pause will not eliminate any safety follow-up procedures specified by protocol. The Sponsor will pause the study if the SRC determines that study pause criteria have been met. The DSMB will be convened by teleconference to review study pauses and provide recommendations to the Sponsor regarding continuation or permanent discontinuation of study vaccinations, or study modification.

9.4.1. Study Pause Rules

The following study pause rules will automatically pause or halt further study injections. These stopping rules refer to suspected adverse reactions, as defined below:

Study pause rules: Meeting one or more of the following criteria will automatically pause or halt further study injections in the study. These pause rules refer to suspected adverse reactions across all cohorts, as defined below:

- One participant with a vaccine-related serious AE (SAE).
- Two participants with ≥grade 3 (severe) localized inflammatory reaction at the injection site.
- The same objective ≥grade 3 (severe) systemic reactogenicity signs or symptoms (fever, vomiting), within seven days following study injection in three participants in Groups A, B or C, and ≥5% of participants in Group D.

- Two participants with a systemic rash, including, but not limited to, generalized urticaria, generalized petechiae, or erythema multiforme, within seven days following study injection.
- Two participants with the same ≥grade 3 (severe) vaccine-related abnormal clinical laboratory evaluation, within seven days following study injection.

9.4.2. Study Pause Procedure

- If the site PI (or designee), the Emmes medical monitor or any member of the SRC identifies that a pause criterion may have been met or proposes that the study be paused on a discretionary basis, all study injections and enrollment will be suspended. The DSMB will be notified and will convene expeditiously by conference call to review all available and relevant information, confirm whether pause criteria have been met, and provide recommendations to the Sponsor to permanently halt, re-start or otherwise modify the study. In the event that the study is permanently stopped, the HREC and WIRB will be notified within 48 hours.
- At scheduled study review meetings, the SRC may determine that pause criteria have been met, and the DSMB will be notified, as above.
- If the Sponsor re-starts the study after DSMB review and recommendation to restart, study injections may resume. Study injections that are delayed beyond the protocol-specified window may be administered. The appropriate follow-up and subsequent injection schedule for participants with delayed study injections will be determined by the Sponsor on a case-by-case basis.

9.5. Dose Escalation

The SRC will review available safety data through post-study injection Day 7 for all Group A participants in the 30 µg dose group (including safety monitoring laboratory tests) and determine whether to proceed with enrollment of the 90 µg dose group.

The SRC's evaluation of the safety data through Day 7 of all Group A participants in the 30 µg dose group, and any other safety data from Cohort A available at that time, will also be the basis for determining whether it is safe to proceed with enrollment of the 30 µg dose group in Group B. As above, the SRC will review safety data through post-study injection Day 7 in both the group A 90 µg dose and the group B 30 µg dose in deciding whether to progress from the 30 µg dose group to the 90 µg dose in Group B.

Similarly, the SRC's evaluation of the safety data through Day 7 of all Group B participants in the 30 µg dose group, and any other safety data from Groups A and B available at that time, will also be the basis for determining whether it is safe to proceed with enrollment of the 15 µg dose group in Group C. As above, the SRC will review safety data through post-study injection Day 7 in deciding whether to progress from the 15 µg dose group to the 30 µg dose in Group C, and then from the 30 µg dose group to the 90 µg dose.

The SRC will review the safety data through Day 7 for all participants in Groups A, B and C, and any additional data available at the time of deliberation, and determine whether to proceed with enrollment of Group D.

10. DATA MANAGEMENT

The site PIs are responsible for ensuring the accuracy, completeness, and timeliness of the data reported. Data collection is the responsibility of the clinical trial staff at the study site under the supervision of the site PIs. The Emmes Corporation is responsible for data management activities, including quality review, analysis, and reporting of the study data according to SOPs.

10.1. Case Report Form Development and Completion

Electronic Data Capture (EDC) will be the method of data collection in this study. The eCRFs will be developed by The Emmes Corporation and approved by PVS. Clinical data (including AEs, concomitant medications, and reactogenicity data) and clinical laboratory data will be entered into the 21 CFR Part 11-compliant Internet Data Entry System (IDES) provided by The Emmes Corporation. The data system includes password protection and internal quality checks, such as automatic range checks, to identify data that appear inconsistent, incomplete, or inaccurate. Clinical data will be entered directly from the source documents.

Data for each participant will be recorded in the eCRF. It is the site PIs' responsibility to ensure the accuracy, completeness, and timeliness of the data reported in the participant's eCRF and any supporting documentation. All source documents should be completed in a neat, legible manner to ensure accurate interpretation of data. Source documentation supporting the eCRF data should document the dates and details of study procedures, AEs and participant status. The site PIs/institutions will maintain all information in the eCRFs and all source documents that support the data collected from each participant.

The site PIs or designated representatives should complete the eCRFs as soon as possible after information is collected. Completed eCRFs must be submitted for each screened participant who signs (or whose parent signs) the study-specific informed consent form (ICF). The site PIs will retain all essential documents and a CD-ROM copy of the eCRF data (after the study is closed and unblinded and the Clinical Study Report is completed or at such time that the site no longer has access to the electronic data system).

10.2. Details of Data Management

A Data Management Handbook (DMH) will be written by The Emmes Corporation and contain all study-specific requirements.

10.2.1. Data Validation

The Emmes Corporation will inspect the data entered into the database for completeness and consistency.

10.2.2. Source Data Verification

For source data verification (SDV), the monitor (on behalf of the study Sponsor) must have direct access to source documents that support the data recorded, e.g., medical records, original laboratory records and ICFs. If source data are electronic, these data must be printed, signed and dated by the site PI and stored in the participant's study file. Clinical laboratory data will remain in study participant records. Essential documents, including ICFs, must be filed and kept in the study files on an ongoing basis.

10.2.3. Definition of Source Data

Source data are all information in original records or certified copies of original records of clinical findings, observations, or other activities in a clinical study. Source data are contained in source documents.

10.2.4. Definition of Source Document

Original source documents include data and records, e.g., hospital records, medical notes, laboratory notes, evaluation checklists, pharmacy dispensing records, records kept at the pharmacy and at the laboratory, documentation of shipments. Note that, for this protocol, the participant data collection tool (i.e., the Memory Aid) is not considered a source document (Refer to Appendix III) Data reported in the eCRF derived from source documents should be consistent with the source documents or the discrepancies should be explained.

10.3. Database Locking Procedures

A final database lock for the primary analysis will occur after all participants have completed all follow-up visits, including the 6 month safety follow-up call, a case-by-case review of the severity of any AEs has been performed and finalized, all data anomalies have been resolved and monitoring is complete.

Remaining immunology data will be maintained in a separate immunology database.

10.4. Record Archival

The site PIs are responsible for retaining study records for a period of 2 years following the date that a marketing application is approved for the product or, if no application is to be filed or, if a file application is not approved, until 2 years after the investigation is discontinued and the FDA is notified. The Sponsor will be responsible for providing the site with date of vaccine approval or IND withdrawal.

These records are also to be maintained in compliance with local EC and local authority medical records retention requirements, whichever is longest. Storage of all trial-related documents will be such that confidentiality will be strictly maintained to the extent provided by US and local law.

10.5. Screen Failures

If a participant or participant's parent signs the ICF but the participant is not randomized because of ineligibility (a screen failure), the reason for his/her ineligibility should be entered in the medical records/notes/charts. Also, a screening log must be kept. Data from participants who fail screening will not be recorded in the eCRF, with the exception of demographic data and the reason for screen fail.

10.6. Protocol Deviations

A protocol deviation is any noncompliance with the clinical trial protocol, GCP, or site SOP requirements. The noncompliance may be either on the part of the participant, the site PI, or the study site staff. As a result of deviations, corrective actions are to be developed by the site and implemented promptly.

These practices are consistent with ICH E6:

- Compliance with Protocol, Sections 4.5.1, 4.5.2, and 4.5.3
- Quality Assurance and Quality Control, Section 5.1.1
- Noncompliance, Sections 5.20.1 and 5.20.2.

It is the responsibility of the site to exercise continuous vigilance to identify and report deviations within 5 working days after identification of the protocol deviation, or within 5 working days of the scheduled protocol-required activity. All deviations must be promptly reported via the appropriate eCRF within the electronic data system.

All deviations from the protocol must be addressed in study participant source documents. A completed copy of WIRB reportable Protocol Deviation forms must be maintained in the regulatory file, as well as in the participant's source document. Protocol deviations must be sent to the local EC per its guidelines. The site PI/study staff is responsible for knowing and adhering to the respective EC's requirements.

11. STUDY MONITORING

Sponsor monitoring responsibilities will be provided through a CRO, SCT Consulting, experienced in monitoring clinical site activity. A site initiation visit will be conducted prior to beginning the study, and monitoring will be conducted at initiation, during, and at closeout of the study by the study monitor or designee.

During the course of the study, the monitor will visit the clinical site at intervals to verify compliance to the protocol; completeness, accuracy, and consistency of the data and study product accountability; adherence to CFR and MCC regulations, and any additional regulations and requirements, including GCP, of the conduct of clinical research. The monitor should have access to participant medical records, study product accountability and other study-related records needed to verify the entries on the eCRFs.

The site PIs and the monitor must agree to cooperate to ensure that any problems detected in the course of these monitoring visits, including eCRF completion and query resolution, are resolved in a predefined timeframe.

To ensure the quality of clinical data across all participants at the site, a clinical data management review will be performed on participant data received at The Emmes Corporation. During this review, participant data will be checked for consistency, omissions, and any apparent discrepancies. In addition, the data will be reviewed for adherence to the protocol and GCP. To resolve any questions arising from the clinical data management review process, data queries and/or site notifications will be sent to the site for resolution as soon as possible; all queries must be resolved prior to database lock.

Essential documents must be filed in the site study file on an ongoing basis and available for review by the Sponsor's contracted site monitor. Monitoring visits will be performed according to the Clinical Monitoring Plan.

11.1. Independent Auditing

PVS representatives may audit the study to ensure that study procedures and data collected comply with the protocol and applicable SOPs at the clinical site and The Emmes Corporation, and that data are correct and complete. The site PIs will permit auditors (employees of the Sponsor or an external company designated by the Sponsor) to verify source data validation of the regularly monitored clinical study. The auditors will compare the entries in the eCRFs with

the source data and evaluate the study site for its adherence to the clinical study protocol and GCP guidelines and applicable regulatory requirements.

11.2. Regulatory Agency Auditing

The site PIs must be aware that representatives from regulatory authorities or WIRB and their local ECs may wish to inspect the eCRFs and associated study records. The site PIs will notify the Sponsor within 24 hours following contact by a regulatory agency. The site PIs and study coordinators must make the relevant records available for inspection and will be available to respond to reasonable requests and audit queries made by authorized representatives of regulatory agencies. The site PIs will provide the Sponsor with copies of all correspondence that may affect the review of the current study or his qualification as an investigator in clinical studies conducted by the Sponsor. The Sponsor will provide any needed assistance in responding to regulatory audits or correspondence.

12. OBLIGATIONS AND ROLES OF THE SPONSOR, SITE PIS AND STUDY PERSONNEL

This study will be conducted according to GCP and in accordance with all US federal regulations regarding the protection of human subjects in research including US 21 CFR Part 50 and US 21 CFR Part 312, as well in accordance with South African MCC regulations.

The Sponsor will assure the trial is conducted in compliance with the protocol, GCP, and regulatory authority requirements. The Sponsor will provide the investigators with the funding and information needed to conduct the trial properly, ensuring proper monitoring of trial activities and that the trial is conducted in accordance with the general investigational plan and protocols contained in the submissions to the regulatory authorities. The Sponsor will ensure that regulatory authorities and the investigators are promptly informed of significant new adverse effects or risks with respect to the study vaccine.

The site PIs agree to perform the research in strict accordance with this protocol, the International Conference on Harmonisation (ICH) Guideline for Good Clinical Practice (E6), as well as in conformity with any US or local regulations regarding the conduct of clinical studies.

In addition, the site PIs must follow local and institutional requirements including, but not limited to, investigational product, clinical research, informed consent and local EC requirements. The Sponsor will provide notification to the site PIs of protocol and amendment approvals by regulatory authorities when applicable. Any modifications to the research protocol, the ICF, and/or the questionnaires or change in site PIs must be submitted to WIRB and respective local EC for review and approval prior to implementation. The site PIs may deviate from the protocol without prior approval only when the deviation is necessary to eliminate an apparent immediate hazard to the participant.

The site PIs will notify their respective ECs of SAEs and protocol deviations according to their EC requirements. Any deviation to the protocol that may have an effect on the safety or rights of the participant, or the integrity of the study, must be reported to the Sponsor by the site PIs as soon as the deviation is identified. In that event, the site PIs will notify the Sponsor immediately by phone, notify their respective ECs, enter the deviation into the appropriate eCRF, and confirm notification to the Sponsor in writing within 10 working days after the change is implemented. All deviations will be noted in the continuing review report to the respective ECs, the annual report to the Sponsor, and in the final study report for the ECs.

Except where the site PIs’ signatures are specifically required, it is understood that the term "investigator" as used in this protocol and on the electronic Case Report Forms (eCRFs) refers to the site PIs or appropriate study personnel that the site PIs designate to perform a certain duty. The site PIs are ultimately responsible for the conduct of all aspects of the study. Sub-

investigators or other appropriate study personnel are eligible to sign for the site PIs on designated eCRFs.

The Emmes medical monitor will be responsible for reviewing all serious and unexpected AEs and providing an unbiased written report of the event.

13. ETHICAL CONSIDERATIONS AND INFORMED CONSENT

13.1. Informed Consent Process

Before any study-related activities and in agreement with applicable regulatory requirements, the site PI must ensure that the participant's parent is fully informed about the aims, procedures, potential risks, and potential benefits of the study. The participant/participant's parent will be given the written, local EC/WIRB approved ICF, allowed ample time to read the consent form, encouraged to ask questions about the study, have the questions answered and then be given time to decide if s/he would like to participate or have her/his child participate in the study. It will be emphasized that participation is voluntary, and that the participant/parent has the right to withdraw from the study at any time without prejudice.

The site PIs or designees must obtain the participant's/parent's voluntary, signed and dated ICF (or, if the participant/parent is unable to sign, independently witnessed and documented consent) before any study-related procedures are performed. Study staff must document the informed consent process. The original, signed ICF must be kept in the site study file.

13.2. Risk/Benefit

No benefits can be guaranteed to participants for their participation in this research study.

Previous experience with the study product is limited to the first in human trial of the monovalent (P[8]) P2-VP8 subunit vaccine conducted in the United States and the ongoing study in toddlers and infants in South Africa. In those trials, the vaccine has been well-tolerated, with reactogenicity, when it occurred, predominantly mild in nature and no vaccine related SAEs were observed. See Section 1.6 for details.

As with any vaccine, severe allergic reaction is a potential rare event. None were observed in two clinical trials with the monovalent vaccine.

In this trial, participants in the infant groups are to receive Rotarix following completion of the study product series, to provide the protection that licensed vaccine provides. As it cannot yet be demonstrated that the P2-VP8 subunit vaccine does not interfere with response to Rotarix, participants will be provided a third dose of Rotarix. Additional risk mitigation will be provided by clinical monitoring and access to clinical evaluation and management.

13.3. Protocol Review Process

Scientific review of this protocol will be conducted by PATH. The protocol will be submitted the existing IND at the US FDA and to the MCC. The IND Sponsor will be PVS. Protocol ethical review and oversight will be performed by each site's local EC and WIRB. Continuing review will be undertaken in accordance with existing regulations. The Sponsor will be responsible for trial registration at ClinicalTrials.gov.

Copies of the approved continuing review and final study reports, along with the respective local IRB approval notifications, will be submitted to the Sponsor as soon as these documents become available.

13.4. Participant Confidentiality

The site PIs must ensure that participant confidentiality is maintained. Personal identifiers will not be included in any study reports. All study records will be kept confidential to the extent provided by national and local laws.

All study procedures will be conducted per GCP guidelines, and every effort will be made to protect participant privacy and confidentiality to the extent possible.

All study-related information will be stored securely at the study site or at a designated, secure off-site location. When not in use and under immediate control of study staff, all participant information will be stored in locked areas with access limited to study staff. Data collection, process, and administrative forms, laboratory specimens, and other reports will be identified exclusively by a coded number to maintain participant confidentiality. All local databases will be secured with password-protected access systems. Participants' study information will not be released without written parental permission, except as necessary for monitoring or compliance with legal or regulatory requirements.

Medical records containing identifying information may be made available for review when the study is monitored by the Sponsor or an authorized regulatory agency. Direct access may include examining, analyzing, verifying, and reproducing any records and reports that are important to the evaluation of the study.

13.5. Reimbursement

Pending local EC approval, participants/parents of participants will be compensated for their time and effort in this study, and be reimbursed for travel to study visits. The study ICF will state the plan for reimbursement. Participants/parents of study participants will not be charged for study injections, research clinic visits, research-related examinations, or research-related laboratory tests.

13.6. Storage of Specimens

Stored study research samples (including samples retained for elective analysis) will be labeled by a code that only the study site can link to the participant. All stored research samples will be logged into a secure database and any use documented. Samples may be stored at several different repositories and laboratories to complete the analyses required to meet study primary, secondary and exploratory analyses. As a part of the informed consent process, participants will be informed of and asked to agree to long-term storage of specimens for use in future, related research.

14. APPENDICES

- 14.1. APPENDIX I: Schedule of Events
  - 14.1.1. Schedule of Events for Group A (Adults)
  - 14.1.2. Schedule of Events for Group B (Toddlers)
  - 14.1.3. Schedule of Events for Groups C & D (Infants)
- 14.2. APPENDIX II: Toxicity Grading Scales
- 14.3. APPENDIX III: Memory Aid
- 14.4. APPENDIX IV: Literature Cited

14.1. APPENDIX I: Schedule of Events

14.1.1. Schedule of Events for Group A (Adults)

	Screen	Day 0			Day 7 (±1)	Day 28 (-2 to +7)			Day 35 (±1)	Day 56 (-2 to +7)			Day 63 (±1)	Day 84 (±4)	Day 224 (±14)
**Visit Number**	**1**	**2**			**3**	**4**			**5**	**6**			**7**	**8**	**9**
**Assessment**	**Day -28 to -1**	*****	**0**	**>30 min**		*****	**0**	**>30 min**		*****	**0**	**>30 min**			
Informed Consent	X														
Demographics	X														
Medical History	X														
Medical History Update		X			X	X			X	X			X	X	X
Prior and Concurrent Medications	X	X			X	X			X	X			X	X	
Inclusion & Exclusion Criteria	X	X				X				X					
Physical Examination	X	X			X[C]	X[C]			X[C]	X[C]			X[C]	X[C]	X[C]
Study injections			X				X				X				
Injection Site Assessment		X		X	X	X		X	X	X		X	X	X	
AE Assessment		X		X	X	X		X	X	X		X	X	X	X
Memory Aid / reminders				X	X			X	X			X	X		
Vital Signs	X	X		X	X	X		X	X	X		X	X	X	
Hematology[A]	X				X										
Clinical Chemistry[B]	X				X										
Pregnancy test[D]	X	X				X				X					
HIV 1/2 Ab	X														
Serum for IgG&A/Neutralizing Ab	X					X				X				X	
Safety Blood Volume†	20				10										
Immunogenicity Blood Volume†	10						10				10			10	
Cumulative Blood Volume†	30				40		50				60			70	

* Prior to study injection
† All blood volume units mL
A. Complete blood count (CBC), including WBC, hemoglobin, and platelet count
B. Chemistry Panel: ALT, bilirubin, albumin, and creatinine (albumin at screening only)
C. Targeted physical exam, as indicated
D. Serum pregnancy test at screening and urine pregnancy test on the day of each vaccination for female participants (prior to vaccination)

14.1.2. Schedule of Events for Group B (Toddlers)

	Screen	Day 0			Day 3	Day 7 (±1)	Day 28 (±4)	Day 168 (±14)
**Visit Number**	**1**	**2**			**3**	**4**	**5**	**6**
**Assessment**	**Day -28 to -1**	*****	**0**	**>30 min**				
Informed Consent	X							
Demographics	X							
Medical History	X							
Medical History Update		X			X	X	X	X
Prior and Concurrent Medications	X	X			X	X	X	
Inclusion & Exclusion Criteria	X	X						
Physical Examination	X	X				X[C]	X[C]	X[C]
Study injections			X					
Injection Site Assessment		X		X	X	X	X	
AE Assessment		X		X	X	X	X	X
Memory Aid / reminders				X	X	X		
Vital Signs	X	X		X	X	X	X	
Hematology[A]	X					X		
Clinical Chemistry[B]	X					X		
HIV 1/2 Ab	X							
Safety Blood Volume†	5					5		
Immunogenicity Blood Volume†	5						5	
Cumulative Blood Volume†	10					15	20	

* Prior to study injection
† All blood volume units mL
A. Complete blood count (CBC), including WBC, hemoglobin, and platelet count
B. Chemistry Panel: ALT, bilirubin, albumin, and creatinine (albumin at screening only)
C. Targeted physical exam, as indicated

14.1.3. Schedule of Events for Groups C & D (Infants)

	Screen	Day 0			Day 3	Day 7 (±1)	Day 28 (-2 to +7)			Day 31	Day 35 (±1)	Day 56 (-2 to +7)			Day 59	Day 63 (±1)	Day 84 (±4)	Days 89, 91, 93	Day 112 (±14)	Day 140 (±14)	Day 224 (±14)
**Visit Number**	**1**	**2**			**3**	**4**	**5**			**6**	**7**	**8**			**9**	**10**	**11**		**12**	**13**	**14**
**Assessment**	**Day -7 to -1**	*****	**0**	**>30 min**			*****	**0**	**>30 min**			*****	**0**	**>30 min**							
Informed Consent	X																				
Demographics	X																				
Medical History	X																				
Medical History Update		X			X	X	X			X	X	X			X	X	X		X	X	X
Prior and Concurrent Medications	X	X			X	X	X			X	X	X			X	X	X				
Inclusion & Exclusion Criteria	X	X					X					X									
Physical Examination	X	X				X^C	X				X^C	X				X^C	X^C		X^C	X^C	X^C
Study injections			X					X					X								
EPI vaccinations (except rotavirus)			X					X					X								
Rotarix administration																	X		X	X	
Injection Site Assessment		X		X	X	X	X		X	X	X	X		X	X	X	X				
AE Assessment		X		X	X	X	X		X	X	X	X		X	X	X	X		X	X	X
Memory Aid / reminders				X	X	X			X	X	X			X	X	X					
Vital Signs	X	X		X	X	X	X		X	X	X	X		X	X	X	X		X	X	
Hematology[A]	X					X															
Clinical Chemistry[B]	X					X															
HIV PCR[D]	X																				
Serum for IgG&A/Neutralizing Ab	X											X					X				
Stool for Rotarix shedding (RMPRU)																		X			
Safety Blood Volume, Group C†	5					5															
Safety Blood Volume, Group D†	5																				
Immunogenicity Blood Volume†	5											5					5				
Cumulative Blood Volume, Group C†	10					15						20					25				
Cumulative Blood Volume, Group D†	10											15					20				

* Prior to study injection
† All blood volume units mL
A. Complete blood count (CBC), including WBC, hemoglobin, and platelet count
B. Chemistry Panel: ALT, bilirubin, albumin, and creatinine (albumin at screening only)
C. Targeted physical exam, as indicated
D. If mother not known to be negative (negative test result between 24 weeks gestation and screening)
E. Stool samples to be obtained from infants with diarrhea to test for rotavirus

14.2. APPENDIX II: Toxicity Grading Scales

Systemic Illness	Mild (Grade 1)	Moderate (Grade 2)	Severe (Grade 3)	Potentially Life Threatening (Grade 4)
Illness or clinical AE (as defined according to applicable regulations)	No or minimal interference with usual activities; no medical intervention/ therapy required	Greater than minimal interference with usual activities; no or minimal medical intervention/ therapy required	Marked limitation in ability to perform usual activities; medical intervention/ therapy required	Inability to perform basic functions OR Medical or operative intervention indicated to prevent permanent impairment, persistent disability, or death

Local Reaction to Injectable Product	Mild (Grade 1)	Moderate (Grade 2)	Severe (Grade 3)	Potentially Life Threatening (Grade 4)
Injection site pain (pain without touching) OR Tenderness (pain when area touched)	Pain/tenderness causing no or minimal limitation of use of limb	Pain or tenderness causing greater than minimal limitation of use of limb	Pain/tenderness causing inability to perform usual activities	Pain/tenderness causing inability to perform basic functions OR Hospitalization indicated
Injection site erythema or induration<br><br>Infants and toddlers	≤ 2.5 cm in diameter	> 2.5 cm in diameter with < 50% surface area of the extremity segment involved (thigh)	≥ 50% surface area of the extremity segment involved (thigh) OR Ulceration OR Secondary infection OR Phlebitis OR Sterile abscess OR Drainage	Potentially life-threatening consequences (e.g., abscess, exfoliative dermatitis, necrosis involving dermis or deeper tissue)
Injection site erythema or induration<br><br>Adults	2.5 to < 5 cm in diameter OR 6.25 to < 25 cm^2 surface area AND Symptoms causing no or minimal interference with usual social & functional activities	≥ 5 to < 10 cm in diameter OR ≥ 25 to < 100 cm^2 surface area OR Symptoms causing greater than minimal interference with usual social & functional activities	≥ 10 cm in diameter OR ≥ 100 cm^2 surface area OR Ulceration OR Secondary infection OR Phlebitis OR Sterile abscess OR Drainage OR Symptoms causing inability to perform usual social & functional activities	Potentially life-threatening consequences (e.g., abscess, exfoliative dermatitis, necrosis involving dermis or deeper tissue)
Injection site pruritus	Itching localized to the injection site that is relieved spontaneously or in < 48 hours of treatment	Itching beyond the injection site that is not generalized OR Itching localized to the injection site requiring ≥ 48 hours treatment	Generalized itching causing inability to perform usual social & functional activities	NA

Systemic (General)	Mild (Grade 1)	Moderate (Grade 2)	Severe (Grade 3)	Potentially Life Threatening (Grade 4)
Acute systemic allergic reaction	Localized urticaria (wheals) with no medical intervention indicated	Localized urticaria with medical intervention indicated OR Mild angioedema with no medical intervention indicated	Generalized urticaria OR Angioedema with medical intervention indicated OR Symptomatic mild bronchospasm	Acute anaphylaxis OR Life-threatening bronchospasm OR laryngeal edema
Fever	37.7 – 38.6°C	38.7 – 39.3°C	39.4 – 40.5°C	> 40.5°C
Vomiting	Transient or intermittent vomiting with no or minimal interference with oral intake	Frequent episodes of vomiting with no or mild dehydration	Persistent vomiting resulting in greater than milde dehydration OR Aggressive rehydration indicated (e.g., IV fluids)	Life-threatening consequences (e.g., hypotensive shock)
Irritability	Crying more than usual but easily consoled	Crying more than usual and somewhat difficult to console	Continuous crying that is inconsolable for 4 hours or more	
Decreased activity	Slightly subdued, but responds normally to stimuli	Subdued and does not respond as readily as normal to stimuli	Lethargic	Obtunded
Decreased appetite	Loss of appetite without decreased oral intake	Loss of appetite associated with decreased oral intake without significant weight loss	Loss of appetite associated with significant weight loss	Life-threatening consequences OR Aggressive intervention indicated [e.g., tube feeding or total parenteral nutrition (TPN)]
Nausea	Transient (< 24 hours) or intermittent AND No or minimal interference with oral intake	Persistent nausea resulting in decreased oral intake for 24 to 48 hours	Persistent nausea resulting in minimal oral intake for > 48 hours OR Rehydration indicated (e.g., IV fluids)	Life-threatening consequences (e.g., hypotensive shock)
Myalgia (generalized)	Muscle pain causing no or minimal interference with usual social & functional activities	Muscle pain causing greater than minimal interference with usual social & functional activities	Muscle pain causing inability to perform usual social & functional activities	Disabling muscle pain causing inability to perform basic self-care functions
Headache	Symptoms causing no or minimal interference with usual social & functional activities	Symptoms causing greater than minimal interference with usual social & functional activities	Symptoms causing inability to perform usual social & functional activities	Symptoms causing inability to perform basic self-care functions OR Hospitalization indicated OR Headache with significant impairment of alertness or other neurologic function

Systemic (General)	Mild (Grade 1)	Moderate (Grade 2)	Severe (Grade 3)	Potentially Life Threatening (Grade 4)
Chills	Symptoms causing no or minimal interference with usual social & functional activities	Symptoms causing greater than minimal interference with usual social & functional activities	Symptoms causing inability to perform usual social & functional activities	NA
Fatigue	Symptoms causing no or minimal interference with usual social & functional activities	Symptoms causing greater than minimal interference with usual social & functional activities	Symptoms causing inability to perform usual social & functional activities	Incapacitating symptoms of fatigue or malaise causing inability to perform basic self-care functions

**Chemistry**	**Mild (Grade 1)**	**Moderate (Grade 2)**	**Severe (Grade 3)**	**Potentially Life Threatening (Grade 4)****
**Creatinine**	1.1-1.3 ULN	>1.3-1.8 ULN	>1.8 to <3.5 ULN	≥3.5 ULN
**Albumin – Hypoalbuminemia G/L**	NA	20 to <30	<20	--
**ALT**	1.25 to <2.5 ULN	2.5 to <5.0 ULN	5.0 to <10.0 ULN	≥10 ULN
**Bilirubin**	1.1 to <1.6 ULN	1.6 to <2.6 ULN	2.6 to <5.0 ULN	≥5.0 ULN
**Hematology**	**Mild (Grade 1)**	**Moderate (Grade 2)**	**Severe (Grade 3)**	**Potentially Life Threatening (Grade 4)**
**Hemoglobin - g/dL**				
36 to 56 days of age (male and female)	8.5 to 9.6	7.0 to <8.5	6.0 to <7.0	<6.0
57 days of age to < 13 years of age (male and female)	9.5 to 10.4	8.5 to <9.5	6.5 to <8.5	<6.5
≥ 13 years of age (male only)	10.0 to 10.9	9.0 to <10.0	7.0 to <9.0	<7.0
≥ 13 years of age (female only)	9.5 to 10.4	8.5 to <9.5	6.5 to <8.5	<6.5
**WBC Decrease** - cell/mm^3	2,000 to 2,499	1,500 to 1,999	1,000 to 1,499	< 1,000
**Platelets** - cell/mm^3	100 to <124 X10^3	50 to <100 X10^3	25 to <50 X10^3	<25 X10^3

14.3. APPENDIX III: Memory Aid

Participant and Parental Assessments and Use of Memory Aid

The Memory Aid prompts daily participant and parental assessment and recording in the Memory Aid until 7 days following dosing, beginning about 8 hours post-dose on the dosing day and thereafter each night. The participant/parent assesses and records observations of all signs and symptoms at the injection site, systemic signs and symptoms, and other AEs.

The Memory Aid specifically prompts assessment of the solicited injection site reactions considered to be study endpoints (Section 5.5.6). The participant's/parent's assessment of reactogenicity will follow the Toxicity Grading Scale provided in Appendix II. The participant/parent takes and records oral (adults) or axillary (toddlers and infants) temperature (with thermometer provided by study staff) at each assessment time. The Memory Aid prompts that, if temperature exceeds 38 degrees Celsius, the participant/parent then repeats and records temperature measurements every 4 hours until temperature is lower than 38 degrees Celsius. Participants/parents will be provided with a standardized tool to measure diameter of palpable swelling and erythema (redness). The Memory Aid prompts the participant/parent to call site staff if redness or swelling widest diameter exceeds grade 1.

The Memory Aid is not used as the source document for data entry into the electronic data capture system. Study staff will review the Memory Aid with the participant/parent to elicit as much information as possible about any reported symptoms. Based on this information, study staff will use their clinical judgment to assess the event, and its severity, and record the data on the source document that captures reactogenicity symptoms, which will be used for the purposes of data entry. The existence of any discrepancies between the participant/parent and staff assessment of event severity will be noted on the case report form. The Memory Aid will be retained with participant study records.

On Days 3 and 7 after each study injection, study staff will review the Memory Aid information during a scheduled clinic visit (for adults, Day 7 only). The participant/parent will be instructed to bring the Memory Aid to any in-person clinic visits within 7 days after a vaccine administration.

Any additional, unsolicited symptoms will be similarly discussed with the participant/parent, and clinic staff should evaluate the event(s) according to their clinical judgment and guidance provided in the protocol. This assessment should be described carefully in source documents and entered on the AE form. Any reported Concomitant Medications are recorded on the Concomitant Medications form.

14.4. APPENDIX IV: Literature Cited

1. Tate, J.E., et al., 2008 estimate of worldwide rotavirus-associated mortality in children younger than 5 years before the introduction of universal rotavirus vaccination programmes: a systematic review and meta-analysis. The Lancet infectious diseases, 2012. **12**(2): p. 136-41.
2. WHO, Rotavirus vaccines. Weekly Epidemiological Record, 2007. **82**(32): p. 285-95.
3. Jiang, B., J.R. Gentsch, and R.I. Glass, Inactivated rotavirus vaccines: a priority for accelerated vaccine development. Vaccine, 2008. **26**(52): p. 6754-8.
4. Diggle, L., Rotavirus diarrhoea and future prospects for prevention. Br J Nurs, 2007. **16**(16): p. 970-4.
5. Santos, N. and Y. Hoshino, Global distribution of rotavirus serotypes/genotypes and its implication for the development and implementation of an effective rotavirus vaccine. Reviews in medical virology, 2005. **15**(1): p. 29-56.
6. Tshangela, A., et al., Rotavirus Surveillance in South Africa, 2011. Communicable Diseases Surveillance Bulletin, 2012. **10**(3): p. 42-46.
7. Mwenda, J.M., et al., Burden and epidemiology of rotavirus diarrhea in selected African countries: preliminary results from the African Rotavirus Surveillance Network. The Journal of infectious diseases, 2010. **202 Suppl**: p. S5-S11.
8. Estes, M.K., D.Y. Graham, and B.B. Mason, Proteolytic enhancement of rotavirus infectivity: molecular mechanisms. Journal of virology, 1981. **39**(3): p. 879-88.
9. Arias, C.F., M. Lizano, and S. Lopez, Synthesis in Escherichia coli and immunological characterization of a polypeptide containing the cleavage sites associated with trypsin enhancement of rotavirus SA11 infectivity. The Journal of general virology, 1987. **68 (Pt 3)**: p. 633-42.
10. Bellido, D., et al., Brucella spp. lumazine synthase as a bovine rotavirus antigen delivery system. Vaccine, 2009. **27**(1): p. 136-45.
11. Dunn, S.J., et al., Immunogenicity, antigenicity, and protection efficacy of baculovirus expressed VP4 trypsin cleavage products, VP5(1)* and VP8* from rhesus rotavirus. Arch Virol, 1995. **140**(11): p. 1969-78.
12. Gil, M.T., et al., Homotypic protection against rotavirus-induced diarrhea in infant mice breast-fed by dams immunized with the recombinant VP8* subunit of the VP4 capsid protein. Viral Immunol, 2000. **13**(2): p. 187-200.
13. Kovacs-Nolan, J., et al., Cloning and expression of human rotavirus spike protein, VP8*, in Escherichia coli. Biochem Biophys Res Commun, 2001. **282**(5): p. 1183-8.
14. Larralde, G., et al., Serotype-specific epitope(s) present on the VP8 subunit of rotavirus VP4 protein. Journal of virology, 1991. **65**(6): p. 3213-8.
15. Marelli, B., et al., Oral immunization with live Lactococcus lactis expressing rotavirus VP8 subunit induces specific immune response in mice. J Virol Methods, 2011. **175**(1): p. 28-37.
16. Padilla-Noriega, L., et al., Humoral immune responses to VP4 and its cleavage products VP5* and VP8* in infants vaccinated with rhesus rotavirus. Journal of clinical microbiology, 1992. **30**(6): p. 1392-7.
17. Perez Filgueira, D.M., et al., Passive protection to bovine rotavirus (BRV) infection induced by a BRV VP8* produced in plants using a TMV-based vector. Arch Virol, 2004. **149**(12): p. 2337-48.

18. Tan, M., et al., Norovirus P particle, a novel platform for vaccine development and antibody production. Journal of virology, 2011. **85**(2): p. 753-64.

19. Matsui, S.M., et al., Passive protection against rotavirus-induced diarrhea by monoclonal antibodies to the heterotypic neutralization domain of VP7 and the VP8 fragment of VP4. Journal of clinical microbiology, 1989. **27**(4): p. 780-2.

20. Kirkwood, C.D., R.F. Bishop, and B.S. Coulson, Human rotavirus VP4 contains strain-specific, serotype-specific and cross-reactive neutralization sites. Arch Virol, 1996. **141**(3-4): p. 587-600.

21. Kovacs-Nolan, J., D. Yoo, and Y. Mine, Fine mapping of sequential neutralization epitopes on the subunit protein VP8 of human rotavirus. Biochem J, 2003. **376**(Pt 1): p. 269-75.

22. Favacho, A.R., et al., Cloning, expression, and purification of recombinant bovine rotavirus hemagglutinin, VP8*, in Escherichia coli. Protein Expr Purif, 2006. **46**(2): p. 196-203.

23. Lee, J., et al., Immunological response to recombinant VP8* subunit protein of bovine roravirus in pregnant cattle. The Journal of general virology, 1995. **76 (Pt 10)**: p. 2477-83.

24. Andres, I., et al., Yeast expression of the VP8* fragment of the rotavirus spike protein and its use as immunogen in mice. Biotechnol Bioeng, 2006. **93**(1): p. 89-98.

25. Lentz, E.M., et al., VP8* antigen produced in tobacco transplastomic plants confers protection against bovine rotavirus infection in a suckling mouse model. J Biotechnol, 2011. **156**(2): p. 100-7.

26. Istrate, C., et al., Parenteral administration of RF 8-2/6/7 rotavirus-like particles in a one-dose regimen induce protective immunity in mice. Vaccine, 2008. **26**(35): p. 4594-601.

27. Ahlborg, N., et al., Linkage of exogenous T-cell epitopes to the 19-kilodalton region of Plasmodium yoelii merozoite surface protein 1 (MSP1(19)) can enhance protective immunity against malaria and modulate the immunoglobulin subclass response to MSP1(19). Infection and immunity, 2000. **68**(4): p. 2102-9.

28. Falugi, F., et al., Rationally designed strings of promiscuous CD4(+) T cell epitopes provide help to Haemophilus influenzae type b oligosaccharide: a model for new conjugate vaccines. Eur J Immunol, 2001. **31**(12): p. 3816-24.

29. Wen, X., et al., Construction and characterization of human rotavirus recombinant VP8* subunit parenteral vaccine candidates. Vaccine, 2012. **30**(43): p. 6121-6.

30. Wen, X., et al., Inclusion of a universal tetanus toxoid CD4(+) T cell epitope P2 significantly enhanced the immunogenicity of recombinant rotavirus DeltaVP8* subunit parenteral vaccines. Vaccine, 2014. **32**(35): p. 4420-7.

31. Baylor, N.W., W. Egan, and P. Richman, Aluminum salts in vaccines--US perspective. Vaccine, 2002. **20 Suppl 3**: p. S18-23.

32. Cadranel, S., et al., Factors affecting antibody response of newborns to repeated administrations of the rotavirus vaccine RIT 4237. Journal of pediatric gastroenterology and nutrition, 1987. **6**(4): p. 525-8.

33. Westerman, L.E., et al., Serum IgG mediates mucosal immunity against rotavirus infection. Proc Natl Acad Sci U S A, 2005. **102**(20): p. 7268-73.

List of VAC 046 Protocol Changes (version 3.0 to version 4.0)

The overarching reason for the protocol modifications is the addition of assessment of fecal shedding of Rotarix subsequently administered in healthy South African infants

Section in amended protocol	Old text	New/Revised text	Reason for change
All page headers	Version 3.0; 18 February 2016	Version 4.0; 09 June 2016	Updated protocol version number and date.
Cover page	Version 3.0; 18 February 2016	Version 4.0; 09 June 2016	Updated protocol version number and date.
**STUDY SYNOPSIS – STUDY OBJECTIVES**	No previous text	Exploratory Objective:<br>Efficacy<br>• To evaluate the impact of the trivalent P2-VP8 subunit rotavirus vaccination on shedding of Rotarix subsequently administered in healthy South African infants as a test of concept	Evaluation of the impact of the trivalent vaccine on shedding of Rotarix added as an Exploratory Objective.
**STUDY SYNOPSIS - STUDY ENDPOINTS**	No previous text	Exploratory Endpoint:<br>• Proportion of participants shedding Rotarix after administration of Rotarix "challenge"	Assessment of fecal shedding of Rotarix in infants added as an Exploratory Endpoint.
**STUDY SYNOPSIS - STUDY DESIGN**	All infants will receive 3 doses of Rotarix, at monthly intervals, starting a month after the final study injection (on study Day 84, at the time the post-vaccination blood sample is obtained)	All infants will receive 3 doses of Rotarix, at monthly intervals, starting a month after the final study injection (on study Day 84, at the time the post-vaccination blood sample is obtained), and fecal shedding of Rotarix will be assessed during the week after the first dose on a subset of infants.	Assessment of fecal shedding of Rotarix on a subset of infants added.
**STUDY SYNOPSIS - STUDY PROCEDURES & VISIT SCHEDULE**	Infants will receive Rotarix vaccination 28, 56 and 84 days following completion of the 3 study injections. (Study Days 84, 112 and 140).	Infants will receive Rotarix vaccination 28, 56 and 84 days following completion of the 3 study injections. (Study Days 84, 112 and 140). Stool samples will be obtained to assess Rotarix shedding 5, 7, and 9 days following the first dose of Rotarix (Study Days 89, 91, and 93) on a subset of infants.	Updated text to include collection of stool at 5, 7, and 9 days following the first dose of Rotarix in infants. (Study Days 89, 91, and 93) on a subset of infants.
**1.5.2 GLP Immunogenicity and Toxicology Study of the Trivalent P2-VP8 Subunit Vaccine in NZW Rabbits**	Microscopic changes observed two days following the final administration of trivalent P2-VP8 subunit vaccine were limited to the injection site and were resolved or resolving following a six-week recovery period.	Microscopic changes observed two days following the final administration of trivalent P2-VP8 subunit vaccine were limited to the injection site and were resolved or resolving following a four-week recovery period	Corrected the study recovery period from six weeks to four weeks.

Section in amended protocol	Old text	New/Revised text	Reason for change
**2.2 Scientific Rationale**	No previous text	Further, as in VAC 013, the impact of the trivalent vaccine on the replication of Rotarix, which will be administered to the infants after completion of the 3-dose regimen of P2-VP8, will be assessed. Impact on shedding of Rotarix will be interpreted as a surrogate of protection. This assessment will provide an indication of whether the addition of two more antigens alters the impact observed in VAC 013 with the monovalent vaccine.	Added assessment of the impact of the trivalent vaccine on the replication of Rotarix in infants.
**2.4 Study Design**	No previous text	All infants will receive Rotarix at 4, 8 and 12 weeks after the third study injection (study Days 84, 112 and 140), and fecal shedding of Rotarix will be assessed during the week after the first dose in a subset of infants.	Added in text to address the collection of additional stool samples after the Rotarix vaccinations.
New subsection inserted under Section **2.4 Study Design**	No previous text	2.4.3 Exploratory Objective:<br>Efficacy<br>• To evaluate the impact of the trivalent P2-VP8 subunit rotavirus vaccination on shedding of Rotarix subsequently administered in healthy South African infants as a test of concept	Evaluation of the impact of the trivalent vaccine on shedding of Rotarix added as an Exploratory Objective.
New subsection inserted under Section **2.4 Study Design**	No previous text	2.4.6 Exploratory Endpoint:<br>• Proportion of participants shedding Rotarix after administration of Rotarix "challenge	Assessment of fecal shedding of Rotarix in infants added as an Exploratory Endpoint.
New subsection inserted under Section **5.2.7 Groups C and D (infants)**	No previous text	5.2.7.7 Day 89 – Clinic visit for stool sampling (RMPRU only)<br>The parent will return to the clinic with a stool sample obtained that morning. If the parent is unable to bring the specimen to the clinic, a clinic staff member will visit the home to collect the sample. If the infant does not pass stool during the morning, a specimen will be obtained by rectal swab. Parents will be provided an empty container to obtain stool sample on the morning of Day 91.	Stool collection at Day 89 clinic visit added. Assessment of fecal shedding of Rotarix in infants to be conducted at the RMPRU site only.
New subsection inserted under **Section 5.2.7 Groups C and D (infants)**	No previous text	5.2.7.8 Day 91 – Clinic visit for stool sampling (RMPRU only)<br>The parent will return to the clinic with a stool sample obtained that morning. If the parent is unable to bring the specimen to the clinic, a clinic staff member will visit the home to collect the sample. If the infant does not pass stool during the morning, a specimen will be obtained by rectal swab. Parents will be provided an empty container to obtain stool sample on the morning of Day 93.	Stool collection at Day 91 clinic visit added. Assessment of fecal shedding of Rotarix in infants to be conducted at the RMPRU site only.

Section in amended protocol	Old text	New/Revised text	Reason for change
New subsection inserted under Section **5.2.7 Groups C and D (infants)**	No previous text	5.2.7.9 Day 93 – Clinic visit for stool sampling (RMPRU only)<br>The parent will return to the clinic with a stool sample obtained that morning. If the parent is unable to bring the specimen to the clinic, a clinic staff member will visit the home to collect the sample. If the infant does not pass stool during the morning, a specimen will be obtained by rectal swab. The next clinic visit is scheduled.	Stool collection at Day 93 clinic visit added. Assessment of fecal shedding of Rotarix in infants to be conducted at the RMPRU site only.
New subsection inserted under Section **6.0 Laboratory Evaluations**	No previous text	6.6 Rotarix shedding<br>At one study site, RMPRU, stool samples will be obtained 5, 7 and 9 days following the first dose of Rotarix for qualitative assessment of vaccine virus shedding at NICD.<br>All stool samples will be tested for the presence of rotavirus using the commercially available ProsPecT™ Rotavirus Microplate Assay (Oxoid Ltd, Ely, United Kingdom), according to the manufacturer's instructions.<br>ELISA positive specimens may be confirmed and genotyped by PCR amplification of the VP7 and VP4 genes with a panel of G- and P-specific primers, respectively (WHO Manual of rotavirus detection and characterization methods, Methods 14 and 15 with minor modifications). The primer sets include: G-specific primer set (Beg9, End9, aAT8v, aBT1, aCT2, aDT4, mG3, mG9, mG10, mG12b and EndA) for the detection of G1, G2, G3, G4, G8, G9, G10 and G12 rotavirus strains and P-specific primer set (con2, con3, 1T-1v, 2T-1, 3T-1, 4T-1, 5T-1, mP11, p4943 and VP4F) for the detection of P[4], P[6], P[8], P[9], P[10], P[11] and P[14] rotavirus strains. Genotype G1 and/or P[8] rotavirus strains will be sequenced to determine if the strain is vaccine derived or a wild-type case. The VP7 (1062bp) and VP4 (876 bp) genes amplified by PCR will be purified and sequenced using the BigDye® Terminator v 3.1 cycle sequencing kits (Applied Biosystems). Reactions will be run on a 3500 Genetic Analyzer (Applied Biosystems) and data analyzed with manufacturer supplied software. Sequences will be directly compared to the VP7 and VP4 genes of the Rotarix strains (sequences available on Genbank; JN849114.1 VP7 and JN849113.1 VP4) and submitted to GenBank for BLASTn analyses to establish whether the VP7 and VP4 genes are from the vaccine or from wild-type G1P[8] strains.	Assessment of shedding of Rotarix following the first dose of Rotarix as a measure of efficacy added. Assessment to be conducted at the RMPRU site only.

Section in amended protocol	Old text	New/Revised text	Reason for change
New subsection inserted under Section **7.3 Objectives and Endpoints**	No previous text	7.3.3 Exploratory Objective<br>Efficacy<br>To evaluate the impact of the trivalent P2-VP8 subunit rotavirus vaccine on shedding of Rotarix administered following P2-VP8 subunit rotavirus vaccination of healthy South African infants as a test of concept.<br>Endpoint:<br>• Proportion of participants with Rotarix vaccine virus detected in stools on Days 5-9 following the first dose of Rotarix, which is to be administered 28 days after the third study injection	Evaluation of the impact of the trivalent vaccine on shedding of Rotarix added as an Exploratory Objective. Detection of Rotarix vaccine virus in stools of infants on Days 5-9 added as an Endpoint.
New text inserted under Section **7.5 Analytical Methodology**	No previous text	Assessment of shedding of Rotarix virus will be performed for each of the three specified post-vaccination days and for shedding on any of the three days. Rates of shedding between the placebo and (1) each dose group and (2) the combined three dose groups will be compared using the Fisher's exact test.	Added specific timepoints (three specified post-vaccination days) for Rotarix shedding assessment.
**14.1 APPENDIX I: Schedule of Events**<br>**14.1.3. Schedule of Events for Groups C and D (Infants)**	No previous text	(New table column and row inserted) Clinic visit days 89, 91 and 93	Added clinic visit days 89, 91 and 93 for stool collections at (RMPRU site only.

List of VAC 041 Protocol Changes (version 3.0 to version 4.0)

The overarching reason for the protocol modifications is the addition of assessment of fecal shedding of Rotarix subsequently administered in healthy South African infants

Section in amended protocol	Old text	New/Revised text	Reason for change
All page headers	Version 3.0; 18 February 2016	Version 4.0; 21 June 2016	Updated protocol version number and date.
Cover page	Version 3.0; 18 February 2016	Version 4.0; 21 June 2016	Updated protocol version number and date.
**STUDY SYNOPSIS - STUDY OBJECTIVES**	No previous text	Exploratory Objective:<br>Efficacy<br>• To evaluate the impact of the trivalent P2-VP8 subunit rotavirus vaccination on shedding of Rotarix subsequently administered in healthy South African infants as a test of concept	Evaluation of the impact of the trivalent vaccine on shedding of Rotarix added as an Exploratory Objective.
**STUDY SYNOPSIS - STUDY ENDPOINTS**	No previous text	Exploratory Endpoint:<br>• Proportion of participants shedding Rotarix after administration of Rotarix "challenge"	Assessment of fecal shedding of Rotarix in infants added as an Exploratory Endpoint.
**STUDY SYNOPSIS - STUDY DESIGN**	All infants will receive 3 doses of Rotarix, at monthly intervals, starting a month after the final study injection (on study Day 84, at the time the post-vaccination blood sample is obtained)	All infants will receive 3 doses of Rotarix, at monthly intervals, starting a month after the final study injection (on study Day 84, at the time the post-vaccination blood sample is obtained), and fecal shedding of Rotarix will be assessed during the week after the first dose on a subset of infants.	Assessment of fecal shedding of Rotarix on a subset of infants added.
**STUDY SYNOPSIS - STUDY PROCEDURES & VISIT SCHEDULE**	Infants will receive Rotarix vaccination 28, 56 and 84 days following completion of the 3 study injections. (Study Days 84, 112 and 140).	Infants will receive Rotarix vaccination 28, 56 and 84 days following completion of the 3 study injections. (Study Days 84, 112 and 140). Stool samples will be obtained to assess Rotarix shedding 5, 7, and 9 days following the first dose of Rotarix (Study Days 89, 91, and 93) on a subset of infants.	Updated text to include collection of stool at 5, 7, and 9 days following the first dose of Rotarix in infants. (Study Days 89, 91, and 93) on a subset of infants.
**1.5.2 GLP Immunogenicity and Toxicology Study of the Trivalent P2-VP8 Subunit Vaccine in NZW Rabbits**	Microscopic changes observed two days following the final administration of trivalent P2-VP8 subunit vaccine were limited to the injection site and were resolved or resolving following a six-week recovery period.	Microscopic changes observed two days following the final administration of trivalent P2-VP8 subunit vaccine were limited to the injection site and were resolved or resolving following a four-week recovery period	Corrected the study recovery period from six weeks to four weeks.

List of VAC 041 Protocol Changes (version 3.0 to version 4.0)

Section in amended protocol	Old text	New/Revised text	Reason for change
**2.2 Scientific Rationale**	No previous text	Further, as in VAC 013, the impact of the trivalent vaccine on the replication of Rotarix, which will be administered to the infants after completion of the 3-dose regimen of P2-VP8, will be assessed. Impact on shedding of Rotarix will be interpreted as a surrogate of protection. This assessment will provide an indication of whether the addition of two more antigens alters the impact observed in VAC 013 with the monovalent vaccine.	Added assessment of the impact of the trivalent vaccine on the replication of Rotarix in infants.
**2.4 Study Design**	No previous text	All infants will receive Rotarix at 4, 8 and 12 weeks after the third study injection (study Days 84, 112 and 140), and fecal shedding of Rotarix will be assessed during the week after the first dose in a subset of infants.	Added in text to address the collection of additional stool samples after the Rotarix vaccinations.
New subsection inserted under Section **2.4 Study Design**	No previous text	2.4.3 Exploratory Objective:<br>Efficacy<br>• To evaluate the impact of the trivalent P2-VP8 subunit rotavirus vaccination on shedding of Rotarix subsequently administered in healthy South African infants as a test of concept	Evaluation of the impact of the trivalent vaccine on shedding of Rotarix added as an Exploratory Objective.
New subsection inserted under Section **2.4 Study Design**	No previous text	2.4.6 Exploratory Endpoint:<br>• Proportion of participants shedding Rotarix after administration of Rotarix “challenge	Assessment of fecal shedding of Rotarix in infants added as an Exploratory Endpoint.
New subsection inserted under Section **5.2.7 Groups C and D (infants)**	No previous text	5.2.7.6. Day 84 visit:<br>f) Parents will be provided an empty container to obtain stool sample on the morning of Day 89 (RMPRU only).	Distribution of a stool collection container (RMPRU only) added to visit activity listing.
New subsection inserted under Section **5.2.7 Groups C and D (infants)**	No previous text	5.2.7.7 Day 89 – Clinic visit for stool sampling (RMPRU only)<br>The parent will return to the clinic with a stool sample obtained that morning. If the parent is unable to bring the specimen to the clinic, a clinic staff member will visit the home to collect the sample. If the infant does not pass stool during the morning, a specimen will be obtained by rectal swab. Parents will be provided an empty container to obtain stool sample on the morning of Day 91.	Stool collection at Day 89 clinic visit added. Assessment of fecal shedding of Rotarix in infants to be conducted at the RMPRU site only.

List of VAC 041 Protocol Changes (version 3.0 to version 4.0)

Section in amended protocol	Old text	New/Revised text	Reason for change
New subsection inserted under **Section 5.2.7 Groups C and D (infants)**	No previous text	5.2.7.8 Day 91 – Clinic visit for stool sampling (RMPRU only)<br>The parent will return to the clinic with a stool sample obtained that morning. If the parent is unable to bring the specimen to the clinic, a clinic staff member will visit the home to collect the sample. If the infant does not pass stool during the morning, a specimen will be obtained by rectal swab. Parents will be provided an empty container to obtain stool sample on the morning of Day 93.	Stool collection at Day 91 clinic visit added. Assessment of fecal shedding of Rotarix in infants to be conducted at the RMPRU site only.
New subsection inserted under Section **5.2.7 Groups C and D (infants)**	No previous text	5.2.7.9 Day 93 – Clinic visit for stool sampling (RMPRU only)<br>The parent will return to the clinic with a stool sample obtained that morning. If the parent is unable to bring the specimen to the clinic, a clinic staff member will visit the home to collect the sample. If the infant does not pass stool during the morning, a specimen will be obtained by rectal swab. The next clinic visit is scheduled.	Stool collection at Day 93 clinic visit added. Assessment of fecal shedding of Rotarix in infants to be conducted at the RMPRU site only.

List of VAC 041 Protocol Changes (version 3.0 to version 4.0)

Section in amended protocol	Old text	New/Revised text	Reason for change
New subsection inserted under Section **6.0 Laboratory Evaluations**	No previous text	6.6 Rotarix Shedding<br>At one study site, RMPRU, stool samples will be obtained 5, 7 and 9 days following the first dose of Rotarix for qualitative assessment of vaccine virus shedding at NICD.<br>All stool samples will be tested for the presence of rotavirus using the commercially available ProsPecT™ Rotavirus Microplate Assay (Oxoid Ltd, Ely, United Kingdom), according to the manufacturer's instructions.<br>ELISA positive specimens may be confirmed and genotyped by PCR amplification of the VP7 and VP4 genes with a panel of G- and P-specific primers, respectively (WHO Manual of rotavirus detection and characterization methods, Methods 14 and 15 with minor modifications). The primer sets include: G-specific primer set (Beg9, End9, aAT8v, aBT1, aCT2, aDT4, mG3, mG9, mG10, mG12b and EndA) for the detection of G1, G2, G3, G4, G8, G9, G10 and G12 rotavirus strains and P-specific primer set (con2, con3, 1T-1v, 2T-1, 3T-1, 4T-1, 5T-1, mP11, p4943 and VP4F) for the detection of P[4], P[6], P[8], P[9], P[10], P[11] and P[14] rotavirus strains. Genotype G1 and/or P[8] rotavirus strains will be sequenced to determine if the strain is vaccine derived or a wild-type case. The VP7 (1062bp) and VP4 (876 bp) genes amplified by PCR will be purified and sequenced using the BigDye® Terminator v 3.1 cycle sequencing kits (Applied Biosystems). Reactions will be run on a 3500 Genetic Analyzer (Applied Biosystems) and data analyzed with manufacturer supplied software. Sequences will be directly compared to the VP7 and VP4 genes of the Rotarix strains (sequences available on Genbank; JN849114.1 VP7 and JN849113.1 VP4) and submitted to GenBank for BLASTn analyses to establish whether the VP7 and VP4 genes are from the vaccine or from wild-type G1P[8] strains.	Assessment of shedding of Rotarix following the first dose of Rotarix as a measure of efficacy added. Assessment to be conducted at the RMPRU site only.

List of VAC 041 Protocol Changes (version 3.0 to version 4.0)

Section in amended protocol	Old text	New/Revised text	Reason for change
Section 6.1, Sample collection, distribution and storage	Stool samples obtained from infants with gastroenteritis to assess for presence of rotavirus will be processed at the clinical trial site and transported to the laboratory at the National Institute for Communicable Disease, Sandringham, Johannesburg.	Deleted 'with gastroenteritis to assess for presence of rotavirus', sentence now reads ' Stool samples obtained from infants will be processed at the clinical trial site and transported to the laboratory at the National Institute for Communicable Disease, Sandringham, Johannesburg'.	Amended the language for stool collection to reflect only the collection and distribution procedure.
New subsection inserted under Section **7.3 Objectives and Endpoints**	No previous text	7.3.3 Exploratory Objective<br>Efficacy<br>To evaluate the impact of the trivalent P2-VP8 subunit rotavirus vaccine on shedding of Rotarix administered following P2-VP8 subunit rotavirus vaccination of healthy South African infants as a test of concept.<br>Endpoint:<br>• Proportion of participants with Rotarix vaccine virus detected in stools on Days 5-9 following the first dose of Rotarix, which is to be administered 28 days after the third study injection	Evaluation of the impact of the trivalent vaccine on shedding of Rotarix added as an Exploratory Objective. Detection of Rotarix vaccine virus in stools of infants on Days 5-9 added as an Endpoint.
New text inserted under Section **7.5 Analytical Methodology**	No previous text	Assessment of shedding of Rotarix virus will be performed for each of the three specified post-vaccination days and for shedding on any of the three days. Rates of shedding between the placebo and (1) each dose group and (2) the combined three dose groups will be compared using the Fisher's exact test.	Added specific timepoints (three specified post-vaccination days) for Rotarix shedding assessment.
**14.1 APPENDIX I: Schedule of Events**<br>**14.1.3. Schedule of Events for Groups C and D (Infants)**	No previous text	(New table column and row inserted) Clinic visit days 89, 91 and 93	Added clinic visit days 89, 91 and 93 for stool collections at (RMPRU site only.
